# The Buprestidae (Coleoptera, Buprestoidea) of the Tuscan Archipelago (Italy)

**DOI:** 10.3897/BDJ.12.e117362

**Published:** 2024-02-21

**Authors:** Leonardo Forbicioni, Nicola Tormen, Gianfranco Curletti, Luciano Bani, Andrea Di Giulio, Enrico Ruzzier

**Affiliations:** 1 World Biodiversity Association Onlus - Sezione Arcipelago Toscano, Portoferraio, Italy World Biodiversity Association Onlus - Sezione Arcipelago Toscano Portoferraio Italy; 2 World Biodiversity Association Onlus, Verona, Italy World Biodiversity Association Onlus Verona Italy; 3 Museo Civico di Storia Naturale, Carmagnola, Italy Museo Civico di Storia Naturale Carmagnola Italy; 4 University of Milano-Bicocca, Department of Earth and Environmental Sciences, Milan, Italy University of Milano-Bicocca, Department of Earth and Environmental Sciences Milan Italy; 5 National Biodiversity Future Center - NBFC, Palermo, Italy National Biodiversity Future Center - NBFC Palermo Italy; 6 Department of Science, Roma Tre University, Rome, Italy Department of Science, Roma Tre University Rome Italy

**Keywords:** biodiversity, faunistic, Jewel beetles, PNRR, wood borers

## Abstract

**Background:**

Buprestidae is a group of beetles of important conservation and phytosanitary value that is poorly studied in the Tuscan Archipelago and the limited faunistic knowledge available refers to a few scant historical records.

**New information:**

The present contribution increments the species documented in the Archipelago from 27 to 51, providing more than 300 georeferenced occurrence records, derived from both direct field research and citizen science via iNaturalist. Of particular importance is the discovery of *Eurythyreaquercus* on Isola d'Elba, an uncommon and localised species currently critically endangered.

## Introduction

The Tuscan Archipelago, inclusive of the Tuscan Archipelago National Park and recognised as Man and the Biosphere Reserve by UNESCO, is considered one of the major biodiversity hotspots of the Mediterranean Basin (Mittermeier et al. 2005). This Archipelago, rich in endemisms, Sardinian-Corsican elements and taxa originating from the Italian mainland, presents a rather complex biogeographic history linked to its origin during the convergence between Europe and Africa (Peccerillo 2017), the Pleistocene sea regression and driven by the changes that occurred in more recent times (Miocene to present), including those mediated by humans and their activities (Fattorini 2009, Ruzzier et al. 2021). The area has been the subject of extensive floristic, faunistic, and biogeographic studies in historical and more recent times; however, if we exclude vascular plants (e.g. Arrigoni et al. (2003), Foggi et al. (2014), Lazzaro et al. (2014), Carta et al. (2018)), vertebrates (e.g. Marinis et al. 1996, Angelici et al. 2009, Amori et al. 2015) and some selected groups of invertebrates (e.g. Dapporto and Cini (2007), Ceccolini et al. (2012), Rocchi et al. (2014), Fattorini et al. (2017), Barbato et al. (2018), Bellò and Ruzzier (2019), Ruzzier et al. (2020)), much still remains to be studied and important knowledge gaps exist regarding the richness and diversity of invertebrates on the various islands of the Archipelago. Coleoptera, in particular, represent a huge part of this hidden biodiversity and several families have never been addressed in their entirety. Buprestidae Leach, 1815 (Coleoptera, Buprestoidea) are a rather diverse beetle family counting more than 15,000 described species worldwide (Bellamy 2008), with more than 200 taxa recorded in Italy (Lobl and Lobl 2016). On the Tuscan Archipelago, the group has been poorly investigated, with only twenty-seven species recorded to date (Poggi 1976, Gobbi 1983, Curletti 1994, Curletti 2021). Buprestid beetles, thanks to their biology closely linked to plants, constitute an important element of biodiversity whose conservation is directly dependent on the protection of woodland and meadow environments. Many species are, in fact, included in the Red List of Saproxylic Beetles at both the Italian and European levels, demonstrating their vulnerability to habitat loss, degradation and fragmentation (Nieto and Alexander 2010, Audisio et al. 2014). In addition, several Buprestidae are of phytosanitary relevance (e.g. Evans et al. (2004), Sever et al. (2012), Ohsawa (2017), Jendek et al. (2018)) and interest in them grows with a view to protection and prevention against the introduction of non-native species (Ruzzier et al. 2023). The main objective of this contribution is, therefore, to update the knowledge status of the Buprestidae of the Tuscan Archipelago, providing up-to-date distribution data of the species on the various islands and to highlight, where possible, important elements of value or any critical issue.

## Materials and methods

Specimens cited in this paper are deposited in the following collections: LFPC - Leonardo Forbicioni private collection, NatLab Museum, Portoferraio (LI, Italy); MGPC - Maurizio Gigli private collection, Guidonia (Rome, Italy); SNPC - Stefano Nappini private collection, Castiglion della Pescaia (GR, Italy); MBPC - Marco Bastianini private collection Gavorrano (GR, Italy); ABPC - Andrea Beltramini private collection, Firenze (FI, Italy). The IUCN Red List categorisation for saproxylic species used in this manuscript follows that of Audisio et al. (2014). Since there is no unanimous interpretation of the use and validity of *Agrilus* subgenrea, we refrain from their use in this paper.

### Study area

The Archipelago, located in the northern part of the Tyrrhenian Sea, sits at about halfway between Corsica and the Italian Peninsula and consists of seven main islands (Elba, Giglio, Capraia, Montecristo, Pianosa, Giannutri and Gorgona) and a series of islets and skerries.

## Checklists

### Checklist of the Buprestidae of the Tuscan Archipelago

#### Agrilinae

82709D15-0694-5F85-8035-F9C3451B41D3

#### Agrilini

E4DA2E7E-B23E-5AD8-AE8C-E123467D9BF4

#### Agrilus angustulus angustulus

(Illiger, 1803)

FA883DC0-8B80-5ECA-B30B-F98315724BD7

##### Materials

- **Type status:** Other material. **Occurrence:** recordedBy: Leonardo Forbicioni; individualCount: 1; lifeStage: adult; occurrenceID: 79722C5C-555D-5AE1-BF89-3836796E5107; **Taxon:** scientificName: Agrilusangustulusangustulus (Illiger, 1803); order: Coleoptera; family: Buprestidae; genus: Agrilus; subgenus: Anambus; specificEpithet: angustulus; infraspecificEpithet: angustulus; scientificNameAuthorship: (Illiger, 1803); **Location:** islandGroup: Tuscan Archipelago; island: Isola d'Elba; country: Italy; countryCode: IT; stateProvince: Livorno; county: Portoferraio; locality: Colle Reciso/Mulino a vento; decimalLatitude: 42.779015; decimalLongitude: 10.293692; geodeticDatum: WGS84; coordinatePrecision: 0.0002; **Identification:** identifiedBy: G. Curletti; **Event:** eventDate: 2013-06-17; **Record Level:** collectionCode: LFPC
- **Type status:** Other material. **Occurrence:** recordedBy: Leonardo Forbicioni; individualCount: 1; lifeStage: adult; occurrenceID: F9CEE26F-99AF-500A-A1C2-D709317C3F72; **Taxon:** scientificName: Agrilusangustulusangustulus (Illiger, 1803); order: Coleoptera; family: Buprestidae; genus: Agrilus; specificEpithet: angustulus; infraspecificEpithet: angustulus; scientificNameAuthorship: (Illiger, 1803); **Location:** islandGroup: Tuscan Archipelago; island: Isola d'Elba; country: Italy; countryCode: IT; stateProvince: Livorno; county: Porto Azzurro; locality: Buraccio; decimalLatitude: 42.779458; decimalLongitude: 10.354036; geodeticDatum: WGS84; coordinatePrecision: 0.0002; **Identification:** identifiedBy: G. Curletti; **Event:** eventDate: 2013-05-21; **Record Level:** collectionCode: LFPC
- **Type status:** Other material. **Occurrence:** recordedBy: Leonardo Forbicioni; individualCount: 11; lifeStage: adult; occurrenceID: 34A75325-5249-5268-96D6-4288D064E28F; **Taxon:** scientificName: Agrilusangustulusangustulus (Illiger, 1803); order: Coleoptera; family: Buprestidae; genus: Agrilus; specificEpithet: angustulus; infraspecificEpithet: angustulus; scientificNameAuthorship: (Illiger, 1803); **Location:** islandGroup: Tuscan Archipelago; island: Isola d'Elba; country: Italy; countryCode: IT; stateProvince: Livorno; county: Campo nell'Elba; municipality: San Piero; decimalLatitude: 42.761243; decimalLongitude: 10.208388; geodeticDatum: WGS84; coordinatePrecision: 0.0002; **Identification:** identifiedBy: E. Paggetti, F. Terzani & F. Ceccolini; **Event:** eventDate: 2012-06-10; **Record Level:** collectionCode: LFPC
- **Type status:** Other material. **Occurrence:** recordedBy: Leonardo Forbicioni; individualCount: 1; lifeStage: adult; occurrenceID: CF294548-9C59-5AC2-82FF-53178CDB6DDB; **Taxon:** scientificName: Agrilusangustulusangustulus (Illiger, 1803); order: Coleoptera; family: Buprestidae; genus: Agrilus; specificEpithet: angustulus; infraspecificEpithet: angustulus; scientificNameAuthorship: (Illiger, 1803); **Location:** islandGroup: Tuscan Archipelago; island: Isola d'Elba; country: Italy; countryCode: IT; stateProvince: Livorno; county: Portoferraio; locality: Monte Orello; decimalLatitude: 42.780249; decimalLongitude: 10.327917; geodeticDatum: WGS84; coordinatePrecision: 0.0002; **Identification:** identifiedBy: F. Rosso; **Event:** eventDate: 2017-06-04; **Record Level:** collectionCode: LFPC
- **Type status:** Other material. **Occurrence:** recordedBy: Leonardo Forbicioni; individualCount: 2; lifeStage: adult; occurrenceID: E8300EC8-B3C4-5A24-8407-24FCDC0B1458; **Taxon:** scientificName: Agrilusangustulusangustulus (Illiger, 1803); order: Coleoptera; family: Buprestidae; genus: Agrilus; specificEpithet: angustulus; infraspecificEpithet: angustulus; scientificNameAuthorship: (Illiger, 1803); **Location:** islandGroup: Tuscan Archipelago; island: Isola d'Elba; country: Italy; countryCode: IT; stateProvince: Livorno; county: Portoferraio; locality: Acquabona; decimalLatitude: 42.786235; decimalLongitude: 10.346564; geodeticDatum: WGS84; coordinatePrecision: 0.0002; **Identification:** identifiedBy: E. Paggetti, F. Terzani & F. Ceccolini; **Event:** eventDate: 2011-05-26; **Record Level:** collectionCode: LFPC
- **Type status:** Other material. **Occurrence:** recordedBy: Leonardo Forbicioni; individualCount: 1; lifeStage: adult; occurrenceID: CABCE4C6-DE3D-58AE-B60E-ED0B713AC586; **Taxon:** scientificName: Agrilusangustulusangustulus (Illiger, 1803); order: Coleoptera; family: Buprestidae; genus: Agrilus; specificEpithet: angustulus; infraspecificEpithet: angustulus; scientificNameAuthorship: (Illiger, 1803); **Location:** islandGroup: Tuscan Archipelago; island: Isola d'Elba; country: Italy; countryCode: IT; stateProvince: Livorno; county: Campo nell'Elba; locality: Monte Perone; decimalLatitude: 42.761458; decimalLongitude: 10.198383; geodeticDatum: WGS84; coordinatePrecision: 0.0002; **Identification:** identifiedBy: E. Paggetti, F. Terzani & F. Ceccolini; **Event:** eventDate: 2011-06-19; **Record Level:** collectionCode: LFPC
- **Type status:** Other material. **Occurrence:** recordedBy: Leonardo Forbicioni; individualCount: 2; lifeStage: adult; occurrenceID: 5BDECEBD-6D64-5C7C-8696-BC710AD8BA2E; **Taxon:** scientificName: Agrilusangustulusangustulus (Illiger, 1803); order: Coleoptera; family: Buprestidae; genus: Agrilus; specificEpithet: angustulus; infraspecificEpithet: angustulus; scientificNameAuthorship: (Illiger, 1803); **Location:** islandGroup: Tuscan Archipelago; island: Isola d'Elba; country: Italy; countryCode: IT; stateProvince: Livorno; county: Campo nell'Elba; locality: Monte Perone/Masso alla Quata; decimalLatitude: 42.761703; decimalLongitude: 10.185286; geodeticDatum: WGS84; coordinatePrecision: 0.0002; **Identification:** identifiedBy: F. Rosso; G. Curletti; **Event:** eventDate: 2020-06-21; **Record Level:** collectionCode: LFPC
- **Type status:** Other material. **Occurrence:** individualCount: 1; lifeStage: adult; occurrenceID: EA14BE24-2CA9-5D55-BF3E-CCDF7870FFA7; **Taxon:** scientificName: Agrilusangustulusangustulus (Illiger, 1803); order: Coleoptera; family: Buprestidae; genus: Agrilus; specificEpithet: angustulus; infraspecificEpithet: angustulus; scientificNameAuthorship: (Illiger, 1803); **Location:** islandGroup: Tuscan Archipelago; island: Isola d'Elba; country: Italy; countryCode: IT; stateProvince: Livorno; **Identification:** identifiedBy: G. Curletti; **Record Level:** source: Curletti G. (1994) I Buprestidi d’Italia. Catalogo geonemico, sinonimico, bibliografico, biologico. Monografie di Natura Bresciana, Ed. Vannini, Brescia, 19.

##### Conservation status

LC

##### Distribution

Recorded for the Tuscan Archipelago (Isola d'Elba) by Curletti (1994).

#### Agrilus cuprescens

(Ménétriés, 1832)

9358B60B-7C3B-5D28-B032-A365FB6C6499

##### Materials

- **Type status:** Other material. **Occurrence:** recordedBy: Leonardo Forbicioni; individualCount: 1; lifeStage: adult; occurrenceID: 4836DC0D-BB2C-55B9-9B3E-E7936FB60182; **Taxon:** scientificName: Agriluscuprescens (Ménétriés, 1832); order: Coleoptera; family: Buprestidae; genus: Agrilus; specificEpithet: cuprescens; scientificNameAuthorship: (Ménétriés, 1832); **Location:** islandGroup: Tuscan Archipelago; island: Isola d'Elba; country: Italy; countryCode: IT; stateProvince: Livorno; county: Porto Azzurro; locality: Buraccio; decimalLatitude: 42.779661; decimalLongitude: 10.356152; geodeticDatum: WGS84; coordinatePrecision: 0.0002; **Identification:** identifiedBy: G. Curletti; **Event:** eventDate: 2022-06-13; **Record Level:** collectionCode: LFPC

#### Agrilus cyanescens

(Ratzeburg, 1837)

08B61AFF-0BBC-575E-B27B-030E9411C2FD

##### Materials

- **Type status:** Other material. **Occurrence:** recordedBy: Leonardo Forbicioni; individualCount: 1; lifeStage: adult; occurrenceID: 48F65216-44B3-5286-AE89-425C1CB8CB34; **Taxon:** scientificName: Agriluscyanescens (Ratzeburg, 1837); order: Coleoptera; family: Buprestidae; genus: Agrilus; specificEpithet: cyanescens; scientificNameAuthorship: (Ratzeburg, 1837); **Location:** islandGroup: Tuscan Archipelago; island: Isola d'Elba; country: Italy; countryCode: IT; stateProvince: Livorno; county: Portoferraio; locality: Colle Reciso; decimalLatitude: 42.785982; decimalLongitude: 10.318454; geodeticDatum: WGS84; coordinatePrecision: 0.0002; **Identification:** identifiedBy: G. Curletti; **Event:** eventDate: 2010-05-28; **Record Level:** collectionCode: LFPC

##### Conservation status

LC

#### Agrilus derasofasciatus

Lacordaire, 1835

AC32FFB4-61A5-514A-8F62-04554741176C

##### Materials

- **Type status:** Other material. **Occurrence:** recordedBy: Leonardo Forbicioni; individualCount: 1; lifeStage: adult; occurrenceID: 572015ED-8C86-5F5E-8DB6-122542AA003D; **Taxon:** scientificName: Agrilusderasofasciatus Lacordaire, 1835; order: Coleoptera; family: Buprestidae; genus: Agrilus; specificEpithet: derasofasciatus; scientificNameAuthorship: Lacordaire, 1835; **Location:** islandGroup: Tuscan Archipelago; island: Isola d'Elba; country: Italy; countryCode: IT; stateProvince: Livorno; county: Portoferraio; locality: Monte Orello/Le Picchiaie; decimalLatitude: 42.786263; decimalLongitude: 10.328854; geodeticDatum: WGS84; coordinatePrecision: 0.0002; **Identification:** identifiedBy: E. Paggetti & F. Terzani; **Event:** eventDate: 2011-07-03; **Record Level:** collectionCode: LFPC
- **Type status:** Other material. **Occurrence:** recordedBy: Leonardo Forbicioni; individualCount: 1; lifeStage: adult; occurrenceID: D3EB5EA9-5192-557A-9C3A-D644F2CC9DBF; **Taxon:** scientificName: Agrilusderasofasciatus Lacordaire, 1835; order: Coleoptera; family: Buprestidae; genus: Agrilus; specificEpithet: derasofasciatus; scientificNameAuthorship: Lacordaire, 1835; **Location:** islandGroup: Tuscan Archipelago; island: Isola d'Elba; country: Italy; countryCode: IT; stateProvince: Livorno; county: Portoferraio; locality: Monte Orello/Le Picchiaie; decimalLatitude: 42.788797; decimalLongitude: 10.328287; geodeticDatum: WGS84; coordinatePrecision: 0.0002; **Identification:** identifiedBy: E. Paggetti; **Event:** eventDate: 2011-07-03; **Record Level:** collectionCode: LFPC
- **Type status:** Other material. **Occurrence:** recordedBy: Leonardo Forbicioni; individualCount: 1; lifeStage: adult; occurrenceID: D15403B1-FBE3-50CD-AB73-B60A1F0DC210; **Taxon:** scientificName: Agrilusderasofasciatus Lacordaire, 1835; order: Coleoptera; family: Buprestidae; genus: Agrilus; specificEpithet: derasofasciatus; scientificNameAuthorship: Lacordaire, 1835; **Location:** islandGroup: Tuscan Archipelago; island: Isola d'Elba; country: Italy; countryCode: IT; stateProvince: Livorno; county: Capoliveri; locality: Norsi; decimalLatitude: 42.769745; decimalLongitude: 10.346316; geodeticDatum: WGS84; coordinatePrecision: 0.0002; **Identification:** identifiedBy: E. Paggetti; **Event:** eventDate: 2014-06-18; **Record Level:** collectionCode: LFPC
- **Type status:** Other material. **Occurrence:** recordedBy: Leonardo Forbicioni; individualCount: 1; lifeStage: adult; occurrenceID: 17CD7FCD-D702-5F4C-B95C-C3F60B0FBA52; **Taxon:** scientificName: Agrilusderasofasciatus Lacordaire, 1835; order: Coleoptera; family: Buprestidae; genus: Agrilus; specificEpithet: derasofasciatus; scientificNameAuthorship: Lacordaire, 1835; **Location:** islandGroup: Tuscan Archipelago; island: Isola d'Elba; country: Italy; countryCode: IT; stateProvince: Livorno; county: Capoliveri; locality: Pian di Mola; decimalLatitude: 42.758896; decimalLongitude: 10.365985; geodeticDatum: WGS84; coordinatePrecision: 0.0002; **Identification:** identifiedBy: L. Forbicioni; **Event:** eventDate: 2013-07-02; **Record Level:** collectionCode: LFPC
- **Type status:** Other material. **Occurrence:** recordedBy: Leonardo Forbicioni; individualCount: 1; lifeStage: adult; occurrenceID: 9C2FC543-7981-5022-833C-DB87ED289FD4; **Taxon:** scientificName: Agrilusderasofasciatus Lacordaire, 1835; order: Coleoptera; family: Buprestidae; genus: Agrilus; specificEpithet: derasofasciatus; scientificNameAuthorship: Lacordaire, 1835; **Location:** islandGroup: Tuscan Archipelago; island: Isola d'Elba; country: Italy; countryCode: IT; stateProvince: Livorno; county: Portoferraio; locality: Acquabona; decimalLatitude: 42.785964; decimalLongitude: 10.346364; geodeticDatum: WGS84; coordinatePrecision: 0.0002; **Identification:** identifiedBy: F. Rosso; **Event:** eventDate: 2022-06-15; **Record Level:** collectionCode: LFPC
- **Type status:** Other material. **Occurrence:** individualCount: 1; lifeStage: adult; occurrenceID: 7348C44D-B955-5AC1-BF0C-D96EBE74A08C; **Taxon:** scientificName: Agrilusderasofasciatus Lacordaire, 1835; order: Coleoptera; family: Buprestidae; genus: Agrilus; subgenus: Anambus; specificEpithet: derasofasciatus; scientificNameAuthorship: Lacordaire, 1835; **Location:** islandGroup: Tuscan Archipelago; island: Isola d'Elba; country: Italy; countryCode: IT; stateProvince: Livorno; county: Rio; municipality: Rio Marina; locality: Ortano; **Identification:** identifiedBy: G. Curletti; **Record Level:** source: Curletti G. (1994) I Buprestidi d’Italia. Catalogo geonemico, sinonimico, bibliografico, biologico. Monografie di Natura Bresciana, Ed. Vannini, Brescia, 19.

##### Conservation status

LC

##### Distribution

Recorded for the Tuscan Archipelago (Isola d'Elba) by Curletti (1994).

#### Agrilus elegans elegans

Mulsant & Rey, 1863

0760783E-B1C9-51DB-9340-9D8D94E6FA53

##### Materials

- **Type status:** Other material. **Occurrence:** recordedBy: Leonardo Forbicioni; individualCount: 1; lifeStage: adult; occurrenceID: 253283D9-191C-58E4-AFA8-C8D044512892; **Taxon:** scientificName: Agriluseleganselegans Mulsant & Rey, 1863; order: Coleoptera; family: Buprestidae; genus: Agrilus; specificEpithet: elegans; infraspecificEpithet: elegans; scientificNameAuthorship: Mulsant & Rey, 1863; **Location:** islandGroup: Tuscan Archipelago; island: Isola di Pianosa; country: Italy; countryCode: IT; stateProvince: Livorno; county: Pianosa; locality: Grotta delle Vacche; decimalLatitude: 42.596118; decimalLongitude: 10.091151; geodeticDatum: WGS84; coordinatePrecision: 0.0002; **Identification:** identifiedBy: E. Paggetti; **Event:** eventDate: 2014-05-06; **Record Level:** collectionCode: LFPC
- **Type status:** Other material. **Occurrence:** recordedBy: Leonardo Forbicioni; individualCount: 1; lifeStage: adult; occurrenceID: 8DB632BC-49A2-5074-AB69-D72F88841005; **Taxon:** scientificName: Agriluseleganselegans Mulsant & Rey, 1863; order: Coleoptera; family: Buprestidae; genus: Agrilus; specificEpithet: elegans; infraspecificEpithet: elegans; scientificNameAuthorship: Mulsant & Rey, 1863; **Location:** islandGroup: Tuscan Archipelago; island: Isola del Giglio; country: Italy; countryCode: IT; stateProvince: Grosseto; county: Campese; locality: Mezzo Franco; decimalLatitude: 42.361040; decimalLongitude: 10.876393; geodeticDatum: WGS84; coordinatePrecision: 0.0002; **Identification:** identifiedBy: E. Paggetti; **Event:** eventDate: 17-19/05/2012; **Record Level:** collectionCode: LFPC

##### Conservation status

LC

#### Agrilus etruscus

Curletti, 2013

711C970C-F605-589A-995E-D346AE655325

##### Materials

- **Type status:** Other material. **Occurrence:** recordedBy: Leonardo Forbicioni; individualCount: 2; lifeStage: adult; occurrenceID: 492BCB60-40CC-55EE-80EB-A2FD877CFA66; **Taxon:** scientificName: Agrilusetruscus Curletti, 2013; order: Coleoptera; family: Buprestidae; genus: Agrilus; specificEpithet: etruscus; scientificNameAuthorship: Curletti, 2013; **Location:** islandGroup: Tuscan Archipelago; island: Isola d'Elba; country: Italy; countryCode: IT; stateProvince: Livorno; county: Portoferraio; locality: Acquabona; decimalLatitude: 42.786136; decimalLongitude: 10.345748; geodeticDatum: WGS84; coordinatePrecision: 0.0002; **Identification:** identifiedBy: G. Curletti; **Event:** eventDate: 2012-06-02; **Record Level:** collectionCode: LFPC
- **Type status:** Other material. **Occurrence:** recordedBy: Leonardo Forbicioni; individualCount: 1; lifeStage: adult; occurrenceID: 51A3FE2D-338C-5A60-9EEE-5BCF85BE1610; **Taxon:** scientificName: Agrilusetruscus Curletti, 2013; order: Coleoptera; family: Buprestidae; genus: Agrilus; specificEpithet: etruscus; scientificNameAuthorship: Curletti, 2013; **Location:** islandGroup: Tuscan Archipelago; island: Isola d'Elba; country: Italy; countryCode: IT; stateProvince: Livorno; county: Portoferraio; locality: Volterraio; decimalLatitude: 42.803537; decimalLongitude: 10.390625; geodeticDatum: WGS84; coordinatePrecision: 0.0002; **Identification:** identifiedBy: L. Forbicioni; **Event:** eventDate: 2011-06-15; **Record Level:** collectionCode: LFPC
- **Type status:** Other material. **Occurrence:** recordedBy: Leonardo Forbicioni; individualCount: 1; lifeStage: adult; occurrenceID: E21C7D7C-F5D6-5526-A259-DC925A350E74; **Taxon:** scientificName: Agrilusetruscus Curletti, 2013; order: Coleoptera; family: Buprestidae; genus: Agrilus; specificEpithet: etruscus; scientificNameAuthorship: Curletti, 2013; **Location:** islandGroup: Tuscan Archipelago; island: Isola d'Elba; country: Italy; countryCode: IT; stateProvince: Livorno; county: Portoferraio; locality: Colle Reciso/Buca di Bomba; decimalLatitude: 42.779893; decimalLongitude: 10.272784; geodeticDatum: WGS84; coordinatePrecision: 0.0002; **Identification:** identifiedBy: L. Forbicioni; **Event:** eventDate: 2011-07-03; **Record Level:** collectionCode: LFPC
- **Type status:** Other material. **Occurrence:** recordedBy: Leonardo Forbicioni; individualCount: 1; lifeStage: adult; occurrenceID: E3252F88-4AAD-57D4-8D09-A39A3C271719; **Taxon:** scientificName: Agrilusetruscus Curletti, 2013; order: Coleoptera; family: Buprestidae; genus: Agrilus; specificEpithet: etruscus; scientificNameAuthorship: Curletti, 2013; **Location:** islandGroup: Tuscan Archipelago; island: Isola d'Elba; country: Italy; countryCode: IT; stateProvince: Livorno; county: Capoliveri; locality: Monte Calamita/Costa dei Gabbiani; decimalLatitude: 42.731903; decimalLongitude: 10.417231; geodeticDatum: WGS84; coordinatePrecision: 0.0002; **Identification:** identifiedBy: L. Forbicioni; **Event:** eventDate: 2013-06-28; **Record Level:** collectionCode: LFPC
- **Type status:** Other material. **Occurrence:** recordedBy: Leonardo Forbicioni; individualCount: 1; lifeStage: adult; occurrenceID: AB6F8297-F1B0-5097-8305-D72CE22D65B9; **Taxon:** scientificName: Agrilusetruscus Curletti, 2013; order: Coleoptera; family: Buprestidae; genus: Agrilus; specificEpithet: etruscus; scientificNameAuthorship: Curletti, 2013; **Location:** islandGroup: Tuscan Archipelago; island: Isola d'Elba; country: Italy; countryCode: IT; stateProvince: Livorno; county: Portoferraio; locality: Monte Poppe; decimalLatitude: 42.797959; decimalLongitude: 10.275927; geodeticDatum: WGS84; coordinatePrecision: 0.0002; **Identification:** identifiedBy: L. Forbicioni; **Event:** eventDate: 2011-06-20; **Record Level:** collectionCode: LFPC

#### Agrilus evocatus

Curletti, 2021

0E1CD8AA-F63E-5746-9DCF-59D812E63422

##### Materials

- **Type status:** Other material. **Occurrence:** recordedBy: Regalin; individualCount: 4; lifeStage: adult; occurrenceID: 8FD56B33-0435-5C77-8E9E-A7AF9C9A6D95; **Taxon:** scientificName: Agrilusevocatus Curletti, 2021; order: Coleoptera; family: Buprestidae; genus: Agrilus; subgenus: Agrilus; scientificNameAuthorship: Curletti, 2021; **Location:** islandGroup: Tuscan Archipelago; island: Isola di Capraia; country: Italy; countryCode: IT; stateProvince: Livorno; county: Capraia; locality: Il Piano; **Identification:** identifiedBy: G. Curletti; **Event:** eventDate: 1993-06-27

##### Distribution

A recently described Italian endemism, known in the Tuscan Archipelago only for Capraia (Curletti 2021).

#### Agrilus hyperici

(Creutzer, 1799)

E25FCFD5-C941-5C06-898F-D925AB8B84AA

##### Materials

- **Type status:** Other material. **Occurrence:** recordedBy: Leonardo Forbicioni; individualCount: 1; lifeStage: adult; occurrenceID: 7F50A89A-B43B-5F62-A9E3-3D3480912B76; **Taxon:** scientificName: Agrilushyperici (Creutzer, 1799); order: Coleoptera; family: Buprestidae; genus: Agrilus; specificEpithet: hyperici; scientificNameAuthorship: Creutzer, 1799); **Location:** islandGroup: Tuscan Archipelago; island: Isola d'Elba; country: Italy; countryCode: IT; stateProvince: Livorno; county: Portoferraio; locality: Volterraio; decimalLatitude: 42.803537; decimalLongitude: 10.390625; geodeticDatum: WGS84; coordinatePrecision: 0.0002; **Identification:** identifiedBy: G. Curletti; **Event:** eventDate: 2011-06-15; **Record Level:** collectionCode: LFPC

#### Agrilus integerrimus

(Ratzeburg, 1837)

BC8FFA08-0CD1-5F5A-99E3-03132F69AD05

##### Materials

- **Type status:** Other material. **Occurrence:** recordedBy: Leonardo Forbicioni; individualCount: 1; lifeStage: adult; occurrenceID: 3A925407-46A1-5B0E-A70F-8D1B7B94EA30; **Taxon:** scientificName: Agrilusintegerrimus (Ratzeburg, 1837); order: Coleoptera; family: Buprestidae; genus: Agrilus; specificEpithet: integerrimus; scientificNameAuthorship: (Ratzeburg, 1837); **Location:** islandGroup: Tuscan Archipelago; island: Isola d'Elba; country: Italy; countryCode: IT; stateProvince: Livorno; county: Portoferraio; locality: Acquabona; decimalLatitude: 42.785997; decimalLongitude: 10.346347; geodeticDatum: WGS84; coordinatePrecision: 0.0002; **Identification:** identifiedBy: E. Paggetti; **Event:** eventDate: 2012-06-02; **Record Level:** collectionCode: LFPC
- **Type status:** Other material. **Occurrence:** recordedBy: Leonardo Forbicioni; individualCount: 1; lifeStage: adult; occurrenceID: 5599B273-75AC-5AD7-98C3-CD1F6659A764; **Taxon:** scientificName: Agrilusintegerrimus (Ratzeburg, 1837); order: Coleoptera; family: Buprestidae; genus: Agrilus; specificEpithet: integerrimus; scientificNameAuthorship: (Ratzeburg, 1837); **Location:** islandGroup: Tuscan Archipelago; island: Isola d'Elba; country: Italy; countryCode: IT; stateProvince: Livorno; county: Portoferraio; locality: Volterraio; decimalLatitude: 42.802771; decimalLongitude: 10.389644; geodeticDatum: WGS84; coordinatePrecision: 0.0002; **Identification:** identifiedBy: G. Curletti; **Event:** eventDate: 2011-06-15; **Record Level:** collectionCode: LFPC

#### Agrilus laticornis

(Illiger, 1803)

798A8D63-ACCA-5AA7-B1BB-CA49153DCC1A

##### Materials

- **Type status:** Other material. **Occurrence:** recordedBy: Leonardo Forbicioni; individualCount: 1; lifeStage: adult; occurrenceID: 07B3E878-7D27-5A17-9729-5FCCAA7ECE23; **Taxon:** scientificName: Agriluslaticornis (Illiger, 1803); order: Coleoptera; family: Buprestidae; genus: Agrilus; specificEpithet: laticornis; scientificNameAuthorship: (Illiger, 1803); **Location:** islandGroup: Tuscan Archipelago; island: Isola d'Elba; country: Italy; countryCode: IT; stateProvince: Livorno; county: Portoferraio; locality: Acquaviva; decimalLatitude: 42.819109; decimalLongitude: 10.287890; geodeticDatum: WGS84; coordinatePrecision: 0.0002; **Identification:** identifiedBy: E. Paggetti; **Event:** eventDate: 2014-06-18; **Record Level:** collectionCode: LFPC

##### Conservation status

LC

#### Agrilus marozzinii

Gobbi, 1974

B033FCCB-CDBA-5327-A02B-BEE9A1406681

##### Materials

- **Type status:** Other material. **Occurrence:** recordedBy: Leonardo Forbicioni; individualCount: 1; lifeStage: adult; occurrenceID: F6C6941B-4D0A-54F9-942A-06697C21EB73; **Taxon:** scientificName: Agrilusmarozzinii Gobbi, 1974; order: Coleoptera; family: Buprestidae; genus: Agrilus; specificEpithet: marozzinii; scientificNameAuthorship: Gobbi, 1974; **Location:** islandGroup: Tuscan Archipelago; island: Isola d'Elba; country: Italy; countryCode: IT; stateProvince: Livorno; county: Portoferraio; locality: Colle Reciso/Mulino a vento; decimalLatitude: 42.778966; decimalLongitude: 10.294266; geodeticDatum: WGS84; coordinatePrecision: 0.0002; **Identification:** identifiedBy: M. Gigli; **Event:** eventDate: 2015-06-27; **Record Level:** collectionCode: MGPC

##### Conservation status

LC

#### Agrilus roscidus

Kiesenwetter, 1857

391AC342-E2D3-57A6-96A9-14CA44E6B282

##### Materials

- **Type status:** Other material. **Occurrence:** recordedBy: Leonardo Forbicioni; individualCount: 1; lifeStage: adult; occurrenceID: EE7952F3-8BEC-58F7-8775-ED0649D49DF0; **Taxon:** scientificName: Agrilusroscidus Kiesenwetter, 1857; order: Coleoptera; family: Buprestidae; genus: Agrilus; specificEpithet: roscidus; scientificNameAuthorship: Kiesenwetter, 1857; **Location:** islandGroup: Tuscan Archipelago; island: Isola d'Elba; country: Italy; countryCode: IT; stateProvince: Livorno; county: Portoferraio; locality: Schiopparello/Le Prade; decimalLatitude: 42.794858; decimalLongitude: 10.349664; geodeticDatum: WGS84; coordinatePrecision: 0.0002; **Identification:** identifiedBy: G. Curletti; **Event:** eventDate: 2011-06-17; **Record Level:** collectionCode: LFPC
- **Type status:** Other material. **Occurrence:** recordedBy: Leonardo Forbicioni; individualCount: 2; lifeStage: adult; occurrenceID: BC79C171-1E05-5D43-A0CC-8A971D1AB60E; **Taxon:** scientificName: Agrilusroscidus Kiesenwetter, 1857; order: Coleoptera; family: Buprestidae; genus: Agrilus; specificEpithet: roscidus; scientificNameAuthorship: Kiesenwetter, 1857; **Location:** islandGroup: Tuscan Archipelago; island: Isola d'Elba; country: Italy; countryCode: IT; stateProvince: Livorno; county: Capoliveri; locality: Pian di Mola; decimalLatitude: 42.759336; decimalLongitude: 10.365624; geodeticDatum: WGS84; coordinatePrecision: 0.0002; **Identification:** identifiedBy: G. Curletti; **Event:** eventDate: 2011-06-26; **Record Level:** collectionCode: LFPC
- **Type status:** Other material. **Occurrence:** recordedBy: Leonardo Forbicioni; individualCount: 4; lifeStage: adult; occurrenceID: CFD049DB-1330-5000-BEF2-D07A71160690; **Taxon:** scientificName: Agrilusroscidus Kiesenwetter, 1857; order: Coleoptera; family: Buprestidae; genus: Agrilus; specificEpithet: roscidus; scientificNameAuthorship: Kiesenwetter, 1857; **Location:** islandGroup: Tuscan Archipelago; island: Isola d'Elba; country: Italy; countryCode: IT; stateProvince: Livorno; county: Porto Azzurro; locality: Buraccio; decimalLatitude: 42.780219; decimalLongitude: 10.352051; geodeticDatum: WGS84; coordinatePrecision: 0.0002; **Identification:** identifiedBy: E. Paggetti & F. Ceccolini; **Event:** eventDate: 2013-05-21; **Record Level:** collectionCode: LFPC
- **Type status:** Other material. **Occurrence:** recordedBy: Leonardo Forbicioni; individualCount: 2; lifeStage: adult; occurrenceID: DF881243-394A-54B2-96F2-2D444833DA96; **Taxon:** scientificName: Agrilusroscidus Kiesenwetter, 1857; order: Coleoptera; family: Buprestidae; genus: Agrilus; specificEpithet: roscidus; scientificNameAuthorship: Kiesenwetter, 1857; **Location:** islandGroup: Tuscan Archipelago; island: Isola d'Elba; country: Italy; countryCode: IT; stateProvince: Livorno; county: Capoliveri; locality: Straccoligno/Buzzancone; decimalLatitude: 42.742500; decimalLongitude: 10.408330; geodeticDatum: WGS84; coordinatePrecision: 0.0002; **Identification:** identifiedBy: E. Paggetti; **Event:** eventDate: 2014-07-02; **Record Level:** collectionCode: LFPC
- **Type status:** Other material. **Occurrence:** recordedBy: Leonardo Forbicioni; individualCount: 1; lifeStage: adult; occurrenceID: 3BFDA581-597A-5945-9314-B7BAF23649E9; **Taxon:** scientificName: Agrilusroscidus Kiesenwetter, 1857; order: Coleoptera; family: Buprestidae; genus: Agrilus; specificEpithet: roscidus; scientificNameAuthorship: Kiesenwetter, 1857; **Location:** islandGroup: Tuscan Archipelago; island: Isola d'Elba; country: Italy; countryCode: IT; stateProvince: Livorno; county: Capoliveri; locality: Pian di Mola; decimalLatitude: 42.758896; decimalLongitude: 10.365985; geodeticDatum: WGS84; coordinatePrecision: 0.0002; **Identification:** identifiedBy: G. Curletti; **Event:** eventDate: 2013-07-02; **Record Level:** collectionCode: LFPC
- **Type status:** Other material. **Occurrence:** recordedBy: Leonardo Forbicioni; individualCount: 1; lifeStage: adult; occurrenceID: 51E71E73-0DB1-5C47-9ACF-6121567BC15E; **Taxon:** scientificName: Agrilusroscidus Kiesenwetter, 1857; order: Coleoptera; family: Buprestidae; genus: Agrilus; specificEpithet: roscidus; scientificNameAuthorship: Kiesenwetter, 1857; **Location:** islandGroup: Tuscan Archipelago; island: Isola d'Elba; country: Italy; countryCode: IT; stateProvince: Livorno; county: Porto Azzurro; locality: Buraccio/Lo Stipito; decimalLatitude: 42.762166; decimalLongitude: 10.365712; geodeticDatum: WGS84; coordinatePrecision: 0.0002; **Identification:** identifiedBy: G. Curletti; **Event:** eventDate: 2011-06-30; **Record Level:** collectionCode: LFPC
- **Type status:** Other material. **Occurrence:** recordedBy: Leonardo Forbicioni; individualCount: 1; lifeStage: adult; occurrenceID: 8266F48A-1B5F-5E2F-81D1-9FBB35483462; **Taxon:** scientificName: Agrilusroscidus Kiesenwetter, 1857; order: Coleoptera; family: Buprestidae; genus: Agrilus; specificEpithet: roscidus; scientificNameAuthorship: Kiesenwetter, 1857; **Location:** islandGroup: Tuscan Archipelago; island: Isola d'Elba; country: Italy; countryCode: IT; stateProvince: Livorno; county: Portoferraio; locality: near Carabinieri Station; decimalLatitude: 42.813489; decimalLongitude: 10.316821; geodeticDatum: WGS84; coordinatePrecision: 0.0002; **Identification:** identifiedBy: G. Curletti; **Event:** eventDate: 2012-07-10; **Record Level:** collectionCode: LFPC
- **Type status:** Other material. **Occurrence:** recordedBy: Leonardo Forbicioni; individualCount: 5; lifeStage: adult; occurrenceID: 11390EA9-4121-5788-A266-26BBE207936F; **Taxon:** scientificName: Agrilusroscidus Kiesenwetter, 1857; order: Coleoptera; family: Buprestidae; genus: Agrilus; specificEpithet: roscidus; scientificNameAuthorship: Kiesenwetter, 1857; **Location:** islandGroup: Tuscan Archipelago; island: Isola d'Elba; country: Italy; countryCode: IT; stateProvince: Livorno; county: Porto Azzurro; locality: Buraccio; decimalLatitude: 42.780000; decimalLongitude: 10.354722; geodeticDatum: WGS84; coordinatePrecision: 0.0002; **Identification:** identifiedBy: L. Forbicioni; **Event:** eventDate: 2017-06-30; **Record Level:** collectionCode: LFPC
- **Type status:** Other material. **Occurrence:** recordedBy: Leonardo Forbicioni; individualCount: 1; lifeStage: adult; occurrenceID: 5B125C1F-5A30-5D2F-A357-8A3B0F3EE9AB; **Taxon:** scientificName: Agrilusroscidus Kiesenwetter, 1857; order: Coleoptera; family: Buprestidae; genus: Agrilus; specificEpithet: roscidus; scientificNameAuthorship: Kiesenwetter, 1857; **Location:** islandGroup: Tuscan Archipelago; island: Isola d'Elba; country: Italy; countryCode: IT; stateProvince: Livorno; county: Campo nell'Elba; locality: Monte Maolo; decimalLatitude: 42.773281; decimalLongitude: 10.187927; geodeticDatum: WGS84; coordinatePrecision: 0.0002; **Identification:** identifiedBy: G. Curletti; **Event:** eventDate: 2018-05-05; **Record Level:** collectionCode: LFPC
- **Type status:** Other material. **Occurrence:** recordedBy: Leonardo Forbicioni; individualCount: 1; lifeStage: adult; occurrenceID: DAB0EB81-D5A4-5168-B5E9-2020A7A9A40F; **Taxon:** scientificName: Agrilusroscidus Kiesenwetter, 1857; order: Coleoptera; family: Buprestidae; genus: Agrilus; specificEpithet: roscidus; scientificNameAuthorship: Kiesenwetter, 1857; **Location:** islandGroup: Tuscan Archipelago; island: Isola d'Elba; country: Italy; countryCode: IT; stateProvince: Livorno; county: Capoliveri; locality: Monte Calamita/Remaiolo; decimalLatitude: 42.724434; decimalLongitude: 10.419224; geodeticDatum: WGS84; coordinatePrecision: 0.0002; **Identification:** identifiedBy: G. Curletti; **Event:** eventDate: 2012-07-14; **Record Level:** collectionCode: LFPC
- **Type status:** Other material. **Occurrence:** recordedBy: Leonardo Forbicioni; individualCount: 2; lifeStage: adult; occurrenceID: DCA04728-9D17-5B42-8E54-E19F9E4AC83F; **Taxon:** scientificName: Agrilusroscidus Kiesenwetter, 1857; order: Coleoptera; family: Buprestidae; genus: Agrilus; specificEpithet: roscidus; scientificNameAuthorship: Kiesenwetter, 1857; **Location:** islandGroup: Tuscan Archipelago; island: Isola d'Elba; country: Italy; countryCode: IT; stateProvince: Livorno; county: Portoferraio; locality: Forte Inglese; decimalLatitude: 42.816505; decimalLongitude: 10.318427; geodeticDatum: WGS84; coordinatePrecision: 0.0002; **Identification:** identifiedBy: G. Curletti; **Event:** eventDate: 2022-07-21; **Record Level:** collectionCode: LFPC
- **Type status:** Other material. **Occurrence:** individualCount: 1; lifeStage: adult; occurrenceID: F0AFC1A4-F71C-59FD-83C8-CEEAF8F929D1; **Taxon:** scientificName: Agrilusroscidus Kiesenwetter, 1857; order: Coleoptera; family: Buprestidae; genus: Agrilus; specificEpithet: roscidus; scientificNameAuthorship: Kiesenwetter, 1857; **Location:** islandGroup: Tuscan Archipelago; island: Isola d'Elba; country: Italy; countryCode: IT; stateProvince: Livorno; **Identification:** identifiedBy: G. Curletti; **Record Level:** source: Curletti G. (1994) I Buprestidi d’Italia. Catalogo geonemico, sinonimico, bibliografico, biologico. Monografie di Natura Bresciana, Ed. Vannini, Brescia, 19.
- **Type status:** Other material. **Occurrence:** individualCount: 1; lifeStage: adult; occurrenceID: 32187FD9-364A-5B4C-9D9A-506042A1D2B2; **Taxon:** scientificName: Agrilusroscidus Kiesenwetter, 1857; order: Coleoptera; family: Buprestidae; genus: Agrilus; specificEpithet: roscidus; scientificNameAuthorship: Kiesenwetter, 1857; **Location:** islandGroup: Tuscan Archipelago; island: Isola del Giglio; country: Italy; countryCode: GR; stateProvince: Grosseto; **Identification:** identifiedBy: G. Curletti; **Record Level:** source: Curletti G. (1994) I Buprestidi d’Italia. Catalogo geonemico, sinonimico, bibliografico, biologico. Monografie di Natura Bresciana, Ed. Vannini, Brescia, 19.

##### Conservation status

LC

##### Distribution

Recorded for the Tuscan Archipelago (Isola d'Elba) by Curletti (1994).

#### Agrilus solieri solieri

Gory & Laporte, 1837

A1F801F8-B213-5385-AE7B-0568D79D17BC

##### Materials

- **Type status:** Other material. **Occurrence:** individualCount: 1; lifeStage: adult; occurrenceID: 4D2D42AF-E99B-5F5B-9597-ECD5558F4A20; **Taxon:** scientificName: Agrilussolierisolieri Gory & Laporte, 1837; order: Coleoptera; family: Buprestidae; genus: Agrilus; specificEpithet: solieri; infraspecificEpithet: solieri; scientificNameAuthorship: Gory & Laporte, 1837; **Location:** islandGroup: Tuscan Archipelago; island: Isola d'Elba; country: Italy; countryCode: IT; stateProvince: Livorno; county: Rio; municipality: Rio Marina; locality: Ortano; **Record Level:** source: Curletti G. (1994) I Buprestidi d’Italia. Catalogo geonemico, sinonimico, bibliografico, biologico. Monografie di Natura Bresciana, Ed. Vannini, Brescia, 19.

##### Distribution

Recorded for the Tuscan Archipelago (Isola d'Elba) by Curletti (1994).

#### Agrilus viridicaerulans rubi

Schaefer, 1937

9CCA2E1B-94E7-5D7E-A733-4D33F77D23C6

##### Materials

- **Type status:** Other material. **Occurrence:** recordedBy: Leonardo Forbicioni; individualCount: 1; lifeStage: adult; occurrenceID: 73CB3449-C6BC-58A5-A03E-81A67B56E642; **Taxon:** scientificName: Agrilusviridicaerulansrubi Schaefer, 1937; order: Coleoptera; family: Buprestidae; genus: Agrilus; specificEpithet: viridicaerulans; infraspecificEpithet: rubi; scientificNameAuthorship: Schaefer, 1937; **Location:** islandGroup: Tuscan Archipelago; island: Isola d'Elba; country: Italy; countryCode: IT; stateProvince: Livorno; county: Capoliveri; locality: Pian di Mola; decimalLatitude: 42.759336; decimalLongitude: 10.365624; geodeticDatum: WGS84; coordinatePrecision: 0.0002; **Identification:** identifiedBy: F. Terzani & E. Paggetti; **Event:** eventDate: 2011-06-26; **Record Level:** collectionCode: LFPC
- **Type status:** Other material. **Occurrence:** recordedBy: Leonardo Forbicioni; individualCount: 1; lifeStage: adult; occurrenceID: 9BEE6F59-D005-5763-8AD7-E193BF285EDE; **Taxon:** scientificName: Agrilusviridicaerulansrubi Schaefer, 1937; order: Coleoptera; family: Buprestidae; genus: Agrilus; specificEpithet: viridicaerulans; infraspecificEpithet: rubi; scientificNameAuthorship: Schaefer, 1937; **Location:** islandGroup: Tuscan Archipelago; island: Isola d'Elba; country: Italy; countryCode: IT; stateProvince: Livorno; county: Capoliveri; locality: Pian di Mola; decimalLatitude: 42.759361; decimalLongitude: 10.365511; geodeticDatum: WGS84; coordinatePrecision: 0.0002; **Identification:** identifiedBy: G. Curletti; **Event:** eventDate: 2013-06-06; **Record Level:** collectionCode: LFPC
- **Type status:** Other material. **Occurrence:** recordedBy: Leonardo Forbicioni; individualCount: 8; lifeStage: adult; occurrenceID: 5307BE2B-A4C5-5C9E-A537-E44CC3F7C8C5; **Taxon:** scientificName: Agrilusviridicaerulansrubi Schaefer, 1937; order: Coleoptera; family: Buprestidae; genus: Agrilus; specificEpithet: viridicaerulans; infraspecificEpithet: rubi; scientificNameAuthorship: Schaefer, 1937; **Location:** islandGroup: Tuscan Archipelago; island: Isola d'Elba; country: Italy; countryCode: IT; stateProvince: Livorno; county: Portoferraio; locality: Schiopparello/Le Prade; decimalLatitude: 42.795205; decimalLongitude: 10.349886; geodeticDatum: WGS84; coordinatePrecision: 0.0002; **Identification:** identifiedBy: L. Forbicioni; **Event:** eventDate: 2013-08-08; **Record Level:** collectionCode: LFPC
- **Type status:** Other material. **Occurrence:** recordedBy: Leonardo Forbicioni; individualCount: 1; lifeStage: adult; occurrenceID: 4845A5F9-2F0D-56AF-B749-E7ACD320BD5F; **Taxon:** scientificName: Agrilusviridicaerulansrubi Schaefer, 1937; order: Coleoptera; family: Buprestidae; genus: Agrilus; specificEpithet: viridicaerulans; infraspecificEpithet: rubi; scientificNameAuthorship: Schaefer, 1937; **Location:** islandGroup: Tuscan Archipelago; island: Isola d'Elba; country: Italy; countryCode: IT; stateProvince: Livorno; county: Capoliveri; locality: Mola-Spiaggia; decimalLatitude: 42.759556; decimalLongitude: 10.384912; geodeticDatum: WGS84; coordinatePrecision: 0.0002; **Identification:** identifiedBy: G. Curletti; **Event:** eventDate: 2021-06-27; **Record Level:** collectionCode: LFPC
- **Type status:** Other material. **Occurrence:** individualCount: 1; lifeStage: adult; occurrenceID: 7A00823D-2590-5CB6-BF55-6979DB6832A5; **Taxon:** scientificName: Agrilusviridicaerulansrubi Schaefer, 1937; order: Coleoptera; family: Buprestidae; genus: Agrilus; specificEpithet: viridicaerulans; infraspecificEpithet: rubi; scientificNameAuthorship: Schaefer, 1937; **Location:** islandGroup: Tuscan Archipelago; island: Isola d'Elba; country: Italy; countryCode: IT; stateProvince: Livorno; county: Porto Azzurro; **Identification:** identifiedBy: G. Curletti; **Record Level:** source: Curletti G. (1994) I Buprestidi d’Italia. Catalogo geonemico, sinonimico, bibliografico, biologico. Monografie di Natura Bresciana, Ed. Vannini, Brescia, 19.
- **Type status:** Other material. **Occurrence:** individualCount: 1; lifeStage: adult; occurrenceID: 709B3F06-9DA8-565A-86A9-BDE61A433858; **Taxon:** scientificName: Agrilusviridicaerulansrubi Schaefer, 1937; order: Coleoptera; family: Buprestidae; genus: Agrilus; specificEpithet: viridicaerulans; infraspecificEpithet: rubi; scientificNameAuthorship: Schaefer, 1937; **Location:** islandGroup: Tuscan Archipelago; island: Isola d'Elba; country: Italy; countryCode: IT; stateProvince: Livorno; county: Rio; municipality: Rio Marina; locality: Ortano; **Identification:** identifiedBy: G. Curletti; **Record Level:** source: Curletti G. (1994) I Buprestidi d’Italia. Catalogo geonemico, sinonimico, bibliografico, biologico. Monografie di Natura Bresciana, Ed. Vannini, Brescia, 19

##### Distribution

Recorded for the Tuscan Archipelago (Isola d'Elba) by Curletti (1994).

#### Aphanisticini

058C2FE4-F970-5450-A7AA-18F06097BC50

#### Aphanisticus elongatus elongatus

(Villa & Villa, 1835)

335DA5A0-4C36-5648-94C9-919BA6395D60

##### Materials

- **Type status:** Other material. **Occurrence:** recordedBy: Leonardo Forbicioni; individualCount: 2; lifeStage: adult; occurrenceID: 242C844C-888A-5E1F-9395-807694ACD6F7; **Taxon:** scientificName: Aphanisticus (Aphanisticus) elongatus elongatus (Villa & Villa, 1835); order: Coleoptera; family: Buprestidae; genus: Aphanisticus; specificEpithet: elongatus; infraspecificEpithet: elongatus; scientificNameAuthorship: (Villa & Villa, 1835); **Location:** islandGroup: Tuscan Archipelago; island: Isola d'Elba; country: Italy; countryCode: IT; stateProvince: Livorno; county: Capoliveri; locality: Pian di Mola; decimalLatitude: 42.759541; decimalLongitude: 10.364682; geodeticDatum: WGS84; coordinatePrecision: 0.0002; **Identification:** identifiedBy: E. Paggetti; **Event:** eventDate: 2011-06-02; **Record Level:** collectionCode: LFPC
- **Type status:** Other material. **Occurrence:** recordedBy: Leonardo Forbicioni; individualCount: 1; lifeStage: adult; occurrenceID: F6B97297-5C2B-5ACD-8C49-F2B6128D8CB1; **Taxon:** scientificName: Aphanisticus (Aphanisticus) elongatus elongatus (Villa & Villa, 1835); order: Coleoptera; family: Buprestidae; genus: Aphanisticus; specificEpithet: elongatus; infraspecificEpithet: elongatus; scientificNameAuthorship: (Villa & Villa, 1835); **Location:** islandGroup: Tuscan Archipelago; island: Isola d'Elba; country: Italy; countryCode: IT; stateProvince: Livorno; county: Capoliveri; locality: Pian di Mola; decimalLatitude: 42.759503; decimalLongitude: 10.365007; geodeticDatum: WGS84; coordinatePrecision: 0.0002; **Identification:** identifiedBy: L. Forbicioni; **Event:** eventDate: 2013-04-03; **Record Level:** collectionCode: LFPC
- **Type status:** Other material. **Occurrence:** recordedBy: Leonardo Forbicioni; individualCount: 1; lifeStage: adult; occurrenceID: 912609B3-FCCD-56AF-96E8-859774149370; **Taxon:** scientificName: Aphanisticus (Aphanisticus) elongatus elongatus (Villa & Villa, 1835); order: Coleoptera; family: Buprestidae; genus: Aphanisticus; specificEpithet: elongatus; infraspecificEpithet: elongatus; scientificNameAuthorship: (Villa & Villa, 1835); **Location:** islandGroup: Tuscan Archipelago; island: Isola d'Elba; country: Italy; countryCode: IT; stateProvince: Livorno; county: Portoferraio; locality: Vecchio Papa; decimalLatitude: 42.784872; decimalLongitude: 10.338283; geodeticDatum: WGS84; coordinatePrecision: 0.0002; **Identification:** identifiedBy: E. Paggetti; **Event:** eventDate: 2012-03-21; **Record Level:** collectionCode: LFPC
- **Type status:** Other material. **Occurrence:** recordedBy: Leonardo Forbicioni; individualCount: 1; lifeStage: adult; occurrenceID: 0FABF1DB-6BB4-5B61-9398-2BC13EADAB0D; **Taxon:** scientificName: Aphanisticus (Aphanisticus) elongatus elongatus (Villa & Villa, 1835); order: Coleoptera; family: Buprestidae; genus: Aphanisticus; specificEpithet: elongatus; infraspecificEpithet: elongatus; scientificNameAuthorship: (Villa & Villa, 1835); **Location:** islandGroup: Tuscan Archipelago; island: Isola d'Elba; country: Italy; countryCode: IT; stateProvince: Livorno; county: Capoliveri; locality: Pian di Mola; decimalLatitude: 42.759262; decimalLongitude: 10.367065; geodeticDatum: WGS84; coordinatePrecision: 0.0002; **Identification:** identifiedBy: E. Paggetti; **Event:** eventDate: 2011-06-28; **Record Level:** collectionCode: LFPC
- **Type status:** Other material. **Occurrence:** recordedBy: Leonardo Forbicioni; individualCount: 1; lifeStage: adult; occurrenceID: 59994DF2-2ECA-5ECC-B25F-10F690A6E507; **Taxon:** scientificName: Aphanisticus (Aphanisticus) elongatus elongatus (Villa & Villa, 1835); order: Coleoptera; family: Buprestidae; genus: Aphanisticus; specificEpithet: elongatus; infraspecificEpithet: elongatus; scientificNameAuthorship: (Villa & Villa, 1835); **Location:** islandGroup: Tuscan Archipelago; island: Isola d'Elba; country: Italy; countryCode: IT; stateProvince: Livorno; county: Campo nell'Elba; locality: Monte Perone/Castagnone; decimalLatitude: 42.765943; decimalLongitude: 10.186093; geodeticDatum: WGS84; coordinatePrecision: 0.0002; **Identification:** identifiedBy: L. Forbicioni; **Event:** eventDate: 2013-06-19; **Record Level:** collectionCode: LFPC

#### Aphanisticus pygmaeus

Lucas, 1846

66FA860D-8C74-5DA4-B22B-4EA39D95CC57

##### Materials

- **Type status:** Other material. **Occurrence:** recordedBy: Leonardo Forbicioni; individualCount: 1; lifeStage: adult; occurrenceID: ED568A1E-0876-5AF0-84AF-DDC19B534D7E; **Taxon:** scientificName: Aphanisticus (Aphanisticus) pygmaeus Lucas, 1846; order: Coleoptera; family: Buprestidae; genus: Aphanisticus; specificEpithet: pygmaeus; scientificNameAuthorship: (Villa & Villa, 1835); **Location:** islandGroup: Tuscan Archipelago; island: Isola d'Elba; country: Italy; countryCode: IT; stateProvince: Livorno; county: Portoferraio; locality: Vecchio Papa; decimalLatitude: 42.785390; decimalLongitude: 10.339065; geodeticDatum: WGS84; coordinatePrecision: 0.0002; **Identification:** identifiedBy: E. Paggetti; **Event:** eventDate: 2011-06-28; **Record Level:** collectionCode: LFPC
- **Type status:** Other material. **Occurrence:** recordedBy: Leonardo Forbicioni; individualCount: 1; lifeStage: adult; occurrenceID: 89B792C9-5945-58C1-9C74-733A3D0E9D4F; **Taxon:** scientificName: Aphanisticus (Aphanisticus) pygmaeus Lucas, 1846; order: Coleoptera; family: Buprestidae; genus: Aphanisticus; specificEpithet: pygmaeus; scientificNameAuthorship: Lucas, 1846; **Location:** islandGroup: Tuscan Archipelago; island: Isola d'Elba; country: Italy; countryCode: IT; stateProvince: Livorno; county: Portoferraio; locality: San Govanni; decimalLatitude: 42.801761; decimalLongitude: 10.316416; geodeticDatum: WGS84; coordinatePrecision: 0.0002; **Identification:** identifiedBy: E. Paggetti; **Event:** eventDate: 2010-09-01; **Record Level:** collectionCode: LFPC
- **Type status:** Other material. **Occurrence:** individualCount: 1; lifeStage: adult; occurrenceID: 3EA48EE2-652F-5B37-91BC-B348936779F5; **Taxon:** scientificName: Aphanisticus (Aphanisticus) pygmaeus Lucas, 1846; order: Coleoptera; family: Buprestidae; genus: Aphanisticus; specificEpithet: pygmaeus; scientificNameAuthorship: Lucas, 1846; **Location:** islandGroup: Tuscan Archipelago; island: Isola d'Elba; country: Italy; countryCode: IT; stateProvince: Livorno; **Record Level:** source: Holdhaus K. (1923) Elenco dei Coleotteri dell’isola d’Elba con studi sul problema della Tirrenide. Memorie della Società entomologica italiana 2: 77‑175. - Curletti G. (1994) I Buprestidi d’Italia. Catalogo geonemico, sinonimico, bibliografico, biologico. Monografie di Natura Bresciana, Ed. Vannini, Brescia, 19
- **Type status:** Other material. **Occurrence:** individualCount: 1; lifeStage: adult; occurrenceID: 2C8F1F42-88BF-5D30-A75A-0900A680A567; **Taxon:** scientificName: Aphanisticus (Aphanisticus) pygmaeus Lucas, 1846; order: Coleoptera; family: Buprestidae; genus: Aphanisticus; specificEpithet: pygmaeus; scientificNameAuthorship: Lucas, 1846; **Location:** islandGroup: Tuscan Archipelago; island: Isola d'Elba; country: Italy; countryCode: IT; stateProvince: Livorno; **Record Level:** source: Porta, 1929 - Fauna Coleopterorum Italica Vol III Diversicornia (p.410)

##### Distribution

Recorded for the Tuscan Archipelago (Isola d'Elba) by Holdhaus 1923 and Curletti 1994.

#### Coraebini

5B6F1419-2204-5292-B359-1088409C90DC

#### Coraebus elatus elatus

(Fabricius, 1787)

3B82F828-D865-5123-B730-571A7DF0E9F6

##### Materials

- **Type status:** Other material. **Occurrence:** recordedBy: Leonardo Forbicioni; individualCount: 1; lifeStage: adult; occurrenceID: 90735EFF-162F-507E-98E8-9EBED2492EDC; **Taxon:** scientificName: Coraebuselatuselatus (Fabricius, 1787); order: Coleoptera; family: Buprestidae; genus: Coraebus; specificEpithet: elatus; infraspecificEpithet: elatus; scientificNameAuthorship: (Fabricius, 1787); **Location:** islandGroup: Tuscan Archipelago; island: Isola d'Elba; country: Italy; countryCode: IT; stateProvince: Livorno; county: Portoferraio; locality: Acquabona; decimalLatitude: 42.786867; decimalLongitude: 10.347132; geodeticDatum: WGS84; coordinatePrecision: 0.0002; **Identification:** identifiedBy: E. Paggetti; **Event:** eventDate: 2011-05-26; **Record Level:** collectionCode: LFPC
- **Type status:** Other material. **Occurrence:** recordedBy: Leonardo Forbicioni; individualCount: 5; lifeStage: adult; occurrenceID: 6DDFA1ED-3806-54FE-9690-AB5189540D15; **Taxon:** scientificName: Coraebuselatuselatus (Fabricius, 1787); order: Coleoptera; family: Buprestidae; genus: Coraebus; specificEpithet: elatus; infraspecificEpithet: elatus; scientificNameAuthorship: (Fabricius, 1787); **Location:** islandGroup: Tuscan Archipelago; island: Isola d'Elba; country: Italy; countryCode: IT; stateProvince: Livorno; county: Capoliveri; locality: Pian di Mola; decimalLatitude: 42.759041; decimalLongitude: 10.368619; geodeticDatum: WGS84; coordinatePrecision: 0.0002; **Identification:** identifiedBy: E. Paggetti; **Event:** eventDate: 2011-06-02; **Record Level:** collectionCode: LFPC
- **Type status:** Other material. **Occurrence:** recordedBy: Leonardo Forbicioni; individualCount: 1; lifeStage: adult; occurrenceID: 82A619EB-A98F-5C3C-BDA0-4C40BD499E91; **Taxon:** scientificName: Coraebuselatuselatus (Fabricius, 1787); order: Coleoptera; family: Buprestidae; genus: Coraebus; specificEpithet: elatus; infraspecificEpithet: elatus; scientificNameAuthorship: (Fabricius, 1787); **Location:** islandGroup: Tuscan Archipelago; island: Isola d'Elba; country: Italy; countryCode: IT; stateProvince: Livorno; county: Porto Azzurro; locality: Buraccio/Lo Stipito; decimalLatitude: 42.762166; decimalLongitude: 10.365712; geodeticDatum: WGS84; coordinatePrecision: 0.0002; **Identification:** identifiedBy: E. Paggetti; **Event:** eventDate: 2011-06-30; **Record Level:** collectionCode: LFPC
- **Type status:** Other material. **Occurrence:** individualCount: 1; lifeStage: adult; occurrenceID: 9A951C1A-DF07-5C67-B56A-C8C00CAFA690; **Taxon:** scientificName: Coraebuselatuselatus (Fabricius, 1787); order: Coleoptera; family: Buprestidae; genus: Coraebus; specificEpithet: elatus; infraspecificEpithet: elatus; scientificNameAuthorship: (Fabricius, 1787); **Location:** islandGroup: Tuscan Archipelago; island: Isola d'Elba; country: Italy; countryCode: IT; stateProvince: Livorno; **Record Level:** source: Porta, 1929 - Fauna Coleopterorum Italica, Vol III Diversicornia (p. 400 sub Coraebus sinuatus Creutzer, 1796)
- **Type status:** Other material. **Occurrence:** individualCount: 1; lifeStage: adult; occurrenceID: 8803BD1D-4DB7-5236-B7AF-383DC2853273; **Taxon:** scientificName: Coraebuselatuselatus (Fabricius, 1787); order: Coleoptera; family: Buprestidae; genus: Coraebus; specificEpithet: elatus; infraspecificEpithet: elatus; scientificNameAuthorship: (Fabricius, 1787); **Location:** islandGroup: Tuscan Archipelago; island: Isola d'Elba; country: Italy; countryCode: IT; stateProvince: Livorno; **Identification:** identifiedBy: G. Curletti; **Record Level:** source: Holdhaus K. (1923) Elenco dei Coleotteri dell’isola d’Elba con studi sul problema della Tirrenide. Memorie della Società entomologica italiana 2: 77‑175. Sub Coraebus lampsanae Bon.
- **Type status:** Other material. **Occurrence:** individualCount: 1; lifeStage: adult; occurrenceID: 5A32E2C0-B1D0-54D7-9E91-81A763D4CBD6; **Taxon:** scientificName: Coraebuselatuselatus (Fabricius, 1787); order: Coleoptera; family: Buprestidae; genus: Coraebus; specificEpithet: elatus; infraspecificEpithet: elatus; scientificNameAuthorship: (Fabricius, 1787); **Location:** islandGroup: Tuscan Archipelago; island: Isola d'Elba; country: Italy; countryCode: IT; stateProvince: Livorno; **Identification:** identifiedBy: G. Curletti; **Record Level:** source: Curletti G. (1994) I Buprestidi d’Italia. Catalogo geonemico, sinonimico, bibliografico, biologico. Monografie di Natura Bresciana, Ed. Vannini, Brescia, 19.

##### Distribution

Recorded for the Tuscan Archipelago (Isola d'Elba) by Holdhaus 1923 and Curletti 1994.

#### Coraebus fasciatus

(Villers, 1789)

AA5A4AA3-A7AD-5CAB-BB1A-507002AB1069

##### Materials

- **Type status:** Other material. **Occurrence:** recordedBy: Leonardo Forbicioni; individualCount: 1; lifeStage: adult; occurrenceID: 70D463B9-B77C-59C3-8FE2-58310CF38913; **Taxon:** scientificName: Coraebusfasciatus (Villers, 1789); order: Coleoptera; family: Buprestidae; genus: Coraebus; specificEpithet: fasciatus; scientificNameAuthorship: (Villers, 1789); **Location:** islandGroup: Tuscan Archipelago; island: Isola d'Elba; country: Italy; countryCode: IT; stateProvince: Livorno; county: Portoferraio; locality: Acquabona; decimalLatitude: 42.782714; decimalLongitude: 10.337327; geodeticDatum: WGS84; coordinatePrecision: 0.0002; **Identification:** identifiedBy: L. Forbicioni; **Event:** eventDate: 2008-09-07; **Record Level:** collectionCode: LFPC
- **Type status:** Other material. **Occurrence:** recordedBy: Leonardo Forbicioni; individualCount: 1; lifeStage: adult; occurrenceID: EFB990A3-2844-537A-A643-65F60CA02DFE; **Taxon:** scientificName: Coraebusfasciatus (Villers, 1789); order: Coleoptera; family: Buprestidae; genus: Coraebus; specificEpithet: fasciatus; scientificNameAuthorship: (Villers, 1789); **Location:** islandGroup: Tuscan Archipelago; island: Isola d'Elba; country: Italy; countryCode: IT; stateProvince: Livorno; county: Capoliveri; locality: Monte Calamita; decimalLatitude: 42.727977; decimalLongitude: 10.419073; geodeticDatum: WGS84; coordinatePrecision: 0.0002; **Identification:** identifiedBy: L. Forbicioni; **Event:** eventDate: 2016-06-20; **Record Level:** collectionCode: LFPC
- **Type status:** Other material. **Occurrence:** recordedBy: Leonardo Forbicioni; individualCount: 2; lifeStage: adult; occurrenceID: 89C93508-E6C7-53D8-A9D6-4DE976A7C8AD; **Taxon:** scientificName: Coraebusfasciatus (Villers, 1789); order: Coleoptera; family: Buprestidae; genus: Coraebus; specificEpithet: fasciatus; scientificNameAuthorship: (Villers, 1789); **Location:** islandGroup: Tuscan Archipelago; island: Isola d'Elba; country: Italy; countryCode: IT; stateProvince: Livorno; county: Rio; municipality: Rio nell'Elba; locality: Volterraio; decimalLatitude: 42.809756; decimalLongitude: 10.393489; geodeticDatum: WGS84; coordinatePrecision: 0.0002; **Identification:** identifiedBy: L. Forbicioni; **Event:** eventDate: 2019-07-08; **Record Level:** collectionCode: LFPC
- **Type status:** Other material. **Occurrence:** individualCount: 1; lifeStage: adult; occurrenceID: 2F6A06F4-A33E-521E-9960-6CB3457F6215; **Taxon:** scientificName: Coraebusfasciatus (Villers, 1789); order: Coleoptera; family: Buprestidae; genus: Coraebus; specificEpithet: fasciatus; scientificNameAuthorship: (Villers, 1789); **Location:** islandGroup: Tuscan Archipelago; island: Isola d'Elba; country: Italy; countryCode: IT; stateProvince: Livorno; county: Portoferraio; locality: Enfola; decimalLatitude: 42.824887; decimalLongitude: 10.265280; geodeticDatum: WGS84; **Identification:** identifiedBy: L. Forbicioni; **Event:** eventDate: 2016-08-11; **Record Level:** source: https://www.inaturalist.org/observations/3858057

##### Conservation status

LC

#### Coraebus rubi

(Linnaeus, 1767)

F54E4CD4-6FDF-560C-ADA1-79D1B40B3419

##### Materials

- **Type status:** Other material. **Occurrence:** recordedBy: Leonardo Forbicioni; individualCount: 2; lifeStage: adult; occurrenceID: 047E292B-BAF9-5278-A5B8-A9ABFA1BD8C3; **Taxon:** scientificName: Coraebusrubi (Linnaeus, 1767); order: Coleoptera; family: Buprestidae; genus: Coraebus; specificEpithet: rubi; scientificNameAuthorship: (Linnaeus, 1767); **Location:** islandGroup: Tuscan Archipelago; island: Isola d'Elba; country: Italy; countryCode: IT; stateProvince: Livorno; county: Campo nell'Elba; municipality: San Piero; decimalLatitude: 42.761243; decimalLongitude: 10.208388; geodeticDatum: WGS84; coordinatePrecision: 0.0002; **Identification:** identifiedBy: E. Paggetti; **Event:** eventDate: 2012-06-10; **Record Level:** collectionCode: LFPC
- **Type status:** Other material. **Occurrence:** recordedBy: Leonardo Forbicioni; individualCount: 1; lifeStage: adult; occurrenceID: AADE3FBB-CD7C-5833-9276-A3729ACF89F6; **Taxon:** scientificName: Coraebusrubi (Linnaeus, 1767); order: Coleoptera; family: Buprestidae; genus: Coraebus; specificEpithet: rubi; scientificNameAuthorship: (Linnaeus, 1767); **Location:** islandGroup: Tuscan Archipelago; island: Isola d'Elba; country: Italy; countryCode: IT; stateProvince: Livorno; county: Capoliveri; locality: Pian di Mola; decimalLatitude: 42.759183; decimalLongitude: 10.365978; geodeticDatum: WGS84; coordinatePrecision: 0.0002; **Identification:** identifiedBy: E. Paggetti; **Event:** eventDate: 2011-06-09; **Record Level:** collectionCode: LFPC
- **Type status:** Other material. **Occurrence:** recordedBy: Leonardo Forbicioni; individualCount: 5; lifeStage: adult; occurrenceID: 4BCC9F44-ED22-5641-85A4-29FEF25008AD; **Taxon:** scientificName: Coraebusrubi (Linnaeus, 1767); order: Coleoptera; family: Buprestidae; genus: Coraebus; specificEpithet: rubi; scientificNameAuthorship: (Linnaeus, 1767); **Location:** islandGroup: Tuscan Archipelago; island: Isola d'Elba; country: Italy; countryCode: IT; stateProvince: Livorno; county: Capoliveri; locality: Pian di Mola; decimalLatitude: 42.758563; decimalLongitude: 10.366305; geodeticDatum: WGS84; coordinatePrecision: 0.0002; **Identification:** identifiedBy: E. Paggetti; **Event:** eventDate: 2011-06-26; **Record Level:** collectionCode: LFPC
- **Type status:** Other material. **Occurrence:** recordedBy: Leonardo Forbicioni; individualCount: 1; lifeStage: adult; occurrenceID: EE79F4AE-98E3-5EEE-AFCD-CF24022791AF; **Taxon:** scientificName: Coraebusrubi (Linnaeus, 1767); order: Coleoptera; family: Buprestidae; genus: Coraebus; specificEpithet: rubi; scientificNameAuthorship: (Linnaeus, 1767); **Location:** islandGroup: Tuscan Archipelago; island: Isola d'Elba; country: Italy; countryCode: IT; stateProvince: Livorno; county: Portoferraio; locality: Volterraio; decimalLatitude: 42.800405; decimalLongitude: 10.380389; geodeticDatum: WGS84; coordinatePrecision: 0.0002; **Identification:** identifiedBy: E. Paggetti; **Event:** eventDate: 2011-06-15; **Record Level:** collectionCode: LFPC
- **Type status:** Other material. **Occurrence:** recordedBy: Leonardo Forbicioni; individualCount: 1; lifeStage: adult; occurrenceID: 9A169DD9-85BE-5847-A708-02494F19C3EA; **Taxon:** scientificName: Coraebusrubi (Linnaeus, 1767); order: Coleoptera; family: Buprestidae; genus: Coraebus; specificEpithet: rubi; scientificNameAuthorship: (Linnaeus, 1767); **Location:** islandGroup: Tuscan Archipelago; island: Isola d'Elba; country: Italy; countryCode: IT; stateProvince: Livorno; county: Porto Azzurro; locality: Buraccio; decimalLatitude: 42.779661; decimalLongitude: 10.356152; geodeticDatum: WGS84; coordinatePrecision: 0.0002; **Identification:** identifiedBy: L. Forbicioni; **Event:** eventDate: 2022-06-13; **Record Level:** collectionCode: LFPC
- **Type status:** Other material. **Occurrence:** recordedBy: Leonardo Forbicioni; individualCount: 1; lifeStage: adult; occurrenceID: 890B6400-BBDF-5B8E-9219-0360023A9F41; **Taxon:** scientificName: Coraebusrubi (Linnaeus, 1767); order: Coleoptera; family: Buprestidae; genus: Coraebus; specificEpithet: rubi; scientificNameAuthorship: (Linnaeus, 1767); **Location:** islandGroup: Tuscan Archipelago; island: Isola d'Elba; country: Italy; countryCode: IT; stateProvince: Livorno; county: Porto Azzurro; locality: Buraccio; decimalLatitude: 42.780244; decimalLongitude: 10.355640; geodeticDatum: WGS84; coordinatePrecision: 0.0002; **Identification:** identifiedBy: L. Forbicioni; **Event:** eventDate: 2020-06-17; **Record Level:** collectionCode: LFPC
- **Type status:** Other material. **Occurrence:** recordedBy: Andrea Beltramini; individualCount: 1; lifeStage: adult; occurrenceID: 7D97589E-AC55-5456-854A-ADD2FD9370E4; **Taxon:** scientificName: Coraebusrubi (Linnaeus, 1767); order: Coleoptera; family: Buprestidae; genus: Coraebus; specificEpithet: rubi; scientificNameAuthorship: (Linnaeus, 1767); **Location:** islandGroup: Tuscan Archipelago; island: Isola d'Elba; country: Italy; countryCode: IT; stateProvince: Livorno; county: Portoferraio; locality: Volterraio; decimalLatitude: 42.799103; decimalLongitude: 10.380212; geodeticDatum: WGS84; coordinatePrecision: 0.0002; **Identification:** identifiedBy: L. Forbicioni; **Event:** eventDate: 12-26/06/2023; **Record Level:** collectionCode: ABPC
- **Type status:** Other material. **Occurrence:** recordedBy: Leonardo Forbicioni; individualCount: 1; lifeStage: adult; occurrenceID: A5FD9531-EB6A-50CA-A609-04F7225D9184; **Taxon:** scientificName: Coraebusrubi (Linnaeus, 1767); order: Coleoptera; family: Buprestidae; genus: Coraebus; specificEpithet: rubi; scientificNameAuthorship: (Linnaeus, 1767); **Location:** islandGroup: Tuscan Archipelago; island: Isola d'Elba; country: Italy; countryCode: IT; stateProvince: Livorno; county: Porto Azzurro; locality: Buraccio; decimalLatitude: 42.780400; decimalLongitude: 10.351687; geodeticDatum: WGS84; coordinatePrecision: 4.0E-5; **Identification:** identifiedBy: L. Forbicioni; **Event:** eventDate: 2022-06-12; **Record Level:** source: https://www.inaturalist.org/observations/121404641
- **Type status:** Other material. **Occurrence:** individualCount: 1; lifeStage: adult; occurrenceID: 534F118A-68FE-5566-BE48-135AFC9401E2; **Taxon:** scientificName: Coraebusrubi (Linnaeus, 1767); order: Coleoptera; family: Buprestidae; genus: Coraebus; specificEpithet: rubi; scientificNameAuthorship: (Linnaeus, 1767); **Location:** islandGroup: Tuscan Archipelago; island: Isola d'Elba; country: Italy; countryCode: IT; stateProvince: Livorno; county: Marciana; **Record Level:** source: Razzauti A (1921) Contributi alla conoscenza faunistica delle isole toscane III. Coleotteri delle Isole d’Elba, di Capraia e di Gorgona Atti della Società toscana di Scienze naturali residente in Pisa. 33. Memorie, Pisa, 96-122 pp.. https://www.biodiversitylibrary.org/page/35335669
- **Type status:** Other material. **Occurrence:** individualCount: 1; lifeStage: adult; occurrenceID: E49FC7B0-E3C8-5530-BAB5-912B533F9537; **Taxon:** scientificName: Coraebusrubi (Linnaeus, 1767); order: Coleoptera; family: Buprestidae; genus: Coraebus; specificEpithet: rubi; scientificNameAuthorship: (Linnaeus, 1767); **Location:** islandGroup: Tuscan Archipelago; island: Isola d'Elba; country: Italy; countryCode: IT; stateProvince: Livorno; **Record Level:** source: Holdhaus K. (1923) Elenco dei Coleotteri dell’isola d’Elba con studi sul problema della Tirrenide. Memorie della Società entomologica italiana 2: 77‑175. - Curletti G. (1994) I Buprestidi d’Italia. Catalogo geonemico, sinonimico, bibliografico, biologico. Monografie di Natura Bresciana, Ed. Vannini, Brescia, 19.

##### Distribution

Recorded for the Tuscan Archipelago (Isola d'Elba) by Holdhaus 1923 and Curletti 1994.

#### Meliboeus (Meliboeoides) parvulus

(Küster, 1852)

ABD9B04F-507C-55CA-9BB6-8881575ED049

##### Materials

- **Type status:** Other material. **Occurrence:** recordedBy: Leonardo Forbicioni; individualCount: 2; lifeStage: adult; occurrenceID: 3B41344A-A9A0-53EA-A6E3-C8E69E83ECED; **Taxon:** scientificName: Meliboeus (Meliboeoides) parvulus (Küster, 1852); order: Coleoptera; family: Buprestidae; genus: Meliboeus; subgenus: Meliboeoides; specificEpithet: parvulus; scientificNameAuthorship: (Küster, 1852); **Location:** islandGroup: Tuscan Archipelago; island: Isola d'Elba; country: Italy; countryCode: IT; stateProvince: Livorno; county: Portoferraio; locality: Volterraio; decimalLatitude: 42.800405; decimalLongitude: 10.380389; geodeticDatum: WGS84; coordinatePrecision: 0.0002; **Identification:** identifiedBy: E. Paggetti; **Event:** eventDate: 2011-06-15; **Record Level:** collectionCode: LFPC
- **Type status:** Other material. **Occurrence:** recordedBy: Leonardo Forbicioni; individualCount: 1; lifeStage: adult; occurrenceID: 3E42955E-9DBC-5577-8240-102EC0D60877; **Taxon:** scientificName: Meliboeus (Meliboeoides) parvulus (Küster, 1852); order: Coleoptera; family: Buprestidae; genus: Meliboeus; subgenus: Meliboeoides; specificEpithet: parvulus; scientificNameAuthorship: (Küster, 1852); **Location:** islandGroup: Tuscan Archipelago; island: Isola d'Elba; country: Italy; countryCode: IT; stateProvince: Livorno; county: Capoliveri; locality: Monte Calamita/Le Cavallacce; decimalLatitude: 42.737005; decimalLongitude: 10.386269; geodeticDatum: WGS84; coordinatePrecision: 0.0002; **Identification:** identifiedBy: E. Paggetti; **Event:** eventDate: 2014-07-13; **Record Level:** collectionCode: LFPC
- **Type status:** Other material. **Occurrence:** recordedBy: Leonardo Forbicioni; individualCount: 4; lifeStage: adult; occurrenceID: 22DB1CE7-7290-5826-97AB-24595D80908C; **Taxon:** scientificName: Meliboeus (Meliboeoides) parvulus (Küster, 1852); order: Coleoptera; family: Buprestidae; genus: Meliboeus; subgenus: Meliboeoides; specificEpithet: parvulus; scientificNameAuthorship: (Küster, 1852); **Location:** islandGroup: Tuscan Archipelago; island: Isola d'Elba; country: Italy; countryCode: IT; stateProvince: Livorno; county: Campo nell'Elba; locality: Monte Maolo; decimalLatitude: 42.774734; decimalLongitude: 10.190442; geodeticDatum: WGS84; coordinatePrecision: 0.0002; **Identification:** identifiedBy: E. Paggetti; **Event:** eventDate: 2019-06-02; **Record Level:** collectionCode: LFPC

#### Meliboeus (Meliboeus) fulgidicollis

(Lucas, 1846)

C442CBB9-6B63-5A61-99D3-ADB43F4BC2E5

##### Materials

- **Type status:** Other material. **Occurrence:** recordedBy: Leonardo Forbicioni; individualCount: 2; lifeStage: adult; occurrenceID: F7F7861A-A548-5DD7-A2A4-7DEA45AF26CC; **Taxon:** scientificName: Meliboeus (Meliboeus) fulgidicollis (Lucas, 1846); order: Coleoptera; family: Buprestidae; genus: Meliboeus; subgenus: Meliboeus; specificEpithet: fulgidicollis; scientificNameAuthorship: (Lucas, 1846); **Location:** islandGroup: Tuscan Archipelago; island: Isola d'Elba; country: Italy; countryCode: IT; stateProvince: Livorno; county: Campo nell'Elba; municipality: San Piero; decimalLatitude: 42.761211; decimalLongitude: 10.206770; geodeticDatum: WGS84; coordinatePrecision: 0.0002; **Identification:** identifiedBy: E. Paggetti; **Event:** eventDate: 2012-06-10; **Record Level:** collectionCode: LFPC
- **Type status:** Other material. **Occurrence:** recordedBy: Leonardo Forbicioni; individualCount: 2; lifeStage: adult; occurrenceID: C2B49FB7-05BD-5407-841C-87C107342ADA; **Taxon:** scientificName: Meliboeus (Meliboeus) fulgidicollis (Lucas, 1846); order: Coleoptera; family: Buprestidae; genus: Meliboeus; subgenus: Meliboeus; specificEpithet: fulgidicollis; scientificNameAuthorship: (Lucas, 1846); **Location:** islandGroup: Tuscan Archipelago; island: Isola d'Elba; country: Italy; countryCode: IT; stateProvince: Livorno; county: Capoliveri; locality: Il Gualdo; decimalLatitude: 42.753807; decimalLongitude: 10.392424; geodeticDatum: WGS84; coordinatePrecision: 0.0002; **Identification:** identifiedBy: L. Forbicioni; **Event:** eventDate: 2014-06-08; **Record Level:** collectionCode: LFPC
- **Type status:** Other material. **Occurrence:** recordedBy: Leonardo Forbicioni; individualCount: 1; lifeStage: adult; occurrenceID: 2C7ABC2C-ED20-5A83-8938-4B2BFF1A2AAC; **Taxon:** scientificName: Meliboeus (Meliboeus) fulgidicollis (Lucas, 1846); order: Coleoptera; family: Buprestidae; genus: Meliboeus; subgenus: Meliboeus; specificEpithet: fulgidicollis; scientificNameAuthorship: (Lucas, 1846); **Location:** islandGroup: Tuscan Archipelago; island: Isola d'Elba; country: Italy; countryCode: IT; stateProvince: Livorno; county: Campo nell'Elba; locality: Monte Perone/Masso alla Quata; decimalLatitude: 42.761703; decimalLongitude: 10.185286; geodeticDatum: WGS84; coordinatePrecision: 0.0002; **Identification:** identifiedBy: L. Forbicioni; **Event:** eventDate: 2020-06-21; **Record Level:** collectionCode: LFPC
- **Type status:** Other material. **Occurrence:** recordedBy: Leonardo Forbicioni; individualCount: 1; lifeStage: adult; occurrenceID: 11E29A38-1EFE-5BB2-B4C3-2CE2C3A79BA0; **Taxon:** scientificName: Meliboeus (Meliboeus) fulgidicollis (Lucas, 1846); order: Coleoptera; family: Buprestidae; genus: Meliboeus; subgenus: Meliboeus; specificEpithet: fulgidicollis; scientificNameAuthorship: (Lucas, 1846); **Location:** islandGroup: Tuscan Archipelago; island: Isola d'Elba; country: Italy; countryCode: IT; stateProvince: Livorno; county: Campo nell'Elba; municipality: Sant'Ilario; decimalLatitude: 42.763288; decimalLongitude: 10.224077; geodeticDatum: WGS84; coordinatePrecision: 0.0002; **Identification:** identifiedBy: L. Forbicioni; **Event:** eventDate: 2014-06-22; **Record Level:** collectionCode: LFPC
- **Type status:** Other material. **Occurrence:** recordedBy: Leonardo Forbicioni; individualCount: 1; lifeStage: adult; occurrenceID: 7509F414-54C3-5D88-9503-B05D159737CE; **Taxon:** scientificName: Meliboeus (Meliboeus) fulgidicollis (Lucas, 1846); order: Coleoptera; family: Buprestidae; genus: Meliboeus; subgenus: Meliboeus; specificEpithet: fulgidicollis; scientificNameAuthorship: (Lucas, 1846); **Location:** islandGroup: Tuscan Archipelago; island: Isola d'Elba; country: Italy; countryCode: IT; stateProvince: Livorno; county: Rio; municipality: Rio nell'Elba; locality: Le Secche; decimalLatitude: 42.815722; decimalLongitude: 10.373392; geodeticDatum: WGS84; coordinatePrecision: 0.0002; **Identification:** identifiedBy: L. Forbicioni; **Event:** eventDate: 2012-06-17; **Record Level:** collectionCode: LFPC
- **Type status:** Other material. **Occurrence:** individualCount: 1; lifeStage: adult; occurrenceID: 9AD6D7CB-9ECA-52A7-AAC7-5856ED1B99F4; **Taxon:** scientificName: Meliboeus (Meliboeus) fulgidicollis (Lucas, 1846); order: Coleoptera; family: Buprestidae; genus: Meliboeus; subgenus: Meliboeus; specificEpithet: fulgidicollis; scientificNameAuthorship: (Lucas, 1846); **Location:** islandGroup: Tuscan Archipelago; island: Isola d'Elba; country: Italy; countryCode: IT; stateProvince: Livorno; county: Marciana Marina; locality: Procchio; **Identification:** identifiedBy: G. Curletti; **Record Level:** source: Curletti G. (1994) I Buprestidi d’Italia. Catalogo geonemico, sinonimico, bibliografico, biologico. Monografie di Natura Bresciana, Ed. Vannini, Brescia, 19.

##### Conservation status

LC

##### Distribution

Recorded for the Tuscan Archipelago (Isola d'Elba) by Curletti 1994.

#### Meliboeus (Meliboeus) gibbicollis gibbicollis

(Illiger, 1803)

B59D7472-948E-55CF-A226-8629B89F90C4

##### Materials

- **Type status:** Other material. **Occurrence:** recordedBy: Leonardo Forbicioni; individualCount: 3; lifeStage: adult; occurrenceID: 795D3D07-07AE-5591-B827-BBBFBE1BC296; **Taxon:** scientificName: Meliboeus (Meliboeus) gibbicollis gibbicollis (Illiger, 1803); order: Coleoptera; family: Buprestidae; genus: Meliboeus; subgenus: Meliboeus; specificEpithet: gibbicollis; infraspecificEpithet: gibbicollis; scientificNameAuthorship: (Illiger, 1803); **Location:** islandGroup: Tuscan Archipelago; island: Isola d'Elba; country: Italy; countryCode: IT; stateProvince: Livorno; county: Portoferraio; locality: Punta della Rena; decimalLatitude: 42.805300; decimalLongitude: 10.317092; geodeticDatum: WGS84; coordinatePrecision: 0.0002; **Identification:** identifiedBy: E. Paggetti & F. Terzani; **Event:** eventDate: 2011-06-29; **Record Level:** collectionCode: LFPC
- **Type status:** Other material. **Occurrence:** recordedBy: Leonardo Forbicioni; individualCount: 1; lifeStage: adult; occurrenceID: 58E3F28E-5837-5355-B5EC-51ED8C51F105; **Taxon:** scientificName: Meliboeus (Meliboeus) gibbicollis gibbicollis (Illiger, 1803); order: Coleoptera; family: Buprestidae; genus: Meliboeus; subgenus: Meliboeus; specificEpithet: gibbicollis; infraspecificEpithet: gibbicollis; scientificNameAuthorship: (Illiger, 1803); **Location:** islandGroup: Tuscan Archipelago; island: Isola d'Elba; country: Italy; countryCode: IT; stateProvince: Livorno; county: Capoliveri; locality: Norsi; decimalLatitude: 42.768553; decimalLongitude: 10.346482; geodeticDatum: WGS84; coordinatePrecision: 0.0002; **Identification:** identifiedBy: E. Paggetti & F. Ceccolini; **Event:** eventDate: 2011-06-10; **Record Level:** collectionCode: LFPC
- **Type status:** Other material. **Occurrence:** recordedBy: Leonardo Forbicioni; individualCount: 1; lifeStage: adult; occurrenceID: 329B4D76-A91C-5FB0-BD3A-D7EBECC6B6AD; **Taxon:** scientificName: Meliboeus (Meliboeus) gibbicollis gibbicollis (Illiger, 1803); order: Coleoptera; family: Buprestidae; genus: Meliboeus; subgenus: Meliboeus; specificEpithet: gibbicollis; infraspecificEpithet: gibbicollis; scientificNameAuthorship: (Illiger, 1803); **Location:** islandGroup: Tuscan Archipelago; island: Isola d'Elba; country: Italy; countryCode: IT; stateProvince: Livorno; county: Portoferraio; locality: Colle Reciso/Mulino a vento; decimalLatitude: 42.777242; decimalLongitude: 10.288271; geodeticDatum: WGS84; coordinatePrecision: 0.0002; **Identification:** identifiedBy: L. Forbicioni; **Event:** eventDate: 2020-08-09; **Record Level:** collectionCode: LFPC
- **Type status:** Other material. **Occurrence:** recordedBy: Leonardo Forbicioni; individualCount: 1; lifeStage: adult; occurrenceID: F099D915-6580-539A-8931-B345C4122403; **Taxon:** scientificName: Meliboeus (Meliboeus) gibbicollis gibbicollis (Illiger, 1803); order: Coleoptera; family: Buprestidae; genus: Meliboeus; subgenus: Meliboeus; specificEpithet: gibbicollis; infraspecificEpithet: gibbicollis; scientificNameAuthorship: (Illiger, 1803); **Location:** islandGroup: Tuscan Archipelago; island: Isola d'Elba; country: Italy; countryCode: IT; stateProvince: Livorno; county: Capoliveri; locality: Norsi; decimalLatitude: 42.769667; decimalLongitude: 10.346222; geodeticDatum: WGS84; coordinatePrecision: 0.0002; **Identification:** identifiedBy: L. Forbicioni; **Event:** eventDate: 2013-06-03; **Record Level:** collectionCode: LFPC
- **Type status:** Other material. **Occurrence:** individualCount: 1; lifeStage: adult; occurrenceID: 549D9025-E93A-5226-9035-AED3476A95E0; **Taxon:** scientificName: Meliboeus (Meliboeus) gibbicollis gibbicollis (Illiger, 1803); order: Coleoptera; family: Buprestidae; genus: Meliboeus; subgenus: Meliboeus; specificEpithet: gibbicollis; infraspecificEpithet: gibbicollis; scientificNameAuthorship: (Illiger, 1803); **Location:** islandGroup: Tuscan Archipelago; island: Isola d'Elba; country: Italy; countryCode: IT; stateProvince: Livorno; county: Porto Azzurro; **Identification:** identifiedBy: G. Curletti; **Record Level:** source: Curletti G. (1994) I Buprestidi d’Italia. Catalogo geonemico, sinonimico, bibliografico, biologico. Monografie di Natura Bresciana, Ed. Vannini, Brescia, 19.
- **Type status:** Other material. **Occurrence:** individualCount: 1; lifeStage: adult; occurrenceID: 64D00FC4-B039-5C80-86B8-DA7D3D399D3F; **Taxon:** scientificName: Meliboeus (Meliboeus) gibbicollis gibbicollis (Illiger, 1803); order: Coleoptera; family: Buprestidae; genus: Meliboeus; subgenus: Meliboeus; specificEpithet: gibbicollis; infraspecificEpithet: gibbicollis; scientificNameAuthorship: (Illiger, 1803); **Location:** islandGroup: Tuscan Archipelago; island: Isola d'Elba; country: Italy; countryCode: IT; stateProvince: Livorno; county: Marciana Marina; locality: Procchio; **Identification:** identifiedBy: G. Curletti; **Record Level:** source: Curletti G. (1994) I Buprestidi d’Italia. Catalogo geonemico, sinonimico, bibliografico, biologico. Monografie di Natura Bresciana, Ed. Vannini, Brescia, 19.

##### Distribution

Recorded for the Tuscan Archipelago (Isola d'Elba) by Curletti (1994).

#### Meliboeus (Meliboeus) graminis graminis

(Panzer, 1789)

40E5B46E-660A-5FAF-9350-D364F1DE6FF5

##### Materials

- **Type status:** Other material. **Occurrence:** recordedBy: Leonardo Forbicioni; individualCount: 1; lifeStage: adult; occurrenceID: E543571B-37C9-588A-A588-43CFD3BAEABF; **Taxon:** scientificName: Meliboeus (Meliboeus) graminis graminis (Panzer, 1789); order: Coleoptera; family: Buprestidae; genus: Meliboeus; subgenus: Meliboeus; specificEpithet: graminis; infraspecificEpithet: graminis; scientificNameAuthorship: (Panzer, 1789); **Location:** islandGroup: Tuscan Archipelago; island: Isola d'Elba; country: Italy; countryCode: IT; stateProvince: Livorno; county: Capoliveri; locality: Norsi; decimalLatitude: 42.768477; decimalLongitude: 10.346521; geodeticDatum: WGS84; coordinatePrecision: 0.0002; **Identification:** identifiedBy: E. Paggetti; **Event:** eventDate: 2011-06-10; **Record Level:** collectionCode: LFPC
- **Type status:** Other material. **Occurrence:** recordedBy: Leonardo Forbicioni; individualCount: 2; lifeStage: adult; occurrenceID: 155C9E69-DA74-557F-A943-A163003F307D; **Taxon:** scientificName: Meliboeus (Meliboeus) graminis graminis (Panzer, 1789); order: Coleoptera; family: Buprestidae; genus: Meliboeus; subgenus: Meliboeus; specificEpithet: graminis; infraspecificEpithet: graminis; scientificNameAuthorship: (Panzer, 1789); **Location:** islandGroup: Tuscan Archipelago; island: Isola d'Elba; country: Italy; countryCode: IT; stateProvince: Livorno; county: Capoliveri; locality: Norsi; decimalLatitude: 42.767397; decimalLongitude: 10.347045; geodeticDatum: WGS84; coordinatePrecision: 0.0002; **Identification:** identifiedBy: L. Forbicioni; **Event:** eventDate: 2013-06-03; **Record Level:** collectionCode: LFPC
- **Type status:** Other material. **Occurrence:** recordedBy: Leonardo Forbicioni; individualCount: 1; lifeStage: adult; occurrenceID: 9E49F96F-E518-5F27-9663-381A426231F5; **Taxon:** scientificName: Meliboeus (Meliboeus) graminis graminis (Panzer, 1789); order: Coleoptera; family: Buprestidae; genus: Meliboeus; subgenus: Meliboeus; specificEpithet: graminis; infraspecificEpithet: graminis; scientificNameAuthorship: (Panzer, 1789); **Location:** islandGroup: Tuscan Archipelago; island: Isola d'Elba; country: Italy; countryCode: IT; stateProvince: Livorno; county: Capoliveri; locality: Sassi Neri; decimalLatitude: 42.738017; decimalLongitude: 10.426172; geodeticDatum: WGS84; coordinatePrecision: 0.0002; **Identification:** identifiedBy: E. Paggetti; **Event:** eventDate: 2014-07-28; **Record Level:** collectionCode: LFPC

#### Tracheini

8CCBC3B4-CB82-58F4-8025-C7A4867131F5

#### Trachys puncticollis rectilineatus

Abeille de Perrin, 1900

E08985C2-6ECF-5E37-8230-9CDDE0645A64

##### Materials

- **Type status:** Other material. **Occurrence:** recordedBy: Leonardo Forbicioni; individualCount: 1; lifeStage: adult; occurrenceID: 1FF27A07-49FA-5D1A-80B7-EFE2F3A4F712; **Taxon:** scientificName: Trachyspuncticollisrectilineatus Abeille de Perrin, 1900; order: Coleoptera; family: Buprestidae; genus: Trachys; specificEpithet: puncticollis; infraspecificEpithet: rectilineatus; scientificNameAuthorship: Abeille de Perrin, 1900; **Location:** islandGroup: Tuscan Archipelago; island: Isola d'Elba; country: Italy; countryCode: IT; stateProvince: Livorno; county: Capoliveri; locality: Buraccio; decimalLatitude: 42.780248; decimalLongitude: 10.352027; geodeticDatum: WGS84; coordinatePrecision: 0.0002; **Identification:** identifiedBy: L. Forbicioni; **Event:** eventDate: 2013-10-11; **Record Level:** collectionCode: LFPC
- **Type status:** Other material. **Occurrence:** recordedBy: Leonardo Forbicioni; individualCount: 1; lifeStage: adult; occurrenceID: 4CD4B828-2F0D-5840-82D5-B61ED56FA72F; **Taxon:** scientificName: Trachyspuncticollisrectilineatus Abeille de Perrin, 1900; order: Coleoptera; family: Buprestidae; genus: Trachys; specificEpithet: puncticollis; infraspecificEpithet: rectilineatus; scientificNameAuthorship: Abeille de Perrin, 1900; **Location:** islandGroup: Tuscan Archipelago; island: Isola d'Elba; country: Italy; countryCode: IT; stateProvince: Livorno; county: Capoliveri; locality: Pian di Mola; decimalLatitude: 42.759141; decimalLongitude: 10.367668; geodeticDatum: WGS84; coordinatePrecision: 0.0002; **Identification:** identifiedBy: E. Paggetti & F. Ceccolini; **Event:** eventDate: 2011-07-02; **Record Level:** collectionCode: LFPC

#### Trachys troglodytiformis

Obenberger, 1918

90116B30-4E63-5154-9149-E101BE850D56

##### Materials

- **Type status:** Other material. **Occurrence:** recordedBy: Leonardo Forbicioni; individualCount: 5; lifeStage: adult; occurrenceID: 6470D06C-1C3A-5141-9305-1D1206C4554B; **Taxon:** scientificName: Trachystroglodytiformis Obenberger, 1918; order: Coleoptera; family: Buprestidae; genus: Trachys; specificEpithet: troglodytiformis; scientificNameAuthorship: Obenberger, 1918; **Location:** islandGroup: Tuscan Archipelago; island: Isola d'Elba; country: Italy; countryCode: IT; stateProvince: Livorno; county: Campo nell'Elba; municipality: San Piero; decimalLatitude: 42.752231; decimalLongitude: 10.203791; geodeticDatum: WGS84; coordinatePrecision: 0.0002; **Identification:** identifiedBy: E. Paggetti; **Event:** eventDate: 2011-04-27; **Record Level:** collectionCode: LFPC
- **Type status:** Other material. **Occurrence:** recordedBy: Leonardo Forbicioni; individualCount: 2; lifeStage: adult; occurrenceID: B179CE0E-66EF-535F-91D8-104FBDD61F29; **Taxon:** scientificName: Trachystroglodytiformis Obenberger, 1918; order: Coleoptera; family: Buprestidae; genus: Trachys; specificEpithet: troglodytiformis; scientificNameAuthorship: Obenberger, 1918; **Location:** islandGroup: Tuscan Archipelago; island: Isola d'Elba; country: Italy; countryCode: IT; stateProvince: Livorno; county: Campo nell'Elba; municipality: San Piero; locality: Piana al Canale; decimalLatitude: 42.745657; decimalLongitude: 10.187069; geodeticDatum: WGS84; coordinatePrecision: 0.0002; **Identification:** identifiedBy: L. Forbicioni; **Event:** eventDate: 2015-04-26; **Record Level:** collectionCode: LFPC
- **Type status:** Other material. **Occurrence:** recordedBy: Leonardo Forbicioni; individualCount: 1; lifeStage: adult; occurrenceID: 363D7903-5A97-5876-BA4B-2C1ACB0986F1; **Taxon:** scientificName: Trachystroglodytiformis Obenberger, 1918; order: Coleoptera; family: Buprestidae; genus: Trachys; specificEpithet: troglodytiformis; scientificNameAuthorship: Obenberger, 1918; **Location:** islandGroup: Tuscan Archipelago; island: Isola d'Elba; country: Italy; countryCode: IT; stateProvince: Livorno; county: Portoferraio; locality: Acquaviva; decimalLatitude: 42.820232; decimalLongitude: 10.287908; geodeticDatum: WGS84; coordinatePrecision: 0.0002; **Identification:** identifiedBy: E. Paggetti; **Event:** eventDate: 2014-06-18; **Record Level:** collectionCode: LFPC
- **Type status:** Other material. **Occurrence:** recordedBy: Leonardo Forbicioni; individualCount: 1; lifeStage: adult; occurrenceID: 8ECCCBF8-8464-51F3-ACFB-9C86C92E2E84; **Taxon:** scientificName: Trachystroglodytiformis Obenberger, 1918; order: Coleoptera; family: Buprestidae; genus: Trachys; specificEpithet: troglodytiformis; scientificNameAuthorship: Obenberger, 1918; **Location:** islandGroup: Tuscan Archipelago; island: Isola d'Elba; country: Italy; countryCode: IT; stateProvince: Livorno; county: Portoferraio; locality: San Giovanni; decimalLatitude: 42.796295; decimalLongitude: 10.345902; geodeticDatum: WGS84; coordinatePrecision: 0.0002; **Identification:** identifiedBy: L. Forbicioni; **Event:** eventDate: 2018-05-14; **Record Level:** collectionCode: LFPC
- **Type status:** Other material. **Occurrence:** recordedBy: Leonardo Forbicioni; individualCount: 2; lifeStage: adult; occurrenceID: AE535460-5217-5D62-8064-BE772CA982B1; **Taxon:** scientificName: Trachystroglodytiformis Obenberger, 1918; order: Coleoptera; family: Buprestidae; genus: Trachys; specificEpithet: troglodytiformis; scientificNameAuthorship: Obenberger, 1918; **Location:** islandGroup: Tuscan Archipelago; island: Isola d'Elba; country: Italy; countryCode: IT; stateProvince: Livorno; county: Portoferraio; locality: San Giovanni; decimalLatitude: 42.801466; decimalLongitude: 10.322153; geodeticDatum: WGS84; coordinatePrecision: 0.0002; **Identification:** identifiedBy: E. Paggetti; **Event:** eventDate: 2012-03-28; **Record Level:** collectionCode: LFPC
- **Type status:** Other material. **Occurrence:** recordedBy: Leonardo Forbicioni; individualCount: 1; lifeStage: adult; occurrenceID: E9AAAFD3-2657-5E2A-B8F2-A2DF2AFA9A57; **Taxon:** scientificName: Trachystroglodytiformis Obenberger, 1918; order: Coleoptera; family: Buprestidae; genus: Trachys; specificEpithet: troglodytiformis; scientificNameAuthorship: Obenberger, 1918; **Location:** islandGroup: Tuscan Archipelago; island: Isola d'Elba; country: Italy; countryCode: IT; stateProvince: Livorno; county: Porto Azzurro; locality: Mola-Spiaggia; decimalLatitude: 42.760618; decimalLongitude: 10.385796; geodeticDatum: WGS84; coordinatePrecision: 0.0002; **Identification:** identifiedBy: E. Paggetti; **Event:** eventDate: 2014-08-16; **Record Level:** collectionCode: LFPC

#### Buprestinae

2B265936-52AF-5CA4-91AE-AFC599EF30A8

#### Athaxiini

87474DEE-4EC1-5EA9-8231-6BD54F3ACCA9

#### Anthaxia (Anthaxia) chevrieri

Gory & Laporte, 1839

3CF28BA3-F889-5CB3-BF8A-D3137A114A9D

##### Materials

- **Type status:** Other material. **Occurrence:** recordedBy: Leonardo Forbicioni; individualCount: 1; lifeStage: adult; occurrenceID: 601D3085-4FED-5DA3-859C-4894B0D4B2C5; **Taxon:** scientificName: Anthaxia (Anthaxia) chevrieri Gory & Laporte, 1839; order: Coleoptera; family: Buprestidae; genus: Trachys; subgenus: Anthaxia; specificEpithet: chevrieri; scientificNameAuthorship: Gory & Laporte, 1839; **Location:** islandGroup: Tuscan Archipelago; island: Isola d'Elba; country: Italy; countryCode: IT; stateProvince: Livorno; county: Portoferraio; locality: Monte Orello; decimalLatitude: 42.773876; decimalLongitude: 10.334036; geodeticDatum: WGS84; coordinatePrecision: 0.0002; **Identification:** identifiedBy: E. Paggetti; **Event:** eventDate: 2011-05-07; **Record Level:** collectionCode: LFPC
- **Type status:** Other material. **Occurrence:** recordedBy: Leonardo Forbicioni; individualCount: 1; lifeStage: adult; occurrenceID: A86CAB47-F428-506E-9028-A40F8418A185; **Taxon:** scientificName: Anthaxia (Anthaxia) chevrieri Gory & Laporte, 1839; order: Coleoptera; family: Buprestidae; genus: Anthaxia; subgenus: Anthaxia; specificEpithet: chevrieri; scientificNameAuthorship: Gory & Laporte, 1839; **Location:** islandGroup: Tuscan Archipelago; island: Isola d'Elba; country: Italy; countryCode: IT; stateProvince: Livorno; county: Portoferraio; locality: Monte Orello; decimalLatitude: 42.780318; decimalLongitude: 10.326759; geodeticDatum: WGS84; coordinatePrecision: 0.0002; **Identification:** identifiedBy: G. Curletti; **Event:** eventDate: 2013-04-25; **Record Level:** collectionCode: LFPC

##### Conservation status

LC

#### Anthaxia (Anthaxia) mendizabali

Cobos, 1965

D118DDA3-EAE2-529E-9923-956405846178

##### Materials

- **Type status:** Other material. **Occurrence:** recordedBy: Leonardo Forbicioni; individualCount: 1; lifeStage: adult; occurrenceID: 236AEEF1-4780-5CAE-A374-441925605E00; **Taxon:** scientificName: Anthaxia (Anthaxia) mendizabali Cobos, 1965; order: Coleoptera; family: Buprestidae; genus: Anthaxia; subgenus: Anthaxia; specificEpithet: mendizabali; scientificNameAuthorship: Cobos, 1965; **Location:** islandGroup: Tuscan Archipelago; island: Isola d'Elba; country: Italy; countryCode: IT; stateProvince: Livorno; county: Campo nell'Elba; municipality: Sant'Ilario; locality: Via della Madonnina; decimalLatitude: 42.763056; decimalLongitude: 10.224167; geodeticDatum: WGS84; coordinatePrecision: 0.0002; **Identification:** identifiedBy: G. Curletti; **Event:** eventDate: 2014-06-22; **Record Level:** collectionCode: LFPC
- **Type status:** Other material. **Occurrence:** recordedBy: Leonardo Forbicioni; individualCount: 1; lifeStage: adult; occurrenceID: 2BBABC28-A652-52DD-A340-4C167163B7E5; **Taxon:** scientificName: Anthaxia (Anthaxia) mendizabali Cobos, 1965; order: Coleoptera; family: Buprestidae; genus: Anthaxia; subgenus: Anthaxia; specificEpithet: mendizabali; scientificNameAuthorship: Cobos, 1965; **Location:** islandGroup: Tuscan Archipelago; island: Isola d'Elba; country: Italy; countryCode: IT; stateProvince: Livorno; county: Campo nell'Elba; municipality: Sant'Ilario; decimalLatitude: 42.763232; decimalLongitude: 10.221358; geodeticDatum: WGS84; coordinatePrecision: 0.0002; **Identification:** identifiedBy: E. Paggetti; **Event:** eventDate: 2014-06-01; **Record Level:** collectionCode: LFPC

##### Conservation status

LC

#### Anthaxia (Anthaxia) thalassophila thalassophila

Abeille de Perrin, 1900

FF2AB620-2996-55DC-AB0A-0546DCE96B6C

##### Materials

- **Type status:** Other material. **Occurrence:** recordedBy: Leonardo Forbicioni; individualCount: 4; lifeStage: adult; occurrenceID: 7EE735DA-43C4-5EE6-BCE1-BB442A66B49C; **Taxon:** scientificName: Anthaxia (Anthaxia) thalassophila thalassophila Abeille de Perrin, 1900; order: Coleoptera; family: Buprestidae; genus: Anthaxia; subgenus: Anthaxia; specificEpithet: thalassophila; infraspecificEpithet: thalassophila; scientificNameAuthorship: Abeille de Perrin, 1900; **Location:** islandGroup: Tuscan Archipelago; island: Isola d'Elba; country: Italy; countryCode: IT; stateProvince: Livorno; county: Rio; municipality: Rio nell'Elba; locality: Bagnaia; decimalLatitude: 42.809239; decimalLongitude: 10.374455; geodeticDatum: WGS84; coordinatePrecision: 0.0002; **Identification:** identifiedBy: E. Paggetti; **Event:** eventDate: 2012-06-17; **Record Level:** collectionCode: LFPC
- **Type status:** Other material. **Occurrence:** recordedBy: Leonardo Forbicioni; individualCount: 2; lifeStage: adult; occurrenceID: A9747471-89E0-56E1-AA6D-BFA174DD4425; **Taxon:** scientificName: Anthaxia (Anthaxia) thalassophila thalassophila Abeille de Perrin, 1900; order: Coleoptera; family: Buprestidae; genus: Anthaxia; subgenus: Anthaxia; specificEpithet: thalassophila; infraspecificEpithet: thalassophila; scientificNameAuthorship: Abeille de Perrin, 1900; **Location:** islandGroup: Tuscan Archipelago; island: Isola d'Elba; country: Italy; countryCode: IT; stateProvince: Livorno; county: Portoferraio; locality: Val di Piano; decimalLatitude: 42.790612; decimalLongitude: 10.372348; geodeticDatum: WGS84; coordinatePrecision: 0.0002; **Identification:** identifiedBy: E. Paggetti; **Event:** eventDate: 2011-06-05; **Record Level:** collectionCode: LFPC
- **Type status:** Other material. **Occurrence:** recordedBy: Leonardo Forbicioni; individualCount: 1; lifeStage: adult; occurrenceID: 405939E3-2933-5124-8E5F-09C75AB35E52; **Taxon:** scientificName: Anthaxia (Anthaxia) thalassophila thalassophila Abeille de Perrin, 1900; order: Coleoptera; family: Buprestidae; genus: Anthaxia; subgenus: Anthaxia; specificEpithet: thalassophila; infraspecificEpithet: thalassophila; scientificNameAuthorship: Abeille de Perrin, 1900; **Location:** islandGroup: Tuscan Archipelago; island: Isola d'Elba; country: Italy; countryCode: IT; stateProvince: Livorno; county: Portoferraio; locality: Volterraio; decimalLatitude: 42.801391; decimalLongitude: 10.380774; geodeticDatum: WGS84; coordinatePrecision: 0.0002; **Identification:** identifiedBy: E. Paggetti; **Event:** eventDate: 2011-06-15; **Record Level:** collectionCode: LFPC
- **Type status:** Other material. **Occurrence:** recordedBy: Leonardo Forbicioni; individualCount: 1; lifeStage: adult; occurrenceID: 40320D75-88CF-556D-A3BE-629284B790FC; **Taxon:** scientificName: Anthaxia (Anthaxia) thalassophila thalassophila Abeille de Perrin, 1900; order: Coleoptera; family: Buprestidae; genus: Anthaxia; subgenus: Anthaxia; specificEpithet: thalassophila; infraspecificEpithet: thalassophila; scientificNameAuthorship: Abeille de Perrin, 1900; **Location:** islandGroup: Tuscan Archipelago; island: Isola d'Elba; country: Italy; countryCode: IT; stateProvince: Livorno; county: Porto Azzurro; locality: Monte Castello; decimalLatitude: 42.786514; decimalLongitude: 10.385741; geodeticDatum: WGS84; coordinatePrecision: 0.0002; **Identification:** identifiedBy: E. Paggetti; **Event:** eventDate: 2014-06-11; **Record Level:** collectionCode: LFPC
- **Type status:** Other material. **Occurrence:** recordedBy: Leonardo Forbicioni; individualCount: 5; lifeStage: adult; occurrenceID: E6672968-BC13-515D-A69E-9C234E55313E; **Taxon:** scientificName: Anthaxia (Anthaxia) thalassophila thalassophila Abeille de Perrin, 1900; order: Coleoptera; family: Buprestidae; genus: Anthaxia; subgenus: Anthaxia; specificEpithet: thalassophila; infraspecificEpithet: thalassophila; scientificNameAuthorship: Abeille de Perrin, 1900; **Location:** islandGroup: Tuscan Archipelago; island: Isola d'Elba; country: Italy; countryCode: IT; stateProvince: Livorno; county: Porto Azzurro; locality: Monserrato; decimalLatitude: 42.783960; decimalLongitude: 10.392627; geodeticDatum: WGS84; coordinatePrecision: 0.0002; **Identification:** identifiedBy: L. Forbicioni; **Event:** eventDate: 2014-06-06; **Record Level:** collectionCode: LFPC
- **Type status:** Other material. **Occurrence:** recordedBy: Leonardo Forbicioni; individualCount: 2; lifeStage: adult; occurrenceID: F8DAD650-63D2-553E-B31C-A01BE3C0CC42; **Taxon:** scientificName: Anthaxia (Anthaxia) thalassophila thalassophila Abeille de Perrin, 1900; order: Coleoptera; family: Buprestidae; genus: Anthaxia; subgenus: Anthaxia; specificEpithet: thalassophila; infraspecificEpithet: thalassophila; scientificNameAuthorship: Abeille de Perrin, 1900; **Location:** islandGroup: Tuscan Archipelago; island: Isola d'Elba; country: Italy; countryCode: IT; stateProvince: Livorno; county: Porto Azzurro; locality: Buraccio; decimalLatitude: 42.780262; decimalLongitude: 10.351439; geodeticDatum: WGS84; coordinatePrecision: 0.0002; **Identification:** identifiedBy: L. Forbicioni; **Event:** eventDate: 2022-06-13; **Record Level:** collectionCode: LFPC
- **Type status:** Other material. **Occurrence:** recordedBy: Leonardo Forbicioni; individualCount: 2; lifeStage: adult; occurrenceID: 07B5BBDC-145A-58A0-890C-86E2E89EDA1B; **Taxon:** scientificName: Anthaxia (Anthaxia) thalassophila thalassophila Abeille de Perrin, 1900; order: Coleoptera; family: Buprestidae; genus: Anthaxia; subgenus: Anthaxia; specificEpithet: thalassophila; infraspecificEpithet: thalassophila; scientificNameAuthorship: Abeille de Perrin, 1900; **Location:** islandGroup: Tuscan Archipelago; island: Isola d'Elba; country: Italy; countryCode: IT; stateProvince: Livorno; county: Capoliveri; locality: Lacona (dune); decimalLatitude: 42.760011; decimalLongitude: 10.307320; geodeticDatum: WGS84; coordinatePrecision: 0.0002; **Identification:** identifiedBy: L. Forbicioni; **Event:** eventDate: 2014-05-28; **Record Level:** collectionCode: LFPC
- **Type status:** Other material. **Occurrence:** recordedBy: Leonardo Forbicioni; individualCount: 4; lifeStage: adult; occurrenceID: CD7FEA75-60A8-5A9D-95DD-8641136338B0; **Taxon:** scientificName: Anthaxia (Anthaxia) thalassophila thalassophila Abeille de Perrin, 1900; order: Coleoptera; family: Buprestidae; genus: Anthaxia; subgenus: Anthaxia; specificEpithet: thalassophila; infraspecificEpithet: thalassophila; scientificNameAuthorship: Abeille de Perrin, 1900; **Location:** islandGroup: Tuscan Archipelago; island: Isola d'Elba; country: Italy; countryCode: IT; stateProvince: Livorno; county: Campo nell'Elba; locality: Monte Tambone; decimalLatitude: 42.755946; decimalLongitude: 10.272602; geodeticDatum: WGS84; coordinatePrecision: 0.0002; **Identification:** identifiedBy: G. Curletti; **Event:** eventDate: 2017-05-28; **Record Level:** collectionCode: LFPC
- **Type status:** Other material. **Occurrence:** individualCount: 1; lifeStage: adult; occurrenceID: 4D0A989A-13AA-5068-BC86-C4A6C894922B; **Taxon:** scientificName: Anthaxia (Anthaxia) thalassophila thalassophila Abeille de Perrin, 1900; order: Coleoptera; family: Buprestidae; genus: Anthaxia; subgenus: Anthaxia; specificEpithet: thalassophila; infraspecificEpithet: thalassophila; scientificNameAuthorship: Abeille de Perrin, 1900; **Location:** islandGroup: Tuscan Archipelago; island: Isola d'Elba; country: Italy; countryCode: IT; stateProvince: Livorno; **Identification:** identifiedBy: G. Curletti; **Record Level:** source: Curletti G. (1994) I Buprestidi d’Italia. Catalogo geonemico, sinonimico, bibliografico, biologico. Monografie di Natura Bresciana, Ed. Vannini, Brescia, 19.
- **Type status:** Other material. **Occurrence:** individualCount: 1; lifeStage: adult; occurrenceID: 0C1503D4-F360-5F3A-BD26-AEEF8685CDA8; **Taxon:** scientificName: Anthaxia (Anthaxia) thalassophila thalassophila Abeille de Perrin, 1900; order: Coleoptera; family: Buprestidae; genus: Anthaxia; subgenus: Anthaxia; specificEpithet: thalassophila; infraspecificEpithet: thalassophila; scientificNameAuthorship: Abeille de Perrin, 1900; **Location:** islandGroup: Tuscan Archipelago; island: Isola del Giglio; country: Italy; countryCode: GR; stateProvince: Grosseto; **Identification:** identifiedBy: G. Curletti; **Record Level:** source: Curletti G. (1994) I Buprestidi d’Italia. Catalogo geonemico, sinonimico, bibliografico, biologico. Monografie di Natura Bresciana, Ed. Vannini, Brescia, 19.

##### Conservation status

LC

##### Distribution

Recorded for the Tuscan Archipelago (Isola d'Elba and Isola del Giglio) by Curletti (1994).

#### Anthaxia (Haplanthaxia) croesus

(Villers, 1789)

447E7EDD-486C-525D-A7AB-C8882363A219

##### Materials

- **Type status:** Other material. **Occurrence:** recordedBy: Leonardo Forbicioni; individualCount: 5; lifeStage: adult; occurrenceID: B7855576-9477-5BBB-9820-5D45E2216F0C; **Taxon:** scientificName: Anthaxia (Haplanthaxia) croesus (Villers, 1789); order: Coleoptera; family: Buprestidae; genus: Anthaxia; subgenus: Haplanthaxia; specificEpithet: croesus; scientificNameAuthorship: (Villers, 1789); **Location:** islandGroup: Tuscan Archipelago; island: Isola d'Elba; country: Italy; countryCode: IT; stateProvince: Livorno; county: Portoferraio; locality: Acquabona; decimalLatitude: 42.786503; decimalLongitude: 10.346013; geodeticDatum: WGS84; coordinatePrecision: 0.0002; **Identification:** identifiedBy: E. Paggetti; **Event:** eventDate: 2011-05-20; **Record Level:** collectionCode: LFPC
- **Type status:** Other material. **Occurrence:** recordedBy: Giuliano Frangini; individualCount: 3; lifeStage: adult; occurrenceID: F81035C8-087F-55D3-BA26-1CCA857024D3; **Taxon:** scientificName: Anthaxia (Haplanthaxia) croesus (Villers, 1789); order: Coleoptera; family: Buprestidae; genus: Anthaxia; subgenus: Haplanthaxia; specificEpithet: croesus; scientificNameAuthorship: (Villers, 1789); **Location:** islandGroup: Tuscan Archipelago; island: Isola d'Elba; country: Italy; countryCode: IT; stateProvince: Livorno; county: Portoferraio; locality: Colle Reciso; decimalLatitude: 42.785997; decimalLongitude: 10.318354; geodeticDatum: WGS84; coordinatePrecision: 0.0002; **Identification:** identifiedBy: E. Paggetti; **Event:** eventDate: 2010-05-28; **Record Level:** collectionCode: LFPC
- **Type status:** Other material. **Occurrence:** recordedBy: Leonardo Forbicioni; individualCount: 1; lifeStage: adult; occurrenceID: 2EB57A3D-4967-5F23-A056-EF9EE3544FA4; **Taxon:** scientificName: Anthaxia (Haplanthaxia) croesus (Villers, 1789); order: Coleoptera; family: Buprestidae; genus: Anthaxia; subgenus: Haplanthaxia; specificEpithet: croesus; scientificNameAuthorship: (Villers, 1789); **Location:** islandGroup: Tuscan Archipelago; island: Isola d'Elba; country: Italy; countryCode: IT; stateProvince: Livorno; county: Campo nell'Elba; municipality: San Piero; locality: San Francesco; decimalLatitude: 42.765005; decimalLongitude: 10.196880; geodeticDatum: WGS84; coordinatePrecision: 0.0002; **Identification:** identifiedBy: E. Paggetti; **Event:** eventDate: 2013-06-19; **Record Level:** collectionCode: LFPC
- **Type status:** Other material. **Occurrence:** recordedBy: Leonardo Forbicioni; individualCount: 1; lifeStage: adult; occurrenceID: 0A7C6BBE-07CC-58C1-B77A-2C0FCB1D8709; **Taxon:** scientificName: Anthaxia (Haplanthaxia) croesus (Villers, 1789); order: Coleoptera; family: Buprestidae; genus: Anthaxia; subgenus: Haplanthaxia; specificEpithet: croesus; scientificNameAuthorship: (Villers, 1789); **Location:** islandGroup: Tuscan Archipelago; island: Isola d'Elba; country: Italy; countryCode: IT; stateProvince: Livorno; county: Campo nell'Elba; locality: Monte Perone; decimalLatitude: 42.777768; decimalLongitude: 10.202310; geodeticDatum: WGS84; coordinatePrecision: 0.0002; **Identification:** identifiedBy: L. Forbicioni; **Event:** eventDate: 2013-05-07; **Record Level:** collectionCode: LFPC
- **Type status:** Other material. **Occurrence:** recordedBy: Leonardo Forbicioni; individualCount: 2; lifeStage: adult; occurrenceID: FAE35A2A-36A9-5476-A22E-D032704F350B; **Taxon:** scientificName: Anthaxia (Haplanthaxia) croesus (Villers, 1789); order: Coleoptera; family: Buprestidae; genus: Anthaxia; subgenus: Haplanthaxia; specificEpithet: croesus; scientificNameAuthorship: (Villers, 1789); **Location:** islandGroup: Tuscan Archipelago; island: Isola d'Elba; country: Italy; countryCode: IT; stateProvince: Livorno; county: Portoferraio; locality: Acquabona; decimalLatitude: 42.783300; decimalLongitude: 10.336332; geodeticDatum: WGS84; coordinatePrecision: 0.0002; **Identification:** identifiedBy: E. Paggetti; **Event:** eventDate: 2012-06-02; **Record Level:** collectionCode: LFPC
- **Type status:** Other material. **Occurrence:** recordedBy: Leonardo Forbicioni; individualCount: 1; lifeStage: adult; occurrenceID: D2662A6E-4FEA-54D9-9ECF-C83D220A12CB; **Taxon:** scientificName: Anthaxia (Haplanthaxia) croesus (Villers, 1789); order: Coleoptera; family: Buprestidae; genus: Anthaxia; subgenus: Haplanthaxia; specificEpithet: croesus; scientificNameAuthorship: (Villers, 1789); **Location:** islandGroup: Tuscan Archipelago; island: Isola del Giglio; country: Italy; countryCode: IT; stateProvince: Grosseto; county: Giglio Castello; locality: Colle della Pagana; decimalLatitude: 42.352266; decimalLongitude: 10.904441; geodeticDatum: WGS84; coordinatePrecision: 0.0002; **Identification:** identifiedBy: E. Paggetti; **Event:** eventDate: 17-19/05/2012; **Record Level:** collectionCode: LFPC
- **Type status:** Other material. **Occurrence:** recordedBy: Leonardo Forbicioni; individualCount: 1; lifeStage: adult; occurrenceID: 45FC9911-F3D5-5118-A2B6-573ECB05E516; **Taxon:** scientificName: Anthaxia (Haplanthaxia) croesus (Villers, 1789); order: Coleoptera; family: Buprestidae; genus: Anthaxia; subgenus: Haplanthaxia; specificEpithet: croesus; scientificNameAuthorship: (Villers, 1789); **Location:** islandGroup: Tuscan Archipelago; island: Isola d'Elba; country: Italy; countryCode: IT; stateProvince: Livorno; county: Porto Azzurro; locality: Buraccio/Lo Stipito; decimalLatitude: 42.761498; decimalLongitude: 10.365277; geodeticDatum: WGS84; coordinatePrecision: 0.0002; **Identification:** identifiedBy: E. Paggetti; **Event:** eventDate: 2011-06-30; **Record Level:** collectionCode: LFPC
- **Type status:** Other material. **Occurrence:** recordedBy: Leonardo Forbicioni; individualCount: 1; lifeStage: adult; occurrenceID: BA23F6B5-B224-555E-9867-BA31A0D38A69; **Taxon:** scientificName: Anthaxia (Haplanthaxia) croesus (Villers, 1789); order: Coleoptera; family: Buprestidae; genus: Anthaxia; subgenus: Haplanthaxia; specificEpithet: croesus; scientificNameAuthorship: (Villers, 1789); **Location:** islandGroup: Tuscan Archipelago; island: Isola d'Elba; country: Italy; countryCode: IT; stateProvince: Livorno; county: Portoferraio; locality: Monte Poppe; decimalLatitude: 42.797932; decimalLongitude: 10.276094; geodeticDatum: WGS84; coordinatePrecision: 0.0002; **Identification:** identifiedBy: E. Paggetti; **Event:** eventDate: 2011-06-20; **Record Level:** collectionCode: LFPC
- **Type status:** Other material. **Occurrence:** recordedBy: Leonardo Forbicioni; individualCount: 1; lifeStage: adult; occurrenceID: FD77D289-93E0-5A0D-8FEC-87D61EA231D5; **Taxon:** scientificName: Anthaxia (Haplanthaxia) croesus (Villers, 1789); order: Coleoptera; family: Buprestidae; genus: Anthaxia; subgenus: Haplanthaxia; specificEpithet: croesus; scientificNameAuthorship: (Villers, 1789); **Location:** islandGroup: Tuscan Archipelago; island: Isola d'Elba; country: Italy; countryCode: IT; stateProvince: Livorno; county: Portoferraio; locality: Colle Reciso; decimalLatitude: 42.785734; decimalLongitude: 10.318822; geodeticDatum: WGS84; coordinatePrecision: 0.0002; **Identification:** identifiedBy: E. Paggetti; **Event:** eventDate: 2011-05-30; **Record Level:** collectionCode: LFPC
- **Type status:** Other material. **Occurrence:** recordedBy: Leonardo Forbicioni; individualCount: 1; lifeStage: adult; occurrenceID: 1AA27026-726C-59D0-ADB5-64E8E4880DD5; **Taxon:** scientificName: Anthaxia (Haplanthaxia) croesus (Villers, 1789); order: Coleoptera; family: Buprestidae; genus: Anthaxia; subgenus: Haplanthaxia; specificEpithet: croesus; scientificNameAuthorship: (Villers, 1789); **Location:** islandGroup: Tuscan Archipelago; island: Isola d'Elba; country: Italy; countryCode: IT; stateProvince: Livorno; county: Capoliveri; locality: Norsi; decimalLatitude: 42.770184; decimalLongitude: 10.346211; geodeticDatum: WGS84; coordinatePrecision: 0.0002; **Identification:** identifiedBy: E. Paggetti; **Event:** eventDate: 2011-06-10; **Record Level:** collectionCode: LFPC
- **Type status:** Other material. **Occurrence:** recordedBy: Leonardo Forbicioni; individualCount: 1; lifeStage: adult; occurrenceID: 81BF1EB6-167B-5516-9C92-5364B4D7920C; **Taxon:** scientificName: Anthaxia (Haplanthaxia) croesus (Villers, 1789); order: Coleoptera; family: Buprestidae; genus: Anthaxia; subgenus: Haplanthaxia; specificEpithet: croesus; scientificNameAuthorship: (Villers, 1789); **Location:** islandGroup: Tuscan Archipelago; island: Isola d'Elba; country: Italy; countryCode: IT; stateProvince: Livorno; county: Campo nell'Elba; locality: Monte Perone; decimalLatitude: 42.761413; decimalLongitude: 10.197959; geodeticDatum: WGS84; coordinatePrecision: 0.0002; **Identification:** identifiedBy: E. Paggetti; **Event:** eventDate: 2011-06-19; **Record Level:** collectionCode: LFPC
- **Type status:** Other material. **Occurrence:** recordedBy: Leonardo Forbicioni; individualCount: 16; lifeStage: adult; occurrenceID: 4CE3D296-1239-50F6-8D12-C3FD1F7B0ABF; **Taxon:** scientificName: Anthaxia (Haplanthaxia) croesus (Villers, 1789); order: Coleoptera; family: Buprestidae; genus: Anthaxia; subgenus: Haplanthaxia; specificEpithet: croesus; scientificNameAuthorship: (Villers, 1789); **Location:** islandGroup: Tuscan Archipelago; island: Isola d'Elba; country: Italy; countryCode: IT; stateProvince: Livorno; county: Portoferraio; locality: Colle Reciso/Mulino a vento; decimalLatitude: 42.777066; decimalLongitude: 10.288258; geodeticDatum: WGS84; coordinatePrecision: 0.0002; **Identification:** identifiedBy: L. Forbicioni; **Event:** eventDate: 2013-06-17; **Record Level:** collectionCode: LFPC
- **Type status:** Other material. **Occurrence:** recordedBy: Leonardo Forbicioni; individualCount: 1; lifeStage: adult; occurrenceID: 0C5EC5CD-7085-56F5-B579-0B0C36A3ADC0; **Taxon:** scientificName: Anthaxia (Haplanthaxia) croesus (Villers, 1789); order: Coleoptera; family: Buprestidae; genus: Anthaxia; subgenus: Haplanthaxia; specificEpithet: croesus; scientificNameAuthorship: (Villers, 1789); **Location:** islandGroup: Tuscan Archipelago; island: Isola di Gorgona; country: Italy; countryCode: IT; stateProvince: Livorno; county: Gorgona; locality: Torre Vecchia/Ferro di Cavallo; decimalLatitude: 43.432613; decimalLongitude: 9.894604; geodeticDatum: WGS84; coordinatePrecision: 0.0002; **Identification:** identifiedBy: L. Forbicioni; **Event:** eventDate: 2015-06-29; **Record Level:** collectionCode: LFPC
- **Type status:** Other material. **Occurrence:** recordedBy: Leonardo Forbicioni; individualCount: 1; lifeStage: adult; occurrenceID: 7978E22B-D190-512B-947B-259D520701C7; **Taxon:** scientificName: Anthaxia (Haplanthaxia) croesus (Villers, 1789); order: Coleoptera; family: Buprestidae; genus: Anthaxia; subgenus: Haplanthaxia; specificEpithet: croesus; scientificNameAuthorship: (Villers, 1789); **Location:** islandGroup: Tuscan Archipelago; island: Isola di Capraia; country: Italy; countryCode: IT; stateProvince: Livorno; county: Capraia; locality: Vado dello Zenobito; decimalLatitude: 43.031616; decimalLongitude: 9.828856; geodeticDatum: WGS84; coordinatePrecision: 0.0002; **Identification:** identifiedBy: L. Forbicioni; **Event:** eventDate: 2016-05-21; **Record Level:** collectionCode: LFPC
- **Type status:** Other material. **Occurrence:** recordedBy: Leonardo Forbicioni; individualCount: 1; lifeStage: adult; occurrenceID: 7AF0683B-3801-5A5D-BD3D-233B4C85393A; **Taxon:** scientificName: Anthaxia (Haplanthaxia) croesus (Villers, 1789); order: Coleoptera; family: Buprestidae; genus: Anthaxia; subgenus: Haplanthaxia; specificEpithet: croesus; scientificNameAuthorship: (Villers, 1789); **Location:** islandGroup: Tuscan Archipelago; island: Isola del Giglio; country: Italy; countryCode: IT; stateProvince: Grosseto; county: Giglio Castello; locality: Calbugina; decimalLatitude: 42.382899; decimalLongitude: 10.891096; geodeticDatum: WGS84; coordinatePrecision: 0.0002; **Identification:** identifiedBy: L. Forbicioni; **Event:** eventDate: 2016-05-03; **Record Level:** collectionCode: LFPC
- **Type status:** Other material. **Occurrence:** recordedBy: Leonardo Forbicioni; individualCount: 1; lifeStage: adult; occurrenceID: ECD1A04B-C849-5E63-A871-8AFA84E2A570; **Taxon:** scientificName: Anthaxia (Haplanthaxia) croesus (Villers, 1789); order: Coleoptera; family: Buprestidae; genus: Anthaxia; subgenus: Haplanthaxia; specificEpithet: croesus; scientificNameAuthorship: (Villers, 1789); **Location:** islandGroup: Tuscan Archipelago; island: Isola d'Elba; country: Italy; countryCode: IT; stateProvince: Livorno; county: Campo nell'Elba; locality: Monte Tambone; decimalLatitude: 42.755946; decimalLongitude: 10.272602; geodeticDatum: WGS84; coordinatePrecision: 0.0002; **Identification:** identifiedBy: L. Forbicioni; **Event:** eventDate: 2017-05-28; **Record Level:** collectionCode: LFPC
- **Type status:** Other material. **Occurrence:** recordedBy: Leonardo Forbicioni; individualCount: 1; lifeStage: adult; occurrenceID: 408CFCA4-CDCA-5FDE-806F-1CF53A94D8C8; **Taxon:** scientificName: Anthaxia (Haplanthaxia) croesus (Villers, 1789); order: Coleoptera; family: Buprestidae; genus: Anthaxia; subgenus: Haplanthaxia; specificEpithet: croesus; scientificNameAuthorship: (Villers, 1789); **Location:** islandGroup: Tuscan Archipelago; island: Isola d'Elba; country: Italy; countryCode: IT; stateProvince: Livorno; county: Campo nell'Elba; locality: Monte Maolo; decimalLatitude: 42.773281; decimalLongitude: 10.187927; geodeticDatum: WGS84; coordinatePrecision: 0.0002; **Identification:** identifiedBy: L. Forbicioni; **Event:** eventDate: 2018-05-05; **Record Level:** collectionCode: LFPC
- **Type status:** Other material. **Occurrence:** recordedBy: Leonardo Forbicioni; individualCount: 1; lifeStage: adult; occurrenceID: DB7E5C93-4641-560D-89E3-438BC10EA8E1; **Taxon:** scientificName: Anthaxia (Haplanthaxia) croesus (Villers, 1789); order: Coleoptera; family: Buprestidae; genus: Anthaxia; subgenus: Haplanthaxia; specificEpithet: croesus; scientificNameAuthorship: (Villers, 1789); **Location:** islandGroup: Tuscan Archipelago; island: Isola d'Elba; country: Italy; countryCode: IT; stateProvince: Livorno; county: Porto Azzurro; locality: Buraccio; decimalLatitude: 42.780192; decimalLongitude: 10.352185; geodeticDatum: WGS84; coordinatePrecision: 0.0002; **Identification:** identifiedBy: L. Forbicioni; **Event:** eventDate: 2017-06-30; **Record Level:** collectionCode: LFPC
- **Type status:** Other material. **Occurrence:** recordedBy: Leonardo Forbicioni; individualCount: 1; lifeStage: adult; occurrenceID: 44A048C0-A164-57B8-BA85-FD2AE8EC95E7; **Taxon:** scientificName: Anthaxia (Haplanthaxia) croesus (Villers, 1789); order: Coleoptera; family: Buprestidae; genus: Anthaxia; subgenus: Haplanthaxia; specificEpithet: croesus; scientificNameAuthorship: (Villers, 1789); **Location:** islandGroup: Tuscan Archipelago; island: Isola d'Elba; country: Italy; countryCode: IT; stateProvince: Livorno; county: Campo nell'Elba; locality: Monte Perone; decimalLatitude: 42.775721; decimalLongitude: 10.193631; geodeticDatum: WGS84; coordinatePrecision: 0.0002; **Identification:** identifiedBy: L. Forbicioni; **Event:** eventDate: 2018-06-02; **Record Level:** collectionCode: LFPC
- **Type status:** Other material. **Occurrence:** recordedBy: Andrea Beltramini; individualCount: 2; lifeStage: adult; occurrenceID: 1076CA4F-F87E-5F69-A866-AF1524854C47; **Taxon:** scientificName: Anthaxia (Haplanthaxia) croesus (Villers, 1789); order: Coleoptera; family: Buprestidae; genus: Anthaxia; subgenus: Haplanthaxia; specificEpithet: croesus; scientificNameAuthorship: (Villers, 1789); **Location:** islandGroup: Tuscan Archipelago; island: Isola d'Elba; country: Italy; countryCode: IT; stateProvince: Livorno; county: Campo nell'Elba; locality: Monumento; decimalLatitude: 42.766383; decimalLongitude: 10.274056; geodeticDatum: WGS84; coordinatePrecision: 0.0002; **Identification:** identifiedBy: L. Forbicioni; **Event:** eventDate: 15-29/05/2023; **Record Level:** collectionCode: ABPC
- **Type status:** Other material. **Occurrence:** recordedBy: Andrea Beltramini; individualCount: 2; lifeStage: adult; occurrenceID: 28072CA0-2EA2-5943-8F1C-65458A26AF0D; **Taxon:** scientificName: Anthaxia (Haplanthaxia) croesus (Villers, 1789); order: Coleoptera; family: Buprestidae; genus: Anthaxia; subgenus: Haplanthaxia; specificEpithet: croesus; scientificNameAuthorship: (Villers, 1789); **Location:** islandGroup: Tuscan Archipelago; island: Isola d'Elba; country: Italy; countryCode: IT; stateProvince: Livorno; county: Campo nell'Elba; locality: Monumento; decimalLatitude: 42.766383; decimalLongitude: 10.274056; geodeticDatum: WGS84; coordinatePrecision: 0.0002; **Identification:** identifiedBy: L. Forbicioni; **Event:** eventDate: 15-29/05/2023; **Record Level:** collectionCode: ABPC

#### Anthaxia (Haplanthaxia) millefolii polychloros

Abeille de Perrin, 1894

73491CF9-F11D-5277-ACBD-BEACC6CC6209

##### Materials

- **Type status:** Other material. **Occurrence:** recordedBy: Giuliano Frangini; individualCount: 1; lifeStage: adult; occurrenceID: 0238A833-758E-5416-B8A2-7D0176C06BC9; **Taxon:** scientificName: Anthaxia (Haplanthaxia) millefolii polychloros Abeille de Perrin, 1900; order: Coleoptera; family: Buprestidae; genus: Anthaxia; subgenus: Haplanthaxia; specificEpithet: millefolii; infraspecificEpithet: polychloros; scientificNameAuthorship: Abeille de Perrin, 1900; **Location:** islandGroup: Tuscan Archipelago; island: Isola d'Elba; country: Italy; countryCode: IT; stateProvince: Livorno; county: Portoferraio; locality: Schiopparello/Le Prade; decimalLatitude: 42.796221; decimalLongitude: 10.345647; geodeticDatum: WGS84; coordinatePrecision: 0.0002; **Identification:** identifiedBy: E. Paggetti; **Event:** eventDate: 2011-06-29; **Record Level:** collectionCode: LFPC
- **Type status:** Other material. **Occurrence:** recordedBy: Leonardo Forbicioni; individualCount: 3; lifeStage: adult; occurrenceID: 51DF5866-7C19-5CBB-BABD-DDA99F1BC227; **Taxon:** scientificName: Anthaxia (Haplanthaxia) millefolii polychloros Abeille de Perrin, 1900; order: Coleoptera; family: Buprestidae; genus: Anthaxia; subgenus: Haplanthaxia; specificEpithet: millefolii; infraspecificEpithet: polychloros; scientificNameAuthorship: Abeille de Perrin, 1900; **Location:** islandGroup: Tuscan Archipelago; island: Isola d'Elba; country: Italy; countryCode: IT; stateProvince: Livorno; county: Capoliveri; locality: Pian di Mola; decimalLatitude: 42.758917; decimalLongitude: 10.368336; geodeticDatum: WGS84; coordinatePrecision: 0.0002; **Identification:** identifiedBy: E. Paggetti; **Event:** eventDate: 2011-06-02; **Record Level:** collectionCode: LFPC
- **Type status:** Other material. **Occurrence:** recordedBy: Leonardo Forbicioni; individualCount: 2; lifeStage: adult; occurrenceID: B0C73B50-18D3-5A3D-9EE1-812D4FD74CDF; **Taxon:** scientificName: Anthaxia (Haplanthaxia) millefolii polychloros Abeille de Perrin, 1900; order: Coleoptera; family: Buprestidae; genus: Anthaxia; subgenus: Haplanthaxia; specificEpithet: millefolii; infraspecificEpithet: polychloros; scientificNameAuthorship: Abeille de Perrin, 1900; **Location:** islandGroup: Tuscan Archipelago; island: Isola d'Elba; country: Italy; countryCode: IT; stateProvince: Livorno; county: Rio; municipality: Rio nell'Elba; locality: Volterraio; decimalLatitude: 42.803523; decimalLongitude: 10.390606; geodeticDatum: WGS84; coordinatePrecision: 0.0002; **Identification:** identifiedBy: E. Paggetti; **Event:** eventDate: 2011-06-15; **Record Level:** collectionCode: LFPC
- **Type status:** Other material. **Occurrence:** recordedBy: Leonardo Forbicioni; individualCount: 3; lifeStage: adult; occurrenceID: 4AE8A060-435D-506C-A091-0DAD15E99458; **Taxon:** scientificName: Anthaxia (Haplanthaxia) millefolii polychloros Abeille de Perrin, 1900; order: Coleoptera; family: Buprestidae; genus: Anthaxia; subgenus: Haplanthaxia; specificEpithet: millefolii; infraspecificEpithet: polychloros; scientificNameAuthorship: Abeille de Perrin, 1900; **Location:** islandGroup: Tuscan Archipelago; island: Isola d'Elba; country: Italy; countryCode: IT; stateProvince: Livorno; county: Porto Azzurro; locality: Buraccio/Lo Stipito; decimalLatitude: 42.761498; decimalLongitude: 10.365277; geodeticDatum: WGS84; coordinatePrecision: 0.0002; **Identification:** identifiedBy: E. Paggetti; **Event:** eventDate: 2011-06-30; **Record Level:** collectionCode: LFPC
- **Type status:** Other material. **Occurrence:** recordedBy: Leonardo Forbicioni; individualCount: 4; lifeStage: adult; occurrenceID: CA6053EF-F3AA-5E34-9DF0-4BA914060B66; **Taxon:** scientificName: Anthaxia (Haplanthaxia) millefolii polychloros Abeille de Perrin, 1900; order: Coleoptera; family: Buprestidae; genus: Anthaxia; subgenus: Haplanthaxia; specificEpithet: millefolii; infraspecificEpithet: polychloros; scientificNameAuthorship: Abeille de Perrin, 1900; **Location:** islandGroup: Tuscan Archipelago; island: Isola d'Elba; country: Italy; countryCode: IT; stateProvince: Livorno; county: Porto Azzurro; locality: Buraccio; decimalLatitude: 42.779990; decimalLongitude: 10.353760; geodeticDatum: WGS84; coordinatePrecision: 0.0002; **Identification:** identifiedBy: L. Forbicioni; **Event:** eventDate: 2022-07-25; **Record Level:** collectionCode: LFPC
- **Type status:** Other material. **Occurrence:** recordedBy: Leonardo Forbicioni; individualCount: 2; lifeStage: adult; occurrenceID: F1A519EE-7994-5BA5-B88A-A811471B9999; **Taxon:** scientificName: Anthaxia (Haplanthaxia) millefolii polychloros Abeille de Perrin, 1900; order: Coleoptera; family: Buprestidae; genus: Anthaxia; subgenus: Haplanthaxia; specificEpithet: millefolii; infraspecificEpithet: polychloros; scientificNameAuthorship: Abeille de Perrin, 1900; **Location:** islandGroup: Tuscan Archipelago; island: Isola d'Elba; country: Italy; countryCode: IT; stateProvince: Livorno; county: Rio; municipality: Rio nell'Elba; locality: Nisporto; decimalLatitude: 42.819974; decimalLongitude: 10.388064; geodeticDatum: WGS84; coordinatePrecision: 0.0002; **Identification:** identifiedBy: E. Paggetti; **Event:** eventDate: 2012-06-17; **Record Level:** collectionCode: LFPC
- **Type status:** Other material. **Occurrence:** recordedBy: Leonardo Forbicioni; individualCount: 1; lifeStage: adult; occurrenceID: 906792F3-58E5-5553-B084-FD571BCF4CDE; **Taxon:** scientificName: Anthaxia (Haplanthaxia) millefolii polychloros Abeille de Perrin, 1900; order: Coleoptera; family: Buprestidae; genus: Anthaxia; subgenus: Haplanthaxia; specificEpithet: millefolii; infraspecificEpithet: polychloros; scientificNameAuthorship: Abeille de Perrin, 1900; **Location:** islandGroup: Tuscan Archipelago; island: Isola d'Elba; country: Italy; countryCode: IT; stateProvince: Livorno; county: Campo nell'Elba; municipality: San Piero; locality: San Francesco; decimalLatitude: 42.767158; decimalLongitude: 10.193477; geodeticDatum: WGS84; coordinatePrecision: 0.0002; **Identification:** identifiedBy: L. Forbicioni; **Event:** eventDate: 2013-06-19; **Record Level:** collectionCode: LFPC
- **Type status:** Other material. **Occurrence:** recordedBy: Leonardo Forbicioni; individualCount: 2; lifeStage: adult; occurrenceID: B29A8AF7-F77A-51A3-848D-ECCA213239C7; **Taxon:** scientificName: Anthaxia (Haplanthaxia) millefolii polychloros Abeille de Perrin, 1900; order: Coleoptera; family: Buprestidae; genus: Anthaxia; subgenus: Haplanthaxia; specificEpithet: millefolii; infraspecificEpithet: polychloros; scientificNameAuthorship: Abeille de Perrin, 1900; **Location:** islandGroup: Tuscan Archipelago; island: Isola d'Elba; country: Italy; countryCode: IT; stateProvince: Livorno; county: Portoferraio; locality: Colle Reciso/Mulino a vento; decimalLatitude: 42.778955; decimalLongitude: 10.294144; geodeticDatum: WGS84; coordinatePrecision: 0.0002; **Identification:** identifiedBy: L. Forbicioni; **Event:** eventDate: 2013-07-02; **Record Level:** collectionCode: LFPC
- **Type status:** Other material. **Occurrence:** recordedBy: Leonardo Forbicioni; individualCount: 2; lifeStage: adult; occurrenceID: E6142448-81D3-54F3-BC6E-B44619DC594C; **Taxon:** scientificName: Anthaxia (Haplanthaxia) millefolii polychloros Abeille de Perrin, 1900; order: Coleoptera; family: Buprestidae; genus: Anthaxia; subgenus: Haplanthaxia; specificEpithet: millefolii; infraspecificEpithet: polychloros; scientificNameAuthorship: Abeille de Perrin, 1900; **Location:** islandGroup: Tuscan Archipelago; island: Isola d'Elba; country: Italy; countryCode: IT; stateProvince: Livorno; county: Portoferraio; locality: Val di Piano; decimalLatitude: 42.790612; decimalLongitude: 10.372348; geodeticDatum: WGS84; coordinatePrecision: 0.0002; **Identification:** identifiedBy: L. Forbicioni; **Event:** eventDate: 2011-06-05; **Record Level:** collectionCode: LFPC
- **Type status:** Other material. **Occurrence:** recordedBy: Leonardo Forbicioni; individualCount: 1; lifeStage: adult; occurrenceID: C73FB0A3-09B1-5726-91C4-1D829BEE6490; **Taxon:** scientificName: Anthaxia (Haplanthaxia) millefolii polychloros Abeille de Perrin, 1900; order: Coleoptera; family: Buprestidae; genus: Anthaxia; subgenus: Haplanthaxia; specificEpithet: millefolii; infraspecificEpithet: polychloros; scientificNameAuthorship: Abeille de Perrin, 1900; **Location:** islandGroup: Tuscan Archipelago; island: Isola d'Elba; country: Italy; countryCode: IT; stateProvince: Livorno; county: Portoferraio; locality: Monte Poppe; decimalLatitude: 42.797932; decimalLongitude: 10.276094; geodeticDatum: WGS84; coordinatePrecision: 0.0002; **Identification:** identifiedBy: L. Forbicioni; **Event:** eventDate: 2011-06-20; **Record Level:** collectionCode: LFPC
- **Type status:** Other material. **Occurrence:** recordedBy: Leonardo Forbicioni; individualCount: 1; lifeStage: adult; occurrenceID: C0F6C944-D828-59F1-A4C8-C40594B5C162; **Taxon:** scientificName: Anthaxia (Haplanthaxia) millefolii polychloros Abeille de Perrin, 1900; order: Coleoptera; family: Buprestidae; genus: Anthaxia; subgenus: Haplanthaxia; specificEpithet: millefolii; infraspecificEpithet: polychloros; scientificNameAuthorship: Abeille de Perrin, 1900; **Location:** islandGroup: Tuscan Archipelago; island: Isola d'Elba; country: Italy; countryCode: IT; stateProvince: Livorno; county: Portoferraio; locality: Le Prade; decimalLatitude: 42.797109; decimalLongitude: 10.350328; geodeticDatum: WGS84; coordinatePrecision: 0.0002; **Identification:** identifiedBy: L. Forbicioni; **Event:** eventDate: 2011-06-17; **Record Level:** collectionCode: LFPC
- **Type status:** Other material. **Occurrence:** recordedBy: Leonardo Forbicioni; individualCount: 1; lifeStage: adult; occurrenceID: 83C32A27-FCDD-5A70-8725-F7858FC85E95; **Taxon:** scientificName: Anthaxia (Haplanthaxia) millefolii polychloros Abeille de Perrin, 1900; order: Coleoptera; family: Buprestidae; genus: Anthaxia; subgenus: Haplanthaxia; specificEpithet: millefolii; infraspecificEpithet: polychloros; scientificNameAuthorship: Abeille de Perrin, 1900; **Location:** islandGroup: Tuscan Archipelago; island: Isola d'Elba; country: Italy; countryCode: IT; stateProvince: Livorno; county: Porto Azzurro; locality: Monserrato; decimalLatitude: 42.783877; decimalLongitude: 10.392538; geodeticDatum: WGS84; coordinatePrecision: 0.0002; **Identification:** identifiedBy: L. Forbicioni; **Event:** eventDate: 2014-06-06; **Record Level:** collectionCode: LFPC
- **Type status:** Other material. **Occurrence:** recordedBy: Leonardo Forbicioni; individualCount: 1; lifeStage: adult; occurrenceID: 9BFD61EF-CE21-56C6-AB4B-9844A0B5A9AE; **Taxon:** scientificName: Anthaxia (Haplanthaxia) millefolii polychloros Abeille de Perrin, 1900; order: Coleoptera; family: Buprestidae; genus: Anthaxia; subgenus: Haplanthaxia; specificEpithet: millefolii; infraspecificEpithet: polychloros; scientificNameAuthorship: Abeille de Perrin, 1900; **Location:** islandGroup: Tuscan Archipelago; island: Isola d'Elba; country: Italy; countryCode: IT; stateProvince: Livorno; county: Campo nell'Elba; municipality: San Piero; decimalLatitude: 42.751523; decimalLongitude: 10.206725; geodeticDatum: WGS84; coordinatePrecision: 0.0002; **Identification:** identifiedBy: L. Forbicioni; **Event:** eventDate: 2012-06-10; **Record Level:** collectionCode: LFPC
- **Type status:** Other material. **Occurrence:** recordedBy: Leonardo Forbicioni; individualCount: 4; lifeStage: adult; occurrenceID: 93461880-74B5-5F15-9A18-881F701BB696; **Taxon:** scientificName: Anthaxia (Haplanthaxia) millefolii polychloros Abeille de Perrin, 1900; order: Coleoptera; family: Buprestidae; genus: Anthaxia; subgenus: Haplanthaxia; specificEpithet: millefolii; infraspecificEpithet: polychloros; scientificNameAuthorship: Abeille de Perrin, 1900; **Location:** islandGroup: Tuscan Archipelago; island: Isola d'Elba; country: Italy; countryCode: IT; stateProvince: Livorno; county: Campo nell'Elba; municipality: Sant'Ilario; decimalLatitude: 42.763056; decimalLongitude: 10.224167; geodeticDatum: WGS84; coordinatePrecision: 0.0002; **Identification:** identifiedBy: L. Forbicioni; **Event:** eventDate: 2014-06-22; **Record Level:** collectionCode: LFPC
- **Type status:** Other material. **Occurrence:** recordedBy: Leonardo Forbicioni; individualCount: 1; lifeStage: adult; occurrenceID: C4F699B2-171A-56B5-B29C-4B0B7F8378EB; **Taxon:** scientificName: Anthaxia (Haplanthaxia) millefolii polychloros Abeille de Perrin, 1900; order: Coleoptera; family: Buprestidae; genus: Anthaxia; subgenus: Haplanthaxia; specificEpithet: millefolii; infraspecificEpithet: polychloros; scientificNameAuthorship: Abeille de Perrin, 1900; **Location:** islandGroup: Tuscan Archipelago; island: Isola d'Elba; country: Italy; countryCode: IT; stateProvince: Livorno; county: Campo nell'Elba; locality: Monte Tambone; decimalLatitude: 42.756270; decimalLongitude: 10.272057; geodeticDatum: WGS84; coordinatePrecision: 0.0002; **Identification:** identifiedBy: L. Forbicioni; **Event:** eventDate: 2022-06-08; **Record Level:** collectionCode: LFPC
- **Type status:** Other material. **Occurrence:** recordedBy: Leonardo Forbicioni; individualCount: 2; lifeStage: adult; occurrenceID: 293436C7-C996-533F-8215-804E029D71D0; **Taxon:** scientificName: Anthaxia (Haplanthaxia) millefolii polychloros Abeille de Perrin, 1900; order: Coleoptera; family: Buprestidae; genus: Anthaxia; subgenus: Haplanthaxia; specificEpithet: millefolii; infraspecificEpithet: polychloros; scientificNameAuthorship: Abeille de Perrin, 1900; **Location:** islandGroup: Tuscan Archipelago; island: Isola d'Elba; country: Italy; countryCode: IT; stateProvince: Livorno; county: Portoferraio; locality: Acquabona; decimalLatitude: 42.786976; decimalLongitude: 10.346688; geodeticDatum: WGS84; coordinatePrecision: 0.0002; **Identification:** identifiedBy: G. Curletti; **Event:** eventDate: 2022-06-15; **Record Level:** collectionCode: LFPC
- **Type status:** Other material. **Occurrence:** recordedBy: Leonardo Forbicioni; individualCount: 1; lifeStage: adult; occurrenceID: D26F84E8-4CD4-5252-86FA-40E74DB463ED; **Taxon:** scientificName: Anthaxia (Haplanthaxia) millefolii polychloros Abeille de Perrin, 1900; order: Coleoptera; family: Buprestidae; genus: Anthaxia; subgenus: Haplanthaxia; specificEpithet: millefolii; infraspecificEpithet: polychloros; scientificNameAuthorship: Abeille de Perrin, 1900; **Location:** islandGroup: Tuscan Archipelago; island: Isola d'Elba; country: Italy; countryCode: IT; stateProvince: Livorno; county: Campo nell'Elba; locality: Bonalaccia; decimalLatitude: 42.756865; decimalLongitude: 10.248018; geodeticDatum: WGS84; coordinatePrecision: 0.0002; **Identification:** identifiedBy: G. Curletti; **Event:** eventDate: 2016-06-06; **Record Level:** collectionCode: LFPC
- **Type status:** Other material. **Occurrence:** recordedBy: Leonardo Forbicioni; individualCount: 1; lifeStage: adult; occurrenceID: 0C749DDB-02C1-522B-AD95-2C4D7A69E271; **Taxon:** scientificName: Anthaxia (Haplanthaxia) millefolii polychloros Abeille de Perrin, 1900; order: Coleoptera; family: Buprestidae; genus: Anthaxia; subgenus: Haplanthaxia; specificEpithet: millefolii; infraspecificEpithet: polychloros; scientificNameAuthorship: Abeille de Perrin, 1900; **Location:** islandGroup: Tuscan Archipelago; island: Isola d'Elba; country: Italy; countryCode: IT; stateProvince: Livorno; county: Campo nell'Elba; municipality: Seccheto; locality: Vallebuia; decimalLatitude: 42.749455; decimalLongitude: 10.168182; geodeticDatum: WGS84; coordinatePrecision: 0.0002; **Identification:** identifiedBy: G. Curletti; **Event:** eventDate: 2018-05-27; **Record Level:** collectionCode: LFPC
- **Type status:** Other material. **Occurrence:** recordedBy: Leonardo Forbicioni; individualCount: 1; lifeStage: adult; occurrenceID: 16671623-1C03-57B5-AB01-5E3552F6D51B; **Taxon:** scientificName: Anthaxia (Haplanthaxia) millefolii polychloros Abeille de Perrin, 1900; order: Coleoptera; family: Buprestidae; genus: Anthaxia; subgenus: Haplanthaxia; specificEpithet: millefolii; infraspecificEpithet: polychloros; scientificNameAuthorship: Abeille de Perrin, 1900; **Location:** islandGroup: Tuscan Archipelago; island: Isola d'Elba; country: Italy; countryCode: IT; stateProvince: Livorno; county: Porto Azzurro; locality: Buraccio; decimalLatitude: 42.780492; decimalLongitude: 10.351755; geodeticDatum: WGS84; coordinatePrecision: 0.0002; **Identification:** identifiedBy: L. Forbicioni; **Event:** eventDate: 2022-07-25; **Record Level:** collectionCode: LFPC
- **Type status:** Other material. **Occurrence:** recordedBy: Leonardo Forbicioni; individualCount: 1; lifeStage: adult; occurrenceID: 8A17B794-6728-5AB7-8C5B-7C791BE54ED4; **Taxon:** scientificName: Anthaxia (Haplanthaxia) millefolii polychloros Abeille de Perrin, 1900; order: Coleoptera; family: Buprestidae; genus: Anthaxia; subgenus: Haplanthaxia; specificEpithet: millefolii; infraspecificEpithet: polychloros; scientificNameAuthorship: Abeille de Perrin, 1900; **Location:** islandGroup: Tuscan Archipelago; island: Isola d'Elba; country: Italy; countryCode: IT; stateProvince: Livorno; county: Porto Azzurro; locality: Buraccio; decimalLatitude: 42.780244; decimalLongitude: 10.355640; geodeticDatum: WGS84; coordinatePrecision: 0.0002; **Identification:** identifiedBy: G. Curletti; **Event:** eventDate: 2020-06-17; **Record Level:** collectionCode: LFPC
- **Type status:** Other material. **Occurrence:** recordedBy: Leonardo Forbicioni; individualCount: 1; lifeStage: adult; occurrenceID: 816B32FF-2531-5597-9DC6-85A78025D10C; **Taxon:** scientificName: Anthaxia (Haplanthaxia) millefolii polychloros Abeille de Perrin, 1900; order: Coleoptera; family: Buprestidae; genus: Anthaxia; subgenus: Haplanthaxia; specificEpithet: millefolii; infraspecificEpithet: polychloros; scientificNameAuthorship: Abeille de Perrin, 1900; **Location:** islandGroup: Tuscan Archipelago; island: Isola d'Elba; country: Italy; countryCode: IT; stateProvince: Livorno; county: Capoliveri; locality: Pian di Mola; decimalLatitude: 42.759619; decimalLongitude: 10.365966; geodeticDatum: WGS84; coordinatePrecision: 0.0002; **Identification:** identifiedBy: L. Forbicioni; **Event:** eventDate: 2011-05-28; **Record Level:** collectionCode: LFPC
- **Type status:** Other material. **Occurrence:** recordedBy: Marco Huang; individualCount: 1; lifeStage: adult; occurrenceID: 42E86930-0BCF-5D37-81EB-A0DB80BEF1B4; **Taxon:** scientificName: Anthaxia (Haplanthaxia) millefolii polychloros Abeille de Perrin, 1900; order: Coleoptera; family: Buprestidae; genus: Anthaxia; subgenus: Haplanthaxia; specificEpithet: millefolii; infraspecificEpithet: polychloros; scientificNameAuthorship: Abeille de Perrin, 1900; **Location:** islandGroup: Tuscan Archipelago; island: Isola d'Elba; country: Italy; countryCode: IT; stateProvince: Livorno; county: Campo nell'Elba; municipality: San Piero; decimalLatitude: 42.751607; decimalLongitude: 10.200397; geodeticDatum: WGS84; coordinatePrecision: 0.008; **Identification:** identifiedBy: L. Forbicioni; **Event:** eventDate: 2022-06-11; **Record Level:** source: https://www.inaturalist.org/observations/124413324
- **Type status:** Other material. **Occurrence:** individualCount: 1; lifeStage: adult; occurrenceID: 991D0683-FE7C-5FA2-AF53-987149DE62BD; **Taxon:** scientificName: Anthaxia (Haplanthaxia) millefolii polychloros Abeille de Perrin, 1900; order: Coleoptera; family: Buprestidae; genus: Anthaxia; subgenus: Haplanthaxia; specificEpithet: millefolii; infraspecificEpithet: polychloros; scientificNameAuthorship: Abeille de Perrin, 1900; **Location:** islandGroup: Tuscan Archipelago; island: Isola d'Elba; country: Italy; countryCode: IT; stateProvince: Livorno; county: Porto Azzurro; **Identification:** identifiedBy: G. Curletti; **Record Level:** source: Curletti G. (1994) I Buprestidi d’Italia. Catalogo geonemico, sinonimico, bibliografico, biologico. Monografie di Natura Bresciana, Ed. Vannini, Brescia, 19.
- **Type status:** Other material. **Occurrence:** individualCount: 1; lifeStage: adult; occurrenceID: 6096AF15-E777-5F4F-B82E-5EC3EA8818A7; **Taxon:** scientificName: Anthaxia (Haplanthaxia) millefolii polychloros Abeille de Perrin, 1900; order: Coleoptera; family: Buprestidae; genus: Anthaxia; subgenus: Haplanthaxia; specificEpithet: millefolii; infraspecificEpithet: polychloros; scientificNameAuthorship: Abeille de Perrin, 1900; **Location:** islandGroup: Tuscan Archipelago; island: Isola d'Elba; country: Italy; countryCode: IT; stateProvince: Livorno; county: Rio; municipality: Rio Marina; locality: Ortano; **Identification:** identifiedBy: G. Curletti; **Record Level:** source: Curletti G. (1994) I Buprestidi d’Italia. Catalogo geonemico, sinonimico, bibliografico, biologico. Monografie di Natura Bresciana, Ed. Vannini, Brescia, 19.
- **Type status:** Other material. **Occurrence:** individualCount: 1; lifeStage: adult; occurrenceID: 704A6615-9E97-587B-8E4B-AEAC2875DE4F; **Taxon:** scientificName: Anthaxia (Haplanthaxia) millefolii polychloros Abeille de Perrin, 1900; order: Coleoptera; family: Buprestidae; genus: Anthaxia; subgenus: Haplanthaxia; specificEpithet: millefolii; infraspecificEpithet: polychloros; scientificNameAuthorship: Abeille de Perrin, 1900; **Location:** islandGroup: Tuscan Archipelago; island: Isola del Giglio; country: Italy; countryCode: GR; stateProvince: Grosseto; **Identification:** identifiedBy: G. Curletti; **Record Level:** source: Curletti G. (1994) I Buprestidi d’Italia. Catalogo geonemico, sinonimico, bibliografico, biologico. Monografie di Natura Bresciana, Ed. Vannini, Brescia, 19.

##### Conservation status

LC

##### Distribution

Recorded for the Tuscan Archipelago (Isola d'Elba and Isola del Giglio) by Curletti (1994).

#### Anthaxia (Haplanthaxia) umbellatarum umbellatarum

(Fabricius, 1787)

2CBC635D-0562-5CFF-A158-DA0CA9E0B47F

##### Materials

- **Type status:** Other material. **Occurrence:** recordedBy: Leonardo Forbicioni; individualCount: 2; lifeStage: adult; occurrenceID: 1DF8688F-E2BF-5A45-BBE9-57296EF6E7C3; **Taxon:** scientificName: Anthaxia (Haplanthaxia) umbellatarum umbellatarum (Fabricius, 1787); order: Coleoptera; family: Buprestidae; genus: Anthaxia; subgenus: Haplanthaxia; specificEpithet: umbellatarum; infraspecificEpithet: umbellatarum; scientificNameAuthorship: (Fabricius, 1787); **Location:** islandGroup: Tuscan Archipelago; island: Isola d'Elba; country: Italy; countryCode: IT; stateProvince: Livorno; county: Capoliveri; locality: Pian di Mola; decimalLatitude: 42.759619; decimalLongitude: 10.365966; geodeticDatum: WGS84; coordinatePrecision: 0.0002; **Identification:** identifiedBy: L. Forbicioni; **Event:** eventDate: 2011-05-28; **Record Level:** collectionCode: LFPC
- **Type status:** Other material. **Occurrence:** recordedBy: Leonardo Forbicioni; individualCount: 5; lifeStage: adult; occurrenceID: 84989E2A-0FDA-50E1-A1EE-FB3F0E872E27; **Taxon:** scientificName: Anthaxia (Haplanthaxia) umbellatarum umbellatarum (Fabricius, 1787); order: Coleoptera; family: Buprestidae; genus: Anthaxia; subgenus: Haplanthaxia; specificEpithet: umbellatarum; infraspecificEpithet: umbellatarum; scientificNameAuthorship: (Fabricius, 1787); **Location:** islandGroup: Tuscan Archipelago; island: Isola d'Elba; country: Italy; countryCode: IT; stateProvince: Livorno; county: Portoferraio; locality: Val di Piano; decimalLatitude: 42.790903; decimalLongitude: 10.371978; geodeticDatum: WGS84; coordinatePrecision: 0.0002; **Identification:** identifiedBy: E. Paggetti; **Event:** eventDate: 2011-06-08; **Record Level:** collectionCode: LFPC
- **Type status:** Other material. **Occurrence:** recordedBy: Leonardo Forbicioni; individualCount: 1; lifeStage: adult; occurrenceID: C6F4EF4D-BD17-5DAC-9679-6648B9F5A4A7; **Taxon:** scientificName: Anthaxia (Haplanthaxia) umbellatarum umbellatarum (Fabricius, 1787); order: Coleoptera; family: Buprestidae; genus: Anthaxia; subgenus: Haplanthaxia; specificEpithet: umbellatarum; infraspecificEpithet: umbellatarum; scientificNameAuthorship: (Fabricius, 1787); **Location:** islandGroup: Tuscan Archipelago; island: Isola d'Elba; country: Italy; countryCode: IT; stateProvince: Livorno; county: Capoliveri; locality: Norsi; decimalLatitude: 42.769582; decimalLongitude: 10.346034; geodeticDatum: WGS84; coordinatePrecision: 0.0002; **Identification:** identifiedBy: L. Forbicioni; **Event:** eventDate: 2013-06-03; **Record Level:** collectionCode: LFPC
- **Type status:** Other material. **Occurrence:** recordedBy: Leonardo Forbicioni; individualCount: 1; lifeStage: adult; occurrenceID: 44BFE82A-3AB9-5666-B641-123D3163B55A; **Taxon:** scientificName: Anthaxia (Haplanthaxia) umbellatarum umbellatarum (Fabricius, 1787); order: Coleoptera; family: Buprestidae; genus: Anthaxia; subgenus: Haplanthaxia; specificEpithet: umbellatarum; infraspecificEpithet: umbellatarum; scientificNameAuthorship: (Fabricius, 1787); **Location:** islandGroup: Tuscan Archipelago; island: Isola d'Elba; country: Italy; countryCode: IT; stateProvince: Livorno; county: Capoliveri; locality: Norsi; decimalLatitude: 42.769582; decimalLongitude: 10.346034; geodeticDatum: WGS84; coordinatePrecision: 0.0002; **Identification:** identifiedBy: L. Forbicioni; **Event:** eventDate: 2013-06-09; **Record Level:** collectionCode: LFPC
- **Type status:** Other material. **Occurrence:** recordedBy: Leonardo Forbicioni; individualCount: 1; lifeStage: adult; occurrenceID: 71EC050D-B416-5B6D-BB50-EBE1FA88E496; **Taxon:** scientificName: Anthaxia (Haplanthaxia) umbellatarum umbellatarum (Fabricius, 1787); order: Coleoptera; family: Buprestidae; genus: Anthaxia; subgenus: Haplanthaxia; specificEpithet: umbellatarum; infraspecificEpithet: umbellatarum; scientificNameAuthorship: (Fabricius, 1787); **Location:** islandGroup: Tuscan Archipelago; island: Isola d'Elba; country: Italy; countryCode: IT; stateProvince: Livorno; county: Capoliveri; locality: Norsi; decimalLatitude: 42.768054; decimalLongitude: 10.345058; geodeticDatum: WGS84; coordinatePrecision: 0.0002; **Identification:** identifiedBy: E. Paggetti; **Event:** eventDate: 2011-06-10; **Record Level:** collectionCode: LFPC
- **Type status:** Other material. **Occurrence:** recordedBy: Leonardo Forbicioni; individualCount: 1; lifeStage: adult; occurrenceID: C855FA40-E2A6-55BD-AFE4-3F859A6CC654; **Taxon:** scientificName: Anthaxia (Haplanthaxia) umbellatarum umbellatarum (Fabricius, 1787); order: Coleoptera; family: Buprestidae; genus: Anthaxia; subgenus: Haplanthaxia; specificEpithet: umbellatarum; infraspecificEpithet: umbellatarum; scientificNameAuthorship: (Fabricius, 1787); **Location:** islandGroup: Tuscan Archipelago; island: Isola d'Elba; country: Italy; countryCode: IT; stateProvince: Livorno; county: Porto Azzurro; locality: Buraccio/Lo Stipito; decimalLatitude: 42.761498; decimalLongitude: 10.365277; geodeticDatum: WGS84; coordinatePrecision: 0.0002; **Identification:** identifiedBy: E. Paggetti; **Event:** eventDate: 2011-06-30; **Record Level:** collectionCode: LFPC
- **Type status:** Other material. **Occurrence:** recordedBy: Leonardo Forbicioni; individualCount: 1; lifeStage: adult; occurrenceID: C817D941-A3E7-55BC-87F1-B32AAD07A3B6; **Taxon:** scientificName: Anthaxia (Haplanthaxia) umbellatarum umbellatarum (Fabricius, 1787); order: Coleoptera; family: Buprestidae; genus: Anthaxia; subgenus: Haplanthaxia; specificEpithet: umbellatarum; infraspecificEpithet: umbellatarum; scientificNameAuthorship: (Fabricius, 1787); **Location:** islandGroup: Tuscan Archipelago; island: Isola d'Elba; country: Italy; countryCode: IT; stateProvince: Livorno; county: Portoferraio; locality: Buca di Bomba; decimalLatitude: 42.780677; decimalLongitude: 10.271738; geodeticDatum: WGS84; coordinatePrecision: 0.0002; **Identification:** identifiedBy: E. Paggetti; **Event:** eventDate: 2011-07-17; **Record Level:** collectionCode: LFPC
- **Type status:** Other material. **Occurrence:** recordedBy: Leonardo Forbicioni; individualCount: 1; lifeStage: adult; occurrenceID: 989A4E0B-D01E-5208-9787-82373C7F40E4; **Taxon:** scientificName: Anthaxia (Haplanthaxia) umbellatarum umbellatarum (Fabricius, 1787); order: Coleoptera; family: Buprestidae; genus: Anthaxia; subgenus: Haplanthaxia; specificEpithet: umbellatarum; infraspecificEpithet: umbellatarum; scientificNameAuthorship: (Fabricius, 1787); **Location:** islandGroup: Tuscan Archipelago; island: Isola d'Elba; country: Italy; countryCode: IT; stateProvince: Livorno; county: Portoferraio; locality: San Martino; decimalLatitude: 42.783042; decimalLongitude: 10.281678; geodeticDatum: WGS84; coordinatePrecision: 0.0002; **Identification:** identifiedBy: E. Paggetti; **Event:** eventDate: 2012-06-03; **Record Level:** collectionCode: LFPC
- **Type status:** Other material. **Occurrence:** recordedBy: Leonardo Forbicioni; individualCount: 1; lifeStage: adult; occurrenceID: CE193256-418A-5D23-B4C3-D8BA006CFF29; **Taxon:** scientificName: Anthaxia (Haplanthaxia) umbellatarum umbellatarum (Fabricius, 1787); order: Coleoptera; family: Buprestidae; genus: Anthaxia; subgenus: Haplanthaxia; specificEpithet: umbellatarum; infraspecificEpithet: umbellatarum; scientificNameAuthorship: (Fabricius, 1787); **Location:** islandGroup: Tuscan Archipelago; island: Isola d'Elba; country: Italy; countryCode: IT; stateProvince: Livorno; county: Rio; municipality: Rio nell'Elba; locality: Nisporto; decimalLatitude: 42.819974; decimalLongitude: 10.388064; geodeticDatum: WGS84; coordinatePrecision: 0.0002; **Identification:** identifiedBy: E. Paggetti; **Event:** eventDate: 2012-06-17; **Record Level:** collectionCode: LFPC
- **Type status:** Other material. **Occurrence:** recordedBy: Leonardo Forbicioni; individualCount: 1; lifeStage: adult; occurrenceID: E3539A8A-F217-5CD5-8F75-6783B796B74F; **Taxon:** scientificName: Anthaxia (Haplanthaxia) umbellatarum umbellatarum (Fabricius, 1787); order: Coleoptera; family: Buprestidae; genus: Anthaxia; subgenus: Haplanthaxia; specificEpithet: umbellatarum; infraspecificEpithet: umbellatarum; scientificNameAuthorship: (Fabricius, 1787); **Location:** islandGroup: Tuscan Archipelago; island: Isola d'Elba; country: Italy; countryCode: IT; stateProvince: Livorno; county: Porto Azzurro; locality: Mola-Spiaggia; decimalLatitude: 42.759699; decimalLongitude: 10.385523; geodeticDatum: WGS84; coordinatePrecision: 0.0002; **Identification:** identifiedBy: L. Forbicioni; **Event:** eventDate: 2014-05-10; **Record Level:** collectionCode: LFPC
- **Type status:** Other material. **Occurrence:** recordedBy: Leonardo Forbicioni; individualCount: 1; lifeStage: adult; occurrenceID: 954C1B7E-2FB4-5199-AE68-20D29C3F2BD1; **Taxon:** scientificName: Anthaxia (Haplanthaxia) umbellatarum umbellatarum (Fabricius, 1787); order: Coleoptera; family: Buprestidae; genus: Anthaxia; subgenus: Haplanthaxia; specificEpithet: umbellatarum; infraspecificEpithet: umbellatarum; scientificNameAuthorship: (Fabricius, 1787); **Location:** islandGroup: Tuscan Archipelago; island: Isola d'Elba; country: Italy; countryCode: IT; stateProvince: Livorno; county: Campo nell'Elba; municipality: Sant'Ilario; decimalLatitude: 42.763297; decimalLongitude: 10.224256; geodeticDatum: WGS84; coordinatePrecision: 0.0002; **Identification:** identifiedBy: L. Forbicioni; **Event:** eventDate: 2014-07-11; **Record Level:** collectionCode: LFPC
- **Type status:** Other material. **Occurrence:** recordedBy: Leonardo Forbicioni; individualCount: 1; lifeStage: adult; occurrenceID: E9D66D34-FBD4-5992-ADDF-08493D565347; **Taxon:** scientificName: Anthaxia (Haplanthaxia) umbellatarum umbellatarum (Fabricius, 1787); order: Coleoptera; family: Buprestidae; genus: Anthaxia; subgenus: Haplanthaxia; specificEpithet: umbellatarum; infraspecificEpithet: umbellatarum; scientificNameAuthorship: (Fabricius, 1787); **Location:** islandGroup: Tuscan Archipelago; island: Isola d'Elba; country: Italy; countryCode: IT; stateProvince: Livorno; county: Porto Azzurro; locality: Buraccio; decimalLatitude: 42.780000; decimalLongitude: 10.354722; geodeticDatum: WGS84; coordinatePrecision: 0.0002; **Identification:** identifiedBy: G. Curletti; **Event:** eventDate: 2017-06-30; **Record Level:** collectionCode: LFPC
- **Type status:** Other material. **Occurrence:** recordedBy: Leonardo Forbicioni; individualCount: 1; lifeStage: adult; occurrenceID: 0FF71D3C-D55F-5316-AF07-D2FA81C42B0E; **Taxon:** scientificName: Anthaxia (Haplanthaxia) umbellatarum umbellatarum (Fabricius, 1787); order: Coleoptera; family: Buprestidae; genus: Anthaxia; subgenus: Haplanthaxia; specificEpithet: umbellatarum; infraspecificEpithet: umbellatarum; scientificNameAuthorship: (Fabricius, 1787); **Location:** islandGroup: Tuscan Archipelago; island: Isola d'Elba; country: Italy; countryCode: IT; stateProvince: Livorno; county: Portoferraio; locality: Colle Reciso/Mulino a vento; decimalLatitude: 42.779005; decimalLongitude: 10.293395; geodeticDatum: WGS84; coordinatePrecision: 0.0002; **Identification:** identifiedBy: G. Curletti; **Event:** eventDate: 2020-08-09; **Record Level:** collectionCode: LFPC
- **Type status:** Other material. **Occurrence:** individualCount: 1; lifeStage: adult; occurrenceID: 21516C2F-CF5F-5833-ADED-F727FEDBB50B; **Taxon:** scientificName: Anthaxia (Haplanthaxia) umbellatarum umbellatarum (Fabricius, 1787); order: Coleoptera; family: Buprestidae; genus: Anthaxia; subgenus: Haplanthaxia; specificEpithet: umbellatarum; infraspecificEpithet: umbellatarum; scientificNameAuthorship: (Fabricius, 1787); **Location:** islandGroup: Tuscan Archipelago; island: Isola d'Elba; country: Italy; countryCode: IT; stateProvince: Livorno; county: Portoferraio; **Record Level:** source: Sub Anthaxia inculta Germr, 1817 - Razzauti A (1921) Contributi alla conoscenza faunistica delle isole toscane III. Coleotteri delle Isole d’Elba, di Capraia e di Gorgona Atti della Società toscana di Scienze naturali residente in Pisa. 33. Memorie, Pisa, 96-122 pp.. https://www.biodiversitylibrary.org/page/35335669
- **Type status:** Other material. **Occurrence:** individualCount: 1; lifeStage: adult; occurrenceID: CD67C87C-B1DE-51C7-A1DD-C68F2E66CCCF; **Taxon:** scientificName: Anthaxia (Haplanthaxia) umbellatarum umbellatarum (Fabricius, 1787); order: Coleoptera; family: Buprestidae; genus: Anthaxia; subgenus: Haplanthaxia; specificEpithet: umbellatarum; infraspecificEpithet: umbellatarum; scientificNameAuthorship: (Fabricius, 1787); **Location:** islandGroup: Tuscan Archipelago; island: Isola d'Elba; country: Italy; countryCode: IT; stateProvince: Livorno; county: Portoferraio; **Record Level:** source: Holdhaus K. (1923) Elenco dei Coleotteri dell’isola d’Elba con studi sul problema della Tirrenide. Memorie della Società entomologica italiana 2: 77‑175. Sub Anthaxia inculta Germr, 1817
- **Type status:** Other material. **Occurrence:** individualCount: 1; lifeStage: adult; occurrenceID: 59F8F027-1B4E-5024-B8CA-BE1A775F1F8F; **Taxon:** scientificName: Anthaxia (Haplanthaxia) umbellatarum umbellatarum (Fabricius, 1787); order: Coleoptera; family: Buprestidae; genus: Anthaxia; subgenus: Haplanthaxia; specificEpithet: umbellatarum; infraspecificEpithet: umbellatarum; scientificNameAuthorship: (Fabricius, 1787); **Location:** islandGroup: Tuscan Archipelago; island: Isola d'Elba; country: Italy; countryCode: IT; stateProvince: Livorno; **Identification:** identifiedBy: G. Curletti; **Record Level:** source: Curletti G. (1994) I Buprestidi d’Italia. Catalogo geonemico, sinonimico, bibliografico, biologico. Monografie di Natura Bresciana, Ed. Vannini, Brescia, 19.

##### Conservation status

LC

##### Distribution

Recorded for the Tuscan Archipelago (Elba) by Holdhaus 1923 and Curletti 1994.

#### Anthaxia (Melanthaxia) nigritula nigritula

Ratzeburg, 1837

10965DD2-12E9-5A74-AFCB-376D5B617113

##### Materials

- **Type status:** Other material. **Occurrence:** recordedBy: Leonardo Forbicioni; individualCount: 5; lifeStage: adult; occurrenceID: A93387CF-453D-5A0A-BCDB-1F94BC020F6D; **Taxon:** scientificName: Anthaxia (Melanthaxia) nigritula nigritula Ratzeburg, 1837; order: Coleoptera; family: Buprestidae; genus: Anthaxia; subgenus: Melanthaxia; specificEpithet: nigritula; infraspecificEpithet: nigritula; scientificNameAuthorship: Ratzeburg, 1837; **Location:** islandGroup: Tuscan Archipelago; island: Isola d'Elba; country: Italy; countryCode: IT; stateProvince: Livorno; county: Campo nell'Elba; locality: Monte Perone; decimalLatitude: 42.776281; decimalLongitude: 10.195317; geodeticDatum: WGS84; coordinatePrecision: 0.0002; **Identification:** identifiedBy: L. Forbicioni; **Event:** eventDate: 2013-05-07; **Record Level:** collectionCode: LFPC
- **Type status:** Other material. **Occurrence:** recordedBy: Leonardo Forbicioni; individualCount: 1; lifeStage: adult; occurrenceID: CDE081C1-AA75-5CCF-8EEE-85E104087E59; **Taxon:** scientificName: Anthaxia (Melanthaxia) nigritula nigritula Ratzeburg, 1837; order: Coleoptera; family: Buprestidae; genus: Anthaxia; subgenus: Melanthaxia; specificEpithet: nigritula; infraspecificEpithet: nigritula; scientificNameAuthorship: Ratzeburg, 1837; **Location:** islandGroup: Tuscan Archipelago; island: Isola d'Elba; country: Italy; countryCode: IT; stateProvince: Livorno; county: Capoliveri; locality: Monte Calamita; decimalLatitude: 42.734475; decimalLongitude: 10.390292; geodeticDatum: WGS84; coordinatePrecision: 0.0002; **Identification:** identifiedBy: L. Forbicioni; **Event:** eventDate: 2015-04-24; **Record Level:** collectionCode: LFPC
- **Type status:** Other material. **Occurrence:** individualCount: 1; lifeStage: adult; occurrenceID: 26A6F0E9-617F-5473-8B54-FC1C96E15DB3; **Taxon:** scientificName: Anthaxia (Melanthaxia) nigritula nigritula Ratzeburg, 1837; order: Coleoptera; family: Buprestidae; genus: Anthaxia; subgenus: Melanthaxia; specificEpithet: nigritula; infraspecificEpithet: nigritula; scientificNameAuthorship: Ratzeburg, 1837; **Location:** islandGroup: Tuscan Archipelago; island: Isola d'Elba; country: Italy; countryCode: IT; stateProvince: Livorno; **Identification:** identifiedBy: G. Curletti; **Record Level:** source: Curletti G. (1994) I Buprestidi d’Italia. Catalogo geonemico, sinonimico, bibliografico, biologico. Monografie di Natura Bresciana, Ed. Vannini, Brescia, 19.

##### Conservation status

LC

##### Distribution

Recorded for the Tuscan Archipelago (Isola d'Elba) by Curletti (1994).

#### Buprestini

074D77C5-BD53-5EF7-A3DD-D0ACA6DB7D0A

#### Buprestis (Ancylocheira) novemmaculata novemmaculata

Linnaeus, 1767

185DE305-19C6-5987-9235-6D401527EA5F

##### Materials

- **Type status:** Other material. **Occurrence:** recordedBy: Leonardo Forbicioni; individualCount: 1; lifeStage: adult; occurrenceID: 2DE0166F-26B7-598A-9B68-E8F8456DBA12; **Taxon:** scientificName: Buprestis (Ancylocheira) novemmaculata novemmaculata Linnaeus, 1767; order: Coleoptera; family: Buprestidae; genus: Buprestis; subgenus: Ancylocheira; specificEpithet: novemmaculata; infraspecificEpithet: novemmaculata; scientificNameAuthorship: Linnaeus, 1767; **Location:** islandGroup: Tuscan Archipelago; island: Isola d'Elba; country: Italy; countryCode: IT; stateProvince: Livorno; county: Portoferraio; locality: Colle Reciso/Mulino a vento; decimalLatitude: 42.777678; decimalLongitude: 10.289680; geodeticDatum: WGS84; coordinatePrecision: 0.0002; **Identification:** identifiedBy: E. Paggetti; **Event:** eventDate: 2011-07-03; **Record Level:** collectionCode: LFPC
- **Type status:** Other material. **Occurrence:** recordedBy: Leonardo Forbicioni; individualCount: 1; lifeStage: adult; occurrenceID: A02AD33E-B384-5646-B492-2723840501E3; **Taxon:** scientificName: Buprestis (Ancylocheira) novemmaculata novemmaculata Linnaeus, 1767; order: Coleoptera; family: Buprestidae; genus: Buprestis; subgenus: Ancylocheira; specificEpithet: novemmaculata; infraspecificEpithet: novemmaculata; scientificNameAuthorship: Linnaeus, 1767; **Location:** islandGroup: Tuscan Archipelago; island: Isola d'Elba; country: Italy; countryCode: IT; stateProvince: Livorno; county: Portoferraio; locality: Schiopparello/Le Prade; decimalLatitude: 42.796000; decimalLongitude: 10.345238; geodeticDatum: WGS84; coordinatePrecision: 0.0002; **Identification:** identifiedBy: L. Forbicioni; **Event:** eventDate: 2013-06-25; **Record Level:** collectionCode: LFPC
- **Type status:** Other material. **Occurrence:** recordedBy: Leonardo Forbicioni; individualCount: 1; lifeStage: adult; occurrenceID: 69EBA50F-525D-5060-A69C-9D915B8131C5; **Taxon:** scientificName: Buprestis (Ancylocheira) novemmaculata novemmaculata Linnaeus, 1767; order: Coleoptera; family: Buprestidae; genus: Buprestis; subgenus: Ancylocheira; specificEpithet: novemmaculata; infraspecificEpithet: novemmaculata; scientificNameAuthorship: Linnaeus, 1767; **Location:** islandGroup: Tuscan Archipelago; island: Isola d'Elba; country: Italy; countryCode: IT; stateProvince: Livorno; county: Campo nell'Elba; locality: Monte Perone; decimalLatitude: 42.777508; decimalLongitude: 10.201324; geodeticDatum: WGS84; coordinatePrecision: 0.0002; **Identification:** identifiedBy: E. Paggetti; **Event:** eventDate: 2012-09-08; **Record Level:** collectionCode: LFPC
- **Type status:** Other material. **Occurrence:** recordedBy: Leonardo Forbicioni; individualCount: 1; lifeStage: adult; occurrenceID: DEF3A88B-9725-5C97-B098-B1D838A99C41; **Taxon:** scientificName: Buprestis (Ancylocheira) novemmaculata novemmaculata Linnaeus, 1767; order: Coleoptera; family: Buprestidae; genus: Buprestis; subgenus: Ancylocheira; specificEpithet: novemmaculata; infraspecificEpithet: novemmaculata; scientificNameAuthorship: Linnaeus, 1767; **Location:** islandGroup: Tuscan Archipelago; island: Isola d'Elba; country: Italy; countryCode: IT; stateProvince: Livorno; county: Campo nell'Elba; locality: Monte Perone; decimalLatitude: 42.777979; decimalLongitude: 10.202846; geodeticDatum: WGS84; coordinatePrecision: 0.0002; **Identification:** identifiedBy: E. Paggetti; **Event:** eventDate: 2012-08-15; **Record Level:** collectionCode: LFPC
- **Type status:** Other material. **Occurrence:** individualCount: 1; lifeStage: adult; occurrenceID: E4D69AB1-D00D-5CB7-BEB7-E26CE6CC67F7; **Taxon:** scientificName: Buprestis (Ancylocheira) novemmaculata novemmaculata Linnaeus, 1767; order: Coleoptera; family: Buprestidae; genus: Buprestis; subgenus: Ancylocheira; specificEpithet: novemmaculata; infraspecificEpithet: novemmaculata; scientificNameAuthorship: Linnaeus, 1767; **Location:** islandGroup: Tuscan Archipelago; island: Isola d'Elba; country: Italy; countryCode: IT; stateProvince: Livorno; county: Portoferraio; **Record Level:** source: Razzauti A (1921) Contributi alla conoscenza faunistica delle isole toscane III. Coleotteri delle Isole d’Elba, di Capraia e di Gorgona Atti della Società toscana di Scienze naturali residente in Pisa. 33. Memorie, Pisa, 96-122 pp.. https://www.biodiversitylibrary.org/page/35335669 . Holdhaus K. (1923) Elenco dei Coleotteri dell’isola d’Elba con studi sul problema della Tirrenide. Memorie della Società entomologica italiana 2: 77‑175.
- **Type status:** Other material. **Occurrence:** individualCount: 1; lifeStage: adult; occurrenceID: A1994005-CC36-50F4-A341-994DA678707A; **Taxon:** scientificName: Buprestis (Ancylocheira) novemmaculata novemmaculata Linnaeus, 1767; order: Coleoptera; family: Buprestidae; genus: Buprestis; subgenus: Ancylocheira; specificEpithet: novemmaculata; infraspecificEpithet: novemmaculata; scientificNameAuthorship: Linnaeus, 1767; **Location:** islandGroup: Tuscan Archipelago; island: Isola di Gorgona; country: Italy; countryCode: IT; stateProvince: Livorno; county: Gorgona; **Record Level:** source: Razzauti A (1921) Contributi alla conoscenza faunistica delle isole toscane III. Coleotteri delle Isole d’Elba, di Capraia e di Gorgona Atti della Società toscana di Scienze naturali residente in Pisa. 33. Memorie, Pisa, 96-122 pp.. https://www.biodiversitylibrary.org/page/35335669
- **Type status:** Other material. **Occurrence:** individualCount: 1; lifeStage: adult; occurrenceID: EEAF40CD-4B68-5508-B8D8-BA94395A1E5B; **Taxon:** scientificName: Buprestis (Ancylocheira) novemmaculata novemmaculata Linnaeus, 1767; order: Coleoptera; family: Buprestidae; genus: Buprestis; subgenus: Ancylocheira; specificEpithet: novemmaculata; infraspecificEpithet: novemmaculata; scientificNameAuthorship: Linnaeus, 1767; **Location:** islandGroup: Tuscan Archipelago; island: Isola d'Elba; country: Italy; countryCode: IT; stateProvince: Livorno; **Record Level:** source: Holdhaus K. (1923) Elenco dei Coleotteri dell’isola d’Elba con studi sul problema della Tirrenide. Memorie della Società entomologica italiana 2: 77‑175. - Curletti G. (1994) I Buprestidi d’Italia. Catalogo geonemico, sinonimico, bibliografico, biologico. Monografie di Natura Bresciana, Ed. Vannini, Brescia, 19.
- **Type status:** Other material. **Occurrence:** individualCount: 1; lifeStage: adult; occurrenceID: A391FD74-F2EF-5BB1-9CBC-049932626962; **Taxon:** scientificName: Buprestis (Ancylocheira) novemmaculata novemmaculata Linnaeus, 1767; order: Coleoptera; family: Buprestidae; genus: Buprestis; subgenus: Ancylocheira; specificEpithet: novemmaculata; infraspecificEpithet: novemmaculata; scientificNameAuthorship: Linnaeus, 1767; **Location:** islandGroup: Tuscan Archipelago; island: Isola del Giglio; country: Italy; countryCode: GR; stateProvince: Grosseto; **Identification:** identifiedBy: G. Curletti; **Record Level:** source: Curletti G. (1994) I Buprestidi d’Italia. Catalogo geonemico, sinonimico, bibliografico, biologico. Monografie di Natura Bresciana, Ed. Vannini, Brescia, 19.

##### Conservation status

LC

##### Distribution

Recorded for the Tuscan Archipelago (Isola d'Elba, Isola del Giglio and Gorgona) by Holdhaus 1923 and Curletti 1994.

#### Eurythyrea micans

(Fabricius, 1792)

67711CF0-5D89-5051-8915-6D9084F8FAED

##### Materials

- **Type status:** Other material. **Occurrence:** recordedBy: Silvia Bracci; individualCount: 1; lifeStage: adult; occurrenceID: 4D0322DD-500D-57D8-9D55-DB841ECBA5D2; **Taxon:** scientificName: Eurythyreamicans (Fabricius, 1793); order: Coleoptera; family: Buprestidae; genus: Eurythyrea; specificEpithet: micans; scientificNameAuthorship: (Fabricius, 1793); **Location:** islandGroup: Tuscan Archipelago; island: Isola d'Elba; country: Italy; countryCode: IT; stateProvince: Livorno; county: Portoferraio; locality: Le Ghiaie; decimalLatitude: 42.816580; decimalLongitude: 10.324647; geodeticDatum: WGS84; coordinatePrecision: 6.0E-5; **Identification:** identifiedBy: L. Forbicioni; **Event:** eventDate: 2021-06-09; **Record Level:** source: https://www.inaturalist.org/observations/82303303
- **Type status:** Other material. **Occurrence:** recordedBy: Roberto Poggi; individualCount: 1; lifeStage: adult; occurrenceID: A0611CA9-FC9D-58C6-A698-E0D71850A0EB; **Taxon:** scientificName: Eurythyreamicans (Fabricius, 1793); order: Coleoptera; family: Buprestidae; genus: Eurythyrea; specificEpithet: micans; scientificNameAuthorship: (Fabricius, 1793); **Location:** islandGroup: Tuscan Archipelago; island: Isola di Montecristo; country: Italy; countryCode: IT; stateProvince: Livorno; county: Montecristo; locality: Collo dei Lecci; **Identification:** identifiedBy: R. Poggi; **Event:** eventDate: 1976-11-24; **Record Level:** source: Poggi R. 1976 (DOI: https://doi.org/10.21426/B65110051)
- **Type status:** Other material. **Occurrence:** individualCount: 1; lifeStage: adult; occurrenceID: E0D8BD1B-2E11-59D6-82E2-C877741FB4BC; **Taxon:** scientificName: Eurythyreamicans (Fabricius, 1793); order: Coleoptera; family: Buprestidae; genus: Eurythyrea; specificEpithet: micans; scientificNameAuthorship: (Fabricius, 1793); **Location:** islandGroup: Tuscan Archipelago; island: Isola del Giglio; country: Italy; countryCode: IT; stateProvince: Grosseto; **Identification:** identifiedBy: Gerini; **Record Level:** source: Poggi R. 1976 (DOI: https://doi.org/10.21426/B65110051)
- **Type status:** Other material. **Occurrence:** individualCount: 1; lifeStage: adult; occurrenceID: A7603C42-5084-5E2A-B254-CE86F3547BB7; **Taxon:** scientificName: Eurythyreamicans (Fabricius, 1793); order: Coleoptera; family: Buprestidae; genus: Eurythyrea; specificEpithet: micans; scientificNameAuthorship: (Fabricius, 1793); **Location:** islandGroup: Tuscan Archipelago; island: Isola di Montecristo; country: Italy; countryCode: IT; stateProvince: Livorno; county: Montecristo; **Identification:** identifiedBy: Gerini; **Record Level:** source: Curletti G. (1994) I Buprestidi d’Italia. Catalogo geonemico, sinonimico, bibliografico, biologico. Monografie di Natura Bresciana, Ed. Vannini, Brescia, 19.

##### Conservation status

LC

##### Distribution

Recorded for the Tuscan Archipelago (Isola di Montecristo and Isola del Giglio) by Poggi 1976 and Curletti 1994.

#### Eurythyrea quercus

(Herbst, 1780)

E5EA9FEC-B24B-5828-A1C9-2F116EACE0BE

##### Materials

- **Type status:** Other material. **Occurrence:** recordedBy: Leonardo Forbicioni & Enrico Ruzzier; individualCount: 1; lifeStage: adult; occurrenceID: F297148B-2B67-5DA3-A866-43BF13EADFDE; **Taxon:** scientificName: Eurythyreaquercus (Herbst, 1780); order: Coleoptera; family: Buprestidae; genus: Eurythyrea; specificEpithet: quercus; scientificNameAuthorship: (Herbst, 1780); **Location:** islandGroup: Tuscan Archipelago; island: Isola d'Elba; country: Italy; countryCode: IT; stateProvince: Livorno; county: Marciana; locality: Fonte di Zeno; decimalLatitude: 42.785630; decimalLongitude: 10.198226; geodeticDatum: WGS84; coordinatePrecision: 0.0002; **Identification:** identifiedBy: L. Forbicioni, G. Curletti, M. Gigli; **Event:** eventDate: 2021-11-20; **Record Level:** collectionCode: LFPC

##### Conservation status

CR

#### Chrysobothrini

D3A64671-9954-5121-938E-469D5185A969

#### Chrysobothris (Chrysobothris) affinis affinis

(Fabricius, 1794)

ED726165-1E3F-5F0E-975A-54DDEEA58C92

##### Materials

- **Type status:** Other material. **Occurrence:** recordedBy: Leonardo Forbicioni; individualCount: 1; lifeStage: adult; occurrenceID: B0D922A6-F464-52B9-9359-78E1C916884E; **Taxon:** scientificName: Chrysobothris (Chrysobothris) affinis affinis (Fabricius, 1794); order: Coleoptera; family: Buprestidae; genus: Chrysobothris; subgenus: Chrysobothris; specificEpithet: affinis; infraspecificEpithet: affinis; scientificNameAuthorship: (Fabricius, 1794); **Location:** islandGroup: Tuscan Archipelago; island: Isola d'Elba; country: Italy; countryCode: IT; stateProvince: Livorno; county: Portoferraio; locality: Volterraio; decimalLatitude: 42.800008; decimalLongitude: 10.388413; geodeticDatum: WGS84; coordinatePrecision: 0.0002; **Identification:** identifiedBy: L. Forbicioni; **Event:** eventDate: 2013-06-15; **Record Level:** collectionCode: LFPC
- **Type status:** Other material. **Occurrence:** recordedBy: Leonardo Forbicioni; individualCount: 2; lifeStage: adult; occurrenceID: C4B9C59C-99BA-54E7-B1C7-0F74989AE131; **Taxon:** scientificName: Chrysobothris (Chrysobothris) affinis affinis (Fabricius, 1794); order: Coleoptera; family: Buprestidae; genus: Chrysobothris; subgenus: Chrysobothris; specificEpithet: affinis; infraspecificEpithet: affinis; scientificNameAuthorship: (Fabricius, 1794); **Location:** islandGroup: Tuscan Archipelago; island: Isola d'Elba; country: Italy; countryCode: IT; stateProvince: Livorno; county: Portoferraio; locality: Acquabona; decimalLatitude: 42.785125; decimalLongitude: 10.345745; geodeticDatum: WGS84; coordinatePrecision: 0.0002; **Identification:** identifiedBy: L. Forbicioni; **Event:** eventDate: 2019-07-11; **Record Level:** collectionCode: LFPC

##### Conservation status

LC

#### Chrysobothris (Chrysobothris) solieri

Gory & Laporte, 1839

280BDF8A-AC90-591A-B455-FE1566F63B02

##### Materials

- **Type status:** Other material. **Occurrence:** recordedBy: Leonardo Forbicioni; individualCount: 2; lifeStage: adult; occurrenceID: 45B3C56A-BA4D-5F3C-811F-23AFE0BA5D16; **Taxon:** scientificName: Chrysobothris (Chrysobothris) solieri Gory & Laporte, 1839; order: Coleoptera; family: Buprestidae; genus: Chrysobothris; subgenus: Chrysobothris; specificEpithet: solieri; scientificNameAuthorship: Gory & Laporte, 1839; **Location:** islandGroup: Tuscan Archipelago; island: Isola d'Elba; country: Italy; countryCode: IT; stateProvince: Livorno; county: Campo nell'Elba; locality: Fetovaia; decimalLatitude: 42.733126; decimalLongitude: 10.145147; geodeticDatum: WGS84; coordinatePrecision: 0.0002; **Identification:** identifiedBy: E. Paggetti; **Event:** eventDate: 2014-06-08; **Record Level:** collectionCode: LFPC

##### Conservation status

LC

#### Chrysochroinae

736E65B2-9C95-571C-8EBF-7D2DEB3520E5

#### Chalchophorini

876C4766-44EC-530F-B889-95DABF79B1B7

#### Chalcophora massiliensis

(Villers, 1789)

7A03BFCB-2A19-5855-AAE2-96217D6B3FA3

##### Materials

- **Type status:** Other material. **Occurrence:** recordedBy: Leonardo Forbicioni; individualCount: 1; lifeStage: adult; occurrenceID: 414508BA-018A-58F9-9856-F929249CC444; **Taxon:** scientificName: Chalcophoramassiliensis (Villers, 1789); order: Coleoptera; family: Buprestidae; genus: Chalcophora; specificEpithet: massiliensis; scientificNameAuthorship: (Villers, 1789); **Location:** islandGroup: Tuscan Archipelago; island: Isola d'Elba; country: Italy; countryCode: IT; stateProvince: Livorno; county: Rio; municipality: Rio Marina; locality: Porticciolo; decimalLatitude: 42.806561; decimalLongitude: 10.428390; geodeticDatum: WGS84; coordinatePrecision: 0.0002; **Identification:** identifiedBy: L. Forbicioni; **Event:** eventDate: 2013-05-08; **Record Level:** collectionCode: LFPC
- **Type status:** Other material. **Occurrence:** recordedBy: Leonardo Forbicioni; individualCount: 1; lifeStage: adult; occurrenceID: D02C0798-2D91-56AF-99E7-8DDFE5D7C1C1; **Taxon:** scientificName: Chalcophoramassiliensis (Villers, 1789); order: Coleoptera; family: Buprestidae; genus: Chalcophora; specificEpithet: massiliensis; scientificNameAuthorship: (Villers, 1789); **Location:** islandGroup: Tuscan Archipelago; island: Isola d'Elba; country: Italy; countryCode: IT; stateProvince: Livorno; county: Portoferraio; locality: Colle Reciso/Mulino a vento; decimalLatitude: 42.778430; decimalLongitude: 10.290509; geodeticDatum: WGS84; coordinatePrecision: 0.0002; **Identification:** identifiedBy: L. Forbicioni; **Event:** eventDate: 2014-07-11; **Record Level:** collectionCode: LFPC
- **Type status:** Other material. **Occurrence:** recordedBy: Leonardo Forbicioni; individualCount: 4; lifeStage: adult; occurrenceID: CA8E3E36-A115-5DC2-B61B-850C57A16A5E; **Taxon:** scientificName: Chalcophoramassiliensis (Villers, 1789); order: Coleoptera; family: Buprestidae; genus: Chalcophora; specificEpithet: massiliensis; scientificNameAuthorship: (Villers, 1789); **Location:** islandGroup: Tuscan Archipelago; island: Isola d'Elba; country: Italy; countryCode: IT; stateProvince: Livorno; county: Portoferraio; locality: Colle Reciso/Mulino a vento; decimalLatitude: 42.778430; decimalLongitude: 10.290509; geodeticDatum: WGS84; coordinatePrecision: 0.0002; **Identification:** identifiedBy: E. Paggetti & F. Ceccolini; **Event:** eventDate: 2011-05-15; **Record Level:** collectionCode: LFPC
- **Type status:** Other material. **Occurrence:** recordedBy: Leonardo Forbicioni; individualCount: 1; lifeStage: adult; occurrenceID: 9A73ED4E-F1CF-55E1-8953-9648DC9E6587; **Taxon:** scientificName: Chalcophoramassiliensis (Villers, 1789); order: Coleoptera; family: Buprestidae; genus: Chalcophora; specificEpithet: massiliensis; scientificNameAuthorship: (Villers, 1789); **Location:** islandGroup: Tuscan Archipelago; island: Isola d'Elba; country: Italy; countryCode: IT; stateProvince: Livorno; county: Portoferraio; locality: Colle Reciso/Mulino a vento; decimalLatitude: 42.778430; decimalLongitude: 10.290509; geodeticDatum: WGS84; coordinatePrecision: 0.0002; **Identification:** identifiedBy: E. Paggetti & F. Ceccolini; **Event:** eventDate: 2011-05-17; **Record Level:** collectionCode: LFPC
- **Type status:** Other material. **Occurrence:** recordedBy: Leonardo Forbicioni; individualCount: 1; lifeStage: adult; occurrenceID: 78A9D1D7-4186-5E9E-A160-063C687AC501; **Taxon:** scientificName: Chalcophoramassiliensis (Villers, 1789); order: Coleoptera; family: Buprestidae; genus: Chalcophora; specificEpithet: massiliensis; scientificNameAuthorship: (Villers, 1789); **Location:** islandGroup: Tuscan Archipelago; island: Isola d'Elba; country: Italy; countryCode: IT; stateProvince: Livorno; county: Portoferraio; locality: Colle Reciso/Mulino a vento; decimalLatitude: 42.777442; decimalLongitude: 10.289100; geodeticDatum: WGS84; coordinatePrecision: 0.0002; **Identification:** identifiedBy: L. Forbicioni; **Event:** eventDate: 2013-07-23; **Record Level:** collectionCode: LFPC
- **Type status:** Other material. **Occurrence:** recordedBy: Leonardo Forbicioni; individualCount: 17; lifeStage: adult; occurrenceID: 4C928DD1-7B34-5529-9D75-B27595463C09; **Taxon:** scientificName: Chalcophoramassiliensis (Villers, 1789); order: Coleoptera; family: Buprestidae; genus: Chalcophora; specificEpithet: massiliensis; scientificNameAuthorship: (Villers, 1789); **Location:** islandGroup: Tuscan Archipelago; island: Isola d'Elba; country: Italy; countryCode: IT; stateProvince: Livorno; county: Portoferraio; locality: Monte Orello/Le Picchiaie; decimalLatitude: 42.780279; decimalLongitude: 10.326670; geodeticDatum: WGS84; coordinatePrecision: 0.0002; **Identification:** identifiedBy: L. Forbicioni; **Event:** eventDate: 2017-07-07; **Record Level:** collectionCode: SNPC
- **Type status:** Other material. **Occurrence:** recordedBy: Leonardo Forbicioni; individualCount: 16; lifeStage: adult; occurrenceID: B3A2C7C5-1429-5505-B116-3EA1C268406D; **Taxon:** scientificName: Chalcophoramassiliensis (Villers, 1789); order: Coleoptera; family: Buprestidae; genus: Chalcophora; specificEpithet: massiliensis; scientificNameAuthorship: (Villers, 1789); **Location:** islandGroup: Tuscan Archipelago; island: Isola d'Elba; country: Italy; countryCode: IT; stateProvince: Livorno; county: Portoferraio; locality: Monte Orello/Le Picchiaie; decimalLatitude: 42.780279; decimalLongitude: 10.326670; geodeticDatum: WGS84; coordinatePrecision: 0.0002; **Identification:** identifiedBy: L. Forbicioni; **Event:** eventDate: 2017-07-07; **Record Level:** collectionCode: MBPC
- **Type status:** Other material. **Occurrence:** recordedBy: Leonardo Forbicioni; individualCount: 2; lifeStage: adult; occurrenceID: 15CF93E1-9C2B-549A-9E54-8C2DDBA3EEDC; **Taxon:** scientificName: Chalcophoramassiliensis (Villers, 1789); order: Coleoptera; family: Buprestidae; genus: Chalcophora; specificEpithet: massiliensis; scientificNameAuthorship: (Villers, 1789); **Location:** islandGroup: Tuscan Archipelago; island: Isola d'Elba; country: Italy; countryCode: IT; stateProvince: Livorno; county: Portoferraio; locality: Monte Orello; decimalLatitude: 42.774648; decimalLongitude: 10.327994; geodeticDatum: WGS84; coordinatePrecision: 0.0002; **Identification:** identifiedBy: L. Forbicioni; **Event:** eventDate: 2012-05-25; **Record Level:** collectionCode: SNPC
- **Type status:** Other material. **Occurrence:** recordedBy: Leonardo Forbicioni; individualCount: 2; lifeStage: adult; occurrenceID: 6E6E6DB4-AABE-51B2-9B2E-E19255981D56; **Taxon:** scientificName: Chalcophoramassiliensis (Villers, 1789); order: Coleoptera; family: Buprestidae; genus: Chalcophora; specificEpithet: massiliensis; scientificNameAuthorship: (Villers, 1789); **Location:** islandGroup: Tuscan Archipelago; island: Isola d'Elba; country: Italy; countryCode: IT; stateProvince: Livorno; county: Portoferraio; locality: Monte Orello; decimalLatitude: 42.774648; decimalLongitude: 10.327994; geodeticDatum: WGS84; coordinatePrecision: 0.0002; **Identification:** identifiedBy: L. Forbicioni; **Event:** eventDate: 2012-05-25; **Record Level:** collectionCode: MBPC
- **Type status:** Other material. **Occurrence:** recordedBy: Leonardo Forbicioni; individualCount: 1; lifeStage: adult; occurrenceID: D1F46B8C-2959-5450-B001-88D764801500; **Taxon:** scientificName: Chalcophoramassiliensis (Villers, 1789); order: Coleoptera; family: Buprestidae; genus: Chalcophora; specificEpithet: massiliensis; scientificNameAuthorship: (Villers, 1789); **Location:** islandGroup: Tuscan Archipelago; island: Isola d'Elba; country: Italy; countryCode: IT; stateProvince: Livorno; county: Campo nell'Elba; locality: Monte Perone; decimalLatitude: 42.776278; decimalLongitude: 10.195311; geodeticDatum: WGS84; coordinatePrecision: 0.0002; **Identification:** identifiedBy: L. Forbicioni; **Event:** eventDate: 2013-08-15; **Record Level:** collectionCode: SNPC
- **Type status:** Other material. **Occurrence:** recordedBy: Leonardo Forbicioni; individualCount: 2; lifeStage: adult; occurrenceID: DBCC9E7A-E7F8-5DD0-8F8C-9D73F6C087B4; **Taxon:** scientificName: Chalcophoramassiliensis (Villers, 1789); order: Coleoptera; family: Buprestidae; genus: Chalcophora; specificEpithet: massiliensis; scientificNameAuthorship: (Villers, 1789); **Location:** islandGroup: Tuscan Archipelago; island: Isola d'Elba; country: Italy; countryCode: IT; stateProvince: Livorno; county: Campo nell'Elba; locality: Monte Perone; decimalLatitude: 42.776278; decimalLongitude: 10.195311; geodeticDatum: WGS84; coordinatePrecision: 0.0002; **Identification:** identifiedBy: L. Forbicioni; **Event:** eventDate: 2013-08-15; **Record Level:** collectionCode: MBPC
- **Type status:** Other material. **Occurrence:** recordedBy: Michele Carraretto; individualCount: 9; lifeStage: adult; occurrenceID: 31CF48F4-2DBB-5C21-9E3C-4E6AFCD97270; **Taxon:** scientificName: Chalcophoramassiliensis (Villers, 1789); order: Coleoptera; family: Buprestidae; genus: Chalcophora; specificEpithet: massiliensis; scientificNameAuthorship: (Villers, 1789); **Location:** islandGroup: Tuscan Archipelago; island: Isola d'Elba; country: Italy; countryCode: IT; stateProvince: Livorno; county: Campo nell'Elba; locality: Monte Perone; decimalLatitude: 42.777776; decimalLongitude: 10.202190; geodeticDatum: WGS84; coordinatePrecision: 0.0002; **Identification:** identifiedBy: L. Forbicioni; **Event:** eventDate: 2004-06-28; **Record Level:** collectionCode: SNPC
- **Type status:** Other material. **Occurrence:** recordedBy: Matteo Serafini; individualCount: 1; lifeStage: adult; occurrenceID: CE901D72-41A0-5535-A635-C30ED2D5249D; **Taxon:** scientificName: Chalcophoramassiliensis (Villers, 1789); order: Coleoptera; family: Buprestidae; genus: Chalcophora; specificEpithet: massiliensis; scientificNameAuthorship: (Villers, 1789); **Location:** islandGroup: Tuscan Archipelago; island: Isola d'Elba; country: Italy; countryCode: IT; stateProvince: Livorno; county: Portoferraio; locality: Santo Stefano/Le Trane; decimalLatitude: 42.789578; decimalLongitude: 10.363830; geodeticDatum: WGS84; coordinatePrecision: 0.0001; **Identification:** identifiedBy: L. Forbicioni; **Event:** eventDate: 2023-07-21; **Record Level:** source: https://www.inaturalist.org/observations/174090094
- **Type status:** Other material. **Occurrence:** recordedBy: Leonardo Forbicioni; individualCount: 2; lifeStage: adult; occurrenceID: B458A95D-1191-5375-BD44-23217BE90851; **Taxon:** scientificName: Chalcophoramassiliensis (Villers, 1789); order: Coleoptera; family: Buprestidae; genus: Chalcophora; specificEpithet: massiliensis; scientificNameAuthorship: (Villers, 1789); **Location:** islandGroup: Tuscan Archipelago; island: Isola d'Elba; country: Italy; countryCode: IT; stateProvince: Livorno; county: Portoferraio; decimalLatitude: 42.815288; decimalLongitude: 10.331755; geodeticDatum: WGS84; coordinatePrecision: 0.0001; **Identification:** identifiedBy: L. Forbicioni; **Event:** eventDate: 2023-06-25; **Record Level:** source: https://www.inaturalist.org/observations/169639923
- **Type status:** Other material. **Occurrence:** recordedBy: Lukas Diehl; individualCount: 1; lifeStage: adult; occurrenceID: 7D10D999-F677-5CCF-9AAD-D98AC83B1492; **Taxon:** scientificName: Chalcophoramassiliensis (Villers, 1789); order: Coleoptera; family: Buprestidae; genus: Chalcophora; specificEpithet: massiliensis; scientificNameAuthorship: (Villers, 1789); **Location:** islandGroup: Tuscan Archipelago; island: Isola d'Elba; country: Italy; countryCode: IT; stateProvince: Livorno; county: Marciana Marina; locality: Punta della Gioma; decimalLatitude: 42.805250; decimalLongitude: 10.168959; geodeticDatum: WGS84; coordinatePrecision: 0.002; **Identification:** identifiedBy: L. Forbicioni; **Event:** eventDate: 2023-06-08; **Record Level:** source: https://www.inaturalist.org/observations/168070227
- **Type status:** Other material. **Occurrence:** recordedBy: Simon Habermann; individualCount: 1; lifeStage: adult; occurrenceID: 7D7DDD02-AEEE-574E-AF1B-112C70F97F3B; **Taxon:** scientificName: Chalcophoramassiliensis (Villers, 1789); order: Coleoptera; family: Buprestidae; genus: Chalcophora; specificEpithet: massiliensis; scientificNameAuthorship: (Villers, 1789); **Location:** islandGroup: Tuscan Archipelago; island: Isola d'Elba; country: Italy; countryCode: IT; stateProvince: Livorno; county: Campo nell'Elba; municipality: Sant'Ilario; decimalLatitude: 42.762179; decimalLongitude: 10.218328; geodeticDatum: WGS84; coordinatePrecision: 1; **Identification:** identifiedBy: L. Forbicioni; **Event:** eventDate: 2023-06-02; **Record Level:** source: https://www.inaturalist.org/observations/165272190
- **Type status:** Other material. **Occurrence:** recordedBy: Lorenzo Sederini; individualCount: 1; lifeStage: adult; occurrenceID: 8DAA1130-2C2A-5D47-8457-296779C762C3; **Taxon:** scientificName: Chalcophoramassiliensis (Villers, 1789); order: Coleoptera; family: Buprestidae; genus: Chalcophora; specificEpithet: massiliensis; scientificNameAuthorship: (Villers, 1789); **Location:** islandGroup: Tuscan Archipelago; island: Isola d'Elba; country: Italy; countryCode: IT; stateProvince: Livorno; county: Campo nell'Elba; locality: Monte Perone; decimalLatitude: 42.776298; decimalLongitude: 10.197120; geodeticDatum: WGS84; coordinatePrecision: 0.0007; **Identification:** identifiedBy: L. Forbicioni; **Event:** eventDate: 2023-05-28; **Record Level:** source: https://www.inaturalist.org/observations/164163149
- **Type status:** Other material. **Occurrence:** recordedBy: Thomas Messner; individualCount: 1; lifeStage: adult; occurrenceID: 1B9FA5B8-3623-5119-806F-36A20AB95112; **Taxon:** scientificName: Chalcophoramassiliensis (Villers, 1789); order: Coleoptera; family: Buprestidae; genus: Chalcophora; specificEpithet: massiliensis; scientificNameAuthorship: (Villers, 1789); **Location:** islandGroup: Tuscan Archipelago; island: Isola d'Elba; country: Italy; countryCode: IT; stateProvince: Livorno; county: Marciana Marina; decimalLatitude: 42.807410; decimalLongitude: 10.190954; geodeticDatum: WGS84; coordinatePrecision: 9.0E-5; **Identification:** identifiedBy: L. Forbicioni; **Event:** eventDate: 2023-05-09; **Record Level:** source: https://www.inaturalist.org/observations/162174143
- **Type status:** Other material. **Occurrence:** recordedBy: Daniel Li Veli; individualCount: 1; lifeStage: adult; occurrenceID: E61EAA93-20A0-5515-95F5-3004E0B67DF0; **Taxon:** scientificName: Chalcophoramassiliensis (Villers, 1789); order: Coleoptera; family: Buprestidae; genus: Chalcophora; specificEpithet: massiliensis; scientificNameAuthorship: (Villers, 1789); **Location:** islandGroup: Tuscan Archipelago; island: Isola d'Elba; country: Italy; countryCode: IT; stateProvince: Livorno; county: Portoferraio; locality: San Giovanni; decimalLatitude: 42.801994; decimalLongitude: 10.325408; geodeticDatum: WGS84; coordinatePrecision: 0.2; **Identification:** identifiedBy: L. Forbicioni; **Event:** eventDate: 2023-05-07; **Record Level:** source: https://www.inaturalist.org/observations/160296358
- **Type status:** Other material. **Occurrence:** recordedBy: Arianna Longarini; individualCount: 1; lifeStage: adult; occurrenceID: EF0913EB-19BB-540C-AF65-F37198AC9639; **Taxon:** scientificName: Chalcophoramassiliensis (Villers, 1789); order: Coleoptera; family: Buprestidae; genus: Chalcophora; specificEpithet: massiliensis; scientificNameAuthorship: (Villers, 1789); **Location:** islandGroup: Tuscan Archipelago; island: Isola d'Elba; country: Italy; countryCode: IT; stateProvince: Livorno; county: Campo nell'Elba; locality: Pieve di San Giovanni; decimalLatitude: 42.760105; decimalLongitude: 10.195795; geodeticDatum: WGS84; coordinatePrecision: 0.0002; **Identification:** identifiedBy: L. Forbicioni; **Event:** eventDate: 2023-04-24; **Record Level:** source: https://www.inaturalist.org/observations/156577272
- **Type status:** Other material. **Occurrence:** recordedBy: Roberto Barsaglini; individualCount: 1; lifeStage: adult; occurrenceID: DED699C3-B2E1-53D4-B8C0-FD0E86D0154E; **Taxon:** scientificName: Chalcophoramassiliensis (Villers, 1789); order: Coleoptera; family: Buprestidae; genus: Chalcophora; specificEpithet: massiliensis; scientificNameAuthorship: (Villers, 1789); **Location:** islandGroup: Tuscan Archipelago; island: Isola d'Elba; country: Italy; countryCode: IT; stateProvince: Livorno; county: Campo nell'Elba; locality: Monte Perone; decimalLatitude: 42.776209; decimalLongitude: 10.195247; geodeticDatum: WGS84; coordinatePrecision: 0.0003; **Identification:** identifiedBy: L. Forbicioni; **Event:** eventDate: 2022-06-02; **Record Level:** source: https://www.inaturalist.org/observations/120043508
- **Type status:** Other material. **Occurrence:** recordedBy: Roberto Barsaglini; individualCount: 1; lifeStage: adult; occurrenceID: 1879D0B0-CE98-503B-9976-4B47F40FA1CC; **Taxon:** scientificName: Chalcophoramassiliensis (Villers, 1789); order: Coleoptera; family: Buprestidae; genus: Chalcophora; specificEpithet: massiliensis; scientificNameAuthorship: (Villers, 1789); **Location:** islandGroup: Tuscan Archipelago; island: Isola d'Elba; country: Italy; countryCode: IT; stateProvince: Livorno; county: Campo nell'Elba; locality: Monte Perone; decimalLatitude: 42.775081; decimalLongitude: 10.190845; geodeticDatum: WGS84; coordinatePrecision: 0.0001; **Identification:** identifiedBy: L. Forbicioni; **Event:** eventDate: 2021-08-15; **Record Level:** source: https://www.inaturalist.org/observations/91333011
- **Type status:** Other material. **Occurrence:** recordedBy: Carlotta Cicotti; individualCount: 1; lifeStage: adult; occurrenceID: 38A1B94D-6F69-505C-A806-0CE7BE4CE6DA; **Taxon:** scientificName: Chalcophoramassiliensis (Villers, 1789); order: Coleoptera; family: Buprestidae; genus: Chalcophora; specificEpithet: massiliensis; scientificNameAuthorship: (Villers, 1789); **Location:** islandGroup: Tuscan Archipelago; island: Isola d'Elba; country: Italy; countryCode: IT; stateProvince: Livorno; county: Marciana; locality: Colle di Procchio; decimalLatitude: 42.778488; decimalLongitude: 10.249245; geodeticDatum: WGS84; coordinatePrecision: 0.0004; **Identification:** identifiedBy: L. Forbicioni; **Event:** eventDate: 2021-05-06; **Record Level:** source: https://www.inaturalist.org/observations/77593169
- **Type status:** Other material. **Occurrence:** recordedBy: Giuseppe Mazza; individualCount: 1; lifeStage: adult; occurrenceID: 1E7DD568-7C12-5EC3-9020-97BC8DD45B6C; **Taxon:** scientificName: Chalcophoramassiliensis (Villers, 1789); order: Coleoptera; family: Buprestidae; genus: Chalcophora; specificEpithet: massiliensis; scientificNameAuthorship: (Villers, 1789); **Location:** islandGroup: Tuscan Archipelago; island: Isola d'Elba; country: Italy; countryCode: IT; stateProvince: Livorno; county: Capoliveri; locality: Monte Calamita; decimalLatitude: 42.727036; decimalLongitude: 10.404852; geodeticDatum: WGS84; coordinatePrecision: 9.0E-5; **Identification:** identifiedBy: L. Forbicioni; **Event:** eventDate: 2020-06-09; **Record Level:** source: www.inaturalist.org/observations/49015979
- **Type status:** Other material. **Occurrence:** individualCount: 1; lifeStage: adult; occurrenceID: 799BC0E1-C2CA-5EC3-AB9C-BCB8A4314726; **Taxon:** scientificName: Chalcophoramassiliensis (Villers, 1789); order: Coleoptera; family: Buprestidae; genus: Chalcophora; specificEpithet: massiliensis; scientificNameAuthorship: (Villers, 1789); **Location:** islandGroup: Tuscan Archipelago; island: Isola d'Elba; country: Italy; countryCode: IT; stateProvince: Livorno; county: Marciana; locality: Campo all'Aia; decimalLatitude: 42.786111; decimalLongitude: 10.257778; geodeticDatum: WGS84; **Identification:** identifiedBy: L. Forbicioni; **Event:** eventDate: 2020-06-02; **Record Level:** source: https://www.inaturalist.org/observations/48223715
- **Type status:** Other material. **Occurrence:** individualCount: 1; lifeStage: adult; occurrenceID: 1EF6A79E-6239-57E3-B6DF-D6FB87CE016C; **Taxon:** scientificName: Chalcophoramassiliensis (Villers, 1789); order: Coleoptera; family: Buprestidae; genus: Chalcophora; specificEpithet: massiliensis; scientificNameAuthorship: (Villers, 1789); **Location:** islandGroup: Tuscan Archipelago; island: Isola d'Elba; country: Italy; countryCode: IT; stateProvince: Livorno; county: Portoferraio; locality: Vecchio Papa; decimalLatitude: 42.786817; decimalLongitude: 10.335400; geodeticDatum: WGS84; coordinatePrecision: 9.0E-5; **Identification:** identifiedBy: L. Forbicioni; **Event:** eventDate: 2020-04-27; **Record Level:** source: https://www.inaturalist.org/observations/43871198
- **Type status:** Other material. **Occurrence:** recordedBy: Silvia Bracci; individualCount: 1; lifeStage: adult; occurrenceID: C1799588-FE1E-5E6C-BE18-1D5DB1978397; **Taxon:** scientificName: Chalcophoramassiliensis (Villers, 1789); order: Coleoptera; family: Buprestidae; genus: Chalcophora; specificEpithet: massiliensis; scientificNameAuthorship: (Villers, 1789); **Location:** islandGroup: Tuscan Archipelago; island: Isola d'Elba; country: Italy; countryCode: IT; stateProvince: Livorno; county: Marciana; locality: La Zanca; decimalLatitude: 42.801725; decimalLongitude: 10.135425; geodeticDatum: WGS84; coordinatePrecision: 0.001; **Identification:** identifiedBy: L. Forbicioni; **Event:** eventDate: 2016-08-03; **Record Level:** source: https://www.inaturalist.org/observations/42962323
- **Type status:** Other material. **Occurrence:** recordedBy: Silvia Bracci; individualCount: 1; lifeStage: adult; occurrenceID: FA1C14C2-6BCC-50CC-BFB1-D7CD2189F7F0; **Taxon:** scientificName: Chalcophoramassiliensis (Villers, 1789); order: Coleoptera; family: Buprestidae; genus: Chalcophora; specificEpithet: massiliensis; scientificNameAuthorship: (Villers, 1789); **Location:** islandGroup: Tuscan Archipelago; island: Isola d'Elba; country: Italy; countryCode: IT; stateProvince: Livorno; county: Marciana; locality: La Zanca; decimalLatitude: 42.801794; decimalLongitude: 10.135335; geodeticDatum: WGS84; coordinatePrecision: 0.001; **Identification:** identifiedBy: L. Forbicioni; **Event:** eventDate: 2016-08-09; **Record Level:** source: https://www.inaturalist.org/observations/42961948
- **Type status:** Other material. **Occurrence:** recordedBy: Leonardo Platania; individualCount: 1; lifeStage: adult; occurrenceID: 84357DE9-64B1-55B7-9F3F-D6722D1BC412; **Taxon:** scientificName: Chalcophoramassiliensis (Villers, 1789); order: Coleoptera; family: Buprestidae; genus: Chalcophora; specificEpithet: massiliensis; scientificNameAuthorship: (Villers, 1789); **Location:** islandGroup: Tuscan Archipelago; island: Isola d'Elba; country: Italy; countryCode: IT; stateProvince: Livorno; county: Campo nell'Elba; municipality: San Piero; decimalLatitude: 42.755448; decimalLongitude: 10.193786; geodeticDatum: WGS84; coordinatePrecision: 0.0002; **Identification:** identifiedBy: L. Forbicioni; **Event:** eventDate: 2017-04-23; **Record Level:** source: https://www.inaturalist.org/observations/42459774
- **Type status:** Other material. **Occurrence:** recordedBy: Riccardo Molajoli; individualCount: 1; lifeStage: adult; occurrenceID: 4A5969BE-C266-58C9-8B43-04E762D93FAD; **Taxon:** scientificName: Chalcophoramassiliensis (Villers, 1789); order: Coleoptera; family: Buprestidae; genus: Chalcophora; specificEpithet: massiliensis; scientificNameAuthorship: (Villers, 1789); **Location:** islandGroup: Tuscan Archipelago; island: Isola d'Elba; country: Italy; countryCode: IT; stateProvince: Livorno; county: Marciana; locality: Spartaia; decimalLatitude: 42.786865; decimalLongitude: 10.232176; geodeticDatum: WGS84; coordinatePrecision: 0.003; **Identification:** identifiedBy: L. Forbicioni; **Event:** eventDate: 2019-07-10; **Record Level:** source: https://www.inaturalist.org/observations/28858077
- **Type status:** Other material. **Occurrence:** individualCount: 1; lifeStage: adult; occurrenceID: D3048290-92FE-5BD3-B8A2-17752156309C; **Taxon:** scientificName: Chalcophoramassiliensis (Villers, 1789); order: Coleoptera; family: Buprestidae; genus: Chalcophora; specificEpithet: massiliensis; scientificNameAuthorship: (Villers, 1789); **Location:** islandGroup: Tuscan Archipelago; island: Isola del Giglio; country: Italy; countryCode: IT; stateProvince: Grosseto; county: Giglio Castello; decimalLatitude: 42.360101; decimalLongitude: 10.892591; geodeticDatum: WGS84; coordinatePrecision: 0.007; **Identification:** identifiedBy: L. Forbicioni; **Event:** eventDate: 2023-06-17; **Record Level:** source: https://www.inaturalist.org/observations/169834038
- **Type status:** Other material. **Occurrence:** recordedBy: Arne Fahrenholz; individualCount: 1; lifeStage: adult; occurrenceID: 1EA5219B-3A42-5CE6-8ABE-3CE2C0BC1283; **Taxon:** scientificName: Chalcophoramassiliensis (Villers, 1789); order: Coleoptera; family: Buprestidae; genus: Chalcophora; specificEpithet: massiliensis; scientificNameAuthorship: (Villers, 1789); **Location:** islandGroup: Tuscan Archipelago; island: Isola del Giglio; country: Italy; countryCode: IT; stateProvince: Grosseto; county: Campese; locality: Allume; decimalLatitude: 42.361574; decimalLongitude: 10.878304; geodeticDatum: WGS84; coordinatePrecision: 0.01; **Identification:** identifiedBy: L. Forbicioni; **Event:** eventDate: 2023-05-09; **Record Level:** source: https://www.inaturalist.org/observations/160859476
- **Type status:** Other material. **Occurrence:** recordedBy: Niklas Fudickar; individualCount: 1; lifeStage: adult; occurrenceID: CCF41E7A-6A53-5D98-A86B-E3F9E0F821F9; **Taxon:** scientificName: Chalcophoramassiliensis (Villers, 1789); order: Coleoptera; family: Buprestidae; genus: Chalcophora; specificEpithet: massiliensis; scientificNameAuthorship: (Villers, 1789); **Location:** islandGroup: Tuscan Archipelago; island: Isola del Giglio; country: Italy; countryCode: IT; stateProvince: Grosseto; county: Campese; locality: Allume; decimalLatitude: 42.363780; decimalLongitude: 10.879182; geodeticDatum: WGS84; coordinatePrecision: 0.0003; **Identification:** identifiedBy: L. Forbicioni; **Event:** eventDate: 2023-05-09; **Record Level:** source: www.inaturalist.org/observations/160720256
- **Type status:** Other material. **Occurrence:** recordedBy: Lorenzo Nesi; individualCount: 1; lifeStage: adult; occurrenceID: 1C4E7AEB-7FA7-5965-A153-08D92D23F2AA; **Taxon:** scientificName: Chalcophoramassiliensis (Villers, 1789); order: Coleoptera; family: Buprestidae; genus: Chalcophora; specificEpithet: massiliensis; scientificNameAuthorship: (Villers, 1789); **Location:** islandGroup: Tuscan Archipelago; island: Isola del Giglio; country: Italy; countryCode: IT; stateProvince: Grosseto; county: Giglio Porto; decimalLatitude: 42.360818; decimalLongitude: 10.919499; geodeticDatum: WGS84; coordinatePrecision: 0.001; **Identification:** identifiedBy: L. Forbicioni; **Event:** eventDate: 2020-09-13; **Record Level:** source: https://www.inaturalist.org/observations/59433350
- **Type status:** Other material. **Occurrence:** recordedBy: Franzini; individualCount: 1; lifeStage: adult; occurrenceID: ED51E778-0EBE-5956-97E3-6C84CB912407; **Taxon:** scientificName: Chalcophoramassiliensis (Villers, 1789); order: Coleoptera; family: Buprestidae; genus: Chalcophora; specificEpithet: massiliensis; scientificNameAuthorship: (Villers, 1789); **Location:** islandGroup: Tuscan Archipelago; island: Isola d'Elba; country: Italy; countryCode: IT; stateProvince: Livorno; county: Portoferraio; locality: Acquaviva; **Identification:** identifiedBy: Giovanni Gobbi; **Event:** eventDate: 1976-08-01; **Record Level:** source: Gobbi G. (1983) Interessanti reperti d Buprestidi italiani e diagnosi di Anthaxialiae n. sp. (Coleoptera, Buprestidae). Bollettino-Associazione romana di entomologia. XXXXVI (36): 33-41.
- **Type status:** Other material. **Occurrence:** recordedBy: Altobelli & Gobbi; individualCount: 1; lifeStage: adult; occurrenceID: 2527B1DE-E513-5773-A99F-0B2DCDE9CC54; **Taxon:** scientificName: Chalcophoramassiliensis (Villers, 1789); order: Coleoptera; family: Buprestidae; genus: Chalcophora; specificEpithet: massiliensis; scientificNameAuthorship: (Villers, 1789); **Location:** islandGroup: Tuscan Archipelago; island: Isola d'Elba; country: Italy; countryCode: IT; stateProvince: Livorno; county: Campo nell'Elba; locality: Monte Perone; **Identification:** identifiedBy: Giovanni Gobbi; **Event:** eventDate: 1979-07-01; **Record Level:** source: Gobbi G. (1983) Interessanti reperti d Buprestidi italiani e diagnosi di Anthaxialiae n. sp. (Coleoptera, Buprestidae). Bollettino-Associazione romana di entomologia. XXXXVI (36): 33-41.
- **Type status:** Other material. **Occurrence:** individualCount: 1; lifeStage: adult; occurrenceID: 7A537694-45A6-563B-8B12-82DD6637C916; **Taxon:** scientificName: Chalcophoramassiliensis (Villers, 1789); order: Coleoptera; family: Buprestidae; genus: Chalcophora; specificEpithet: massiliensis; scientificNameAuthorship: (Villers, 1789); **Location:** islandGroup: Tuscan Archipelago; island: Isola d'Elba; country: Italy; countryCode: IT; stateProvince: Livorno; county: Campo nell'Elba; locality: Monte Perone; **Identification:** identifiedBy: G. Curletti; **Record Level:** source: Curletti G. (1994) I Buprestidi d’Italia. Catalogo geonemico, sinonimico, bibliografico, biologico. Monografie di Natura Bresciana, Ed. Vannini, Brescia, 19.
- **Type status:** Other material. **Occurrence:** individualCount: 1; lifeStage: adult; occurrenceID: BC20F17D-2B26-5DD3-A23F-DCF380B84C3F; **Taxon:** scientificName: Chalcophoramassiliensis (Villers, 1789); order: Coleoptera; family: Buprestidae; genus: Chalcophora; specificEpithet: massiliensis; scientificNameAuthorship: (Villers, 1789); **Location:** islandGroup: Tuscan Archipelago; island: Isola d'Elba; country: Italy; countryCode: IT; stateProvince: Livorno; county: Campo nell'Elba; locality: Marina di Campo; **Identification:** identifiedBy: G. Curletti; **Record Level:** source: Curletti G. (1994) I Buprestidi d’Italia. Catalogo geonemico, sinonimico, bibliografico, biologico. Monografie di Natura Bresciana, Ed. Vannini, Brescia, 19.
- **Type status:** Other material. **Occurrence:** individualCount: 1; lifeStage: adult; occurrenceID: 79A9477C-8C6D-57F3-951F-7F65E3ACF6F8; **Taxon:** scientificName: Chalcophoramassiliensis (Villers, 1789); order: Coleoptera; family: Buprestidae; genus: Chalcophora; specificEpithet: massiliensis; scientificNameAuthorship: (Villers, 1789); **Location:** islandGroup: Tuscan Archipelago; island: Isola d'Elba; country: Italy; countryCode: IT; stateProvince: Livorno; county: Portoferraio; **Identification:** identifiedBy: G. Curletti; **Record Level:** source: Curletti G. (1994) I Buprestidi d’Italia. Catalogo geonemico, sinonimico, bibliografico, biologico. Monografie di Natura Bresciana, Ed. Vannini, Brescia, 19.
- **Type status:** Other material. **Occurrence:** individualCount: 1; lifeStage: adult; occurrenceID: F9A52837-19FD-5A40-AA96-2E7CA2ADD49B; **Taxon:** scientificName: Chalcophoramassiliensis (Villers, 1789); order: Coleoptera; family: Buprestidae; genus: Chalcophora; specificEpithet: massiliensis; scientificNameAuthorship: (Villers, 1789); **Location:** islandGroup: Tuscan Archipelago; island: Isola d'Elba; country: Italy; countryCode: IT; stateProvince: Livorno; county: Rio; **Identification:** identifiedBy: G. Curletti; **Record Level:** source: Curletti G. (1994) I Buprestidi d’Italia. Catalogo geonemico, sinonimico, bibliografico, biologico. Monografie di Natura Bresciana, Ed. Vannini, Brescia, 19.

##### Conservation status

LC

##### Distribution

Recorded for the Tuscan Archipelago (Isola d'Elba) by Gobbi (1983).

#### Dicercini

2642DFF5-31E5-5D1B-922A-289D8A50AAA6

#### Capnodis cariosa cariosa

(Pallas, 1776)

4F57B25F-5DE4-5B6F-AE58-105ACCF94C04

##### Materials

- **Type status:** Other material. **Occurrence:** individualCount: 1; lifeStage: adult; occurrenceID: E25E21DD-A9A5-5679-B934-EBD34BC2B06A; **Taxon:** scientificName: Capnodiscariosacariosa (Pallas, 1776); order: Coleoptera; family: Buprestidae; genus: Capnodis; specificEpithet: cariosa; infraspecificEpithet: cariosa; scientificNameAuthorship: (Pallas, 1776); **Location:** islandGroup: Tuscan Archipelago; island: Isola d'Elba; country: Italy; countryCode: IT; stateProvince: Livorno; county: Marciana; **Record Level:** source: Razzauti A (1921) Contributi alla conoscenza faunistica delle isole toscane III. Coleotteri delle Isole d’Elba, di Capraia e di Gorgona Atti della Società toscana di Scienze naturali residente in Pisa. 33. Memorie, Pisa, 96-122 pp.. https://www.biodiversitylibrary.org/page/35335669 . Holdhaus K. (1923) Elenco dei Coleotteri dell’isola d’Elba con studi sul problema della Tirrenide. Memorie della Società entomologica italiana 2: 77‑175. - Curletti G. (1994) I Buprestidi d’Italia. Catalogo geonemico, sinonimico, bibliografico, biologico. Monografie di Natura Bresciana, Ed. Vannini, Brescia, 19.

##### Conservation status

LC

##### Distribution

Recorded for the Tuscan Archipelago (Isola d'Elba) by Holdhaus (1923).

#### Capnodis tenebricosa tenebricosa

(Olivier, 1790)

234F2DD3-8311-55CD-A050-4B19C32F6BC5

##### Materials

- **Type status:** Other material. **Occurrence:** recordedBy: Leonardo Forbicioni; individualCount: 1; lifeStage: adult; occurrenceID: 9D4D9190-B46A-5553-AB57-81171E5A2742; **Taxon:** scientificName: Capnodistenebricosatenebricosa (A.G.Olivier, 1790); order: Coleoptera; family: Buprestidae; genus: Capnodis; specificEpithet: tenebricosa; infraspecificEpithet: tenebricosa; scientificNameAuthorship: (A.G.Olivier, 1790); **Location:** islandGroup: Tuscan Archipelago; island: Isola d'Elba; country: Italy; countryCode: IT; stateProvince: Livorno; county: Capoliveri; locality: Le Calanchiole; decimalLatitude: 42.759361; decimalLongitude: 10.365511; geodeticDatum: WGS84; coordinatePrecision: 0.0002; **Identification:** identifiedBy: L. Forbicioni; **Event:** eventDate: 2013-06-08; **Record Level:** collectionCode: LFPC
- **Type status:** Other material. **Occurrence:** recordedBy: Leonardo Forbicioni; individualCount: 1; lifeStage: adult; occurrenceID: FB8A4914-A18F-5A1E-92D1-45DC9971955E; **Taxon:** scientificName: Capnodistenebricosatenebricosa (A.G.Olivier, 1790); order: Coleoptera; family: Buprestidae; genus: Capnodis; specificEpithet: tenebricosa; infraspecificEpithet: tenebricosa; scientificNameAuthorship: (A.G.Olivier, 1790); **Location:** islandGroup: Tuscan Archipelago; island: Isola d'Elba; country: Italy; countryCode: IT; stateProvince: Livorno; county: Portoferraio; locality: Colle Reciso/Mulino a vento; decimalLatitude: 42.778923; decimalLongitude: 10.294423; geodeticDatum: WGS84; coordinatePrecision: 0.0002; **Identification:** identifiedBy: L. Forbicioni; **Event:** eventDate: 2011-07-03; **Record Level:** collectionCode: LFPC
- **Type status:** Other material. **Occurrence:** recordedBy: Leonardo Forbicioni; individualCount: 1; lifeStage: adult; occurrenceID: 1F1CD144-4D2E-5E12-9BA2-CCB9FDB690CA; **Taxon:** scientificName: Capnodistenebricosatenebricosa (A.G.Olivier, 1790); order: Coleoptera; family: Buprestidae; genus: Capnodis; specificEpithet: tenebricosa; infraspecificEpithet: tenebricosa; scientificNameAuthorship: (A.G.Olivier, 1790); **Location:** islandGroup: Tuscan Archipelago; island: Isola d'Elba; country: Italy; countryCode: IT; stateProvince: Livorno; county: Portoferraio; locality: Colle Reciso/Mulino a vento; decimalLatitude: 42.778701; decimalLongitude: 10.290473; geodeticDatum: WGS84; coordinatePrecision: 0.0002; **Identification:** identifiedBy: L. Forbicioni; **Event:** eventDate: 2008-07-20; **Record Level:** collectionCode: LFPC
- **Type status:** Other material. **Occurrence:** recordedBy: Giuliano Frangini; individualCount: 1; lifeStage: adult; occurrenceID: 3057BA54-D063-5507-A7FD-FDE6C84CB30B; **Taxon:** scientificName: Capnodistenebricosatenebricosa (A.G.Olivier, 1790); order: Coleoptera; family: Buprestidae; genus: Capnodis; specificEpithet: tenebricosa; infraspecificEpithet: tenebricosa; scientificNameAuthorship: (A.G.Olivier, 1790); **Location:** islandGroup: Tuscan Archipelago; island: Isola d'Elba; country: Italy; countryCode: IT; stateProvince: Livorno; county: Portoferraio; locality: Albereto; decimalLatitude: 42.809652; decimalLongitude: 10.305888; geodeticDatum: WGS84; coordinatePrecision: 0.0002; **Identification:** identifiedBy: L. Forbicioni; **Event:** eventDate: 2010-10-02; **Record Level:** collectionCode: LFPC
- **Type status:** Other material. **Occurrence:** individualCount: 1; lifeStage: adult; occurrenceID: 1A407882-1AC4-5E60-9065-270C633622D6; **Taxon:** scientificName: Capnodistenebricosatenebricosa (A.G.Olivier, 1790); order: Coleoptera; family: Buprestidae; genus: Capnodis; specificEpithet: tenebricosa; infraspecificEpithet: tenebricosa; scientificNameAuthorship: (A.G.Olivier, 1790); **Location:** islandGroup: Tuscan Archipelago; island: Isola d'Elba; country: Italy; countryCode: IT; stateProvince: Livorno; county: Marciana Marina; decimalLatitude: 42.804304; decimalLongitude: 10.194503; geodeticDatum: WGS84; coordinatePrecision: 0.001; **Identification:** identifiedBy: L. Forbicioni; **Event:** eventDate: 2023-06-05; **Record Level:** source: https://www.inaturalist.org/observations/166277316
- **Type status:** Other material. **Occurrence:** recordedBy: Riccardo Molajoli; individualCount: 1; lifeStage: adult; occurrenceID: 0A7E20DD-F187-5C37-BD49-8EF7675E53E4; **Taxon:** scientificName: Capnodistenebricosatenebricosa (A.G.Olivier, 1790); order: Coleoptera; family: Buprestidae; genus: Capnodis; specificEpithet: tenebricosa; infraspecificEpithet: tenebricosa; scientificNameAuthorship: (A.G.Olivier, 1790); **Location:** islandGroup: Tuscan Archipelago; island: Isola d'Elba; country: Italy; countryCode: IT; stateProvince: Livorno; county: Marciana; locality: Procchio; decimalLatitude: 42.786797; decimalLongitude: 10.236618; geodeticDatum: WGS84; **Identification:** identifiedBy: L. Forbicioni; **Event:** eventDate: 2022-08-20; **Record Level:** source: https://www.inaturalist.org/observations/131640485
- **Type status:** Other material. **Occurrence:** recordedBy: Roberto Barsaglini; individualCount: 1; lifeStage: adult; occurrenceID: 7F3E5001-BB6A-5292-B492-EF370375074D; **Taxon:** scientificName: Capnodistenebricosatenebricosa (A.G.Olivier, 1790); order: Coleoptera; family: Buprestidae; genus: Capnodis; specificEpithet: tenebricosa; infraspecificEpithet: tenebricosa; scientificNameAuthorship: (A.G.Olivier, 1790); **Location:** islandGroup: Tuscan Archipelago; island: Isola d'Elba; country: Italy; countryCode: IT; stateProvince: Livorno; county: Capoliveri; locality: Lido di Capoliveri; decimalLatitude: 42.762960; decimalLongitude: 10.362333; geodeticDatum: WGS84; coordinatePrecision: 9.0E-5; **Identification:** identifiedBy: L. Forbicioni; **Event:** eventDate: 2019-10-16; **Record Level:** source: https://www.inaturalist.org/observations/34530034
- **Type status:** Other material. **Occurrence:** individualCount: 1; lifeStage: adult; occurrenceID: 3B0EBD44-AD33-5858-8965-241DEB60B125; **Taxon:** scientificName: Capnodistenebricosatenebricosa (A.G.Olivier, 1790); order: Coleoptera; family: Buprestidae; genus: Capnodis; specificEpithet: tenebricosa; infraspecificEpithet: tenebricosa; scientificNameAuthorship: (A.G.Olivier, 1790); **Location:** islandGroup: Tuscan Archipelago; island: Isola d'Elba; country: Italy; countryCode: IT; stateProvince: Livorno; county: Portoferraio; **Record Level:** source: Razzauti A (1921) Contributi alla conoscenza faunistica delle isole toscane III. Coleotteri delle Isole d’Elba, di Capraia e di Gorgona Atti della Società toscana di Scienze naturali residente in Pisa. 33. Memorie, Pisa, 96-122 pp.. https://www.biodiversitylibrary.org/page/35335669
- **Type status:** Other material. **Occurrence:** individualCount: 1; lifeStage: adult; occurrenceID: 0EF4FE94-823E-5F23-83C7-35367C85057B; **Taxon:** scientificName: Capnodistenebricosatenebricosa (A.G.Olivier, 1790); order: Coleoptera; family: Buprestidae; genus: Capnodis; specificEpithet: tenebricosa; infraspecificEpithet: tenebricosa; scientificNameAuthorship: (A.G.Olivier, 1790); **Location:** islandGroup: Tuscan Archipelago; island: Isola d'Elba; country: Italy; countryCode: IT; stateProvince: Livorno; county: Marciana; **Record Level:** source: Razzauti A (1921) Contributi alla conoscenza faunistica delle isole toscane III. Coleotteri delle Isole d’Elba, di Capraia e di Gorgona Atti della Società toscana di Scienze naturali residente in Pisa. 33. Memorie, Pisa, 96-122 pp.. https://www.biodiversitylibrary.org/page/35335669
- **Type status:** Other material. **Occurrence:** individualCount: 1; lifeStage: adult; occurrenceID: 1033CCA3-9095-5FF5-A9EF-A7DF0EEBDDD5; **Taxon:** scientificName: Capnodistenebricosatenebricosa (A.G.Olivier, 1790); order: Coleoptera; family: Buprestidae; genus: Capnodis; specificEpithet: tenebricosa; infraspecificEpithet: tenebricosa; scientificNameAuthorship: (A.G.Olivier, 1790); **Location:** islandGroup: Tuscan Archipelago; island: Isola d'Elba; country: Italy; countryCode: IT; stateProvince: Livorno; county: Marciana; **Record Level:** source: Holdhaus K. (1923) Elenco dei Coleotteri dell’isola d’Elba con studi sul problema della Tirrenide. Memorie della Società entomologica italiana 2: 77‑175. - Curletti G. (1994) I Buprestidi d’Italia. Catalogo geonemico, sinonimico, bibliografico, biologico. Monografie di Natura Bresciana, Ed. Vannini, Brescia, 19.
- **Type status:** Other material. **Occurrence:** individualCount: 1; lifeStage: adult; occurrenceID: 66C5A11A-F4C6-59CE-B0F4-7128C318D9C7; **Taxon:** scientificName: Capnodistenebricosatenebricosa (A.G.Olivier, 1790); order: Coleoptera; family: Buprestidae; genus: Capnodis; specificEpithet: tenebricosa; infraspecificEpithet: tenebricosa; scientificNameAuthorship: (A.G.Olivier, 1790); **Location:** islandGroup: Tuscan Archipelago; island: Isola d'Elba; country: Italy; countryCode: IT; stateProvince: Livorno; county: Portoferraio; **Record Level:** source: Holdhaus K. (1923) Elenco dei Coleotteri dell’isola d’Elba con studi sul problema della Tirrenide. Memorie della Società entomologica italiana 2: 77‑175. - Curletti G. (1994) I Buprestidi d’Italia. Catalogo geonemico, sinonimico, bibliografico, biologico. Monografie di Natura Bresciana, Ed. Vannini, Brescia, 19.

##### Distribution

Recorded for the Tuscan Archipelago (Isola d'Elba) by Holdhaus (1923).

#### Capnodis tenebrionis

(Linnaeus, 1761)

6C4DC0AA-AF23-5EDF-8AFF-4DECA6F25269

##### Materials

- **Type status:** Other material. **Occurrence:** recordedBy: Roberto Ballini; individualCount: 1; lifeStage: adult; occurrenceID: 85100B73-CB87-5651-BA30-48A31B10A25A; **Taxon:** scientificName: Capnodistenebrionis (Linnaeus, 1761); order: Coleoptera; family: Buprestidae; genus: Capnodis; specificEpithet: tenebrionis; scientificNameAuthorship: (Linnaeus, 1761); **Location:** islandGroup: Tuscan Archipelago; island: Isola d'Elba; country: Italy; countryCode: IT; stateProvince: Livorno; county: Rio; municipality: Rio nell'Elba; locality: Santa Caterina; decimalLatitude: 42.827137; decimalLongitude: 10.409287; geodeticDatum: WGS84; coordinatePrecision: 0.0002; **Identification:** identifiedBy: L. Forbicioni; **Event:** eventDate: 2014-06-10; **Record Level:** collectionCode: LFPC
- **Type status:** Other material. **Occurrence:** recordedBy: Leonardo Forbicioni; individualCount: 1; lifeStage: adult; occurrenceID: 31BFC13B-64BA-5DA6-B6F9-CDAC90BD51DD; **Taxon:** scientificName: Capnodistenebrionis (Linnaeus, 1761); order: Coleoptera; family: Buprestidae; genus: Capnodis; specificEpithet: tenebrionis; scientificNameAuthorship: (Linnaeus, 1761); **Location:** islandGroup: Tuscan Archipelago; island: Isola d'Elba; country: Italy; countryCode: IT; stateProvince: Livorno; county: Portoferraio; locality: Via Mentana; decimalLatitude: 42.811824; decimalLongitude: 10.310799; geodeticDatum: WGS84; coordinatePrecision: 0.0002; **Identification:** identifiedBy: L. Forbicioni; **Event:** eventDate: 2022-03-28; **Record Level:** collectionCode: LFPC
- **Type status:** Other material. **Occurrence:** recordedBy: Leonardo Forbicioni; individualCount: 1; lifeStage: adult; occurrenceID: A3CD6389-3B2C-5EB8-9A09-5DAD505C99AD; **Taxon:** scientificName: Capnodistenebrionis (Linnaeus, 1761); order: Coleoptera; family: Buprestidae; genus: Capnodis; specificEpithet: tenebrionis; scientificNameAuthorship: (Linnaeus, 1761); **Location:** islandGroup: Tuscan Archipelago; island: Isola d'Elba; country: Italy; countryCode: IT; stateProvince: Livorno; county: Portoferraio; locality: San Giovanni; decimalLatitude: 42.801569; decimalLongitude: 10.316184; geodeticDatum: WGS84; coordinatePrecision: 0.0002; **Identification:** identifiedBy: L. Forbicioni; **Event:** eventDate: 2008-06-20; **Record Level:** collectionCode: LFPC
- **Type status:** Other material. **Occurrence:** recordedBy: Leonardo Forbicioni; individualCount: 1; lifeStage: adult; occurrenceID: F42DC0EE-C5EE-5BDF-8FF5-58E81B99381A; **Taxon:** scientificName: Capnodistenebrionis (Linnaeus, 1761); order: Coleoptera; family: Buprestidae; genus: Capnodis; specificEpithet: tenebrionis; scientificNameAuthorship: (Linnaeus, 1761); **Location:** islandGroup: Tuscan Archipelago; island: Isola d'Elba; country: Italy; countryCode: IT; stateProvince: Livorno; county: Campo nell'Elba; municipality: San Piero; locality: Piana al Canale; decimalLatitude: 42.755220; decimalLongitude: 10.189910; geodeticDatum: WGS84; coordinatePrecision: 0.0002; **Identification:** identifiedBy: L. Forbicioni; **Event:** eventDate: 2015-07-30; **Record Level:** collectionCode: LFPC
- **Type status:** Other material. **Occurrence:** recordedBy: Alberto Inghilesi; individualCount: 1; lifeStage: adult; occurrenceID: 5B33971B-CF7B-58F8-A101-0D3B0351FC18; **Taxon:** scientificName: Capnodistenebrionis (Linnaeus, 1761); order: Coleoptera; family: Buprestidae; genus: Capnodis; specificEpithet: tenebrionis; scientificNameAuthorship: (Linnaeus, 1761); **Location:** islandGroup: Tuscan Archipelago; island: Isola d'Elba; country: Italy; countryCode: IT; stateProvince: Livorno; county: Rio; municipality: Rio nell'Elba; locality: Nisportino; decimalLatitude: 42.831872; decimalLongitude: 10.390034; geodeticDatum: WGS84; coordinatePrecision: 0.0002; **Identification:** identifiedBy: L. Forbicioni; **Event:** eventDate: 2023-07-29; **Record Level:** source: https://www.inaturalist.org/observations/175530457
- **Type status:** Other material. **Occurrence:** recordedBy: Leonardo Mantovani; individualCount: 1; lifeStage: adult; occurrenceID: B1EB78FD-998F-592B-BD5A-8C00D07783C6; **Taxon:** scientificName: Capnodistenebrionis (Linnaeus, 1761); order: Coleoptera; family: Buprestidae; genus: Capnodis; specificEpithet: tenebrionis; scientificNameAuthorship: (Linnaeus, 1761); **Location:** islandGroup: Tuscan Archipelago; island: Isola d'Elba; country: Italy; countryCode: IT; stateProvince: Livorno; county: Campo nell'Elba; locality: Pomonte; decimalLatitude: 42.748686; decimalLongitude: 10.121622; geodeticDatum: WGS84; coordinatePrecision: 0.003; **Identification:** identifiedBy: L. Forbicioni; **Event:** eventDate: 2022-08-27; **Record Level:** source: https://www.inaturalist.org/observations/132520819
- **Type status:** Other material. **Occurrence:** recordedBy: Francesca Romana Dani; individualCount: 1; lifeStage: adult; occurrenceID: 20507415-AD5B-5408-A245-F61EF3A9A568; **Taxon:** scientificName: Capnodistenebrionis (Linnaeus, 1761); order: Coleoptera; family: Buprestidae; genus: Capnodis; specificEpithet: tenebrionis; scientificNameAuthorship: (Linnaeus, 1761); **Location:** islandGroup: Tuscan Archipelago; island: Isola d'Elba; country: Italy; countryCode: IT; stateProvince: Livorno; county: Campo nell'Elba; locality: Cavoli; decimalLatitude: 42.741026; decimalLongitude: 10.187186; geodeticDatum: WGS84; coordinatePrecision: 4.0E-5; **Identification:** identifiedBy: L. Forbicioni; **Event:** eventDate: 2022-08-14; **Record Level:** source: https://www.inaturalist.org/observations/130765447
- **Type status:** Other material. **Occurrence:** recordedBy: Emanuele Santarelli; individualCount: 1; lifeStage: adult; occurrenceID: 9A5299C3-1A8E-5C4E-840D-CF8571532972; **Taxon:** scientificName: Capnodistenebrionis (Linnaeus, 1761); order: Coleoptera; family: Buprestidae; genus: Capnodis; specificEpithet: tenebrionis; scientificNameAuthorship: (Linnaeus, 1761); **Location:** islandGroup: Tuscan Archipelago; island: Isola d'Elba; country: Italy; countryCode: IT; stateProvince: Livorno; county: Marciana; locality: Sant'Andrea; decimalLatitude: 42.806464; decimalLongitude: 10.142381; geodeticDatum: WGS84; coordinatePrecision: 0.0006; **Identification:** identifiedBy: L. Forbicioni; **Event:** eventDate: 2019-08-13; **Record Level:** source: https://www.inaturalist.org/observations/30865839
- **Type status:** Other material. **Occurrence:** recordedBy: Marco Serrajotto; individualCount: 1; lifeStage: adult; occurrenceID: D7773911-9D4A-5D32-95E2-95545108F1CF; **Taxon:** scientificName: Capnodistenebrionis (Linnaeus, 1761); order: Coleoptera; family: Buprestidae; genus: Capnodis; specificEpithet: tenebrionis; scientificNameAuthorship: (Linnaeus, 1761); **Location:** islandGroup: Tuscan Archipelago; island: Isola d'Elba; country: Italy; countryCode: IT; stateProvince: Livorno; county: Rio; municipality: Rio nell'Elba; locality: Santa Caterina; decimalLatitude: 42.826797; decimalLongitude: 10.409312; geodeticDatum: WGS84; coordinatePrecision: 7.0E-5; **Identification:** identifiedBy: L. Forbicioni; **Event:** eventDate: 2019-07-18; **Record Level:** source: https://www.inaturalist.org/observations/29051826
- **Type status:** Other material. **Occurrence:** recordedBy: Leonardo Forbicioni; individualCount: 1; lifeStage: adult; occurrenceID: 287396F4-F390-5F4B-9759-1EB6FCD4E59F; **Taxon:** scientificName: Capnodistenebrionis (Linnaeus, 1761); order: Coleoptera; family: Buprestidae; genus: Capnodis; specificEpithet: tenebrionis; scientificNameAuthorship: (Linnaeus, 1761); **Location:** islandGroup: Tuscan Archipelago; island: Isola d'Elba; country: Italy; countryCode: IT; stateProvince: Livorno; county: Portoferraio; locality: San Martino; decimalLatitude: 42.783012; decimalLongitude: 10.281719; geodeticDatum: WGS84; coordinatePrecision: 0.0002; **Identification:** identifiedBy: L. Forbicioni; **Event:** eventDate: 2018-08-30; **Record Level:** collectionCode: LFPC
- **Type status:** Other material. **Occurrence:** individualCount: 1; lifeStage: adult; occurrenceID: DE548645-37F3-515C-89B7-FEC6C9B41685; **Taxon:** scientificName: Capnodistenebrionis (Linnaeus, 1761); order: Coleoptera; family: Buprestidae; genus: Capnodis; specificEpithet: tenebrionis; scientificNameAuthorship: (Linnaeus, 1761); **Location:** islandGroup: Tuscan Archipelago; island: Isola d'Elba; country: Italy; countryCode: IT; stateProvince: Livorno; **Record Level:** source: Holdhaus K. (1923) Elenco dei Coleotteri dell’isola d’Elba con studi sul problema della Tirrenide. Memorie della Società entomologica italiana 2: 77‑175. - Curletti G. (1994) I Buprestidi d’Italia. Catalogo geonemico, sinonimico, bibliografico, biologico. Monografie di Natura Bresciana, Ed. Vannini, Brescia, 19.
- **Type status:** Other material. **Occurrence:** individualCount: 1; lifeStage: adult; occurrenceID: 5C7AB6CE-930A-5D79-B6C4-5946533275D5; **Taxon:** scientificName: Capnodistenebrionis (Linnaeus, 1761); order: Coleoptera; family: Buprestidae; genus: Capnodis; specificEpithet: tenebrionis; scientificNameAuthorship: (Linnaeus, 1761); **Location:** islandGroup: Tuscan Archipelago; island: Isola d'Elba; country: Italy; countryCode: IT; stateProvince: Livorno; county: Marciana; **Record Level:** source: Razzauti A (1921) Contributi alla conoscenza faunistica delle isole toscane III. Coleotteri delle Isole d’Elba, di Capraia e di Gorgona Atti della Società toscana di Scienze naturali residente in Pisa. 33. Memorie, Pisa, 96-122 pp.. https://www.biodiversitylibrary.org/page/35335669

##### Conservation status

LC

##### Distribution

Recorded for the Tuscan Archipelago (Isola d'Elba) by Razzauti 1921 and Holdhaus 1923.

#### Dicerca aenea aenea

(Linnaeus, 1767)

912F10C8-B43C-5804-8DDC-24111C812260

##### Materials

- **Type status:** Other material. **Occurrence:** recordedBy: Leonardo Forbicioni; individualCount: 1; lifeStage: adult; occurrenceID: 62711B47-2BED-5874-98DD-1CB0D3203662; **Taxon:** scientificName: Dicercaaeneaaenea (Linnaeus, 1767); order: Coleoptera; family: Buprestidae; genus: Dicerca; specificEpithet: aenea; infraspecificEpithet: aenea; scientificNameAuthorship: (Linnaeus, 1767); **Location:** islandGroup: Tuscan Archipelago; island: Isola d'Elba; country: Italy; countryCode: IT; stateProvince: Livorno; county: Portoferraio; locality: San Giovanni; decimalLatitude: 42.801531; decimalLongitude: 10.322325; geodeticDatum: WGS84; coordinatePrecision: 0.0002; **Identification:** identifiedBy: E. Paggetti; **Event:** eventDate: 2009-04-20; **Record Level:** collectionCode: LFPC
- **Type status:** Other material. **Occurrence:** recordedBy: Leonardo Forbicioni; individualCount: 1; lifeStage: adult; occurrenceID: C7DA05B6-C7AC-5553-8031-5E56F4D5E42B; **Taxon:** scientificName: Dicercaaeneaaenea (Linnaeus, 1767); order: Coleoptera; family: Buprestidae; genus: Dicerca; specificEpithet: aenea; infraspecificEpithet: aenea; scientificNameAuthorship: (Linnaeus, 1767); **Location:** islandGroup: Tuscan Archipelago; island: Isola d'Elba; country: Italy; countryCode: IT; stateProvince: Livorno; county: Portoferraio; locality: San Giovanni; decimalLatitude: 42.801817; decimalLongitude: 10.322531; geodeticDatum: WGS84; coordinatePrecision: 0.0002; **Identification:** identifiedBy: E. Paggetti; **Event:** eventDate: 2011-08-23; **Record Level:** collectionCode: LFPC
- **Type status:** Other material. **Occurrence:** recordedBy: Giuliano Frangini; individualCount: 1; lifeStage: adult; occurrenceID: 76D69EF5-2042-55D1-8507-0DD1FF5D4055; **Taxon:** scientificName: Dicercaaeneaaenea (Linnaeus, 1767); order: Coleoptera; family: Buprestidae; genus: Dicerca; specificEpithet: aenea; infraspecificEpithet: aenea; scientificNameAuthorship: (Linnaeus, 1767); **Location:** islandGroup: Tuscan Archipelago; island: Isola d'Elba; country: Italy; countryCode: IT; stateProvince: Livorno; county: Portoferraio; locality: Schiopparello/Le Prade; decimalLatitude: 42.796216; decimalLongitude: 10.345984; geodeticDatum: WGS84; coordinatePrecision: 0.0002; **Identification:** identifiedBy: L. Forbicioni; **Event:** eventDate: 2013-05-29; **Record Level:** collectionCode: LFPC
- **Type status:** Other material. **Occurrence:** recordedBy: Leonardo Forbicioni; individualCount: 1; lifeStage: adult; occurrenceID: 364D8E9D-41EE-5234-A1DE-A4EAFD39A655; **Taxon:** scientificName: Dicercaaeneaaenea (Linnaeus, 1767); order: Coleoptera; family: Buprestidae; genus: Dicerca; specificEpithet: aenea; infraspecificEpithet: aenea; scientificNameAuthorship: (Linnaeus, 1767); **Location:** islandGroup: Tuscan Archipelago; island: Isola d'Elba; country: Italy; countryCode: IT; stateProvince: Livorno; county: Portoferraio; locality: Buraccio; decimalLatitude: 42.779853; decimalLongitude: 10.353521; geodeticDatum: WGS84; coordinatePrecision: 0.0002; **Identification:** identifiedBy: L. Forbicioni; **Event:** eventDate: 2015-05-20; **Record Level:** collectionCode: LFPC
- **Type status:** Other material. **Occurrence:** recordedBy: Leonardo Forbicioni; individualCount: 2; lifeStage: adult; occurrenceID: E8891DAA-7F94-57E4-A30F-B874F959CF05; **Taxon:** scientificName: Dicercaaeneaaenea (Linnaeus, 1767); order: Coleoptera; family: Buprestidae; genus: Dicerca; specificEpithet: aenea; infraspecificEpithet: aenea; scientificNameAuthorship: (Linnaeus, 1767); **Location:** islandGroup: Tuscan Archipelago; island: Isola d'Elba; country: Italy; countryCode: IT; stateProvince: Livorno; county: Portoferraio; locality: Buraccio; decimalLatitude: 42.779447; decimalLongitude: 10.353286; geodeticDatum: WGS84; coordinatePrecision: 0.0002; **Identification:** identifiedBy: L. Forbicioni; **Event:** eventDate: 2013-05-21; **Record Level:** collectionCode: LFPC
- **Type status:** Other material. **Occurrence:** individualCount: 1; lifeStage: adult; occurrenceID: B0D33E54-82C2-5BC9-B576-20D339F4CDF7; **Taxon:** scientificName: Dicercaaeneaaenea (Linnaeus, 1767); order: Coleoptera; family: Buprestidae; genus: Dicerca; specificEpithet: aenea; infraspecificEpithet: aenea; scientificNameAuthorship: (Linnaeus, 1767); **Location:** islandGroup: Tuscan Archipelago; island: Isola d'Elba; country: Italy; countryCode: IT; stateProvince: Livorno; county: Portoferraio; **Record Level:** source: Razzauti A (1921) Contributi alla conoscenza faunistica delle isole toscane III. Coleotteri delle Isole d’Elba, di Capraia e di Gorgona Atti della Società toscana di Scienze naturali residente in Pisa. 33. Memorie, Pisa, 96-122 pp.. https://www.biodiversitylibrary.org/page/35335669. Holdhaus K. (1923) Elenco dei Coleotteri dell’isola d’Elba con studi sul problema della Tirrenide. Memorie della Società entomologica italiana 2: 77‑175. - Curletti G. (1994) I Buprestidi d’Italia. Catalogo geonemico, sinonimico, bibliografico, biologico. Monografie di Natura Bresciana, Ed. Vannini, Brescia, 19.
- **Type status:** Other material. **Occurrence:** individualCount: 1; lifeStage: adult; occurrenceID: CDDFC336-1B2D-529D-A0EA-90DE443D1C89; **Taxon:** scientificName: Dicercaaeneaaenea (Linnaeus, 1767); order: Coleoptera; family: Buprestidae; genus: Dicerca; specificEpithet: aenea; infraspecificEpithet: aenea; scientificNameAuthorship: (Linnaeus, 1767); **Location:** islandGroup: Tuscan Archipelago; island: Isola d'Elba; country: Italy; countryCode: IT; stateProvince: Livorno; county: Marciana; **Record Level:** source: Holdhaus K. (1923) Elenco dei Coleotteri dell’isola d’Elba con studi sul problema della Tirrenide. Memorie della Società entomologica italiana 2: 77‑175. - Curletti G. (1994) I Buprestidi d’Italia. Catalogo geonemico, sinonimico, bibliografico, biologico. Monografie di Natura Bresciana, Ed. Vannini, Brescia, 19.

##### Conservation status

LC

##### Distribution

Recorded for the Tuscan Archipelago (Isola d'Elba) by Holdhaus 1923 and Curletti 1994.

#### Dicerca alni

(Fischer von Waldheim, 1824)

CCAFCFC0-C74B-5DBF-B6B5-257EB8BD0A5B

##### Materials

- **Type status:** Other material. **Occurrence:** recordedBy: Andrea Beltramini; individualCount: 1; lifeStage: adult; occurrenceID: 56723360-2C8F-5C99-988E-C3CDDCEAF196; **Taxon:** scientificName: Dicercaalni (Fischer von Waldheim, 1824); order: Coleoptera; family: Buprestidae; genus: Dicerca; specificEpithet: alni; scientificNameAuthorship: (Fischer von Waldheim, 1824); **Location:** islandGroup: Tuscan Archipelago; island: Isola d'Elba; country: Italy; countryCode: IT; stateProvince: Livorno; county: Marciana; locality: Monte Capanne/Cabinovia; decimalLatitude: 42.785100; decimalLongitude: 10.167000; geodeticDatum: WGS84; coordinatePrecision: 0.0002; **Identification:** identifiedBy: G. Curletti; **Event:** eventDate: 2023-08-08; **Record Level:** collectionCode: LFPC
- **Type status:** Other material. **Occurrence:** recordedBy: Federico Rosso; individualCount: 1; lifeStage: adult; occurrenceID: 1BE461E9-EBDA-5DAF-9C7E-2FE074EA0362; **Taxon:** scientificName: Dicercaalni (Fischer von Waldheim, 1824); order: Coleoptera; family: Buprestidae; genus: Dicerca; specificEpithet: alni; scientificNameAuthorship: (Fischer von Waldheim, 1824); **Location:** islandGroup: Tuscan Archipelago; island: Isola d'Elba; country: Italy; countryCode: IT; stateProvince: Livorno; county: Campo nell'Elba; municipality: Sant'Ilario; locality: Circonvallazione Pietri; decimalLatitude: 42.764511; decimalLongitude: 10.214822; geodeticDatum: WGS84; coordinatePrecision: 0.0002; **Identification:** identifiedBy: G. Curletti; **Event:** eventDate: 2023-04-21; **Record Level:** collectionCode: LFPC

##### Conservation status

NT

#### Latipalpis plana plana

(Olivier, 1790)

CC974D50-0EDD-544D-9E9C-C4F4385074A3

##### Materials

- **Type status:** Other material. **Occurrence:** recordedBy: Leonardo Forbicioni; individualCount: 4; lifeStage: adult; occurrenceID: 6B876BD8-DE9B-5B6F-8A61-F55210993018; **Taxon:** scientificName: Latipalpisplanaplana (A.G.Olivier, 1790); order: Coleoptera; family: Buprestidae; genus: Latipalpis; specificEpithet: plana; infraspecificEpithet: plana; scientificNameAuthorship: (A.G.Olivier, 1790); **Location:** islandGroup: Tuscan Archipelago; island: Isola d'Elba; country: Italy; countryCode: IT; stateProvince: Livorno; county: Rio; municipality: Rio Marina; locality: Cavo; decimalLatitude: 42.859239; decimalLongitude: 10.408321; geodeticDatum: WGS84; coordinatePrecision: 0.0002; **Identification:** identifiedBy: L. Forbicioni; **Event:** eventDate: 2013-05-15; **Record Level:** collectionCode: LFPC
- **Type status:** Other material. **Occurrence:** recordedBy: Leonardo Forbicioni; individualCount: 1; lifeStage: adult; occurrenceID: 1F509726-721C-5D44-9908-23C151B003D6; **Taxon:** scientificName: Latipalpisplanaplana (A.G.Olivier, 1790); order: Coleoptera; family: Buprestidae; genus: Latipalpis; specificEpithet: plana; infraspecificEpithet: plana; scientificNameAuthorship: (A.G.Olivier, 1790); **Location:** islandGroup: Tuscan Archipelago; island: Isola d'Elba; country: Italy; countryCode: IT; stateProvince: Livorno; county: Portoferraio; locality: Colle Reciso/Mulino a vento; decimalLatitude: 42.779815; decimalLongitude: 10.272588; geodeticDatum: WGS84; coordinatePrecision: 0.0002; **Identification:** identifiedBy: L. Forbicioni; **Event:** eventDate: 2011-07-03; **Record Level:** collectionCode: LFPC
- **Type status:** Other material. **Occurrence:** recordedBy: Leonardo Forbicioni; individualCount: 1; lifeStage: adult; occurrenceID: CE88BADB-A7F1-5CD5-9BA5-DD53C52F04D6; **Taxon:** scientificName: Latipalpisplanaplana (A.G.Olivier, 1790); order: Coleoptera; family: Buprestidae; genus: Latipalpis; specificEpithet: plana; infraspecificEpithet: plana; scientificNameAuthorship: (A.G.Olivier, 1790); **Location:** islandGroup: Tuscan Archipelago; island: Isola d'Elba; country: Italy; countryCode: IT; stateProvince: Livorno; county: Portoferraio; locality: Colle Reciso/Mulino a vento; decimalLatitude: 42.776521; decimalLongitude: 10.280773; geodeticDatum: WGS84; coordinatePrecision: 0.0002; **Identification:** identifiedBy: L. Forbicioni; **Event:** eventDate: 2008-06-13; **Record Level:** collectionCode: LFPC
- **Type status:** Other material. **Occurrence:** recordedBy: Maurizio Mei; individualCount: 1; lifeStage: adult; occurrenceID: CF9C2083-FAD1-5D83-9089-89AAB04BE4F3; **Taxon:** scientificName: Latipalpisplanaplana (A.G.Olivier, 1790); order: Coleoptera; family: Buprestidae; genus: Latipalpis; specificEpithet: plana; infraspecificEpithet: plana; scientificNameAuthorship: (A.G.Olivier, 1790); **Location:** islandGroup: Tuscan Archipelago; island: Isola d'Elba; country: Italy; countryCode: IT; stateProvince: Livorno; county: Portoferraio; locality: San Martino; decimalLatitude: 42.781356; decimalLongitude: 10.281645; geodeticDatum: WGS84; coordinatePrecision: 0.0002; **Identification:** identifiedBy: L. Forbicioni; **Event:** eventDate: 2014-08-03; **Record Level:** collectionCode: LFPC
- **Type status:** Other material. **Occurrence:** recordedBy: Leonardo Forbicioni; individualCount: 1; lifeStage: adult; occurrenceID: C5520091-DA5D-555F-A994-94E6BB754352; **Taxon:** scientificName: Latipalpisplanaplana (A.G.Olivier, 1790); order: Coleoptera; family: Buprestidae; genus: Latipalpis; specificEpithet: plana; infraspecificEpithet: plana; scientificNameAuthorship: (A.G.Olivier, 1790); **Location:** islandGroup: Tuscan Archipelago; island: Isola d'Elba; country: Italy; countryCode: IT; stateProvince: Livorno; county: Portoferraio; locality: Acquabona; decimalLatitude: 42.785989; decimalLongitude: 10.346270; geodeticDatum: WGS84; coordinatePrecision: 0.0002; **Identification:** identifiedBy: L. Forbicioni; **Event:** eventDate: 2011-05-26; **Record Level:** collectionCode: LFPC
- **Type status:** Other material. **Occurrence:** recordedBy: Leonardo Forbicioni; individualCount: 1; lifeStage: adult; occurrenceID: 1C7FC2F7-568F-54A8-9130-182FC252BF00; **Taxon:** scientificName: Latipalpisplanaplana (A.G.Olivier, 1790); order: Coleoptera; family: Buprestidae; genus: Latipalpis; specificEpithet: plana; infraspecificEpithet: plana; scientificNameAuthorship: (A.G.Olivier, 1790); **Location:** islandGroup: Tuscan Archipelago; island: Isola d'Elba; country: Italy; countryCode: IT; stateProvince: Livorno; county: Portoferraio; locality: near Carabinieri Station; decimalLatitude: 42.813489; decimalLongitude: 10.316821; geodeticDatum: WGS84; coordinatePrecision: 0.0002; **Identification:** identifiedBy: L. Forbicioni; **Event:** eventDate: 2022-05-27; **Record Level:** collectionCode: LFPC
- **Type status:** Other material. **Occurrence:** recordedBy: Cesare Bellò & Silvana Chemello; individualCount: 1; lifeStage: adult; occurrenceID: 9844C783-4F03-5FA8-AF19-F6BC3BDBD0C9; **Taxon:** scientificName: Latipalpisplanaplana (A.G.Olivier, 1790); order: Coleoptera; family: Buprestidae; genus: Latipalpis; specificEpithet: plana; infraspecificEpithet: plana; scientificNameAuthorship: (A.G.Olivier, 1790); **Location:** islandGroup: Tuscan Archipelago; island: Isola del Giglio; country: Italy; countryCode: IT; stateProvince: Grosseto; county: Campese; locality: Il Franco; decimalLatitude: 42.359742; decimalLongitude: 10.871606; geodeticDatum: WGS84; coordinatePrecision: 0.0002; **Identification:** identifiedBy: L. Forbicioni; **Event:** eventDate: 2019-06-10; **Record Level:** collectionCode: LFPC
- **Type status:** Other material. **Occurrence:** recordedBy: Andrea Beltramini; individualCount: 1; lifeStage: adult; occurrenceID: 990764CF-3F40-51A3-8EA5-A297600927AA; **Taxon:** scientificName: Latipalpisplanaplana (A.G.Olivier, 1790); order: Coleoptera; family: Buprestidae; genus: Latipalpis; specificEpithet: plana; infraspecificEpithet: plana; scientificNameAuthorship: (A.G.Olivier, 1790); **Location:** islandGroup: Tuscan Archipelago; island: Isola d'Elba; country: Italy; countryCode: IT; stateProvince: Livorno; county: Marciana Marina; decimalLatitude: 42.805400; decimalLongitude: 10.176400; geodeticDatum: WGS84; coordinatePrecision: 0.0002; **Identification:** identifiedBy: A. Beltramini; **Event:** eventDate: 2023-07-25; **Record Level:** collectionCode: LFPC
- **Type status:** Other material. **Occurrence:** recordedBy: Sergio Tarsiero; individualCount: 1; lifeStage: adult; occurrenceID: 6E6F115C-C948-57D3-866E-FF2C03DC9D68; **Taxon:** scientificName: Latipalpisplanaplana (A.G.Olivier, 1790); order: Coleoptera; family: Buprestidae; genus: Latipalpis; specificEpithet: plana; infraspecificEpithet: plana; scientificNameAuthorship: (A.G.Olivier, 1790); **Location:** islandGroup: Tuscan Archipelago; island: Isola d'Elba; country: Italy; countryCode: IT; stateProvince: Livorno; county: Rio; municipality: Rio nell'Elba; locality: Spiaggia di Sambroni; decimalLatitude: 42.843668; decimalLongitude: 10.390685; geodeticDatum: WGS84; coordinatePrecision: 0.01; **Identification:** identifiedBy: L. Forbicioni; **Event:** eventDate: 2023-08-15; **Record Level:** source: https://www.inaturalist.org/observations/178584798
- **Type status:** Other material. **Occurrence:** recordedBy: Mattia Diomedi; individualCount: 1; lifeStage: adult; occurrenceID: 718A9501-F145-5AF6-9F05-896317FC2BE4; **Taxon:** scientificName: Latipalpisplanaplana (A.G.Olivier, 1790); order: Coleoptera; family: Buprestidae; genus: Latipalpis; specificEpithet: plana; infraspecificEpithet: plana; scientificNameAuthorship: (A.G.Olivier, 1790); **Location:** islandGroup: Tuscan Archipelago; island: Isola d'Elba; country: Italy; countryCode: IT; stateProvince: Livorno; county: Portoferraio; locality: Capannone; decimalLatitude: 42.794009; decimalLongitude: 10.269974; geodeticDatum: WGS84; coordinatePrecision: 0.001; **Identification:** identifiedBy: L. Forbicioni; **Event:** eventDate: 2023-08-03; **Record Level:** source: https://www.inaturalist.org/observations/177971770
- **Type status:** Other material. **Occurrence:** recordedBy: Riccardo Rocchi; individualCount: 1; lifeStage: adult; occurrenceID: D615C668-0ACE-56F8-B775-AEB0911FAD98; **Taxon:** scientificName: Latipalpisplanaplana (A.G.Olivier, 1790); order: Coleoptera; family: Buprestidae; genus: Latipalpis; specificEpithet: plana; infraspecificEpithet: plana; scientificNameAuthorship: (A.G.Olivier, 1790); **Location:** islandGroup: Tuscan Archipelago; island: Isola d'Elba; country: Italy; countryCode: IT; stateProvince: Livorno; county: Rio; municipality: Rio Marina; locality: Il Porticciolo; decimalLatitude: 42.805774; decimalLongitude: 10.429616; geodeticDatum: WGS84; coordinatePrecision: 0.0001; **Identification:** identifiedBy: L. Forbicioni; **Event:** eventDate: 2023-08-06; **Record Level:** source: https://www.inaturalist.org/observations/176937158
- **Type status:** Other material. **Occurrence:** recordedBy: Simon Habermann; individualCount: 1; lifeStage: adult; occurrenceID: ACB28526-C2C3-5BF7-8711-89DD0F69ABAE; **Taxon:** scientificName: Latipalpisplanaplana (A.G.Olivier, 1790); order: Coleoptera; family: Buprestidae; genus: Latipalpis; specificEpithet: plana; infraspecificEpithet: plana; scientificNameAuthorship: (A.G.Olivier, 1790); **Location:** islandGroup: Tuscan Archipelago; island: Isola d'Elba; country: Italy; countryCode: IT; stateProvince: Livorno; county: Portoferraio; locality: Monte Castello; decimalLatitude: 42.784279; decimalLongitude: 10.380806; geodeticDatum: WGS84; coordinatePrecision: 0.4; **Identification:** identifiedBy: L. Forbicioni; **Event:** eventDate: 2023-05-30; **Record Level:** source: https://www.inaturalist.org/observations/165272109
- **Type status:** Other material. **Occurrence:** individualCount: 1; lifeStage: adult; occurrenceID: DDF527B8-C203-50FE-839A-A7CCA61F3244; **Taxon:** scientificName: Latipalpisplanaplana (A.G.Olivier, 1790); order: Coleoptera; family: Buprestidae; genus: Latipalpis; specificEpithet: plana; infraspecificEpithet: plana; scientificNameAuthorship: (A.G.Olivier, 1790); **Location:** islandGroup: Tuscan Archipelago; island: Isola d'Elba; country: Italy; countryCode: IT; stateProvince: Livorno; county: Capoliveri; locality: Colle Reciso/Mulino a vento; decimalLatitude: 42.778191; decimalLongitude: 10.304082; geodeticDatum: WGS84; coordinatePrecision: 0.01; **Identification:** identifiedBy: L. Forbicioni; **Event:** eventDate: 2022-06-03; **Record Level:** source: https://www.inaturalist.org/observations/120126520
- **Type status:** Other material. **Occurrence:** individualCount: 1; lifeStage: adult; occurrenceID: 07FD2799-3DCA-5542-8AE8-BD58F9FED312; **Taxon:** scientificName: Latipalpisplanaplana (A.G.Olivier, 1790); order: Coleoptera; family: Buprestidae; genus: Latipalpis; specificEpithet: plana; infraspecificEpithet: plana; scientificNameAuthorship: (A.G.Olivier, 1790); **Location:** islandGroup: Tuscan Archipelago; island: Isola d'Elba; country: Italy; countryCode: IT; stateProvince: Livorno; county: Marciana; decimalLatitude: 42.778013; decimalLongitude: 10.192739; geodeticDatum: WGS84; coordinatePrecision: 0.2; **Identification:** identifiedBy: L. Forbicioni; **Event:** eventDate: 2021-07-30; **Record Level:** source: https://www.inaturalist.org/observations/92451893
- **Type status:** Other material. **Occurrence:** recordedBy: Silvia Bracci; individualCount: 1; lifeStage: adult; occurrenceID: E830D8DD-E425-581E-83DB-2A85F718E401; **Taxon:** scientificName: Latipalpisplanaplana (A.G.Olivier, 1790); order: Coleoptera; family: Buprestidae; genus: Latipalpis; specificEpithet: plana; infraspecificEpithet: plana; scientificNameAuthorship: (A.G.Olivier, 1790); **Location:** islandGroup: Tuscan Archipelago; island: Isola d'Elba; country: Italy; countryCode: IT; stateProvince: Livorno; county: Capoliveri; locality: Monte Calamita/Buzzancone; decimalLatitude: 42.743098; decimalLongitude: 10.414740; geodeticDatum: WGS84; coordinatePrecision: 0.001; **Identification:** identifiedBy: L. Forbicioni; **Event:** eventDate: 2021-08-17; **Record Level:** source: https://www.inaturalist.org/observations/91556384
- **Type status:** Other material. **Occurrence:** recordedBy: Libero Lenzi Pari; individualCount: 1; lifeStage: adult; occurrenceID: 575B7AEA-A2C5-5325-B3CB-CE4A56DE6204; **Taxon:** scientificName: Latipalpisplanaplana (A.G.Olivier, 1790); order: Coleoptera; family: Buprestidae; genus: Latipalpis; specificEpithet: plana; infraspecificEpithet: plana; scientificNameAuthorship: (A.G.Olivier, 1790); **Location:** islandGroup: Tuscan Archipelago; island: Isola d'Elba; country: Italy; countryCode: IT; stateProvince: Livorno; county: Rio; municipality: Rio Marina; locality: Il Porticciolo; decimalLatitude: 42.803094; decimalLongitude: 10.431643; geodeticDatum: WGS84; coordinatePrecision: 0.0006; **Identification:** identifiedBy: L. Forbicioni; **Event:** eventDate: 2021-08-01; **Record Level:** source: https://www.inaturalist.org/observations/89785534
- **Type status:** Other material. **Occurrence:** individualCount: 1; lifeStage: adult; occurrenceID: 76A72D95-A762-5ECA-965B-BC22D0FDEEB0; **Taxon:** scientificName: Latipalpisplanaplana (A.G.Olivier, 1790); order: Coleoptera; family: Buprestidae; genus: Latipalpis; specificEpithet: plana; infraspecificEpithet: plana; scientificNameAuthorship: (A.G.Olivier, 1790); **Location:** islandGroup: Tuscan Archipelago; island: Isola d'Elba; country: Italy; countryCode: IT; stateProvince: Livorno; county: Rio; municipality: Rio Marina; locality: Il Porticciolo; decimalLatitude: 42.805177; decimalLongitude: 10.429182; geodeticDatum: WGS84; coordinatePrecision: 0.0002; **Identification:** identifiedBy: L. Forbicioni; **Event:** eventDate: 2021-07-11; **Record Level:** source: https://www.inaturalist.org/observations/86629021
- **Type status:** Other material. **Occurrence:** recordedBy: Leonardo Ancillotto; individualCount: 1; lifeStage: adult; occurrenceID: 296B8B9C-739B-543D-8C8E-77FDF5C8139E; **Taxon:** scientificName: Latipalpisplanaplana (A.G.Olivier, 1790); order: Coleoptera; family: Buprestidae; genus: Latipalpis; specificEpithet: plana; infraspecificEpithet: plana; scientificNameAuthorship: (A.G.Olivier, 1790); **Location:** islandGroup: Tuscan Archipelago; island: Isola d'Elba; country: Italy; countryCode: IT; stateProvince: Livorno; county: Campo nell'Elba; locality: Bonalaccia; decimalLatitude: 42.766667; decimalLongitude: 10.241944; geodeticDatum: WGS84; **Identification:** identifiedBy: L. Forbicioni; **Event:** eventDate: 2021-05-30; **Record Level:** source: https://www.inaturalist.org/observations/80862810
- **Type status:** Other material. **Occurrence:** recordedBy: Luca Odorico; individualCount: 1; lifeStage: adult; occurrenceID: 716E0394-5C34-5D38-8F97-57B533754B05; **Taxon:** scientificName: Latipalpisplanaplana (A.G.Olivier, 1790); order: Coleoptera; family: Buprestidae; genus: Latipalpis; specificEpithet: plana; infraspecificEpithet: plana; scientificNameAuthorship: (A.G.Olivier, 1790); **Location:** islandGroup: Tuscan Archipelago; island: Isola del Giglio; country: Italy; countryCode: IT; stateProvince: Grosseto; county: Giglio Castello; decimalLatitude: 42.364592; decimalLongitude: 10.907709; geodeticDatum: WGS84; coordinatePrecision: 0.0003; **Identification:** identifiedBy: L. Forbicioni; **Event:** eventDate: 2022-08-19; **Record Level:** source: https://www.inaturalist.org/observations/134274528
- **Type status:** Other material. **Occurrence:** recordedBy: Valentin Moser; individualCount: 1; lifeStage: adult; occurrenceID: 6C603784-FF3A-5A82-AD79-0F946E3534C6; **Taxon:** scientificName: Latipalpisplanaplana (A.G.Olivier, 1790); order: Coleoptera; family: Buprestidae; genus: Latipalpis; specificEpithet: plana; infraspecificEpithet: plana; scientificNameAuthorship: (A.G.Olivier, 1790); **Location:** islandGroup: Tuscan Archipelago; island: Isola del Giglio; country: Italy; countryCode: IT; stateProvince: Grosseto; county: Campese; decimalLatitude: 42.365044; decimalLongitude: 10.879649; geodeticDatum: WGS84; coordinatePrecision: 0.003; **Identification:** identifiedBy: L. Forbicioni; **Event:** eventDate: 2021-06-16; **Record Level:** source: https://www.inaturalist.org/observations/85257157
- **Type status:** Other material. **Occurrence:** individualCount: 1; lifeStage: adult; occurrenceID: C5FCCD33-9375-5B7A-98F6-A0E537D62771; **Taxon:** scientificName: Latipalpisplanaplana (A.G.Olivier, 1790); order: Coleoptera; family: Buprestidae; genus: Latipalpis; specificEpithet: plana; infraspecificEpithet: plana; scientificNameAuthorship: (A.G.Olivier, 1790); **Location:** islandGroup: Tuscan Archipelago; island: Isola del Giglio; country: Italy; countryCode: IT; stateProvince: Grosseto; county: Campese; locality: Mezzo Franco; decimalLatitude: 42.359730; decimalLongitude: 10.874366; geodeticDatum: WGS84; coordinatePrecision: 0.004; **Identification:** identifiedBy: L. Forbicioni; **Event:** eventDate: 2021-06-18; **Record Level:** source: https://www.inaturalist.org/observations/3363668
- **Type status:** Other material. **Occurrence:** individualCount: 1; lifeStage: adult; occurrenceID: 7133B138-2BF0-514D-A753-CB24A9B2CCF2; **Taxon:** scientificName: Latipalpisplanaplana (A.G.Olivier, 1790); order: Coleoptera; family: Buprestidae; genus: Latipalpis; specificEpithet: plana; infraspecificEpithet: plana; scientificNameAuthorship: (A.G.Olivier, 1790); **Location:** islandGroup: Tuscan Archipelago; island: Isola del Giglio; country: Italy; countryCode: IT; stateProvince: Grosseto; county: Campese; locality: Punta Faraglione; decimalLatitude: 42.368769; decimalLongitude: 10.868202; geodeticDatum: WGS84; coordinatePrecision: 0.0008; **Identification:** identifiedBy: L. Forbicioni; **Event:** eventDate: 2023-05-27; **Record Level:** source: https://www.inaturalist.org/observations/163999837
- **Type status:** Other material. **Occurrence:** individualCount: 1; lifeStage: adult; occurrenceID: CF56E8B9-5FF7-5A0E-A5B7-308B07548CBA; **Taxon:** scientificName: Latipalpisplanaplana (A.G.Olivier, 1790); order: Coleoptera; family: Buprestidae; genus: Latipalpis; specificEpithet: plana; infraspecificEpithet: plana; scientificNameAuthorship: (A.G.Olivier, 1790); **Location:** islandGroup: Tuscan Archipelago; island: Isola d'Elba; country: Italy; countryCode: IT; stateProvince: Livorno; **Record Level:** source: Curletti G. (1994) I Buprestidi d’Italia. Catalogo geonemico, sinonimico, bibliografico, biologico. Monografie di Natura Bresciana, Ed. Vannini, Brescia, 19.

##### Conservation status

LC

##### Distribution

Recorded for the Tuscan Archipelago (Isola d'Elba) by Curletti (1994).

#### Perotis lugubris lugubris

(Fabricius, 1777)

98BDDA57-246D-50C8-9777-9573BED0D5EB

##### Materials

- **Type status:** Other material. **Occurrence:** recordedBy: Leonardo Forbicioni & Signorini Walter; individualCount: 1; lifeStage: adult; occurrenceID: 17CB1750-2F81-5150-B776-36AD63453C56; **Taxon:** scientificName: Perotislugubrislugubris (Fabricius, 1777); order: Coleoptera; family: Buprestidae; genus: Perotis; specificEpithet: lugubris; infraspecificEpithet: lugubris; scientificNameAuthorship: (Fabricius, 1777); **Location:** islandGroup: Tuscan Archipelago; island: Isola d'Elba; country: Italy; countryCode: IT; stateProvince: Livorno; county: Rio; municipality: Rio Marina; locality: Il Porticciolo; decimalLatitude: 42.802040; decimalLongitude: 10.432103; geodeticDatum: WGS84; coordinatePrecision: 0.0002; **Identification:** identifiedBy: L. Forbicioni; **Event:** eventDate: 2015-06-24; **Record Level:** collectionCode: LFPC
- **Type status:** Other material. **Occurrence:** recordedBy: Roberto Barsaglini; individualCount: 1; lifeStage: adult; occurrenceID: 3820473C-D82D-5ADA-8BF7-CCB6CBC6E925; **Taxon:** scientificName: Perotislugubrislugubris (Fabricius, 1777); order: Coleoptera; family: Buprestidae; genus: Perotis; specificEpithet: lugubris; infraspecificEpithet: lugubris; scientificNameAuthorship: (Fabricius, 1777); **Location:** islandGroup: Tuscan Archipelago; island: Isola d'Elba; country: Italy; countryCode: IT; stateProvince: Livorno; county: Marciana Marina; locality: La Cala; decimalLatitude: 42.805279; decimalLongitude: 10.169001; geodeticDatum: WGS84; coordinatePrecision: 0.0002; **Identification:** identifiedBy: L. Forbicioni; **Event:** eventDate: 2023-06-10; **Record Level:** collectionCode: LFPC
- **Type status:** Other material. **Occurrence:** recordedBy: Andrea Beltramini; individualCount: 1; lifeStage: adult; occurrenceID: 2FF74DAA-A992-5806-9EE4-AC84CAF836AC; **Taxon:** scientificName: Perotislugubrislugubris (Fabricius, 1777); order: Coleoptera; family: Buprestidae; genus: Perotis; specificEpithet: lugubris; infraspecificEpithet: lugubris; scientificNameAuthorship: (Fabricius, 1777); **Location:** islandGroup: Tuscan Archipelago; island: Isola d'Elba; country: Italy; countryCode: IT; stateProvince: Livorno; county: Rio; municipality: Rio Marina; locality: Capo d'Arco; decimalLatitude: 42.780763; decimalLongitude: 10.424723; geodeticDatum: WGS84; coordinatePrecision: 0.0002; **Identification:** identifiedBy: L. Forbicioni; **Event:** eventDate: 2023-06-26; **Record Level:** collectionCode: LFPC
- **Type status:** Other material. **Occurrence:** recordedBy: Leonardo Forbicioni; individualCount: 1; lifeStage: adult; occurrenceID: 517943CE-9957-59E6-AC1F-38292CE588D5; **Taxon:** scientificName: Perotislugubrislugubris (Fabricius, 1777); order: Coleoptera; family: Buprestidae; genus: Perotis; specificEpithet: lugubris; infraspecificEpithet: lugubris; scientificNameAuthorship: (Fabricius, 1777); **Location:** islandGroup: Tuscan Archipelago; island: Isola d'Elba; country: Italy; countryCode: IT; stateProvince: Livorno; county: Portoferraio; locality: Centro abitato (Via Roma); decimalLatitude: 42.815418; decimalLongitude: 10.331894; geodeticDatum: WGS84; coordinatePrecision: 0.0002; **Identification:** identifiedBy: L. Forbicioni; **Event:** eventDate: 1922-05-19; **Record Level:** collectionCode: LFPC
- **Type status:** Other material. **Occurrence:** recordedBy: Andrea Beltramini; individualCount: 1; lifeStage: adult; occurrenceID: 7D59AA2B-324C-5BF5-B3C8-630A06635EED; **Taxon:** scientificName: Perotislugubrislugubris (Fabricius, 1777); order: Coleoptera; family: Buprestidae; genus: Perotis; specificEpithet: lugubris; infraspecificEpithet: lugubris; scientificNameAuthorship: (Fabricius, 1777); **Location:** islandGroup: Tuscan Archipelago; island: Isola d'Elba; country: Italy; countryCode: IT; stateProvince: Livorno; county: Portoferraio; locality: La Biodola; decimalLatitude: 42.802182; decimalLongitude: 10.272329; geodeticDatum: WGS84; coordinatePrecision: 0.0002; **Identification:** identifiedBy: L. Forbicioni; **Event:** eventDate: 2023-07-23; **Record Level:** collectionCode: LFPC
- **Type status:** Other material. **Occurrence:** recordedBy: Alberto Inghilesi; individualCount: 1; lifeStage: adult; occurrenceID: D5617C0F-78B2-5EBE-A9BB-A083793ECC4D; **Taxon:** scientificName: Perotislugubrislugubris (Fabricius, 1777); order: Coleoptera; family: Buprestidae; genus: Perotis; specificEpithet: lugubris; infraspecificEpithet: lugubris; scientificNameAuthorship: (Fabricius, 1777); **Location:** islandGroup: Tuscan Archipelago; island: Isola d'Elba; country: Italy; countryCode: IT; stateProvince: Livorno; county: Rio; municipality: Rio nell'Elba; locality: Nisportino; decimalLatitude: 42.832746; decimalLongitude: 10.386199; geodeticDatum: WGS84; coordinatePrecision: 0.001; **Identification:** identifiedBy: L. Forbicioni; **Event:** eventDate: 2023-07-28; **Record Level:** source: https://www.inaturalist.org/observations/175356262

##### Conservation status

LC

#### Polycestinae

52320E0A-1E2C-5144-A480-C0C322F0E038

#### Acmaeoderini

27A5A00A-E5B2-55B2-9933-2509F4FA1092

#### Acmaeodera (Palaeotethya) bipunctata bipunctata

(A. G. Olivier, 1790)

A4A00EF1-23E1-51DC-925B-4346658DD1AE

##### Materials

- **Type status:** Other material. **Occurrence:** individualCount: 1; lifeStage: adult; occurrenceID: 133D4C4E-E6D7-5234-95FB-702EBD61265F; **Taxon:** scientificName: Acmaeodera (Palaeotethya) bipunctata bipunctata (A.G. Olivier, 1790); order: Coleoptera; family: Buprestidae; genus: Acmaeodera; subgenus: Palaeotethya; specificEpithet: bipunctata; infraspecificEpithet: bipunctata; scientificNameAuthorship: (A.G. Olivier, 1790); **Location:** islandGroup: Tuscan Archipelago; island: Isola d'Elba; country: Italy; countryCode: IT; stateProvince: Livorno; county: Rio; municipality: Rio Marina; locality: Ortano; **Identification:** identifiedBy: G. Curletti; **Record Level:** source: Curletti G. (1994) I Buprestidi d’Italia. Catalogo geonemico, sinonimico, bibliografico, biologico. Monografie di Natura Bresciana, Ed. Vannini, Brescia, 19.
- **Type status:** Other material. **Occurrence:** individualCount: 1; lifeStage: adult; occurrenceID: 93D93E29-6463-585E-90DE-8AFBF70A2852; **Taxon:** scientificName: Acmaeodera (Palaeotethya) bipunctata bipunctata (A.G. Olivier, 1790); order: Coleoptera; family: Buprestidae; genus: Acmaeodera; subgenus: Palaeotethya; specificEpithet: bipunctata; infraspecificEpithet: bipunctata; scientificNameAuthorship: (A.G. Olivier, 1790); **Location:** islandGroup: Tuscan Archipelago; island: Isola d'Elba; country: Italy; countryCode: IT; stateProvince: Livorno; county: Porto Azzurro; **Identification:** identifiedBy: G. Curletti; **Record Level:** source: Curletti G. (1994) I Buprestidi d’Italia. Catalogo geonemico, sinonimico, bibliografico, biologico. Monografie di Natura Bresciana, Ed. Vannini, Brescia, 19.

##### Distribution

Recorded for the Tuscan Archipelago (Isola d'Elba) by Curletti (1994).

#### Acmaeoderella (Acmaeoderella) discoida

(Fabricius, 1787)

47111544-066F-5A12-B982-1D4065625206

##### Materials

- **Type status:** Other material. **Occurrence:** recordedBy: Leonardo Forbicioni; individualCount: 4; lifeStage: adult; occurrenceID: 494E6E10-4C46-5E9C-BE34-3F44D20E16D9; **Taxon:** scientificName: Acmaeoderella (Acmaeoderella) discoida (Fabricius, 1787); order: Coleoptera; family: Buprestidae; genus: Acmaeoderella; subgenus: Acmaeoderella; specificEpithet: discoidea; scientificNameAuthorship: (Fabricius, 1787); **Location:** islandGroup: Tuscan Archipelago; island: Isola d'Elba; country: Italy; countryCode: IT; stateProvince: Livorno; county: Rio; municipality: Rio Marina; locality: Capo d'Arco; decimalLatitude: 42.779278; decimalLongitude: 10.424782; geodeticDatum: WGS84; coordinatePrecision: 0.0002; **Identification:** identifiedBy: E. Paggetti; **Event:** eventDate: 2012-04-25; **Record Level:** collectionCode: LFPC
- **Type status:** Other material. **Occurrence:** recordedBy: Leonardo Forbicioni; individualCount: 3; lifeStage: adult; occurrenceID: 797FFC51-0806-59CC-B0F1-7B4E4A7D4199; **Taxon:** scientificName: Acmaeoderella (Acmaeoderella) discoida (Fabricius, 1787); order: Coleoptera; family: Buprestidae; genus: Acmaeoderella; subgenus: Acmaeoderella; specificEpithet: discoidea; scientificNameAuthorship: (Fabricius, 1787); **Location:** islandGroup: Tuscan Archipelago; island: Isola d'Elba; country: Italy; countryCode: IT; stateProvince: Livorno; county: Portoferraio; locality: Monte Orello; decimalLatitude: 42.779651; decimalLongitude: 10.333145; geodeticDatum: WGS84; coordinatePrecision: 0.0002; **Identification:** identifiedBy: E. Paggetti; **Event:** eventDate: 2011-04-23; **Record Level:** collectionCode: LFPC
- **Type status:** Other material. **Occurrence:** recordedBy: Leonardo Forbicioni; individualCount: 9; lifeStage: adult; occurrenceID: 04C230D1-ABCF-5228-B5AE-C4ED37C479A6; **Taxon:** scientificName: Acmaeoderella (Acmaeoderella) discoida (Fabricius, 1787); order: Coleoptera; family: Buprestidae; genus: Acmaeoderella; subgenus: Acmaeoderella; specificEpithet: discoidea; scientificNameAuthorship: (Fabricius, 1787); **Location:** islandGroup: Tuscan Archipelago; island: Isola d'Elba; country: Italy; countryCode: IT; stateProvince: Livorno; county: Rio; municipality: Rio Marina; locality: Capo d'Arco; decimalLatitude: 42.778889; decimalLongitude: 10.424722; geodeticDatum: WGS84; coordinatePrecision: 0.0002; **Identification:** identifiedBy: E. Paggetti; **Event:** eventDate: 2014-05-05; **Record Level:** collectionCode: LFPC
- **Type status:** Other material. **Occurrence:** recordedBy: Leonardo Forbicioni; individualCount: 1; lifeStage: adult; occurrenceID: 54191222-4BE6-5922-BEC6-332D343D5EF4; **Taxon:** scientificName: Acmaeoderella (Acmaeoderella) discoida (Fabricius, 1787); order: Coleoptera; family: Buprestidae; genus: Acmaeoderella; subgenus: Acmaeoderella; specificEpithet: discoidea; scientificNameAuthorship: (Fabricius, 1787); **Location:** islandGroup: Tuscan Archipelago; island: Isola d'Elba; country: Italy; countryCode: IT; stateProvince: Livorno; county: Rio; municipality: Rio Marina; locality: Capo d'Arco; decimalLatitude: 42.778889; decimalLongitude: 10.424722; geodeticDatum: WGS84; coordinatePrecision: 0.0002; **Identification:** identifiedBy: E. Paggetti; **Event:** eventDate: 2014-06-06; **Record Level:** collectionCode: LFPC
- **Type status:** Other material. **Occurrence:** individualCount: 1; lifeStage: adult; occurrenceID: 2E525AE2-63D6-5E4A-A376-18D1D140E1EE; **Taxon:** scientificName: Acmaeoderella (Acmaeoderella) discoida (Fabricius, 1787); order: Coleoptera; family: Buprestidae; genus: Acmaeoderella; subgenus: Acmaeoderella; specificEpithet: discoidea; scientificNameAuthorship: (Fabricius, 1787); **Location:** islandGroup: Tuscan Archipelago; island: Isola d'Elba; country: Italy; countryCode: IT; stateProvince: Livorno; **Identification:** identifiedBy: G. Curletti; **Record Level:** source: Curletti G. (1994) I Buprestidi d’Italia. Catalogo geonemico, sinonimico, bibliografico, biologico. Monografie di Natura Bresciana, Ed. Vannini, Brescia, 19.
- **Type status:** Other material. **Occurrence:** individualCount: 1; lifeStage: adult; occurrenceID: 469A20AE-F2F5-5EDB-9D57-0971B716B1C2; **Taxon:** scientificName: Acmaeoderella (Acmaeoderella) discoida (Fabricius, 1787); order: Coleoptera; family: Buprestidae; genus: Acmaeoderella; subgenus: Acmaeoderella; specificEpithet: discoidea; scientificNameAuthorship: (Fabricius, 1787); **Location:** islandGroup: Tuscan Archipelago; island: Isola d'Elba; country: Italy; countryCode: IT; stateProvince: Livorno; **Record Level:** source: Porta, 1929 - Fauna Coleopterorum Italica Vol III Diversicornia (p.397)

##### Distribution

Recorded for the Tuscan Archipelago (Isola d'Elba) by Holdhaus (1923).

#### Acmaeoderella (Carininota) flavofasciata flavofasciata

(Piller & Mitterpacher, 1783)

822A5BA8-2AA9-5A1C-A968-E92E39CBF1B2

##### Materials

- **Type status:** Other material. **Occurrence:** recordedBy: Leonardo Forbicioni; individualCount: 2; lifeStage: adult; occurrenceID: 260742D0-56ED-5113-9716-D51171792195; **Taxon:** scientificName: Acmaeoderella (Carininota) flavofasciata flavofasciata (Piller & Mitterpacher, 1783); order: Coleoptera; family: Buprestidae; genus: Acmaeoderella; subgenus: Carininota; specificEpithet: flavofasciata; infraspecificEpithet: flavofasciata; scientificNameAuthorship: (Piller & Mitterpacher, 1783); **Location:** islandGroup: Tuscan Archipelago; island: Isola d'Elba; country: Italy; countryCode: IT; stateProvince: Livorno; county: Porto Azzurro; locality: Buraccio; decimalLatitude: 42.780000; decimalLongitude: 10.354722; geodeticDatum: WGS84; coordinatePrecision: 0.0002; **Identification:** identifiedBy: L. Forbicioni; **Event:** eventDate: 2017-06-30; **Record Level:** collectionCode: LFPC
- **Type status:** Other material. **Occurrence:** recordedBy: Andrea Pulvirenti; individualCount: 1; lifeStage: adult; occurrenceID: E44E7E41-93F8-5A3F-BAFD-6A8045D293AB; **Taxon:** scientificName: Acmaeoderella (Carininota) flavofasciata flavofasciata (Piller & Mitterpacher, 1783); order: Coleoptera; family: Buprestidae; genus: Acmaeoderella; subgenus: Carininota; specificEpithet: flavofasciata; infraspecificEpithet: flavofasciata; scientificNameAuthorship: (Piller & Mitterpacher, 1783); **Location:** islandGroup: Tuscan Archipelago; island: Isola d'Elba; country: Italy; countryCode: IT; stateProvince: Livorno; county: Porto Azzurro; locality: Monserrato; decimalLatitude: 42.783903; decimalLongitude: 10.392231; geodeticDatum: WGS84; coordinatePrecision: 0.0002; **Identification:** identifiedBy: L. Forbicioni; **Event:** eventDate: 2015-07-15; **Record Level:** collectionCode: LFPC
- **Type status:** Other material. **Occurrence:** recordedBy: Leonardo Forbicioni; individualCount: 1; lifeStage: adult; occurrenceID: 108218FD-8695-526C-8F96-6926850EE263; **Taxon:** scientificName: Acmaeoderella (Carininota) flavofasciata flavofasciata (Piller & Mitterpacher, 1783); order: Coleoptera; family: Buprestidae; genus: Acmaeoderella; subgenus: Carininota; specificEpithet: flavofasciata; infraspecificEpithet: flavofasciata; scientificNameAuthorship: (Piller & Mitterpacher, 1783); **Location:** islandGroup: Tuscan Archipelago; island: Isola d'Elba; country: Italy; countryCode: IT; stateProvince: Livorno; county: Porto Azzurro; locality: Buraccio; decimalLatitude: 42.780320; decimalLongitude: 10.351617; geodeticDatum: WGS84; coordinatePrecision: 0.0002; **Identification:** identifiedBy: L. Forbicioni; **Event:** eventDate: 2021-06-21; **Record Level:** collectionCode: LFPC
- **Type status:** Other material. **Occurrence:** recordedBy: Leonardo Forbicioni; individualCount: 1; lifeStage: adult; occurrenceID: 6BF1D8D9-0CC2-5E96-ACF0-8B8A93085DE2; **Taxon:** scientificName: Acmaeoderella (Carininota) flavofasciata flavofasciata (Piller & Mitterpacher, 1783); order: Coleoptera; family: Buprestidae; genus: Acmaeoderella; subgenus: Carininota; specificEpithet: flavofasciata; infraspecificEpithet: flavofasciata; scientificNameAuthorship: (Piller & Mitterpacher, 1783); **Location:** islandGroup: Tuscan Archipelago; island: Isola d'Elba; country: Italy; countryCode: IT; stateProvince: Livorno; county: Porto Azzurro; locality: Buraccio; decimalLatitude: 42.780075; decimalLongitude: 10.354652; geodeticDatum: WGS84; coordinatePrecision: 0.0002; **Identification:** identifiedBy: L. Forbicioni; **Event:** eventDate: 2021-07-04; **Record Level:** collectionCode: LFPC
- **Type status:** Other material. **Occurrence:** recordedBy: Leonardo Forbicioni; individualCount: 1; lifeStage: adult; occurrenceID: 0615890B-A4E3-5DAE-A42C-58E2A7FCBC10; **Taxon:** scientificName: Acmaeoderella (Carininota) flavofasciata flavofasciata (Piller & Mitterpacher, 1783); order: Coleoptera; family: Buprestidae; genus: Acmaeoderella; subgenus: Carininota; specificEpithet: flavofasciata; infraspecificEpithet: flavofasciata; scientificNameAuthorship: (Piller & Mitterpacher, 1783); **Location:** islandGroup: Tuscan Archipelago; island: Isola d'Elba; country: Italy; countryCode: IT; stateProvince: Livorno; county: Porto Azzurro; locality: Buraccio; decimalLatitude: 42.780558; decimalLongitude: 10.356137; geodeticDatum: WGS84; coordinatePrecision: 0.0002; **Identification:** identifiedBy: L. Forbicioni; **Event:** eventDate: 2017-06-02; **Record Level:** collectionCode: LFPC
- **Type status:** Other material. **Occurrence:** recordedBy: Leonardo Forbicioni; individualCount: 1; lifeStage: adult; occurrenceID: 04C7205B-85A1-5338-9562-EDA675E209D0; **Taxon:** scientificName: Acmaeoderella (Carininota) flavofasciata flavofasciata (Piller & Mitterpacher, 1783); order: Coleoptera; family: Buprestidae; genus: Acmaeoderella; subgenus: Carininota; specificEpithet: flavofasciata; infraspecificEpithet: flavofasciata; scientificNameAuthorship: (Piller & Mitterpacher, 1783); **Location:** islandGroup: Tuscan Archipelago; island: Isola d'Elba; country: Italy; countryCode: IT; stateProvince: Livorno; county: Porto Azzurro; locality: Buraccio; decimalLatitude: 42.779661; decimalLongitude: 10.356152; geodeticDatum: WGS84; coordinatePrecision: 0.0002; **Identification:** identifiedBy: L. Forbicioni; **Event:** eventDate: 2022-06-13; **Record Level:** collectionCode: LFPC
- **Type status:** Other material. **Occurrence:** recordedBy: Leonardo Forbicioni; individualCount: 1; lifeStage: adult; occurrenceID: 3644685A-B845-5DA7-86F5-D789755AA9D7; **Taxon:** scientificName: Acmaeoderella (Carininota) flavofasciata flavofasciata (Piller & Mitterpacher, 1783); order: Coleoptera; family: Buprestidae; genus: Acmaeoderella; subgenus: Carininota; specificEpithet: flavofasciata; infraspecificEpithet: flavofasciata; scientificNameAuthorship: (Piller & Mitterpacher, 1783); **Location:** islandGroup: Tuscan Archipelago; island: Isola d'Elba; country: Italy; countryCode: IT; stateProvince: Livorno; county: Campo nell'Elba; locality: Monte Tambone; decimalLatitude: 42.756270; decimalLongitude: 10.272057; geodeticDatum: WGS84; coordinatePrecision: 0.0002; **Identification:** identifiedBy: L. Forbicioni; **Event:** eventDate: 2022-06-08; **Record Level:** collectionCode: LFPC
- **Type status:** Other material. **Occurrence:** recordedBy: Giuliano Frangini; individualCount: 1; lifeStage: adult; occurrenceID: C2EB7BAB-808E-5CA3-A5CA-D386D5D518CD; **Taxon:** scientificName: Acmaeoderella (Carininota) flavofasciata flavofasciata (Piller & Mitterpacher, 1783); order: Coleoptera; family: Buprestidae; genus: Acmaeoderella; subgenus: Carininota; specificEpithet: flavofasciata; infraspecificEpithet: flavofasciata; scientificNameAuthorship: (Piller & Mitterpacher, 1783); **Location:** islandGroup: Tuscan Archipelago; island: Isola d'Elba; country: Italy; countryCode: IT; stateProvince: Livorno; county: Porto Azzurro; locality: Monserrato; decimalLatitude: 42.783856; decimalLongitude: 10.392530; geodeticDatum: WGS84; coordinatePrecision: 0.0002; **Identification:** identifiedBy: L. Forbicioni; **Event:** eventDate: 2010-07-09; **Record Level:** collectionCode: LFPC
- **Type status:** Other material. **Occurrence:** recordedBy: Leonardo Forbicioni; individualCount: 1; lifeStage: adult; occurrenceID: B8D14604-C823-52A3-9FB3-5ED0EEFB006F; **Taxon:** scientificName: Acmaeoderella (Carininota) flavofasciata flavofasciata (Piller & Mitterpacher, 1783); order: Coleoptera; family: Buprestidae; genus: Acmaeoderella; subgenus: Carininota; specificEpithet: flavofasciata; infraspecificEpithet: flavofasciata; scientificNameAuthorship: (Piller & Mitterpacher, 1783); **Location:** islandGroup: Tuscan Archipelago; island: Isola d'Elba; country: Italy; countryCode: IT; stateProvince: Livorno; county: Portoferraio; locality: Vecchio Papa; decimalLatitude: 42.784390; decimalLongitude: 10.337895; geodeticDatum: WGS84; coordinatePrecision: 0.0002; **Identification:** identifiedBy: E. Paggetti; **Event:** eventDate: 2012-06-02; **Record Level:** collectionCode: LFPC
- **Type status:** Other material. **Occurrence:** recordedBy: Andrea Beltramini; individualCount: 1; lifeStage: adult; occurrenceID: A4B60C94-512F-52CE-8329-04C98394FF03; **Taxon:** scientificName: Acmaeoderella (Carininota) flavofasciata flavofasciata (Piller & Mitterpacher, 1783); order: Coleoptera; family: Buprestidae; genus: Acmaeoderella; subgenus: Carininota; specificEpithet: flavofasciata; infraspecificEpithet: flavofasciata; scientificNameAuthorship: (Piller & Mitterpacher, 1783); **Location:** islandGroup: Tuscan Archipelago; island: Isola d'Elba; country: Italy; countryCode: IT; stateProvince: Livorno; county: Portoferraio; locality: Volterraio; decimalLatitude: 42.799100; decimalLongitude: 10.380600; geodeticDatum: WGS84; coordinatePrecision: 0.0002; **Identification:** identifiedBy: L. Forbicioni; **Event:** eventDate: 2010-07-09; **Record Level:** collectionCode: ABPC
- **Type status:** Other material. **Occurrence:** individualCount: 1; lifeStage: adult; occurrenceID: 9F99EB44-F9DF-5222-A871-7272820BC59C; **Taxon:** scientificName: Acmaeoderella (Carininota) flavofasciata flavofasciata (Piller & Mitterpacher, 1783); order: Coleoptera; family: Buprestidae; genus: Acmaeoderella; subgenus: Carininota; specificEpithet: flavofasciata; infraspecificEpithet: flavofasciata; scientificNameAuthorship: (Piller & Mitterpacher, 1783); **Location:** islandGroup: Tuscan Archipelago; island: Isola d'Elba; country: Italy; countryCode: IT; stateProvince: Livorno; county: Campo nell'Elba; locality: Marina di Campo; **Identification:** identifiedBy: G. Curletti; **Event:** eventDate: 2010-07-09; **Record Level:** source: Curletti G. (1994) I Buprestidi d’Italia. Catalogo geonemico, sinonimico, bibliografico, biologico. Monografie di Natura Bresciana, Ed. Vannini, Brescia, 19.

##### Conservation status

LC

##### Distribution

Recorded for the Tuscan Archipelago (Isola d'Elba) by Curletti (1994).

#### Acmaeoderella (Liogastria) virgulata

(Illiger, 1803)

045B163F-9C20-5C48-AC64-E07D70814B94

##### Materials

- **Type status:** Other material. **Occurrence:** recordedBy: Leonardo Forbicioni; individualCount: 1; lifeStage: adult; occurrenceID: 2CE4FBA5-9C0D-5C61-A232-7F0D9A9D047E; **Taxon:** scientificName: Acmaeoderella (Liogastria) virgulata (Illiger, 1803); order: Coleoptera; family: Buprestidae; genus: Acmaeoderella; subgenus: Liogastria; specificEpithet: virgulata; scientificNameAuthorship: (Illiger, 1803); **Location:** islandGroup: Tuscan Archipelago; island: Isola d'Elba; country: Italy; countryCode: IT; stateProvince: Livorno; county: Capoliveri; locality: Pian di Mola; decimalLatitude: 42.759262; decimalLongitude: 10.366992; geodeticDatum: WGS84; coordinatePrecision: 0.0002; **Identification:** identifiedBy: E. Paggetti; **Event:** eventDate: 2011-06-26; **Record Level:** collectionCode: LFPC
- **Type status:** Other material. **Occurrence:** recordedBy: Leonardo Forbicioni; individualCount: 1; lifeStage: adult; occurrenceID: 515B8233-05A9-54A9-AD07-1851B33DC96F; **Taxon:** scientificName: Acmaeoderella (Liogastria) virgulata (Illiger, 1803); order: Coleoptera; family: Buprestidae; genus: Acmaeoderella; subgenus: Liogastria; specificEpithet: virgulata; scientificNameAuthorship: (Illiger, 1803); **Location:** islandGroup: Tuscan Archipelago; island: Isola d'Elba; country: Italy; countryCode: IT; stateProvince: Livorno; county: Capoliveri; locality: Norsi; decimalLatitude: 42.769670; decimalLongitude: 10.346037; geodeticDatum: WGS84; coordinatePrecision: 0.0002; **Identification:** identifiedBy: E. Paggetti; **Event:** eventDate: 2011-06-10; **Record Level:** collectionCode: LFPC
- **Type status:** Other material. **Occurrence:** recordedBy: Leonardo Forbicioni; individualCount: 1; lifeStage: adult; occurrenceID: 19C1C091-A24F-5446-A077-E87BE623C934; **Taxon:** scientificName: Acmaeoderella (Liogastria) virgulata (Illiger, 1803); order: Coleoptera; family: Buprestidae; genus: Acmaeoderella; subgenus: Liogastria; specificEpithet: virgulata; scientificNameAuthorship: (Illiger, 1803); **Location:** islandGroup: Tuscan Archipelago; island: Isola d'Elba; country: Italy; countryCode: IT; stateProvince: Livorno; county: Porto Azzurro; locality: Buraccio; decimalLatitude: 42.779982; decimalLongitude: 10.353253; geodeticDatum: WGS84; coordinatePrecision: 0.0002; **Identification:** identifiedBy: L. Forbicioni; **Event:** eventDate: 2021-06-21; **Record Level:** collectionCode: LFPC

#### Acmaeoderella (Omphalothorax) adspersula adspersula

(Illiger, 1803)

CA3E68E1-1A9F-560F-9BF8-D64700557C58

##### Materials

- **Type status:** Other material. **Occurrence:** recordedBy: Leonardo Forbicioni; individualCount: 5; lifeStage: adult; occurrenceID: F250B58E-44E0-5FAA-9B22-FB6E6A88459D; **Taxon:** scientificName: Acmaeoderella (Omphalothorax) adspersula adspersula (Illiger, 1803); order: Coleoptera; family: Buprestidae; genus: Acmaeoderella; subgenus: Omphalothorax; specificEpithet: adspersula; infraspecificEpithet: adspersula; scientificNameAuthorship: (Illiger, 1803); **Location:** islandGroup: Tuscan Archipelago; island: Isola d'Elba; country: Italy; countryCode: IT; stateProvince: Livorno; county: Portoferraio; locality: Volterraio; decimalLatitude: 42.802409; decimalLongitude: 10.388590; geodeticDatum: WGS84; coordinatePrecision: 0.0002; **Identification:** identifiedBy: E. Paggetti; **Event:** eventDate: 2011-06-17; **Record Level:** collectionCode: LFPC
- **Type status:** Other material. **Occurrence:** recordedBy: Leonardo Forbicioni; individualCount: 1; lifeStage: adult; occurrenceID: 75AC8A46-6DB8-5D89-98A5-067D4570416C; **Taxon:** scientificName: Acmaeoderella (Omphalothorax) adspersula adspersula (Illiger, 1803); order: Coleoptera; family: Buprestidae; genus: Acmaeoderella; subgenus: Omphalothorax; specificEpithet: adspersula; infraspecificEpithet: adspersula; scientificNameAuthorship: (Illiger, 1803); **Location:** islandGroup: Tuscan Archipelago; island: Isola d'Elba; country: Italy; countryCode: IT; stateProvince: Livorno; county: Portoferraio; locality: Monte Orello; decimalLatitude: 42.779926; decimalLongitude: 10.332547; geodeticDatum: WGS84; coordinatePrecision: 0.0002; **Identification:** identifiedBy: E. Paggetti; **Event:** eventDate: 2010-07-15; **Record Level:** collectionCode: LFPC
- **Type status:** Other material. **Occurrence:** recordedBy: Leonardo Forbicioni; individualCount: 2; lifeStage: adult; occurrenceID: BA8580DF-450E-57BB-8EBA-68FC0C1EF599; **Taxon:** scientificName: Acmaeoderella (Omphalothorax) adspersula adspersula (Illiger, 1803); order: Coleoptera; family: Buprestidae; genus: Acmaeoderella; subgenus: Omphalothorax; specificEpithet: adspersula; infraspecificEpithet: adspersula; scientificNameAuthorship: (Illiger, 1803); **Location:** islandGroup: Tuscan Archipelago; island: Isola d'Elba; country: Italy; countryCode: IT; stateProvince: Livorno; county: Porto Azzurro; locality: Buraccio/Lo Stipito; decimalLatitude: 42.762166; decimalLongitude: 10.365712; geodeticDatum: WGS84; coordinatePrecision: 0.0002; **Identification:** identifiedBy: E. Paggetti; **Event:** eventDate: 2013-06-07; **Record Level:** collectionCode: LFPC
- **Type status:** Other material. **Occurrence:** recordedBy: Leonardo Forbicioni; individualCount: 2; lifeStage: adult; occurrenceID: 69CB5FBA-7FC6-52DC-AEE2-E49CEE350758; **Taxon:** scientificName: Acmaeoderella (Omphalothorax) adspersula adspersula (Illiger, 1803); order: Coleoptera; family: Buprestidae; genus: Acmaeoderella; subgenus: Omphalothorax; specificEpithet: adspersula; infraspecificEpithet: adspersula; scientificNameAuthorship: (Illiger, 1803); **Location:** islandGroup: Tuscan Archipelago; island: Isola d'Elba; country: Italy; countryCode: IT; stateProvince: Livorno; county: Campo nell'Elba; municipality: San Piero/Sant'Ilario; locality: San Luigi; decimalLatitude: 42.759864; decimalLongitude: 10.211550; geodeticDatum: WGS84; coordinatePrecision: 0.0002; **Identification:** identifiedBy: E. Paggetti; **Event:** eventDate: 2013-07-04; **Record Level:** collectionCode: LFPC
- **Type status:** Other material. **Occurrence:** recordedBy: Leonardo Forbicioni; individualCount: 1; lifeStage: adult; occurrenceID: 76F808B2-EF01-5587-AE32-EC83207A257B; **Taxon:** scientificName: Acmaeoderella (Omphalothorax) adspersula adspersula (Illiger, 1803); order: Coleoptera; family: Buprestidae; genus: Acmaeoderella; subgenus: Omphalothorax; specificEpithet: adspersula; infraspecificEpithet: adspersula; scientificNameAuthorship: (Illiger, 1803); **Location:** islandGroup: Tuscan Archipelago; island: Isola d'Elba; country: Italy; countryCode: IT; stateProvince: Livorno; county: Capoliveri; locality: Monte Calamita; decimalLatitude: 42.725574; decimalLongitude: 10.398945; geodeticDatum: WGS84; coordinatePrecision: 0.0002; **Identification:** identifiedBy: E. Paggetti; **Event:** eventDate: 2010-07-10; **Record Level:** collectionCode: LFPC
- **Type status:** Other material. **Occurrence:** recordedBy: Leonardo Forbicioni; individualCount: 1; lifeStage: adult; occurrenceID: C89F1BE1-55D1-5352-89E9-E9FDE70E06DB; **Taxon:** scientificName: Acmaeoderella (Omphalothorax) adspersula adspersula (Illiger, 1803); order: Coleoptera; family: Buprestidae; genus: Acmaeoderella; subgenus: Omphalothorax; specificEpithet: adspersula; infraspecificEpithet: adspersula; scientificNameAuthorship: (Illiger, 1803); **Location:** islandGroup: Tuscan Archipelago; island: Isola d'Elba; country: Italy; countryCode: IT; stateProvince: Livorno; county: Capoliveri; locality: Monte Calamita; decimalLatitude: 42.725835; decimalLongitude: 10.405555; geodeticDatum: WGS84; coordinatePrecision: 0.0002; **Identification:** identifiedBy: E. Paggetti; **Event:** eventDate: 2017-07-02; **Record Level:** collectionCode: LFPC
- **Type status:** Other material. **Occurrence:** recordedBy: Leonardo Forbicioni; individualCount: 1; lifeStage: adult; occurrenceID: A92155F6-581D-5094-B312-DF28835CD238; **Taxon:** scientificName: Acmaeoderella (Omphalothorax) adspersula adspersula (Illiger, 1803); order: Coleoptera; family: Buprestidae; genus: Acmaeoderella; subgenus: Omphalothorax; specificEpithet: adspersula; infraspecificEpithet: adspersula; scientificNameAuthorship: (Illiger, 1803); **Location:** islandGroup: Tuscan Archipelago; island: Isola d'Elba; country: Italy; countryCode: IT; stateProvince: Livorno; county: Capoliveri; locality: Monte Calamita; decimalLatitude: 42.725593; decimalLongitude: 10.405758; geodeticDatum: WGS84; coordinatePrecision: 0.0002; **Identification:** identifiedBy: E. Paggetti; **Event:** eventDate: 2014-07-13; **Record Level:** collectionCode: LFPC
- **Type status:** Other material. **Occurrence:** recordedBy: Leonardo Forbicioni; individualCount: 1; lifeStage: adult; occurrenceID: EAD43533-7576-5BF1-B95F-65A00CBC5188; **Taxon:** scientificName: Acmaeoderella (Omphalothorax) adspersula adspersula (Illiger, 1803); order: Coleoptera; family: Buprestidae; genus: Acmaeoderella; subgenus: Omphalothorax; specificEpithet: adspersula; infraspecificEpithet: adspersula; scientificNameAuthorship: (Illiger, 1803); **Location:** islandGroup: Tuscan Archipelago; island: Isola d'Elba; country: Italy; countryCode: IT; stateProvince: Livorno; county: Porto Azzurro; locality: Buraccio/Lo Stipito; decimalLatitude: 42.762166; decimalLongitude: 10.365712; geodeticDatum: WGS84; coordinatePrecision: 0.0002; **Identification:** identifiedBy: Leonardo Forbicioni; **Event:** eventDate: 2011-06-30; **Record Level:** collectionCode: LFPC
- **Type status:** Other material. **Occurrence:** individualCount: 1; lifeStage: adult; occurrenceID: B8BC28DC-8A3F-523C-BFD5-FE18191C8444; **Taxon:** scientificName: Acmaeoderella (Omphalothorax) adspersula adspersula (Illiger, 1803); order: Coleoptera; family: Buprestidae; genus: Acmaeoderella; subgenus: Omphalothorax; specificEpithet: adspersula; infraspecificEpithet: adspersula; scientificNameAuthorship: (Illiger, 1803); **Location:** islandGroup: Tuscan Archipelago; island: Isola del Giglio; country: Italy; countryCode: IT; stateProvince: Grosseto; **Identification:** identifiedBy: G. Curletti; **Record Level:** source: Curletti G. (1994) I Buprestidi d’Italia. Catalogo geonemico, sinonimico, bibliografico, biologico. Monografie di Natura Bresciana, Ed. Vannini, Brescia, 19.
- **Type status:** Other material. **Occurrence:** individualCount: 1; lifeStage: adult; occurrenceID: DCDD18ED-B2B6-5B36-8CB3-9E9259B9D9BF; **Taxon:** scientificName: Acmaeoderella (Omphalothorax) adspersula adspersula (Illiger, 1803); order: Coleoptera; family: Buprestidae; genus: Acmaeoderella; subgenus: Omphalothorax; specificEpithet: adspersula; infraspecificEpithet: adspersula; scientificNameAuthorship: (Illiger, 1803); **Location:** islandGroup: Tuscan Archipelago; island: Isola d'Elba; country: Italy; countryCode: IT; stateProvince: Livorno; county: Portoferraio; locality: Enfola; **Identification:** identifiedBy: G. Curletti; **Record Level:** source: Curletti G. (1994) I Buprestidi d’Italia. Catalogo geonemico, sinonimico, bibliografico, biologico. Monografie di Natura Bresciana, Ed. Vannini, Brescia, 19.
- **Type status:** Other material. **Occurrence:** individualCount: 1; lifeStage: adult; occurrenceID: 2A859ADE-B195-59E4-AD47-7EB1031C5B97; **Taxon:** scientificName: Acmaeoderella (Omphalothorax) adspersula adspersula (Illiger, 1803); order: Coleoptera; family: Buprestidae; genus: Acmaeoderella; subgenus: Omphalothorax; specificEpithet: adspersula; infraspecificEpithet: adspersula; scientificNameAuthorship: (Illiger, 1803); **Location:** islandGroup: Tuscan Archipelago; island: Isola d'Elba; country: Italy; countryCode: IT; stateProvince: Livorno; county: Rio; municipality: Rio Marina; locality: Ortano; **Identification:** identifiedBy: G. Curletti; **Record Level:** source: Curletti G. (1994) I Buprestidi d’Italia. Catalogo geonemico, sinonimico, bibliografico, biologico. Monografie di Natura Bresciana, Ed. Vannini, Brescia, 19.
- **Type status:** Other material. **Occurrence:** individualCount: 1; lifeStage: adult; occurrenceID: 0FDBCB2B-67BB-5433-90A6-5DF8A09815E5; **Taxon:** scientificName: Acmaeoderella (Omphalothorax) adspersula adspersula (Illiger, 1803); order: Coleoptera; family: Buprestidae; genus: Acmaeoderella; subgenus: Omphalothorax; specificEpithet: adspersula; infraspecificEpithet: adspersula; scientificNameAuthorship: (Illiger, 1803); **Location:** islandGroup: Tuscan Archipelago; island: Isola d'Elba; country: Italy; countryCode: IT; stateProvince: Livorno; county: Porto Azzurro; **Identification:** identifiedBy: G. Curletti; **Record Level:** source: Curletti G. (1994) I Buprestidi d’Italia. Catalogo geonemico, sinonimico, bibliografico, biologico. Monografie di Natura Bresciana, Ed. Vannini, Brescia, 19.

##### Conservation status

LC

##### Distribution

Recorded for the Tuscan Archipelago (Isola d'Elba and Giglio) by Curletti (1994).

#### Ptosimini

89817350-514D-5384-A4EF-6CB6D36A0173

#### Ptosima undecimmaculata undecimmaculata

(Herbst, 1784)

C6B45C77-0554-5F6F-9F9D-C1BF4C09BC86

##### Materials

- **Type status:** Other material. **Occurrence:** recordedBy: Leonardo Forbicioni; individualCount: 6; lifeStage: adult; occurrenceID: 96F2310F-CF35-5C7C-936D-02BE98483847; **Taxon:** scientificName: Ptosimaundecimmaculataundecimmaculata (Herbst, 1784); order: Coleoptera; family: Buprestidae; genus: Ptosima; specificEpithet: undecimmaculata; infraspecificEpithet: undecimmaculata; scientificNameAuthorship: (Herbst, 1784); **Location:** islandGroup: Tuscan Archipelago; island: Isola d'Elba; country: Italy; countryCode: IT; stateProvince: Livorno; county: Portoferraio; locality: Valdana; decimalLatitude: 42.774144; decimalLongitude: 10.353256; geodeticDatum: WGS84; coordinatePrecision: 0.0002; **Identification:** identifiedBy: L. Forbicioni; **Event:** eventDate: 2012-05-15; **Record Level:** collectionCode: LFPC
- **Type status:** Other material. **Occurrence:** recordedBy: Leonardo Forbicioni; individualCount: 1; lifeStage: adult; occurrenceID: D0B22D61-C201-50C1-A7E3-35B5ACCCB4B6; **Taxon:** scientificName: Ptosimaundecimmaculataundecimmaculata (Herbst, 1784); order: Coleoptera; family: Buprestidae; genus: Ptosima; specificEpithet: undecimmaculata; infraspecificEpithet: undecimmaculata; scientificNameAuthorship: (Herbst, 1784); **Location:** islandGroup: Tuscan Archipelago; island: Isola d'Elba; country: Italy; countryCode: IT; stateProvince: Livorno; county: Capoliveri; locality: Pian di Mola; decimalLatitude: 42.758876; decimalLongitude: 10.365942; geodeticDatum: WGS84; coordinatePrecision: 0.0002; **Identification:** identifiedBy: L. Forbicioni; **Event:** eventDate: 2013-06-06; **Record Level:** collectionCode: LFPC
- **Type status:** Other material. **Occurrence:** recordedBy: Leonardo Forbicioni; individualCount: 4; lifeStage: adult; occurrenceID: 7392B480-6DB0-516E-B699-617612B8677A; **Taxon:** scientificName: Ptosimaundecimmaculataundecimmaculata (Herbst, 1784); order: Coleoptera; family: Buprestidae; genus: Ptosima; specificEpithet: undecimmaculata; infraspecificEpithet: undecimmaculata; scientificNameAuthorship: (Herbst, 1784); **Location:** islandGroup: Tuscan Archipelago; island: Isola d'Elba; country: Italy; countryCode: IT; stateProvince: Livorno; county: Capoliveri; locality: Pian di Mola; decimalLatitude: 42.758851; decimalLongitude: 10.366128; geodeticDatum: WGS84; coordinatePrecision: 0.0002; **Identification:** identifiedBy: L. Forbicioni; **Event:** eventDate: 2013-06-08; **Record Level:** collectionCode: LFPC
- **Type status:** Other material. **Occurrence:** recordedBy: Leonardo Forbicioni; individualCount: 1; lifeStage: adult; occurrenceID: 5A317EB3-A172-513C-8A05-D4D912883C85; **Taxon:** scientificName: Ptosimaundecimmaculataundecimmaculata (Herbst, 1784); order: Coleoptera; family: Buprestidae; genus: Ptosima; specificEpithet: undecimmaculata; infraspecificEpithet: undecimmaculata; scientificNameAuthorship: (Herbst, 1784); **Location:** islandGroup: Tuscan Archipelago; island: Isola d'Elba; country: Italy; countryCode: IT; stateProvince: Livorno; county: Portoferraio; locality: Colle Reciso/La Cavetta; decimalLatitude: 42.785728; decimalLongitude: 10.318795; geodeticDatum: WGS84; coordinatePrecision: 0.0002; **Identification:** identifiedBy: L. Forbicioni; **Event:** eventDate: 2011-06-06; **Record Level:** collectionCode: LFPC
- **Type status:** Other material. **Occurrence:** recordedBy: Leonardo Forbicioni; individualCount: 1; lifeStage: adult; occurrenceID: AE2C82B3-CB92-5471-BE70-5C2D0BF4FEED; **Taxon:** scientificName: Ptosimaundecimmaculataundecimmaculata (Herbst, 1784); order: Coleoptera; family: Buprestidae; genus: Ptosima; specificEpithet: undecimmaculata; infraspecificEpithet: undecimmaculata; scientificNameAuthorship: (Herbst, 1784); **Location:** islandGroup: Tuscan Archipelago; island: Isola d'Elba; country: Italy; countryCode: IT; stateProvince: Livorno; county: Portoferraio; locality: Monte Poppe; decimalLatitude: 42.797959; decimalLongitude: 10.275927; geodeticDatum: WGS84; coordinatePrecision: 0.0002; **Identification:** identifiedBy: L. Forbicioni; **Event:** eventDate: 2011-06-20; **Record Level:** collectionCode: LFPC
- **Type status:** Other material. **Occurrence:** recordedBy: Giuliano Frangini; individualCount: 2; lifeStage: adult; occurrenceID: 023C609B-E6D5-55FA-A67A-EF997687AC75; **Taxon:** scientificName: Ptosimaundecimmaculataundecimmaculata (Herbst, 1784); order: Coleoptera; family: Buprestidae; genus: Ptosima; specificEpithet: undecimmaculata; infraspecificEpithet: undecimmaculata; scientificNameAuthorship: (Herbst, 1784); **Location:** islandGroup: Tuscan Archipelago; island: Isola d'Elba; country: Italy; countryCode: IT; stateProvince: Livorno; county: Portoferraio; locality: San Govanni; decimalLatitude: 42.801695; decimalLongitude: 10.317183; geodeticDatum: WGS84; coordinatePrecision: 0.0002; **Identification:** identifiedBy: L. Forbicioni; **Event:** eventDate: 2010-05-17; **Record Level:** collectionCode: LFPC

##### Conservation status

LC

## Discussion

Prior to the present contribution, only 27 species of Buprestidae were reported from the Tuscan Archipelago and mostly on the basis of single or a few scant specimens (Poggi 1976, Gobbi 1983, Curletti 1994). The present research produced 341 occurrence records (Suppl. material 1); 282 constitute original new records, 49 of which were retrieved from iNaturalist and 233 from collected specimens (430 specimens in total). These observations allowed increasing the species known for the Archipelago from 27 to 51, just slightly under a quarter of all buprestid species in Italy (Figs 1, 2, 3, 4, 5, 6, 7, 8, 9, 10, 11, 12, 13, 14). All the Buprestidae subfamilies present on the Italian territory have their representatives in the Tuscan Archipelago and *Agrilus* Curtis, 1825 (tribe Agrilini) and *Anthaxia* Eschscholtz, 1829 (tribe Anthaxiini) are the genera most represented, with 13 and 10 species, respectively. The island on which the largest number of species are currently documented is Elba (49), followed by Giglio (10), while the remaining islands of the Archipelago have no or few scattered records (Table 1). The local fauna does not include any strictly endemic taxa, except for two Italian endemics, namely *Agilusetruscus* Curletti, 2013 and *Agilusevocatus* Curletti 2021. Concerning larval trophic biology, according to the categories given in Curletti (1994), the 51 species treated are subdivided as follows: two phyllophagous endophyte, five hypogeobiont poephagus endophyte, five epigeobiont poephagus endophyte, two hypogeobiont arboreal xylophagous endophyte, 24 epigeobiont arboreal xylophagous endophyte, five hypogeobiont arboreal xylophagous endophyte and seven epigeobiont arboreal xylophagous endophyte species (Suppl. material 1). The biology of *Agrilusevocatus* Curletti remains still unknown.

Of the 51 species recorded, about 60% are included in the Italian Red List of Saproxylic Beetles (Audisio et al. 2014) and specifically 28 taxa fall in the Least Concern (LC) category, one in the Nearly Threatened (NT) and one taxon, *Eurythyreaquercus* (Herbst, 1780), in the Critically Endangered (CR) category. Of particular importance is the discovery of multiple specimens (fresh remains) of *E.quercus* inside trunks of *Castaneasativa* Mill. on the northern slopes of Monte Perone (Isola d'Elba) (Figs 15, 16). This taxon, with a Pontic-Mediterranean distribution, is generally sporadic and localised in continental Europe and very rarely in Italy, where it is known only for a few historical records and a recent discovery in Piedmont (Curletti 1980, Curletti and Casale 2021). This species usually develops on large branches and trunks of centuries-old Fagaceae of the genus *Quercus* (*Q.ilex* L., *Q.petraea* (Matt.) Liebl., *Q.pubescens* Willd., *Q.robur* L. and *Q.suber* L.) and on *Castaneasativa* Mill. (Hellrigl 1978, Bily 2002), as also confirmed by the present findings. Adults fly during summer, where they rest on the leaves of tree canopies. Mating occurs in the upper canopy and oviposition generally occurs in the afternoon (Helclová 2022). Females lay eggs on naked wood of living trees presenting stressed or dead parts due to lightning, frost or wind damage (Zabransky 1991); in addition, the female may select debarked wood previously attacked by *Cerambyxcerdo* (Linnaeus, 1758), *Trichoferuspallidus* (Olivier, 1790) (Cerambycidae) and *Dermestoidessanguinicollis* (Fabricius, 1787) (Cleridae) for oviposition (Bily 2002, Helclová 2022). Larval development takes two years under optimal conditions and the larvae need trees with large diameter trunks or branches (> 50 cm) to maintain the ideal humidity level (Zabransky 1991). Due to these peculiar ecological exigencies and the substrate required for its reproduction, *E.quercus* is considered a first-degree forest relict that has disappeared in most of its potential range and its presence is currently heavily localised and limited to almost pristine biotopes (Zabransky 1991).

### Final considerations

This research has further confirmed the importance of the Tuscan Archipelago as a Mediterranean biodiversity hotspot and, in particular, how such a restricted and scattered ecosystem can host almost a quarter of all Italian Buprestidae. Currently, Elba is the island with the highest species richness, a condition influenced by the greater sampling effort, but also by its highly diversified flora, a feature that is substantially reduced in most of the other islands of the Archipelago. Therefore, as research continues, we can certainly expect an increase in the number of taxa on smaller islands despite not being as substantial as recorded for Isola d'Elba. We expect in the next few years to be able to sample the smaller islands more extensively, varying the periods and methods of capture to increase the likelihood of finding species whose presence is expected, but which have, so far, remained elusive. The substantial diversity of species found, 60% of which are included in the Red List of Italian saproxylic beetles, further enhances the Tuscan Archipelago as a national biodiversity repository and an important core for the conservation of localised, rare or declining species. The conservation of these taxa obviously requires appropriate management and conservation plans, especially for private areas and those not included in the Tuscan Archipelago National Park; in fact, for the most endangered species, the existence of key forest elements where wise management is applied is crucial. In addition, although there are no strictly endemic elements, the protection of the local fauna from non-native species or non-native populations is fundamental to the preservation of the Archipelago's specific and genetic heritage. In this regard, better regulation and control of naval traffic and the transduction of wood material, drivers already widely recognised as the main ones for the introduction of non-native and invasive elements, would prevent or at least reduce the risk of introducing potentially harmful taxa to local biodiversity.

## Supplementary Material

XML Treatment for Agrilinae

XML Treatment for Agrilini

XML Treatment for Agrilus angustulus angustulus

XML Treatment for Agrilus cuprescens

XML Treatment for Agrilus cyanescens

XML Treatment for Agrilus derasofasciatus

XML Treatment for Agrilus elegans elegans

XML Treatment for Agrilus etruscus

XML Treatment for Agrilus evocatus

XML Treatment for Agrilus hyperici

XML Treatment for Agrilus integerrimus

XML Treatment for Agrilus laticornis

XML Treatment for Agrilus marozzinii

XML Treatment for Agrilus roscidus

XML Treatment for Agrilus solieri solieri

XML Treatment for Agrilus viridicaerulans rubi

XML Treatment for Aphanisticini

XML Treatment for Aphanisticus elongatus elongatus

XML Treatment for Aphanisticus pygmaeus

XML Treatment for Coraebini

XML Treatment for Coraebus elatus elatus

XML Treatment for Coraebus fasciatus

XML Treatment for Coraebus rubi

XML Treatment for Meliboeus (Meliboeoides) parvulus

XML Treatment for Meliboeus (Meliboeus) fulgidicollis

XML Treatment for Meliboeus (Meliboeus) gibbicollis gibbicollis

XML Treatment for Meliboeus (Meliboeus) graminis graminis

XML Treatment for Tracheini

XML Treatment for Trachys puncticollis rectilineatus

XML Treatment for Trachys troglodytiformis

XML Treatment for Buprestinae

XML Treatment for Athaxiini

XML Treatment for Anthaxia (Anthaxia) chevrieri

XML Treatment for Anthaxia (Anthaxia) mendizabali

XML Treatment for Anthaxia (Anthaxia) thalassophila thalassophila

XML Treatment for Anthaxia (Haplanthaxia) croesus

XML Treatment for Anthaxia (Haplanthaxia) millefolii polychloros

XML Treatment for Anthaxia (Haplanthaxia) umbellatarum umbellatarum

XML Treatment for Anthaxia (Melanthaxia) nigritula nigritula

XML Treatment for Buprestini

XML Treatment for Buprestis (Ancylocheira) novemmaculata novemmaculata

XML Treatment for Eurythyrea micans

XML Treatment for Eurythyrea quercus

XML Treatment for Chrysobothrini

XML Treatment for Chrysobothris (Chrysobothris) affinis affinis

XML Treatment for Chrysobothris (Chrysobothris) solieri

XML Treatment for Chrysochroinae

XML Treatment for Chalchophorini

XML Treatment for Chalcophora massiliensis

XML Treatment for Dicercini

XML Treatment for Capnodis cariosa cariosa

XML Treatment for Capnodis tenebricosa tenebricosa

XML Treatment for Capnodis tenebrionis

XML Treatment for Dicerca aenea aenea

XML Treatment for Dicerca alni

XML Treatment for Latipalpis plana plana

XML Treatment for Perotis lugubris lugubris

XML Treatment for Polycestinae

XML Treatment for Acmaeoderini

XML Treatment for Acmaeodera (Palaeotethya) bipunctata bipunctata

XML Treatment for Acmaeoderella (Acmaeoderella) discoida

XML Treatment for Acmaeoderella (Carininota) flavofasciata flavofasciata

XML Treatment for Acmaeoderella (Liogastria) virgulata

XML Treatment for Acmaeoderella (Omphalothorax) adspersula adspersula

XML Treatment for Ptosimini

XML Treatment for Ptosima undecimmaculata undecimmaculata

17DFDA8B-2D25-5B54-8358-91D1202DF457 10.3897/BDJ.12.e117362.suppl1 Supplementary material 1 Buprestidae of the Tuscan Archipelago Data type occurrences and biological notes. File: oo_960340.xlsx https://binary.pensoft.net/file/960340 L. Forbicioni, G. Curletti, E. Ruzzier

## Figures and Tables

**Figure 1a.**
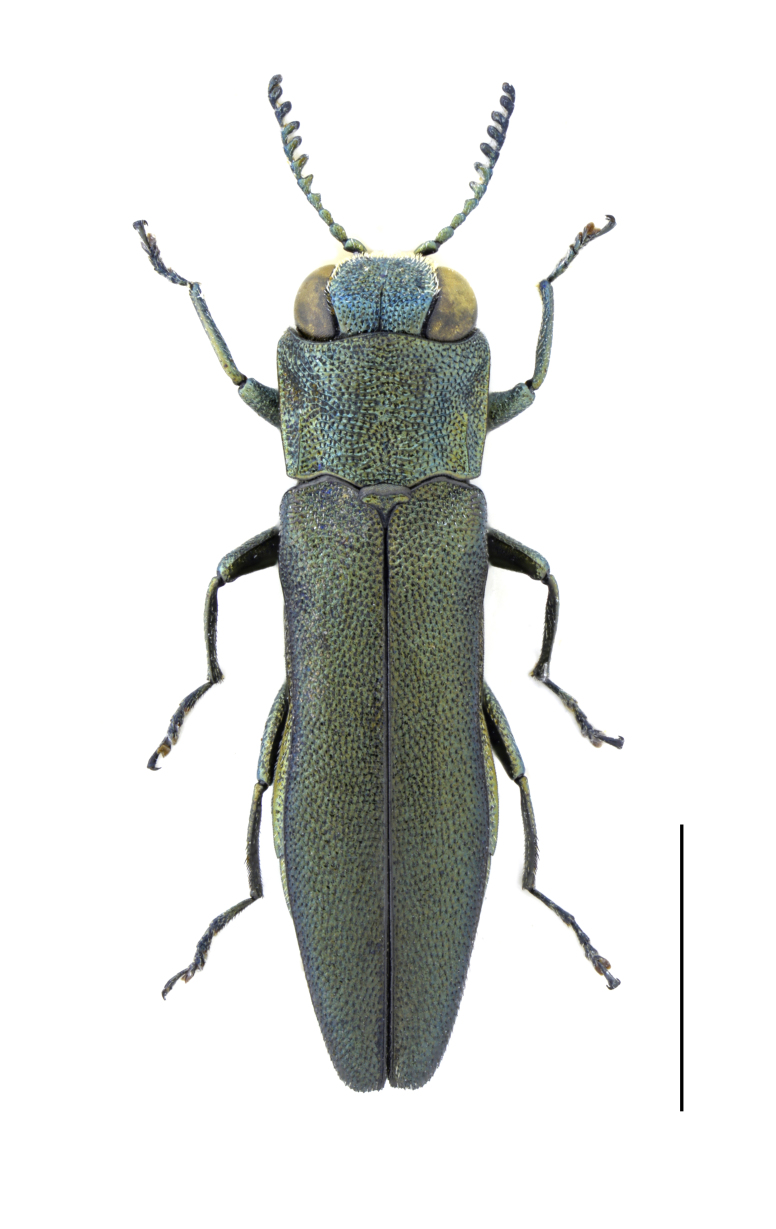
Agrilusangustulusssp.angustulus (Illiger, 1803) - scale bar: 2.0 mm;

**Figure 1b.**
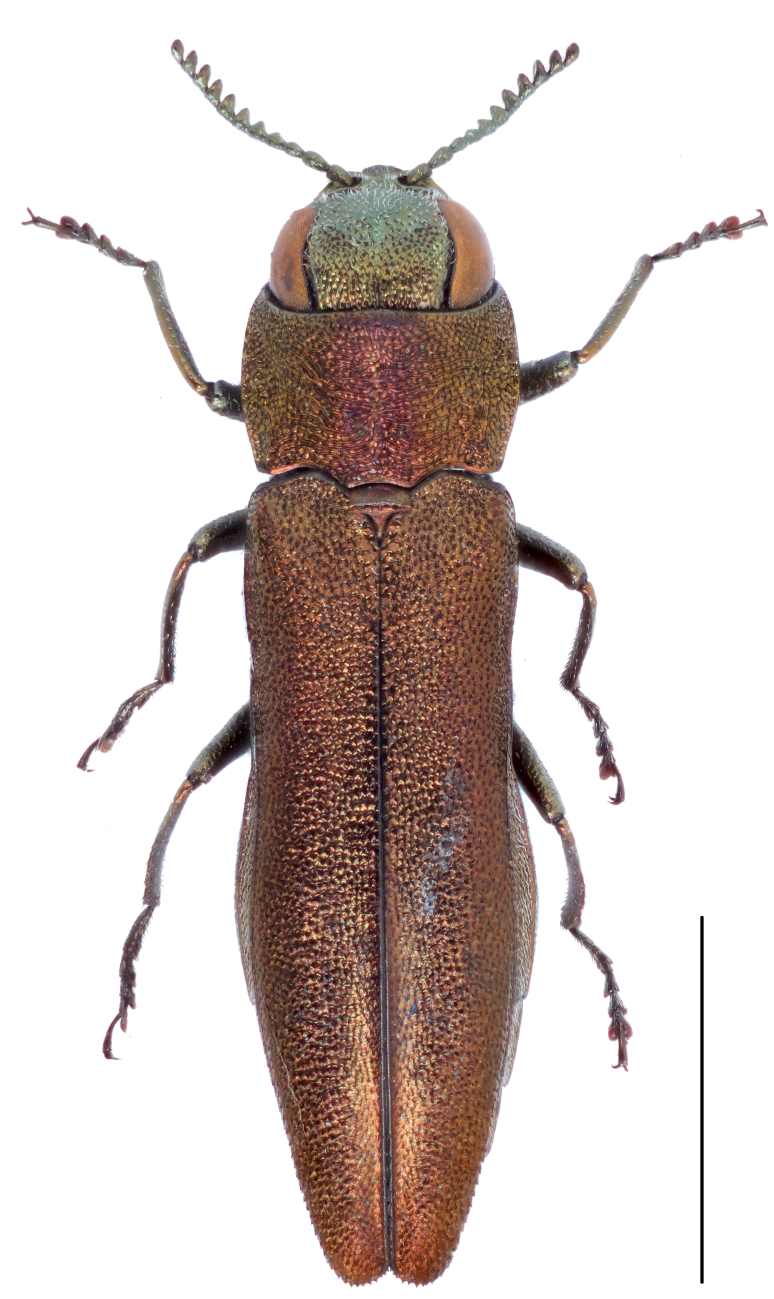
*Agriluscuprescens* (Ménétriés, 1832) - scale bar: 2.0 mm;

**Figure 1c.**
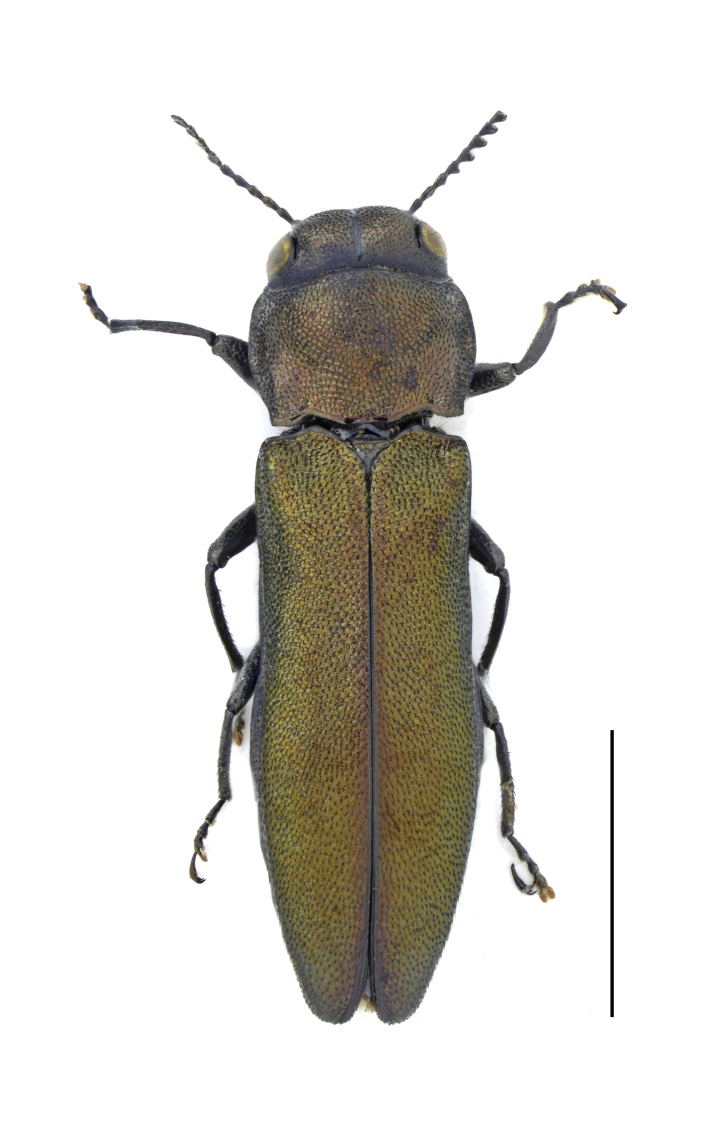
*Agriluscyanescens* (Ratzeburg, 1837) - scale bar: 2.0 mm;

**Figure 1d.**
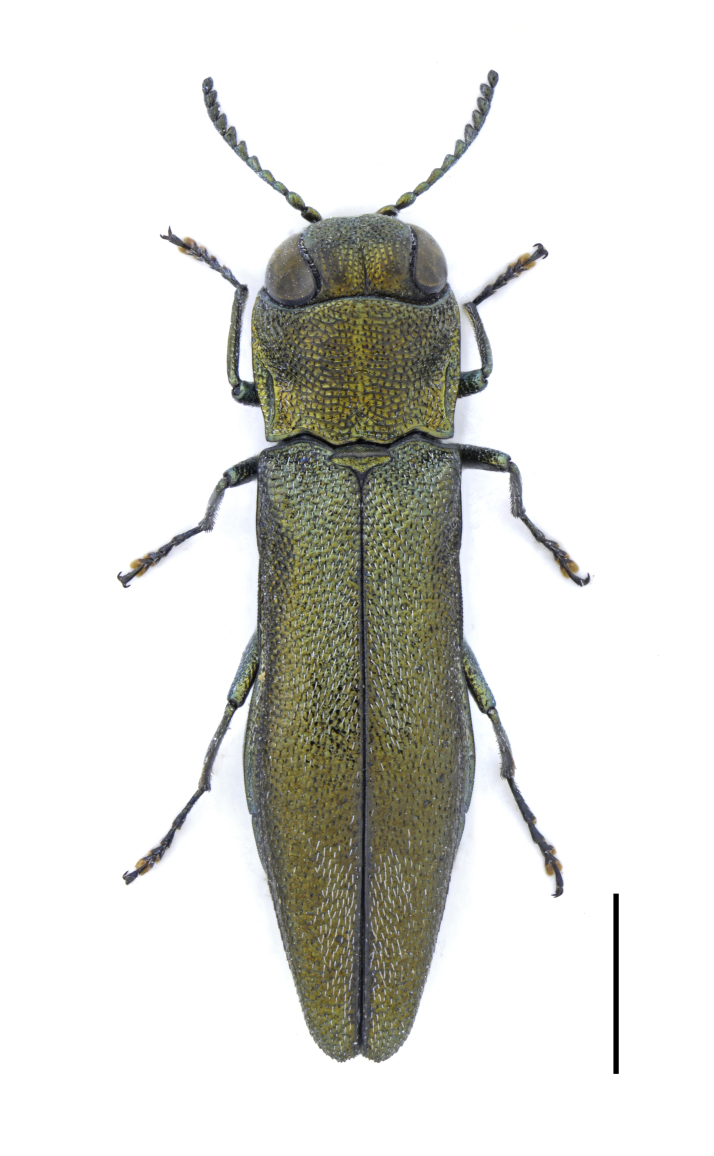
*Agrilusderasofasciatus* Lacordaire, 1835 - scale bar: 1.0 mm.

**Figure 2a.**
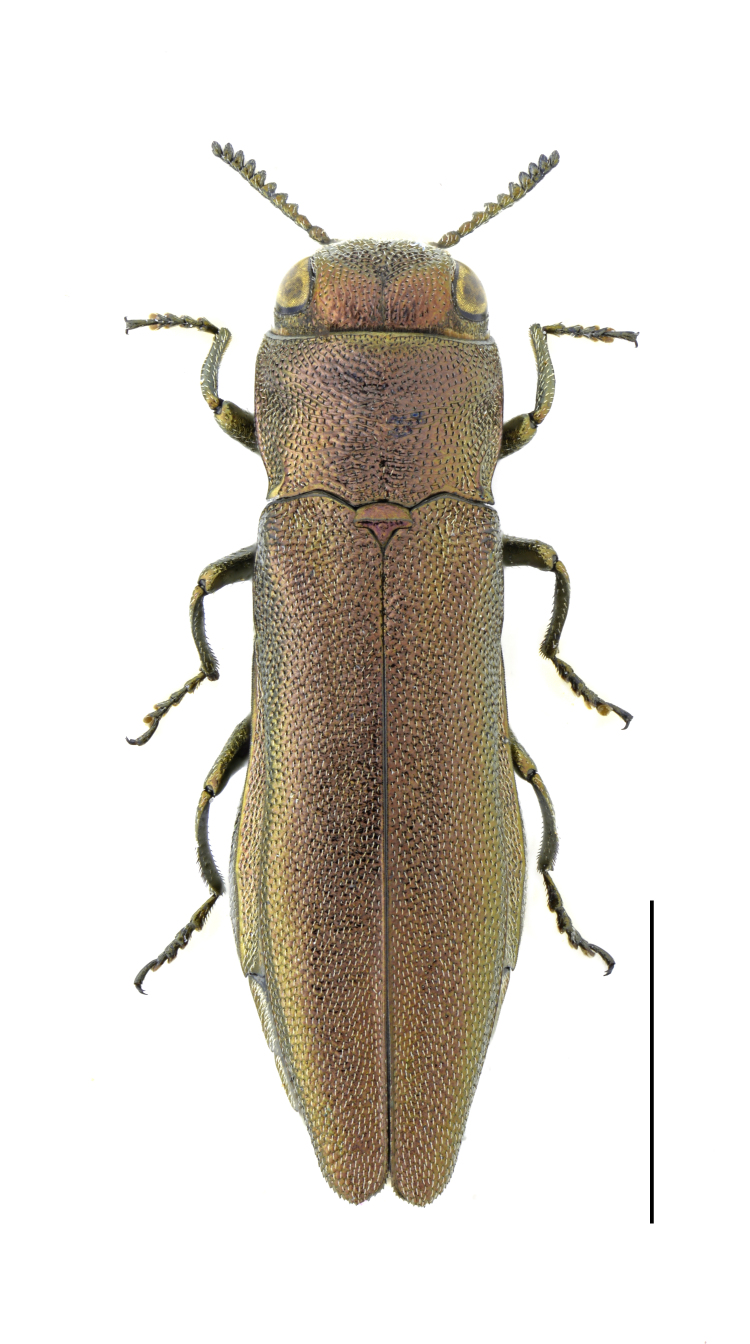
Agriluselegansssp.elegans Mulsant & Rey, 1863 - scale bar: 2.0 mm;

**Figure 2b.**
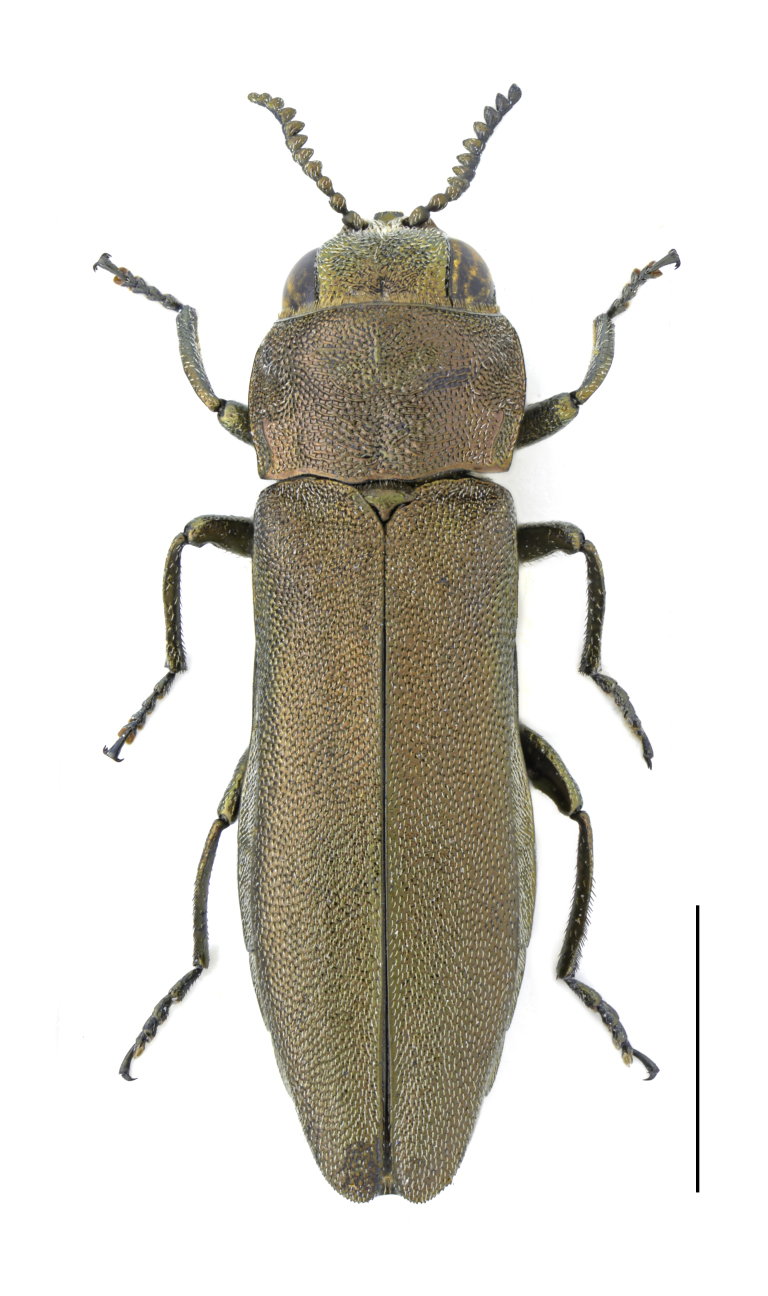
*Agrilusetruscus* Curletti, 2013 - scale bar: 2.0 mm;

**Figure 2c.**
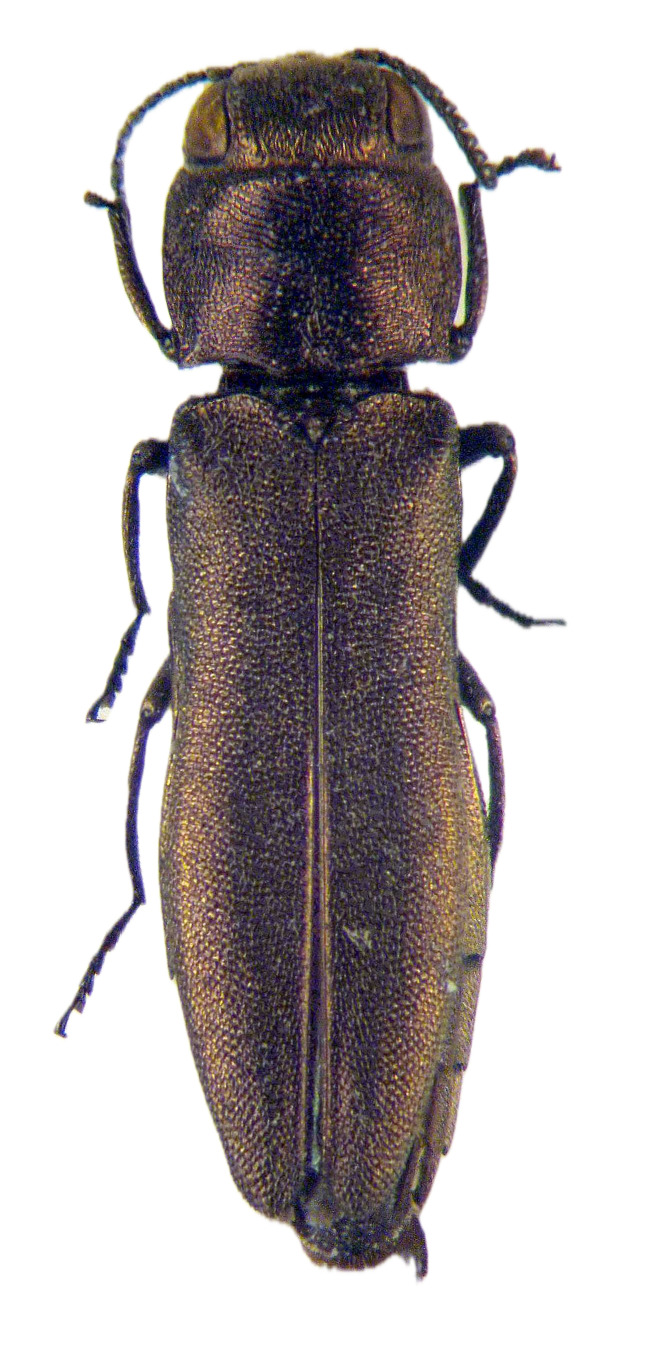
*Agrilusevocatus* Curletti, 2021 (Holotype). Total length 6.3 mm;

**Figure 2d.**
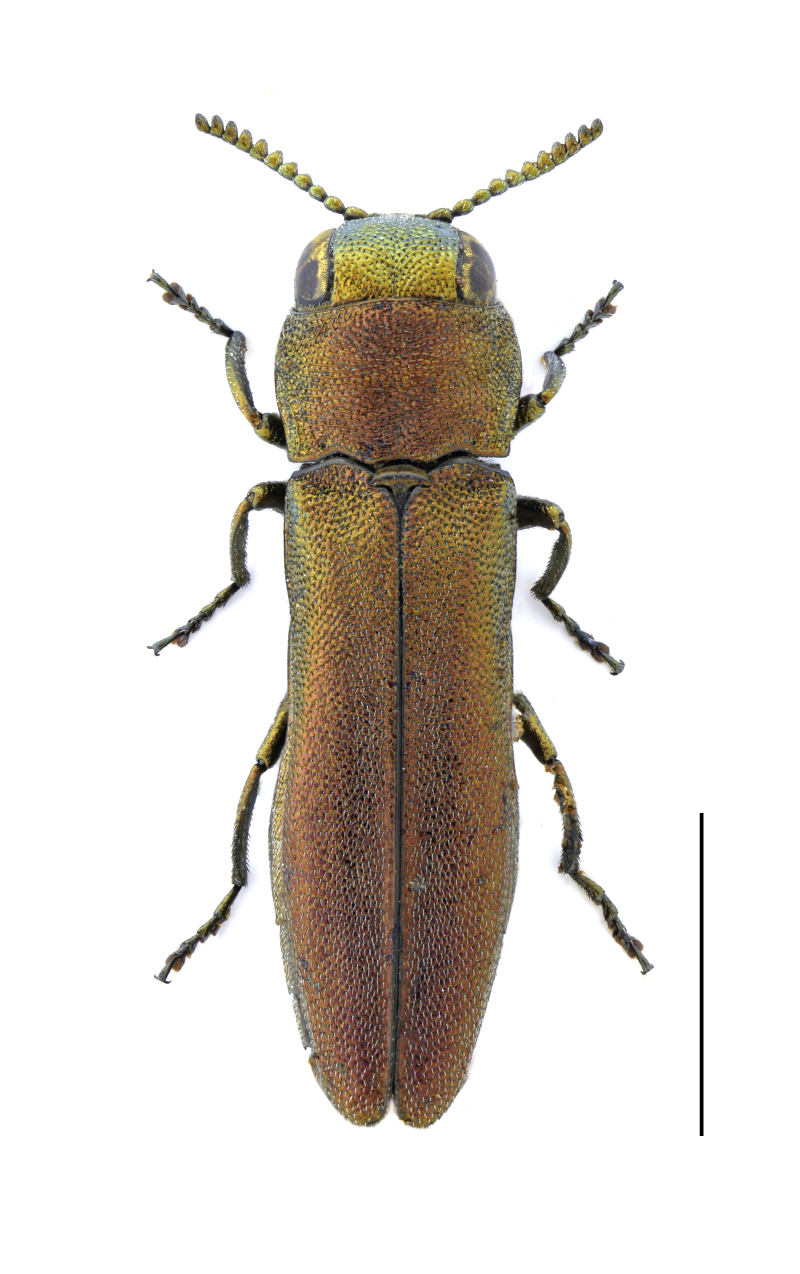
*Agrilushyperici* (Creutzer, 1799) - scale bar: 2.0 mm.

**Figure 3a.**
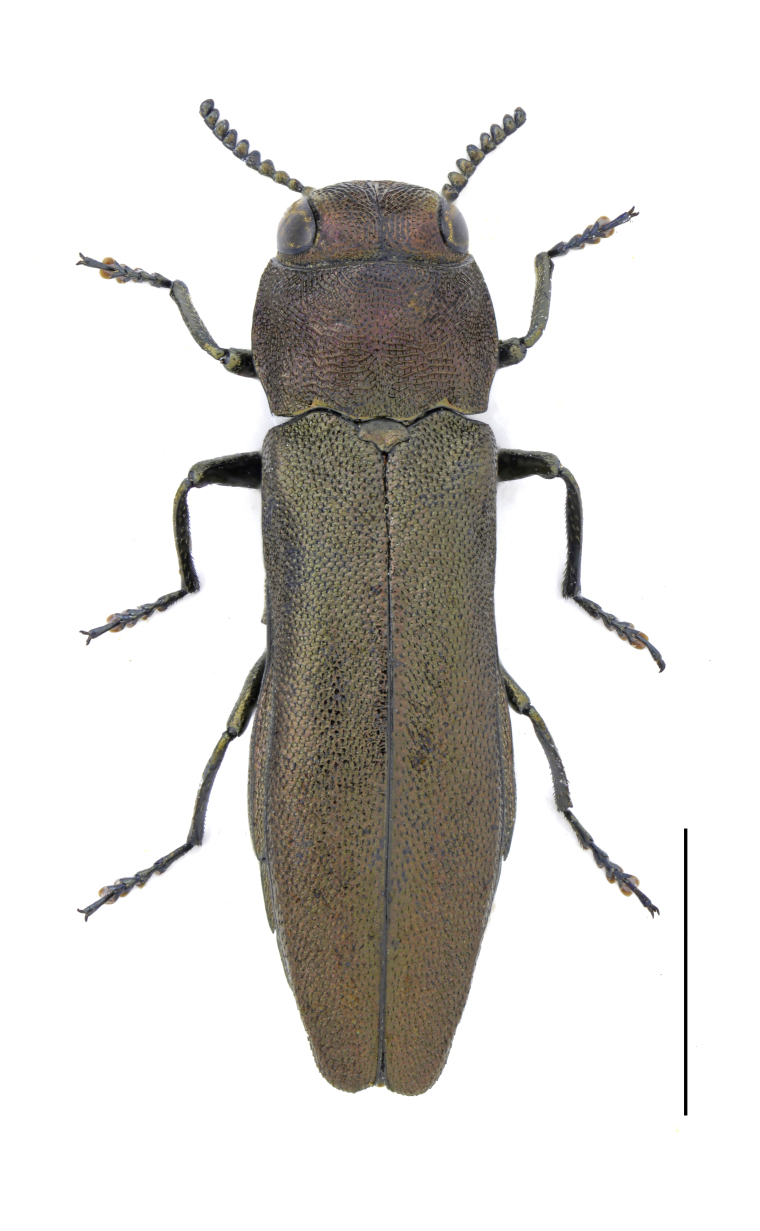
*Agrilusintegerrimus* (Ratzeburg, 1837) - scale bar: 2.0 mm;

**Figure 3b.**
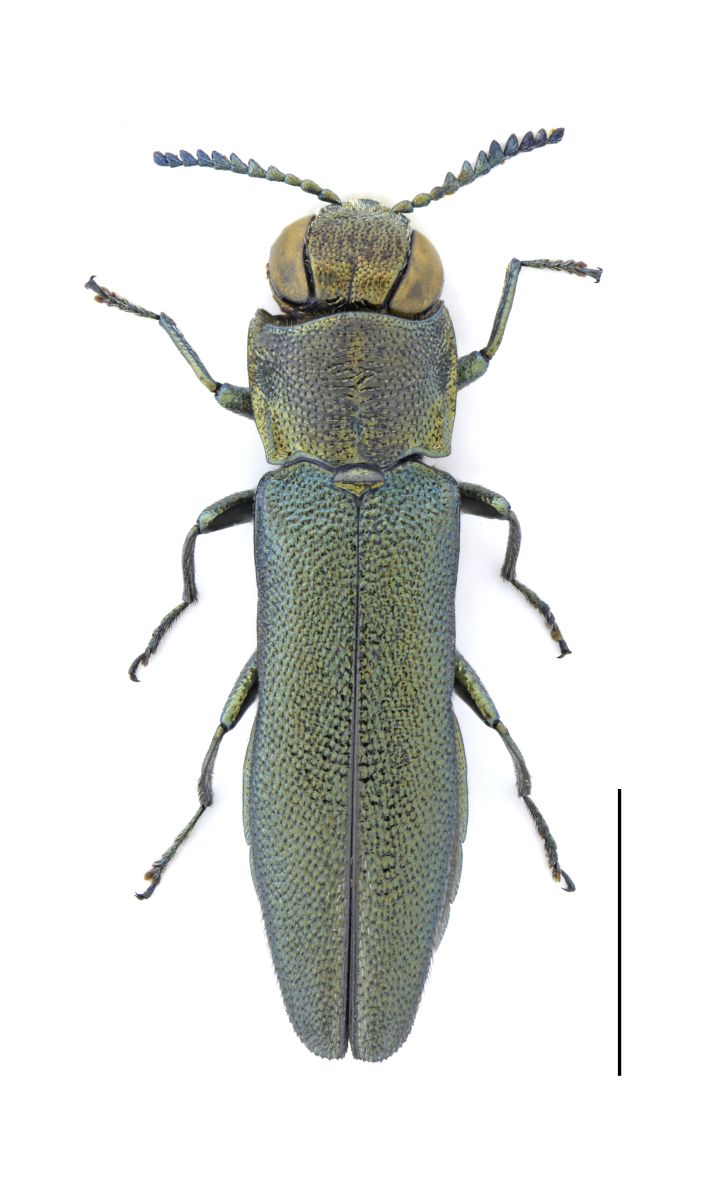
*Agriluslaticornis* (Illiger, 1803) - scale bar: 2.0 mm;

**Figure 3c.**
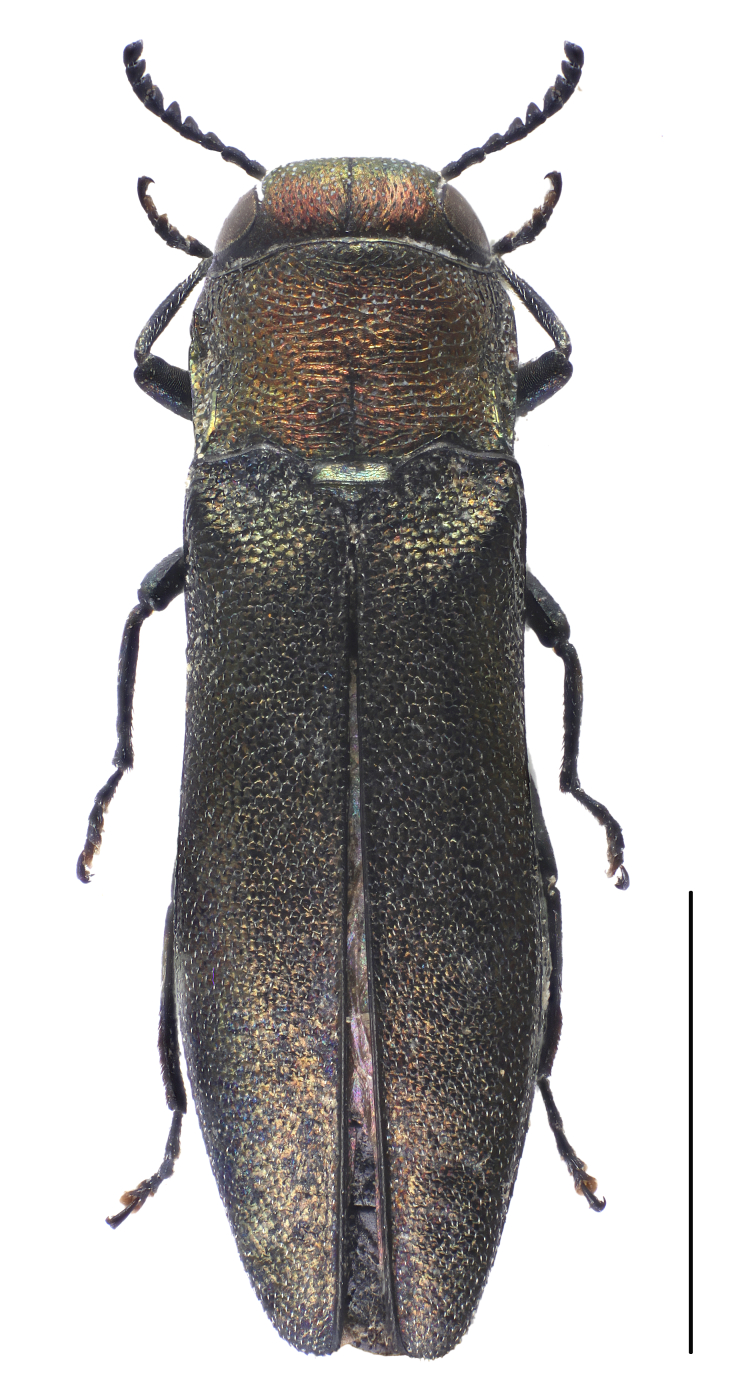
*Agrilusmarozzinii* Gobbi, 1974- scale bar: 2.0 mm;

**Figure 3d.**
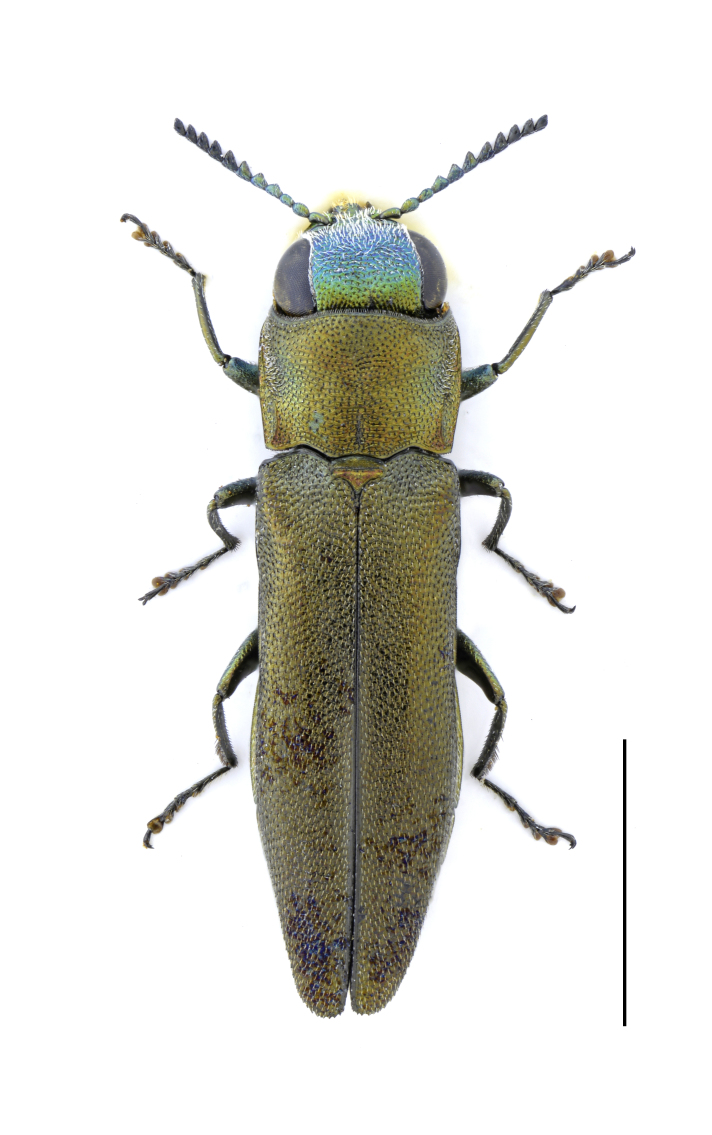
*Agrilusroscidus* Kiesenwetter, 1857 - scale bar: 2.0 mm.

**Figure 4a.**
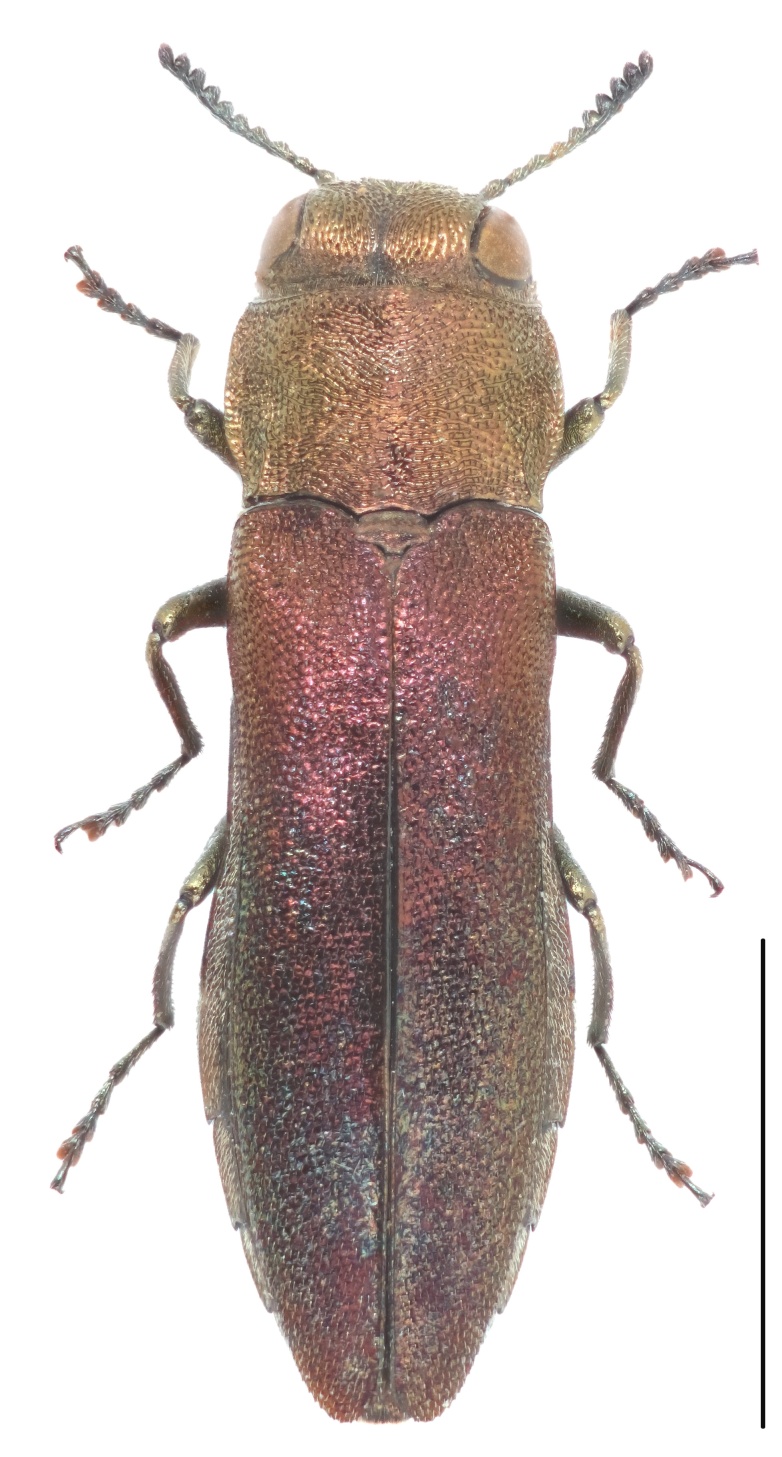
Agrilussolierissp.solieri Gory & Laporte, 1837 - scale bar: 2.0 mm;

**Figure 4b.**
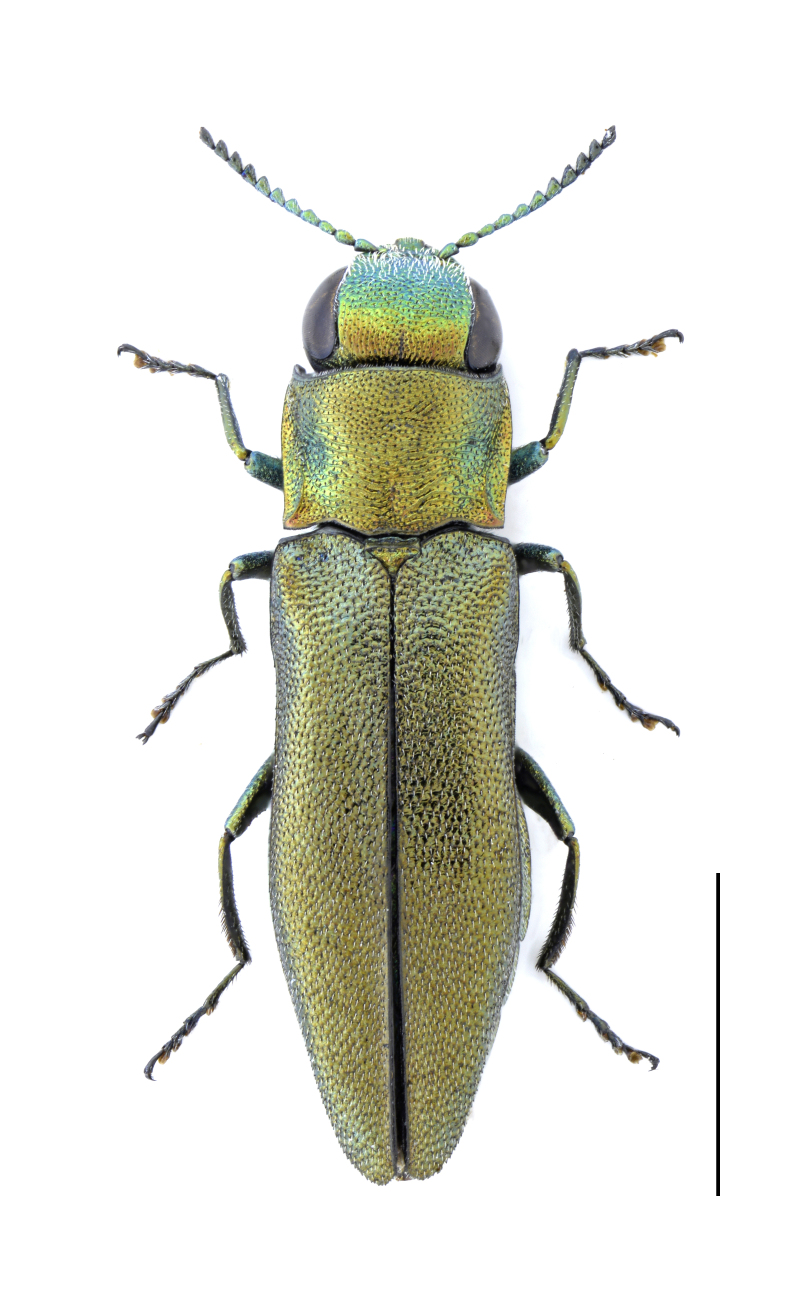
Agrilusviridicaerulansssp.rubi Schaefer, 1937 - scale bar: 2.0 mm;

**Figure 4c.**
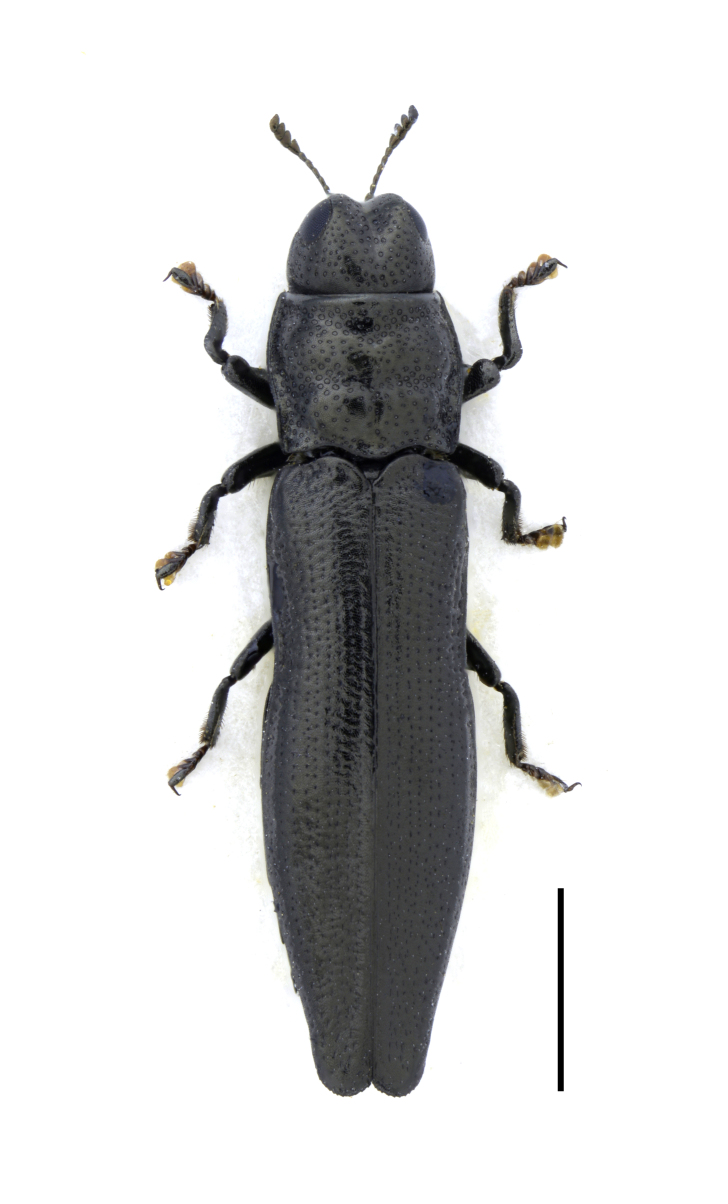
Aphanisticuselongatusssp.elongatus (Villa & Villa, 1835) - scale bar: 1.0 mm;

**Figure 4d.**
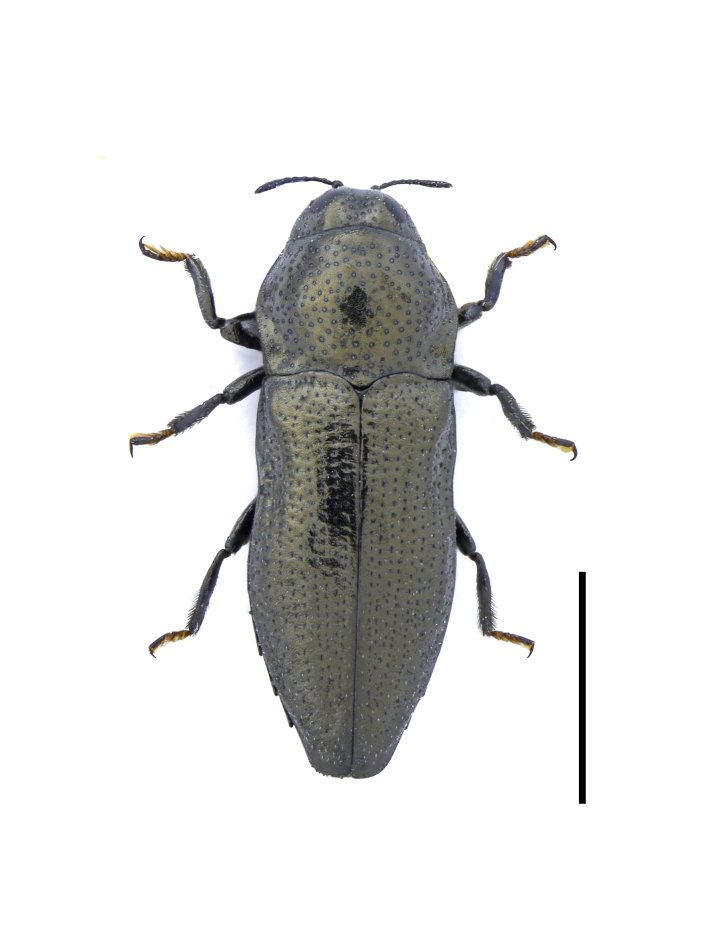
*Aphanisticuspygmaeus* Lucas, 1846 - scale bar: 1.0 mm.

**Figure 5a.**
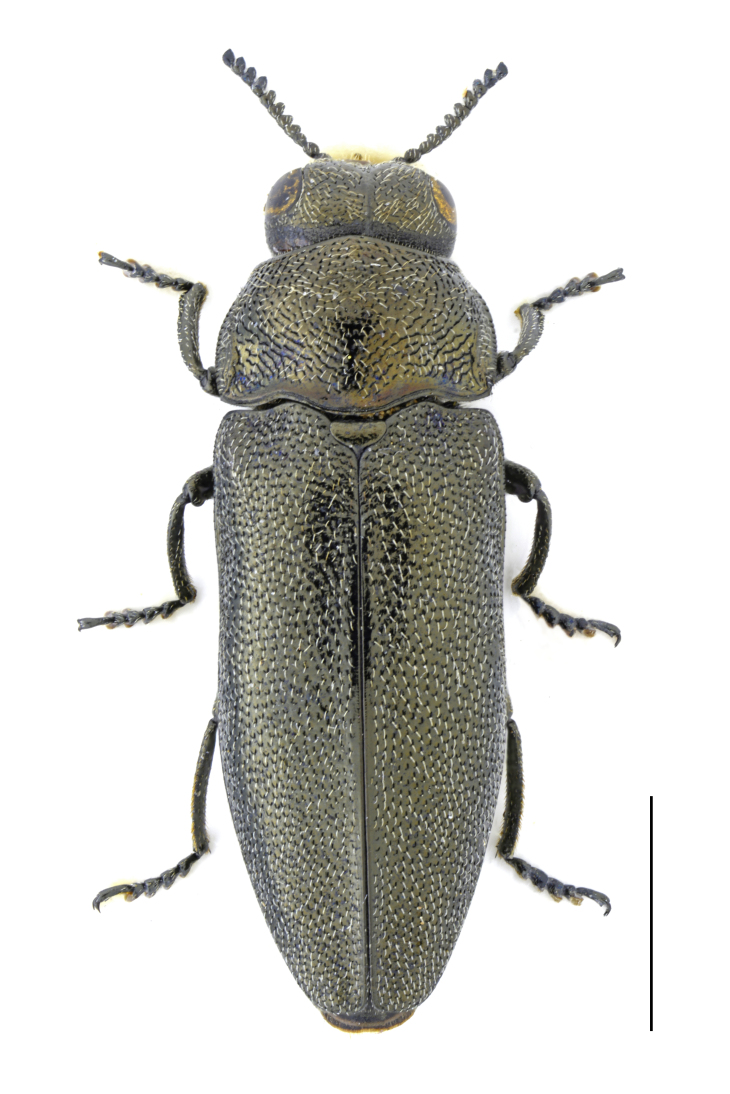
Coraebuselatusssp.elatus (Fabricius, 1787) - scale bar: 2.0 mm;

**Figure 5b.**
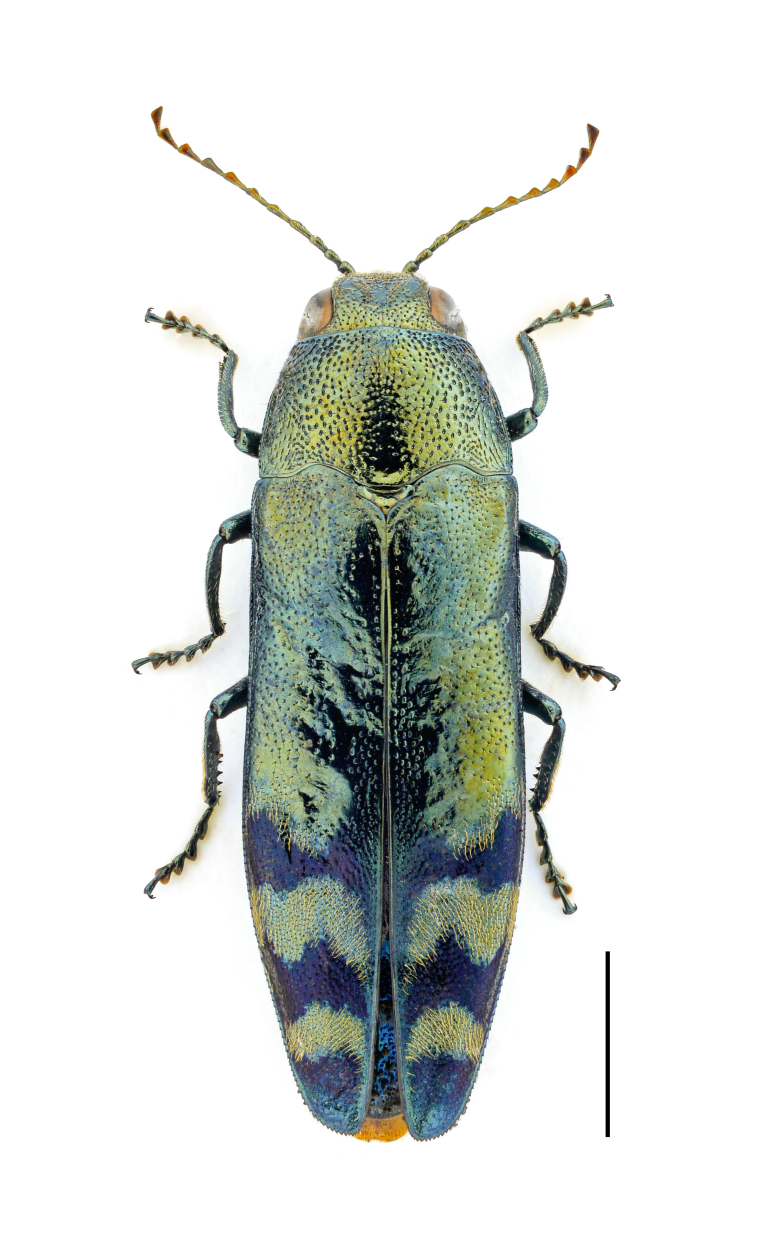
*Coraebusfasciatus* (Villers, 1789) - scale bar: 3.0 mm;

**Figure 5c.**
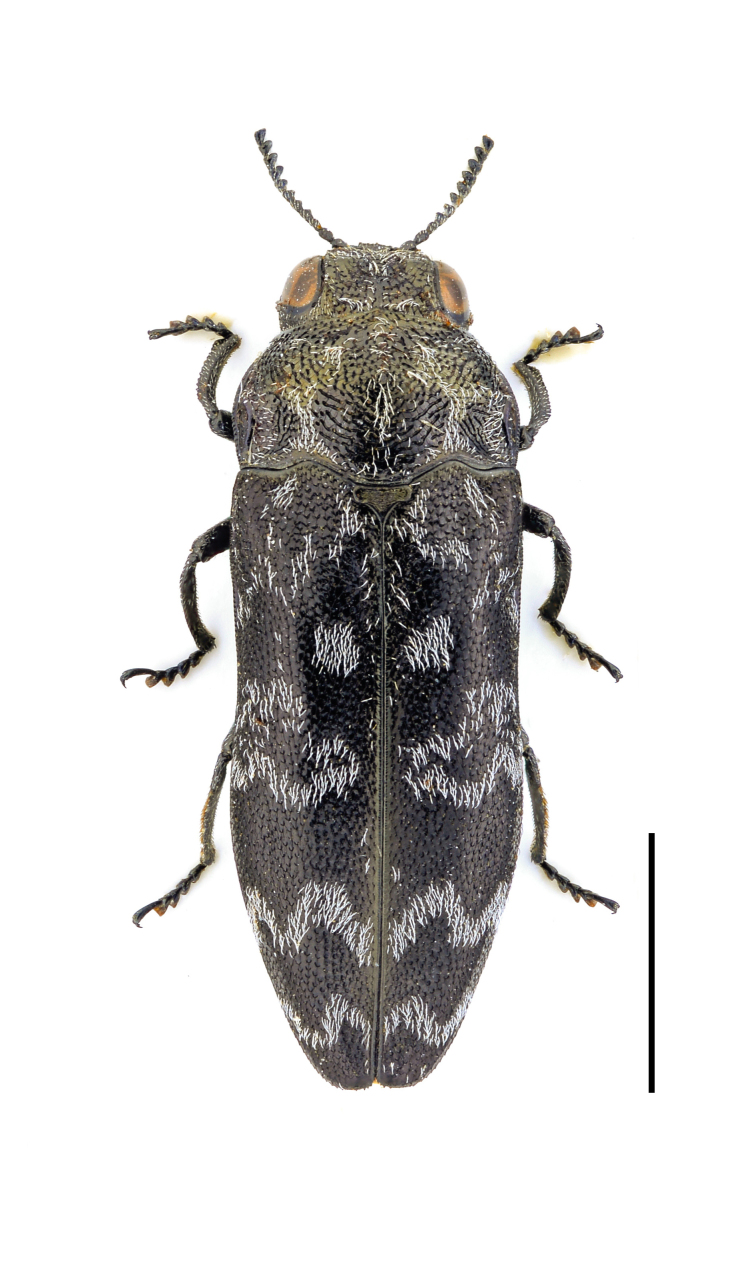
*Coraebusrubi* (Linnaeus, 1767) - scale bar: 3.0 mm;

**Figure 5d.**
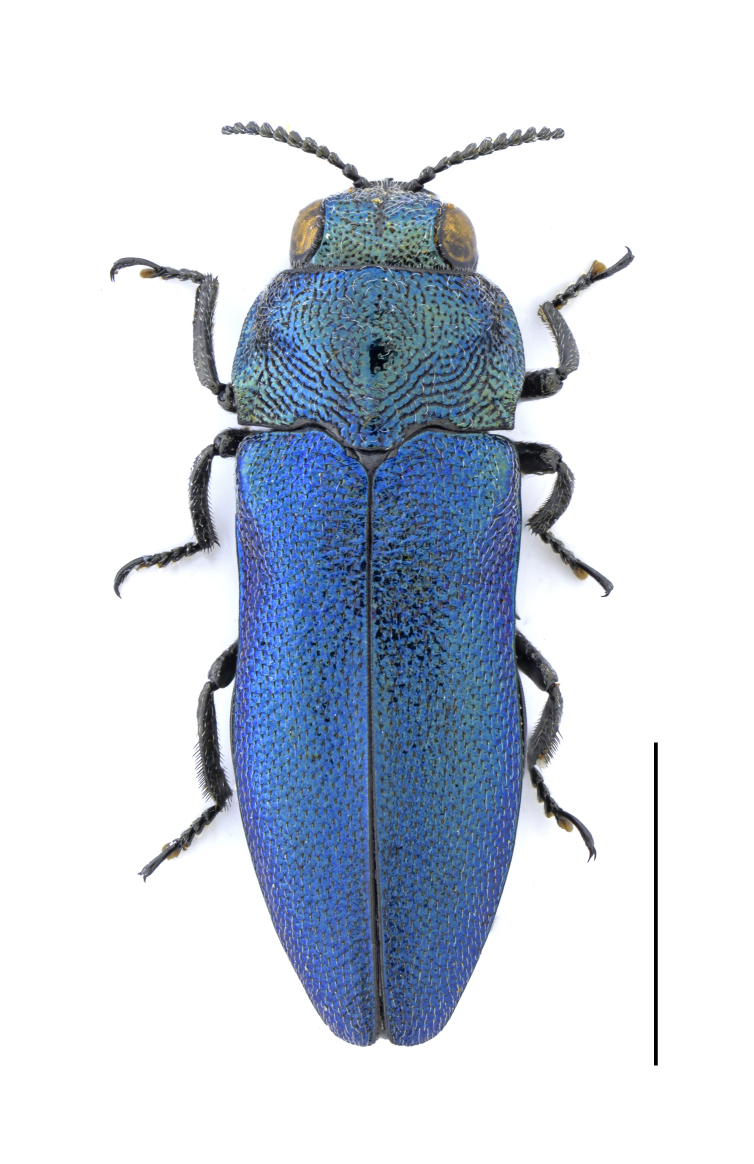
Meliboeus (Meliboeoides) parvulus (Kuster, 1852) - scale bar: 2.0 mm.

**Figure 6a.**
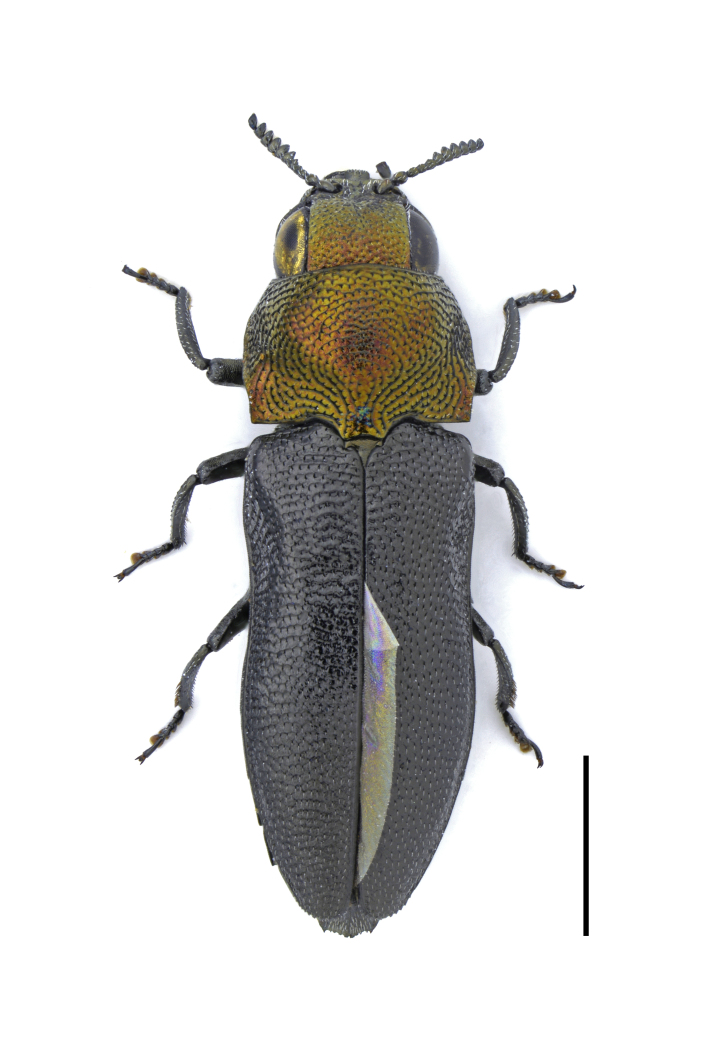
Meliboeus (Meliboeus) fulgidicollis (Lucas, 1846) - scale bar: 1.0 mm;

**Figure 6b.**
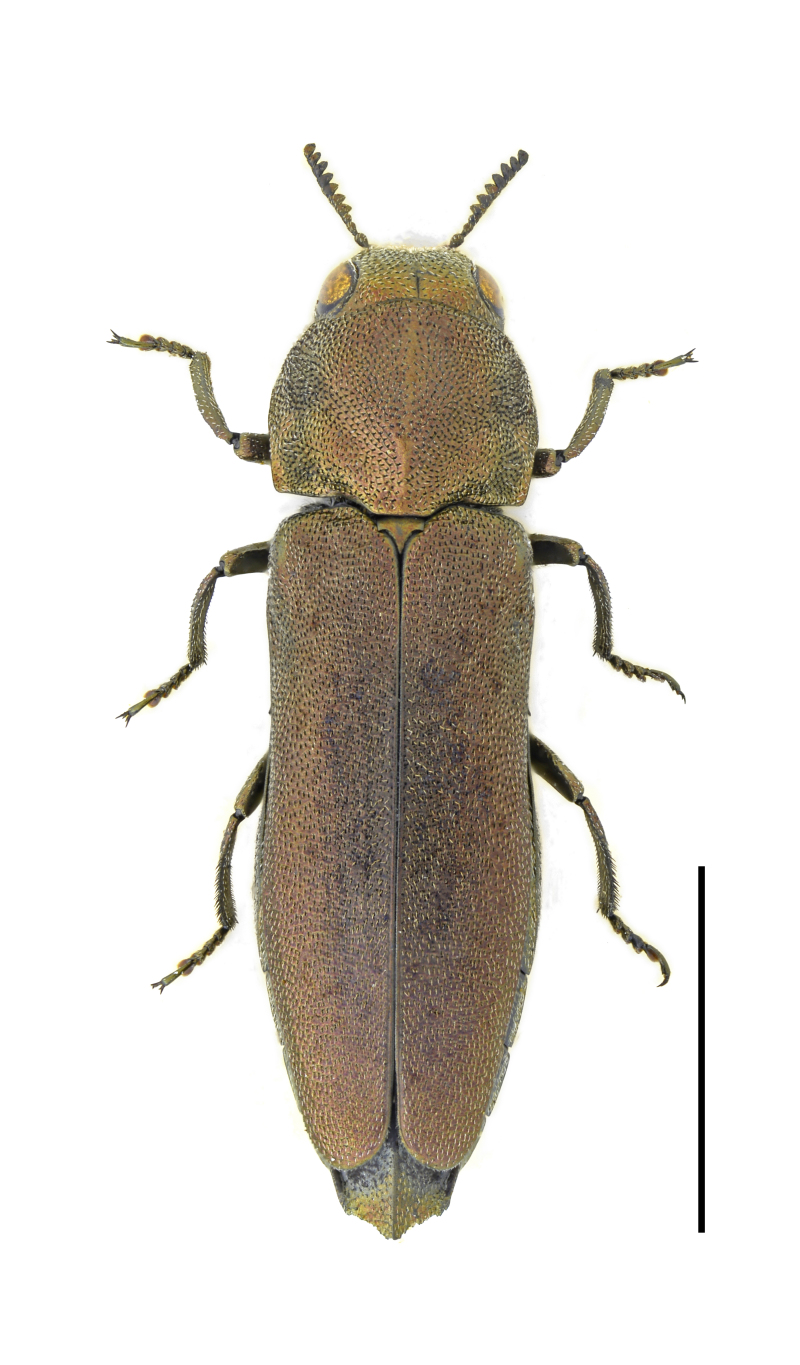
Meliboeus (Meliboeus) gibbicollis ssp. gibbicollis (Illiger, 1803) - scale bar: 3.0 mm;

**Figure 6c.**
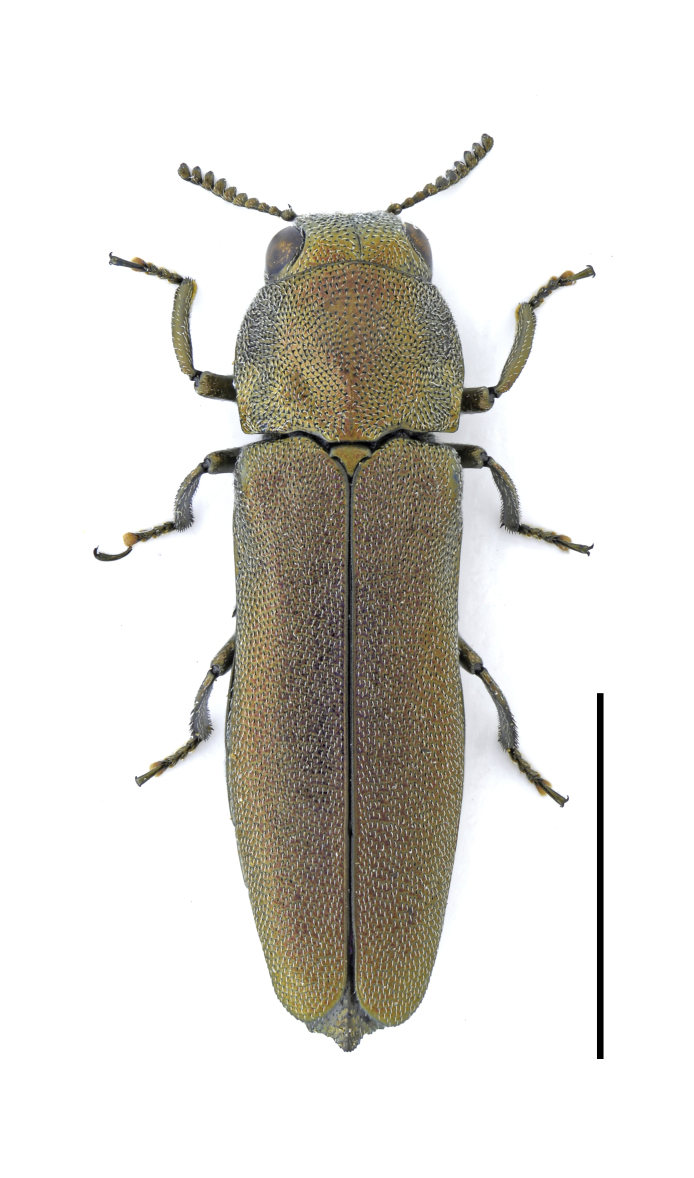
Meliboeus (Meliboeus) graminis ssp. graminis (Panzer, 1789) - scale bar: 3.0 mm;

**Figure 6d.**
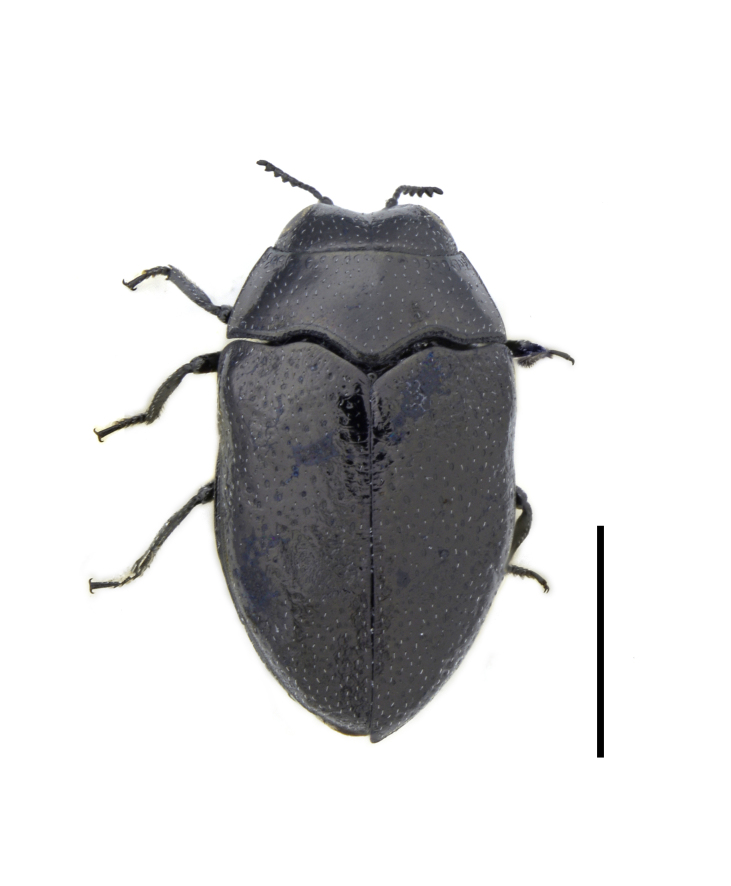
Trachyspuncticollisssp.rectilineatus Abeille de Perrin, 1900 - scale bar: 1.0 mm.

**Figure 7a.**
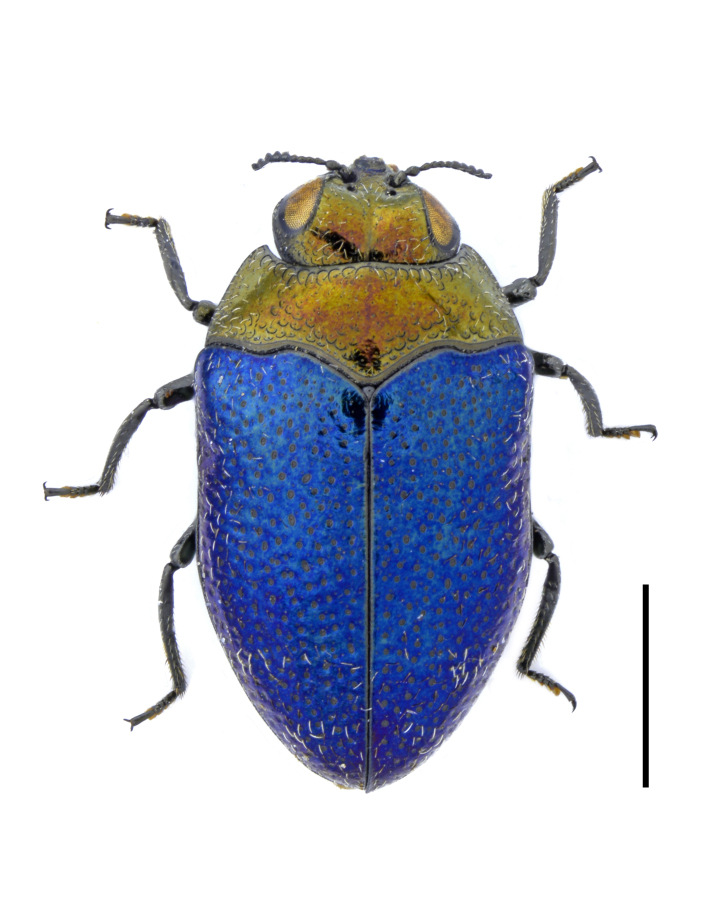
*Trachystroglodytiformis* Obenberger, 1918 - scale bar: 1.0 mm;

**Figure 7b.**
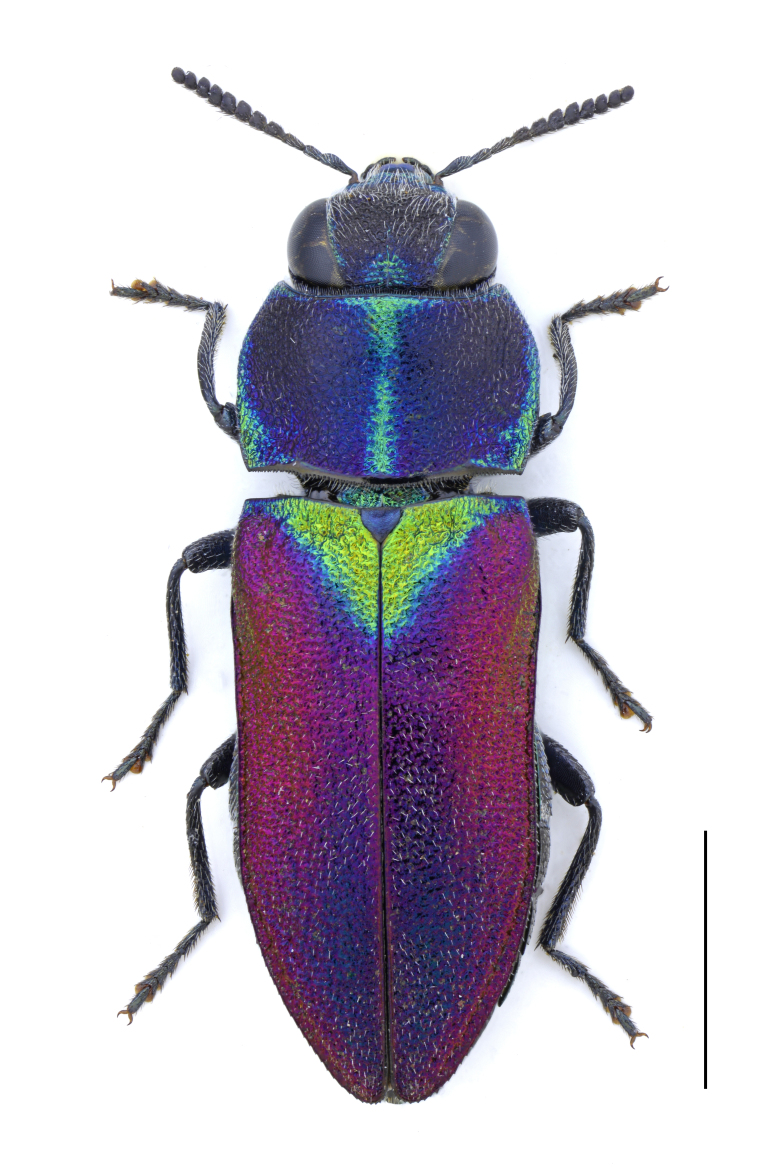
Anthaxia (Haplanthaxia) croesus (Villers, 1789) - scale bar: 2.0 mm;

**Figure 7c.**
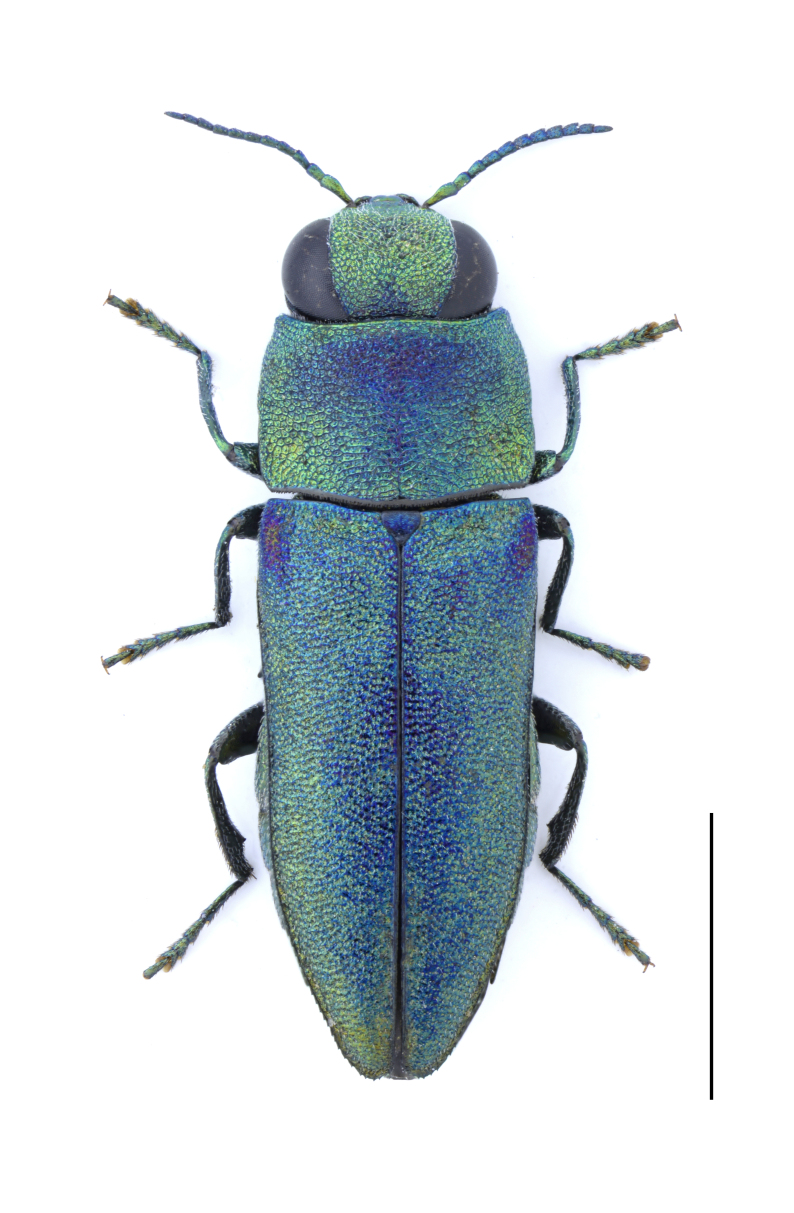
Anthaxia (Haplanthaxia) millefolii ssp. polychloros Abeille de Perrin, 1894 (blue form) - scale bar: 20 mm;

**Figure 7d.**
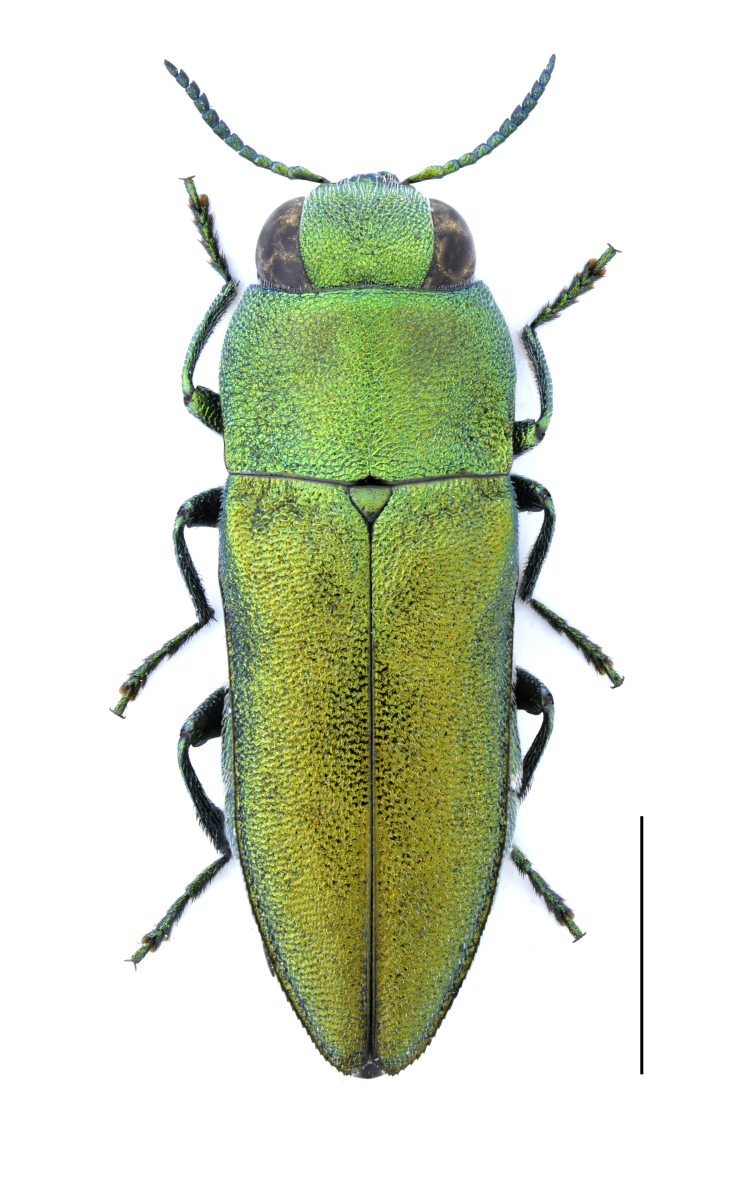
Anthaxia (Haplanthaxia) millefolii ssp. polychloros Abeille de Perrin, 1894 (green form) - scale bar: 2.0 mm.

**Figure 8a.**
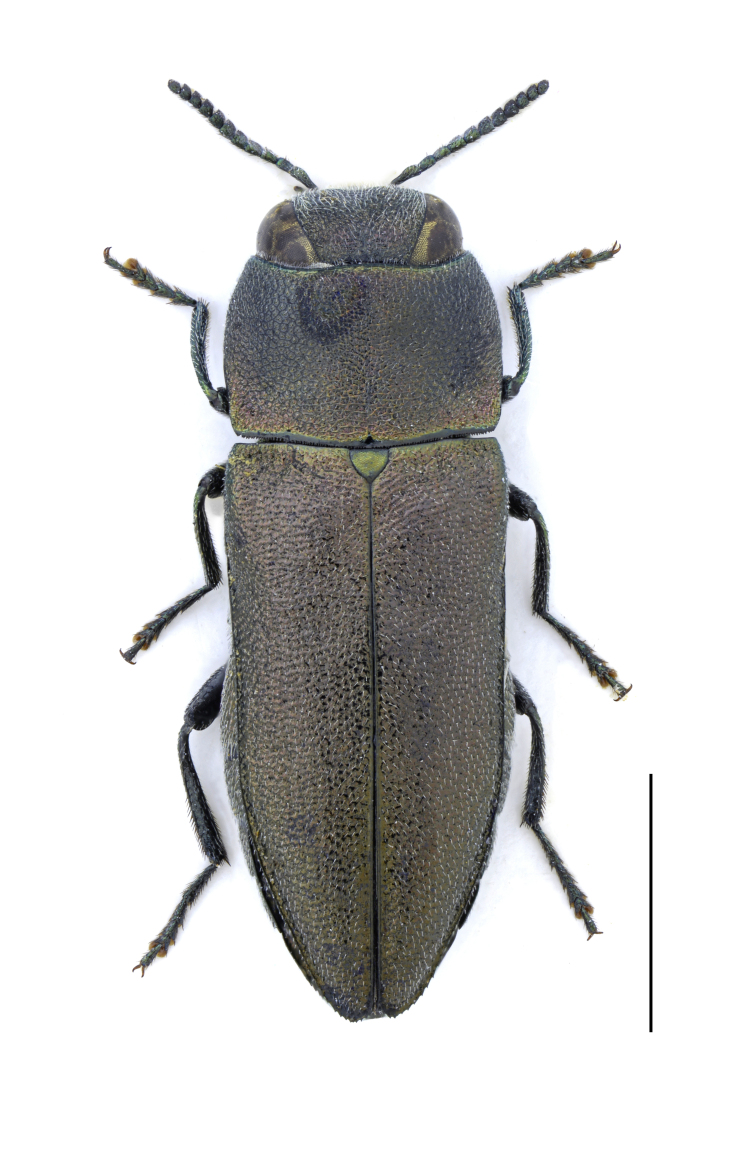
Anthaxia (Haplanthaxia) millefolii ssp. polychloros Abeille de Perrin, 1894 (bronze form) - scale bar: 2.0 mm

**Figure 8b.**
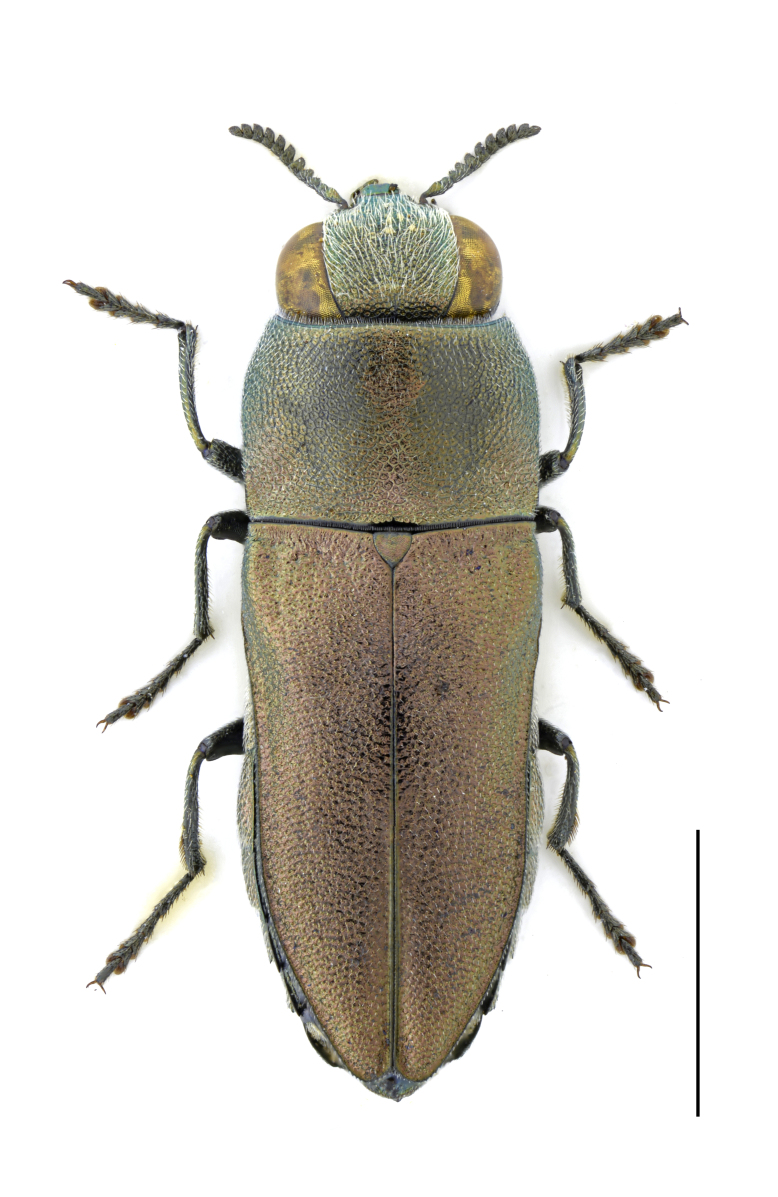
Anthaxia (Haplanthaxia) umbellatarum ssp. umbellatarum (Fabricius, 1787) - scale bar: 2.0 mm;

**Figure 8c.**
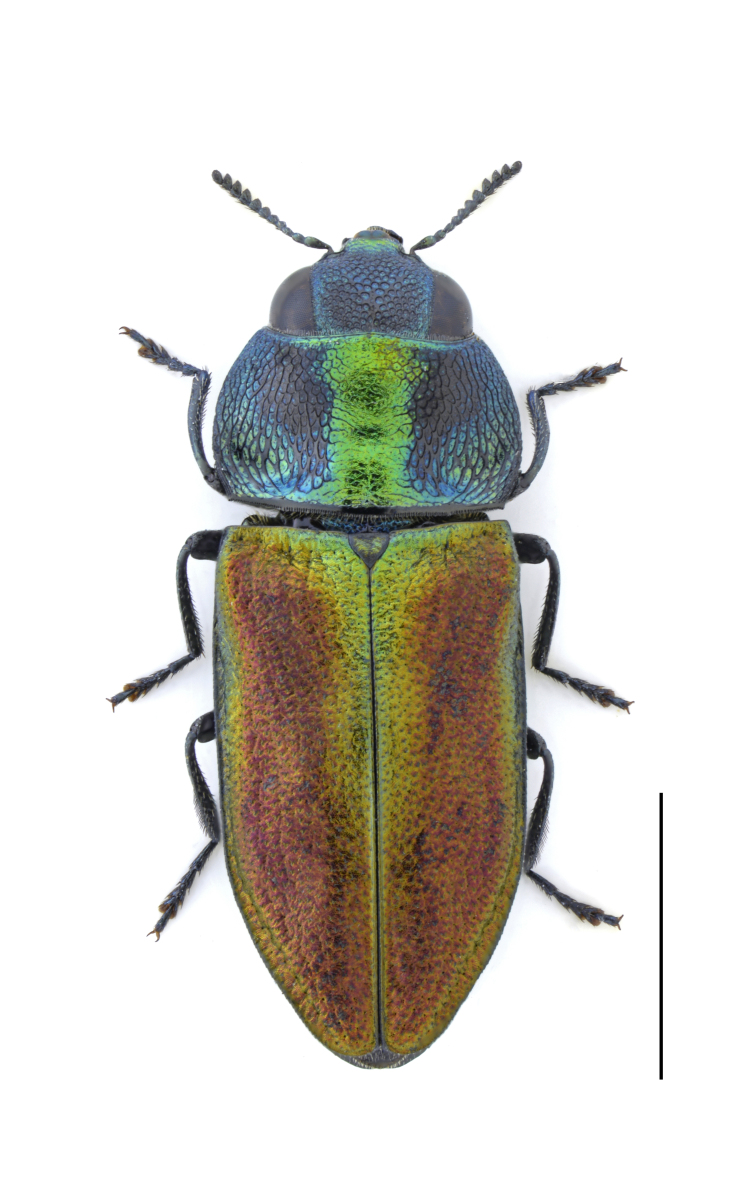
Anthaxia (Anthaxia) thalassophila ssp. thalassophila Abeille de Perrin, 1900 (♀) - scale bar: 2.0 mm;

**Figure 8d.**
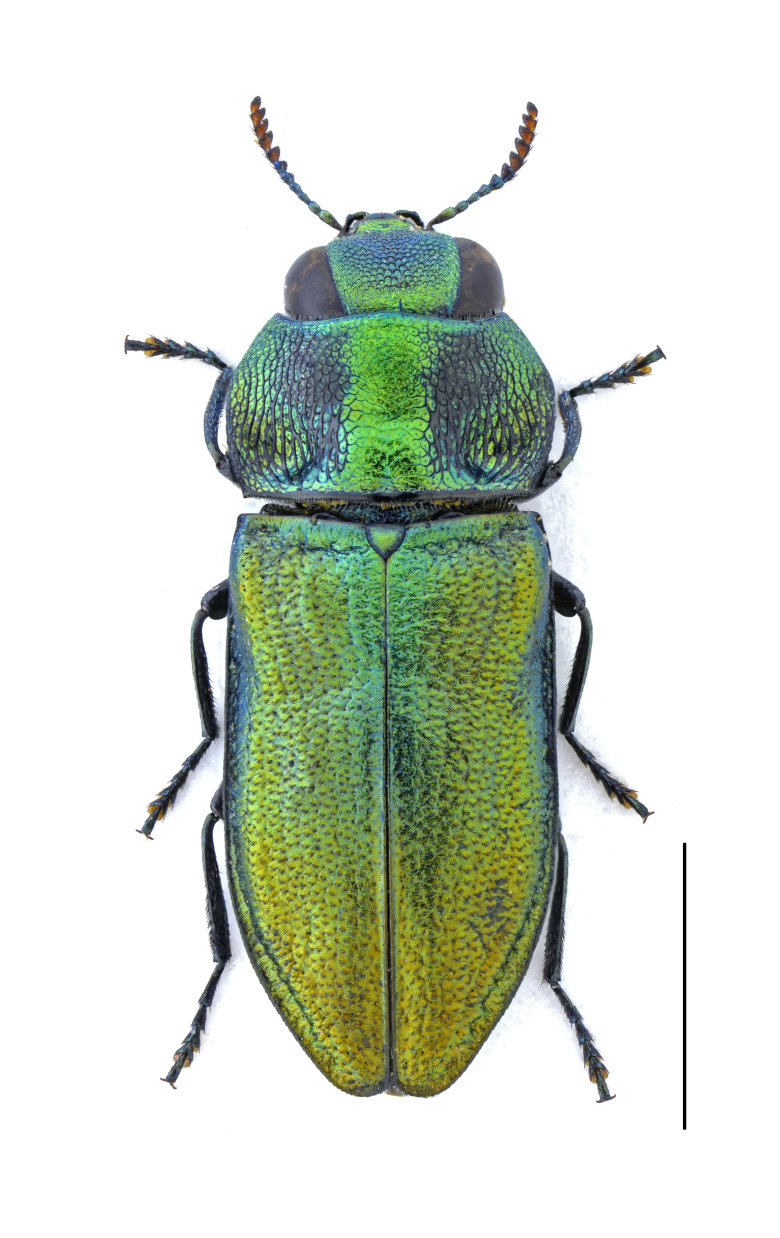
Anthaxia (Anthaxia) thalassophila ssp. thalassophila Abeille de Perrin, 1900 (♂) - scale bar: 2.0 mm.

**Figure 9a.**
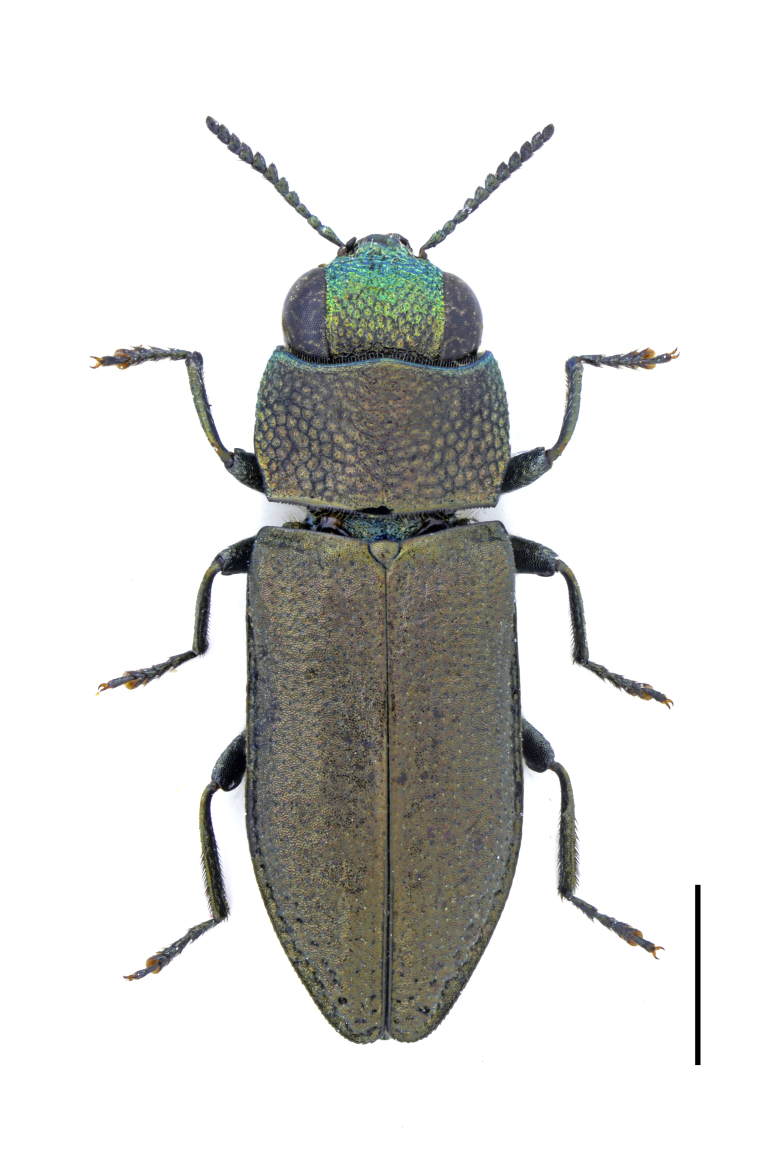
Anthaxia (Anthaxia) chevrieri Gory & Laporte, 1839 - scale bar: 1.0 mm;

**Figure 9b.**
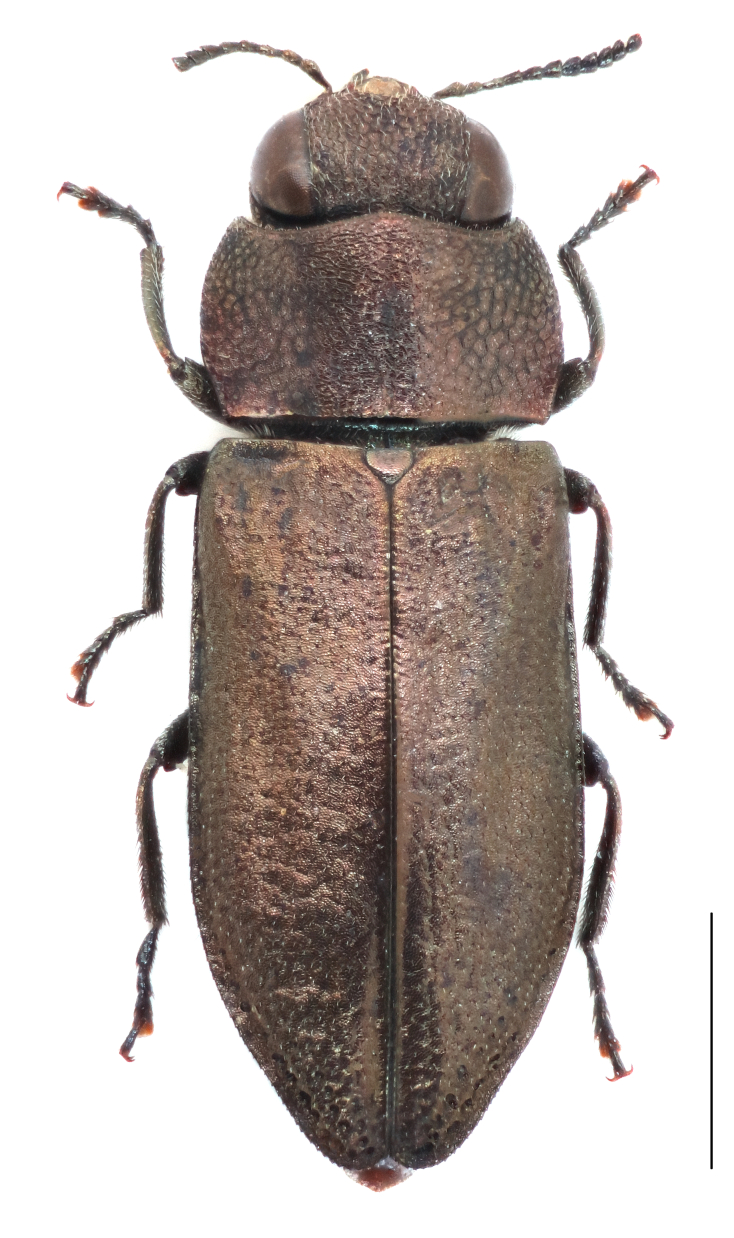
Anthaxia (Anthaxia) mendizabali Cobos, 1965 - scale bar: 1.0 mm;

**Figure 9c.**
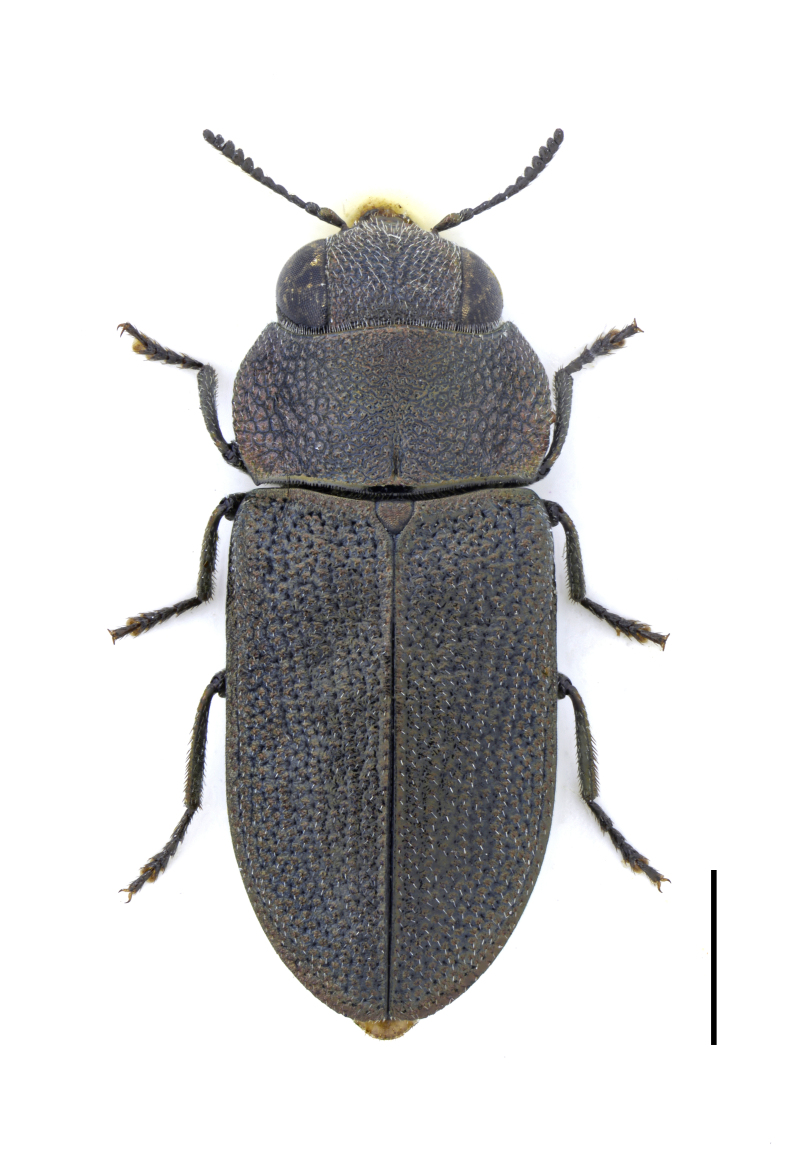
Anthaxia (Melanthaxia) nigritula ssp. nigritula Ratzeburg, 1837 - scale bar: 1.0 mm;

**Figure 9d.**
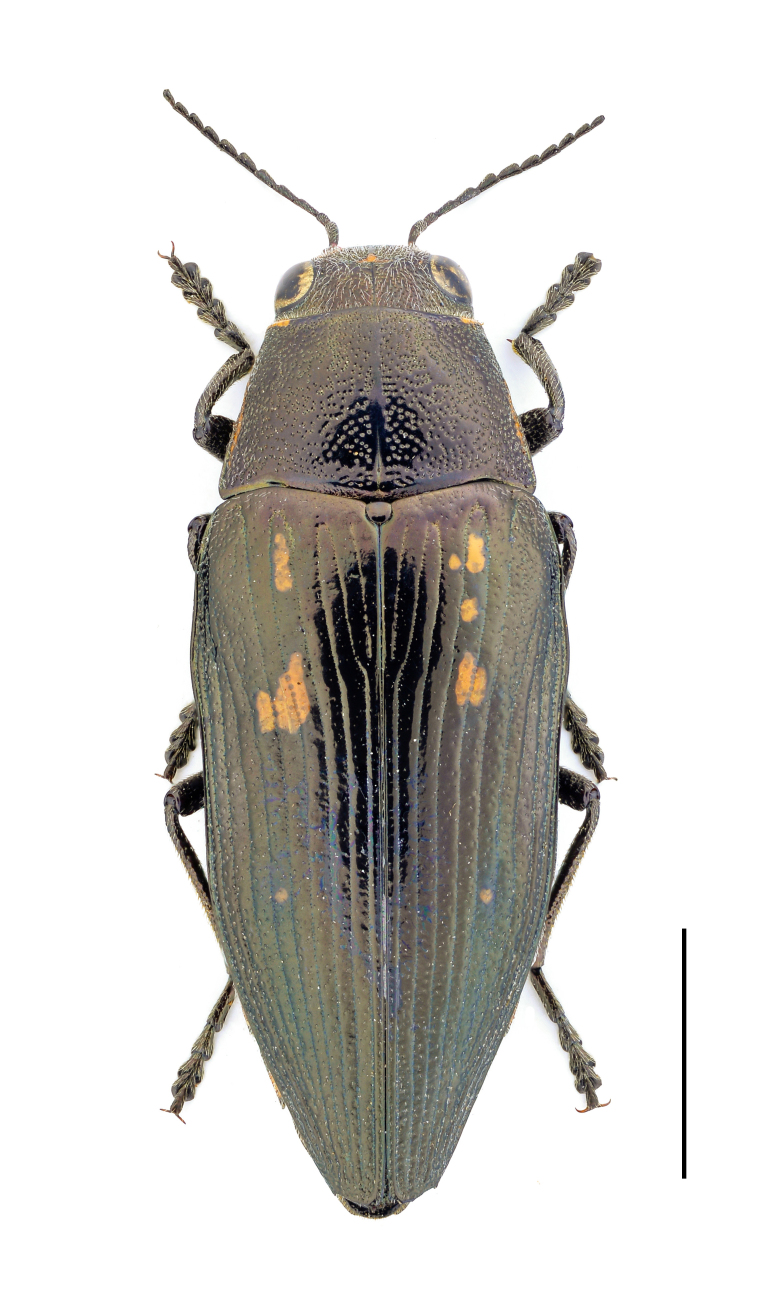
Buprestis (Ancylocheira) novemmaculata ssp. novemmaculata Linnaeus, 1767 - scale bar: 5.0 mm.

**Figure 10a.**
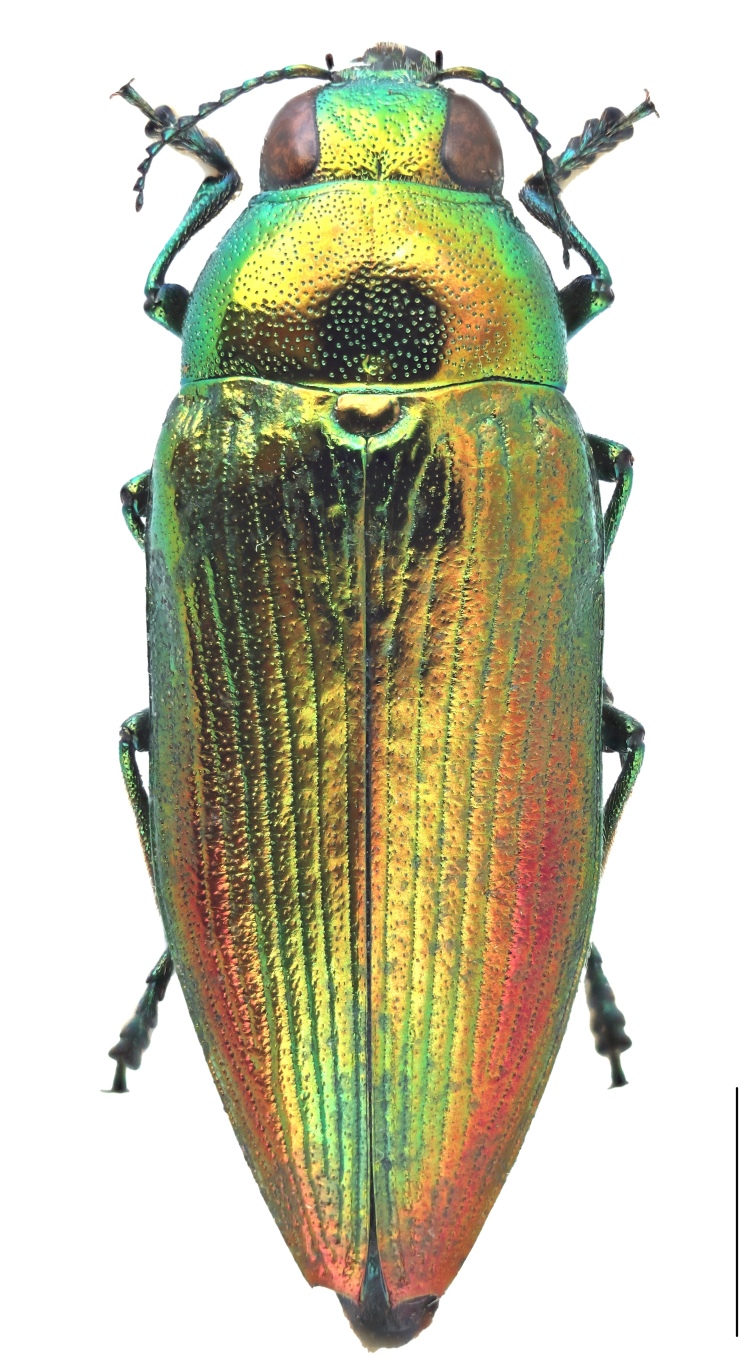
*Eurythyreamicans* (Fabricius, 1792) - scale bar: 4.0 mm;

**Figure 10b.**
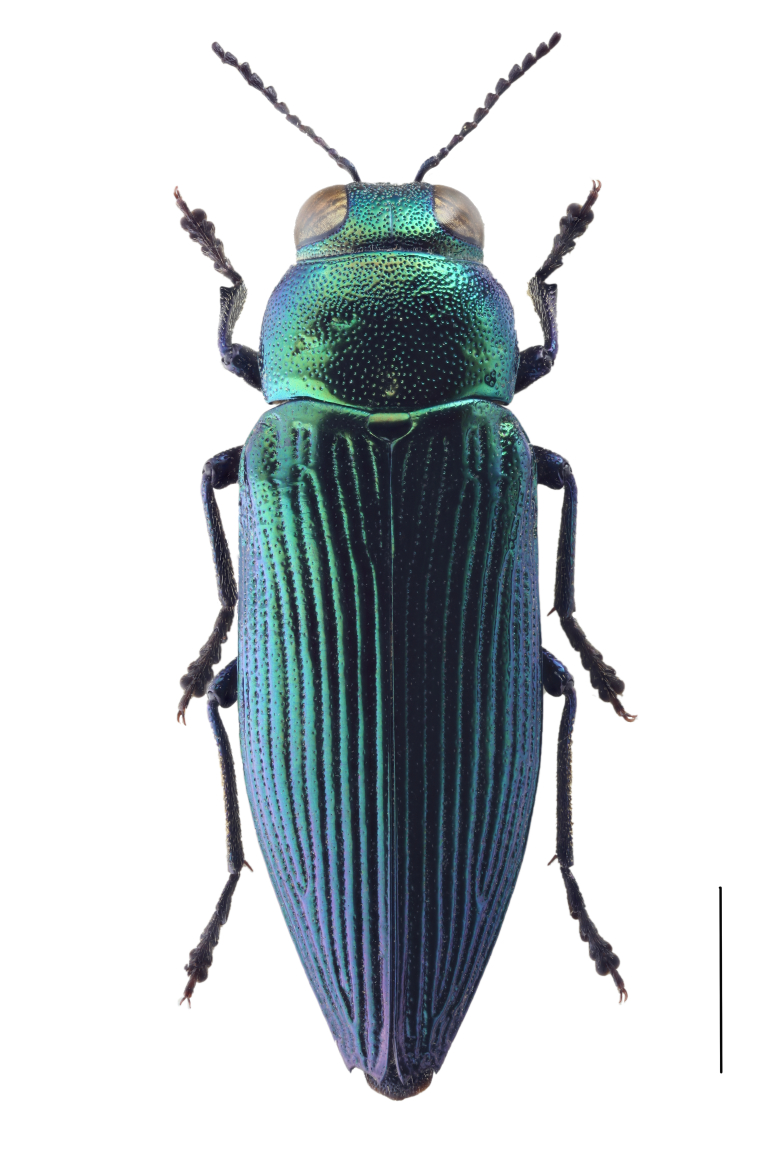
*Eurythyreaquercus* (Herbst, 1780) - scale bar: 4.0 mm;

**Figure 10c.**
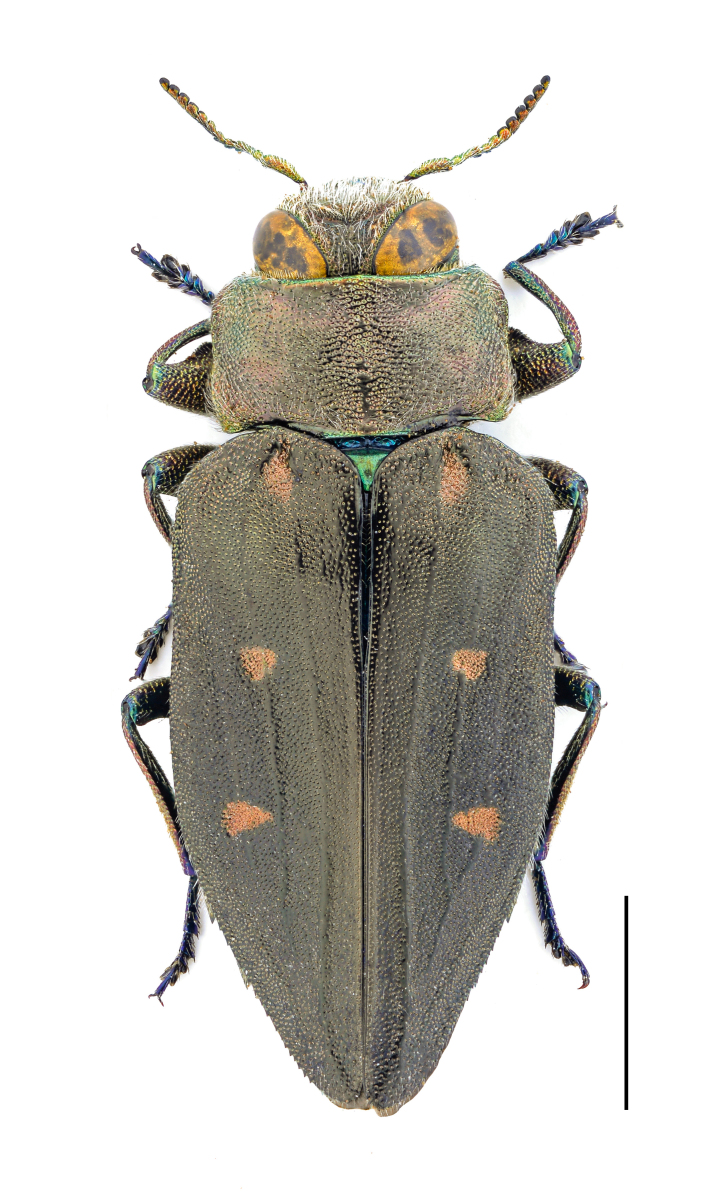
Chrysobothris (Chrysobothris) affinis ssp. affinis (Fabricius, 1794) - scale bar: 5.0 mm;

**Figure 10d.**
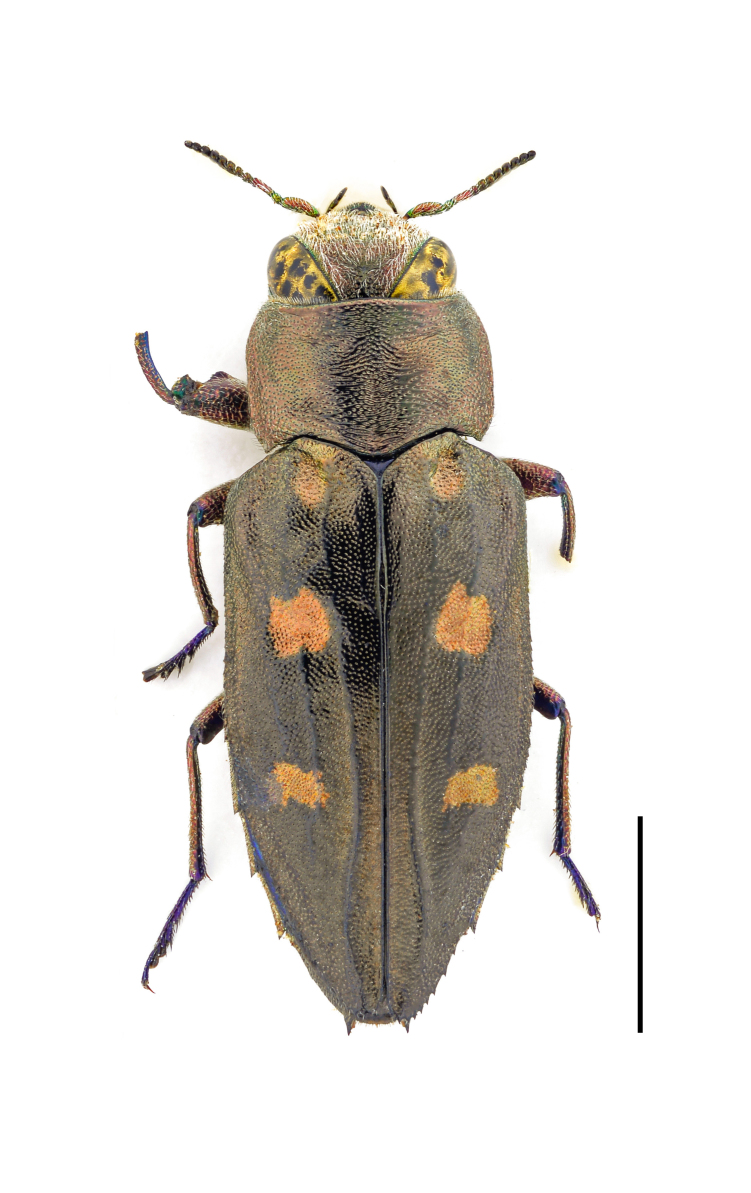
Chrysobothris (Chrysobothris) solieri Gory & Laporte, 1839 - scale bar: 3.0 mm.

**Figure 11a.**
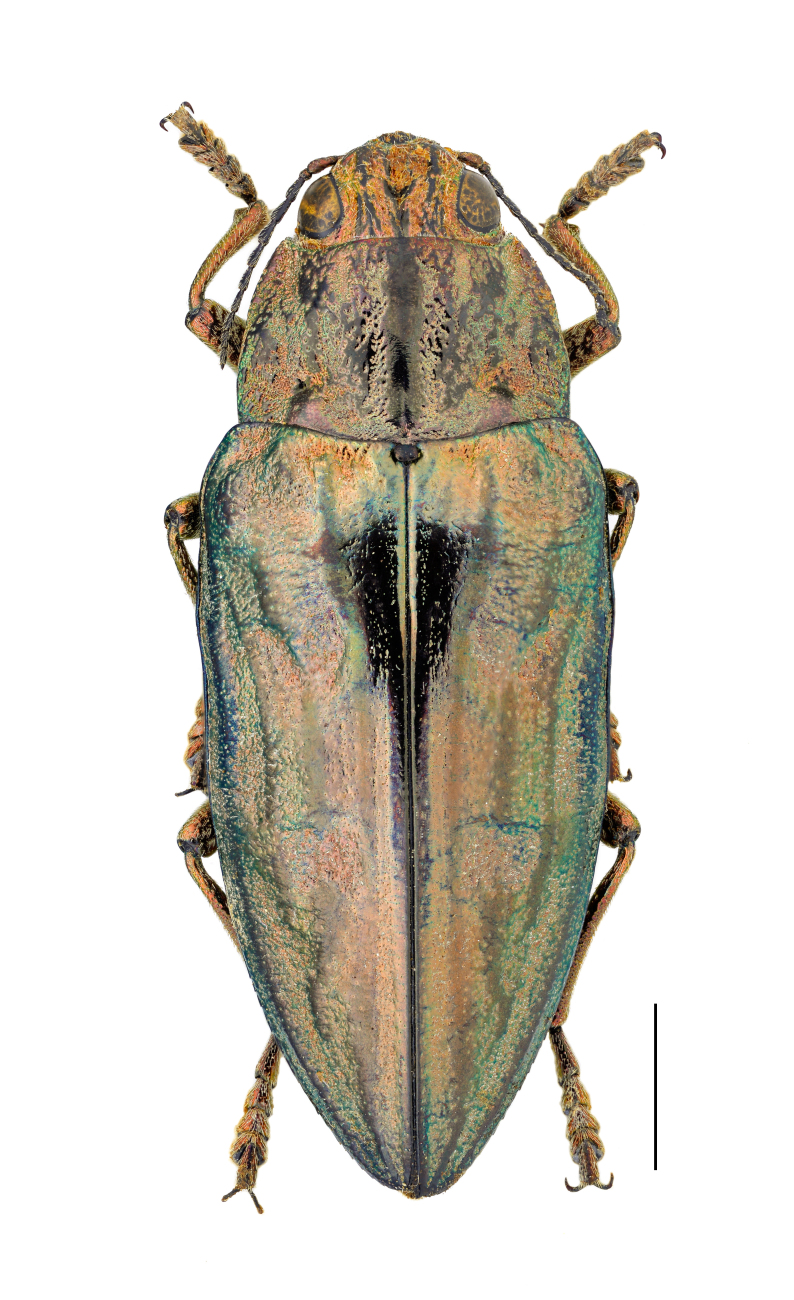
*Chalcophoramassiliensis* (Villers, 1789) - scale bar: 5.0 mm;

**Figure 11b.**
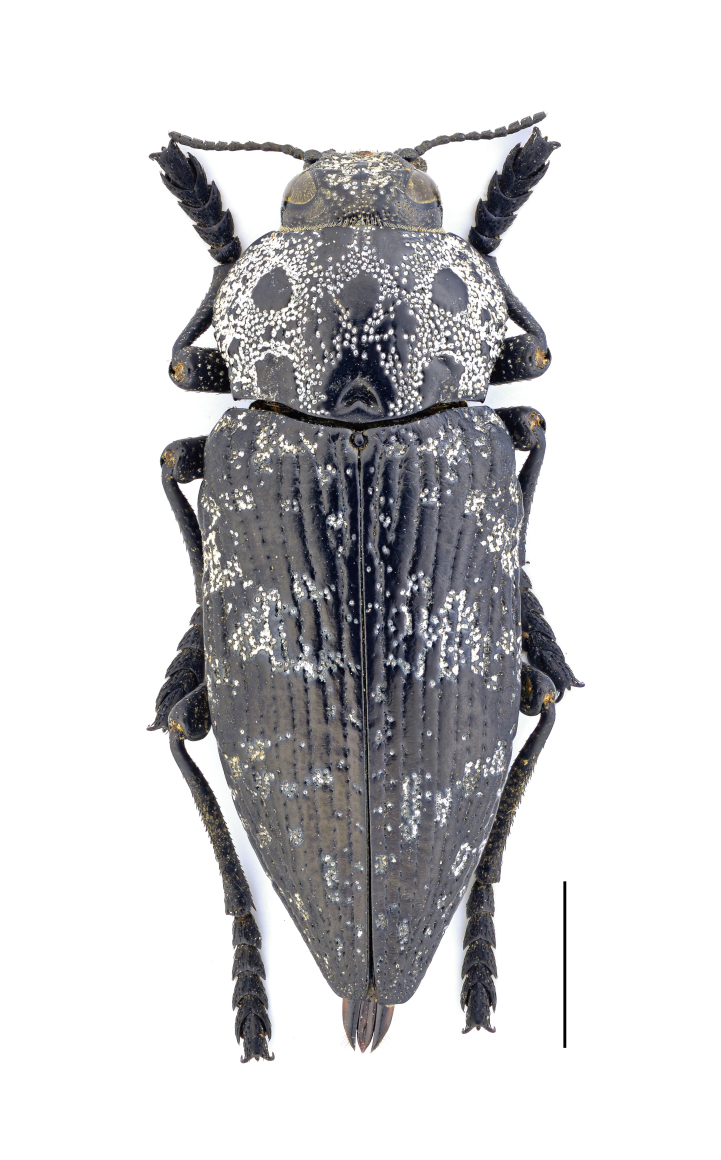
Capnodiscariosassp.cariosa (Pallas, 1776) - scale bar: 5.0 mm;

**Figure 11c.**
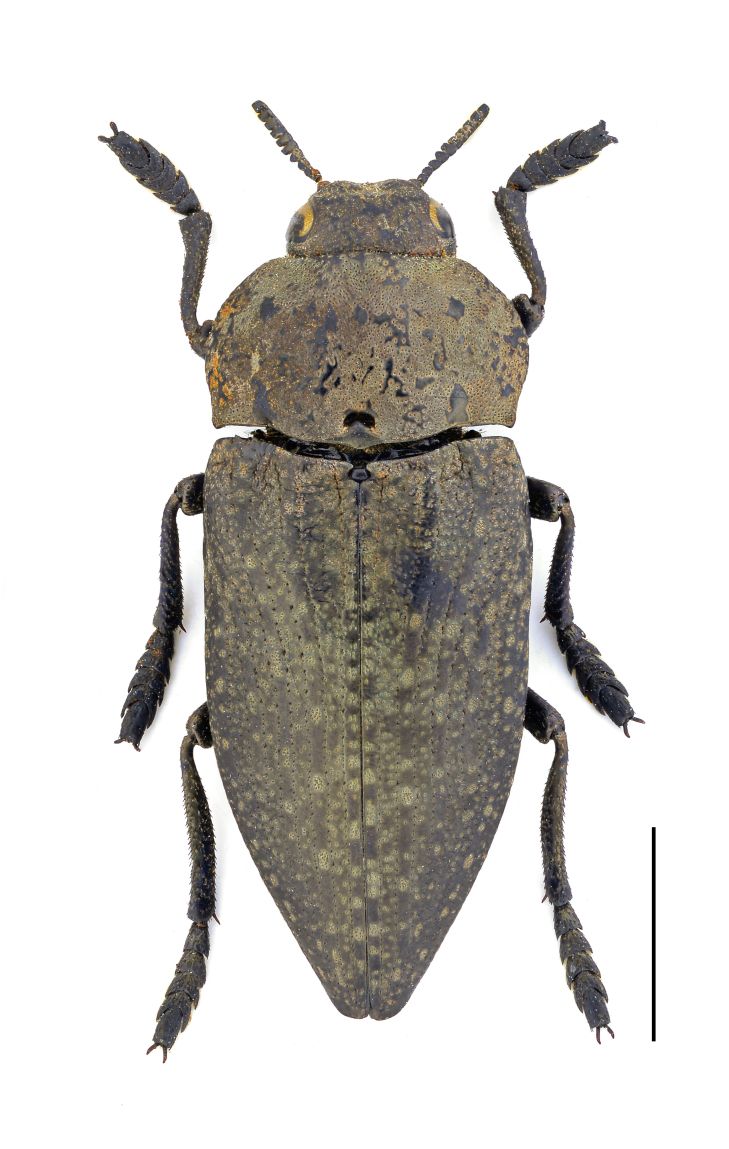
Capnodistenebricosassp.tenebricosa (Olivier, 1790) - scale bar: 5.0 mm;

**Figure 11d.**
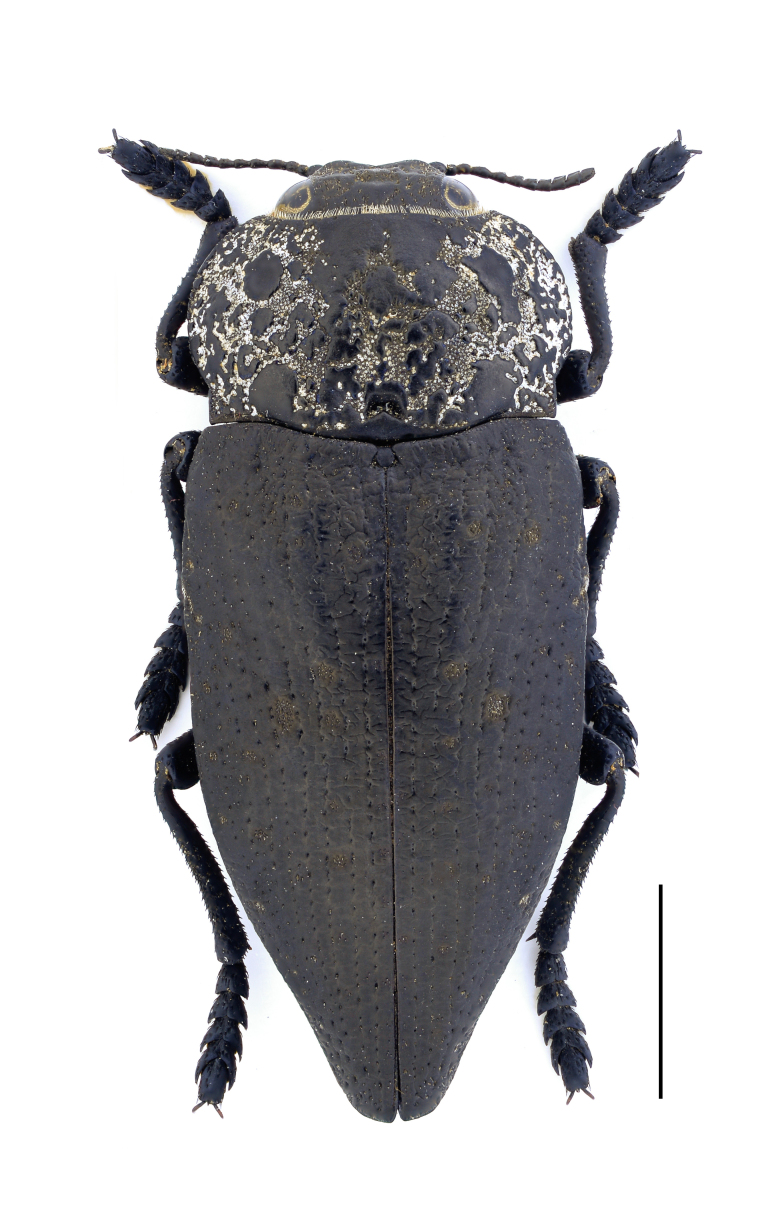
*Capnodistenebrionis* (Linnaeus, 1761) - scale bar: 5.0 mm.

**Figure 12a.**
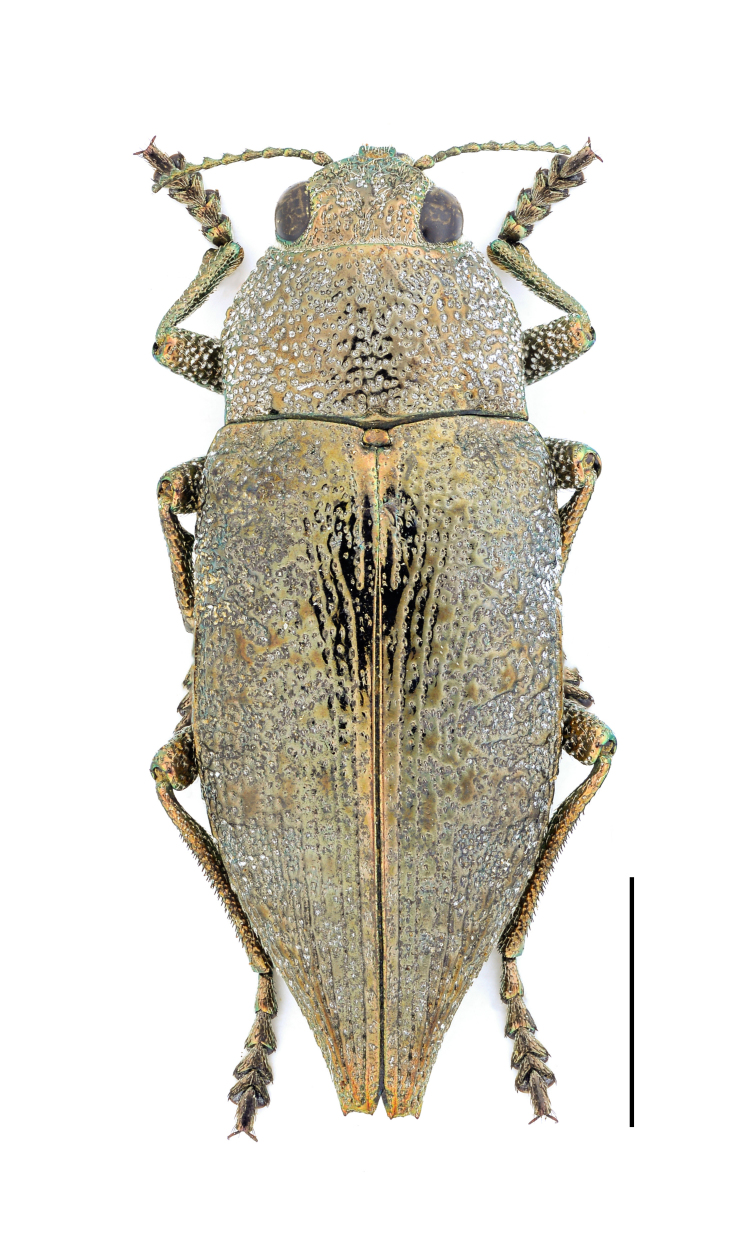
Dicercaaeneassp.aenea (Linnaeus, 1767) - scale bar: 5.0 mm;

**Figure 12b.**
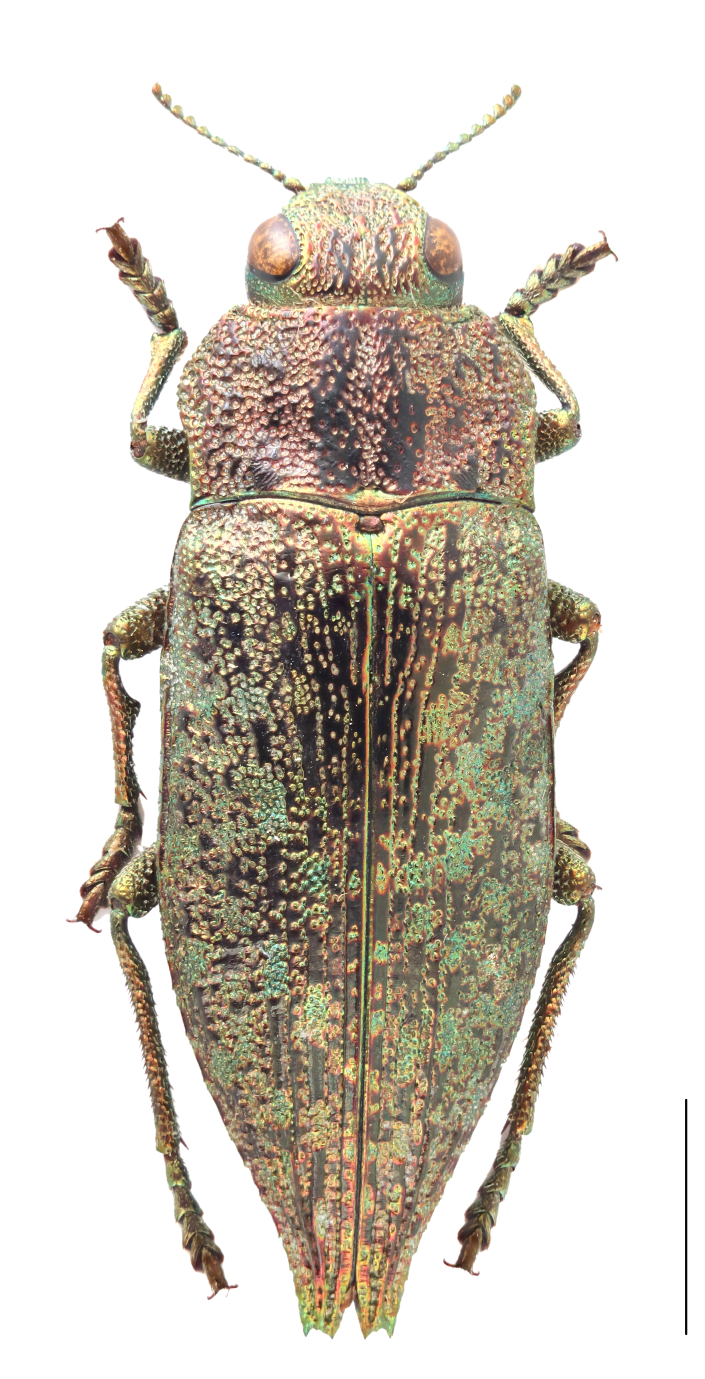
*Dicercaalni* (Fisher von Waldheim, 1824) - scale bar: 4.0 mm;

**Figure 12c.**
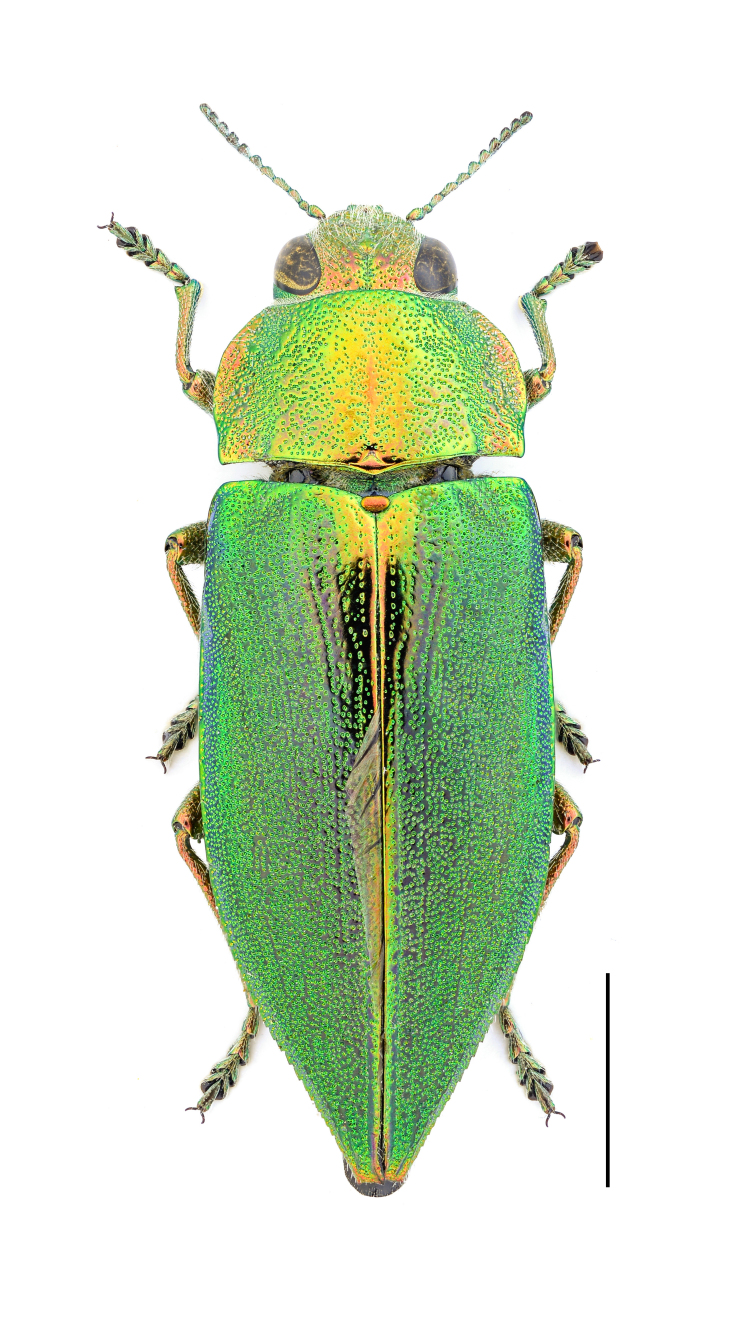
Latipalpisplanassp.plana (Olivier, 1790) - scale bar: 5.0 mm;

**Figure 12d.**
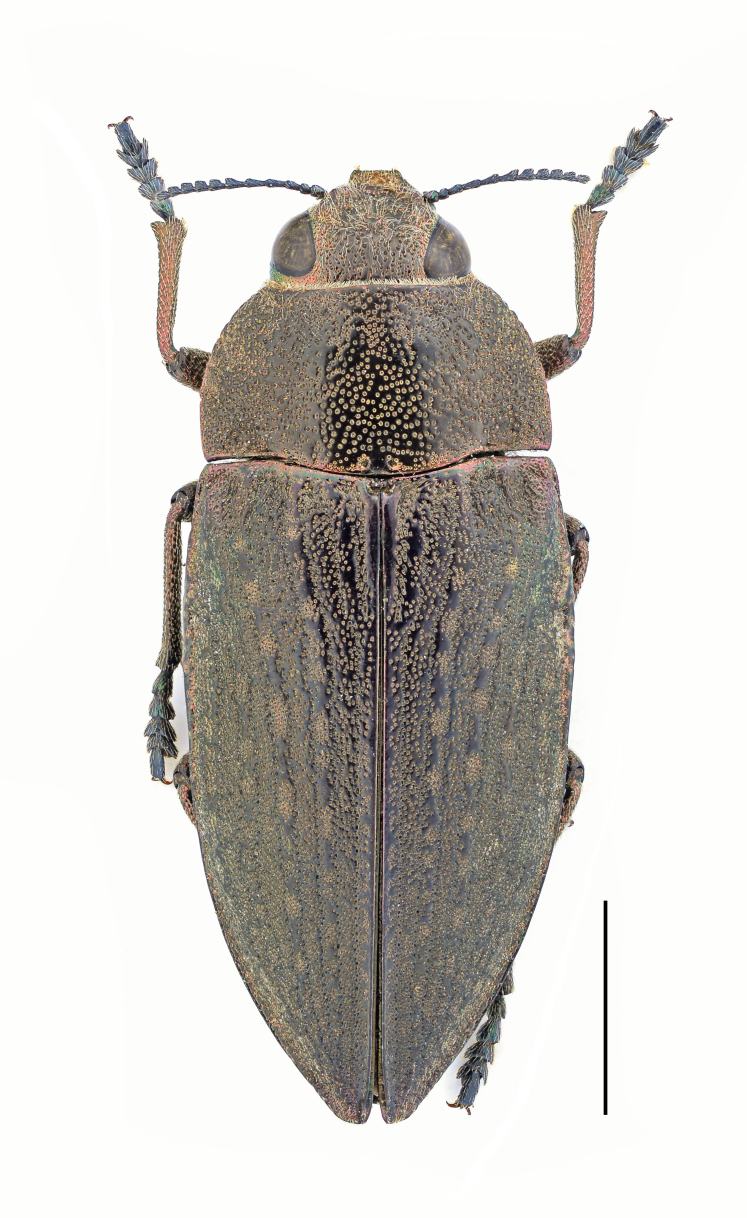
Perotislugubrisssp.lugubris (Fabricius, 1777) - scale bar: 5.0 mm.

**Figure 13a.**
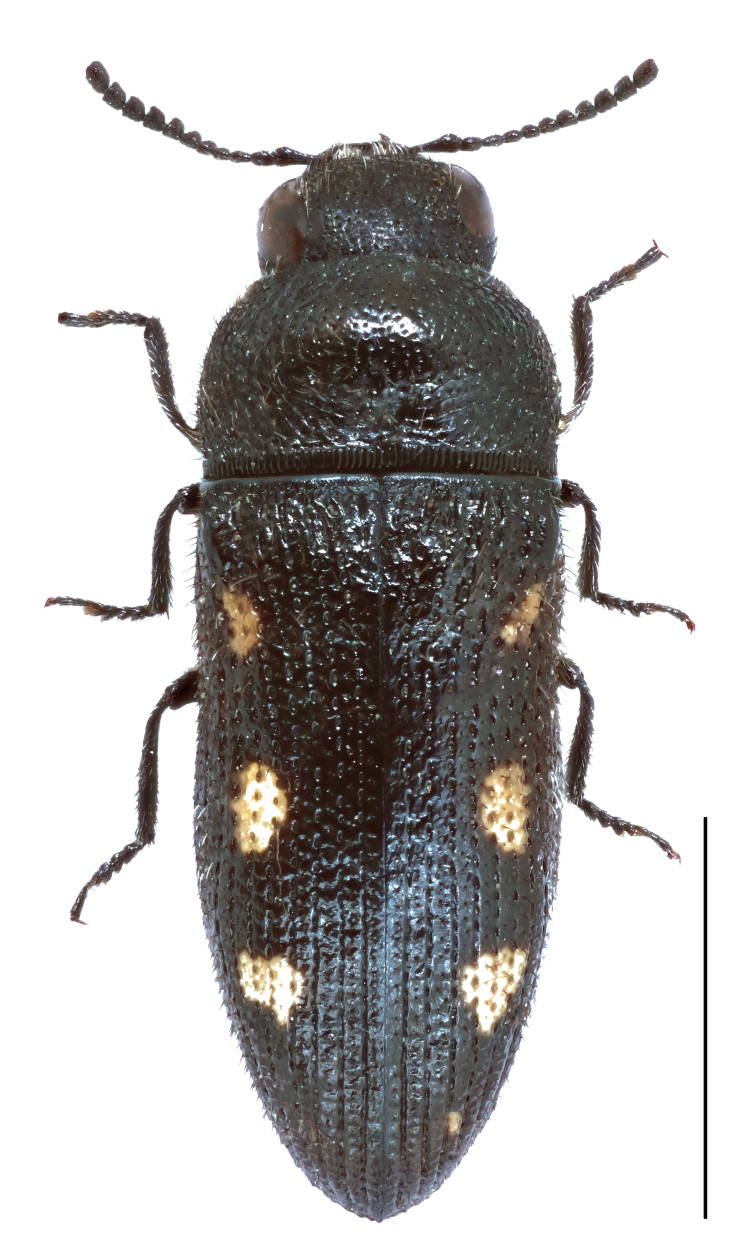
Acmaeodera (Palaeotethya) bipunctata ssp. bipunctata (A. G. Olivier, 1790) - scale bar: 2.0 mm;

**Figure 13b.**
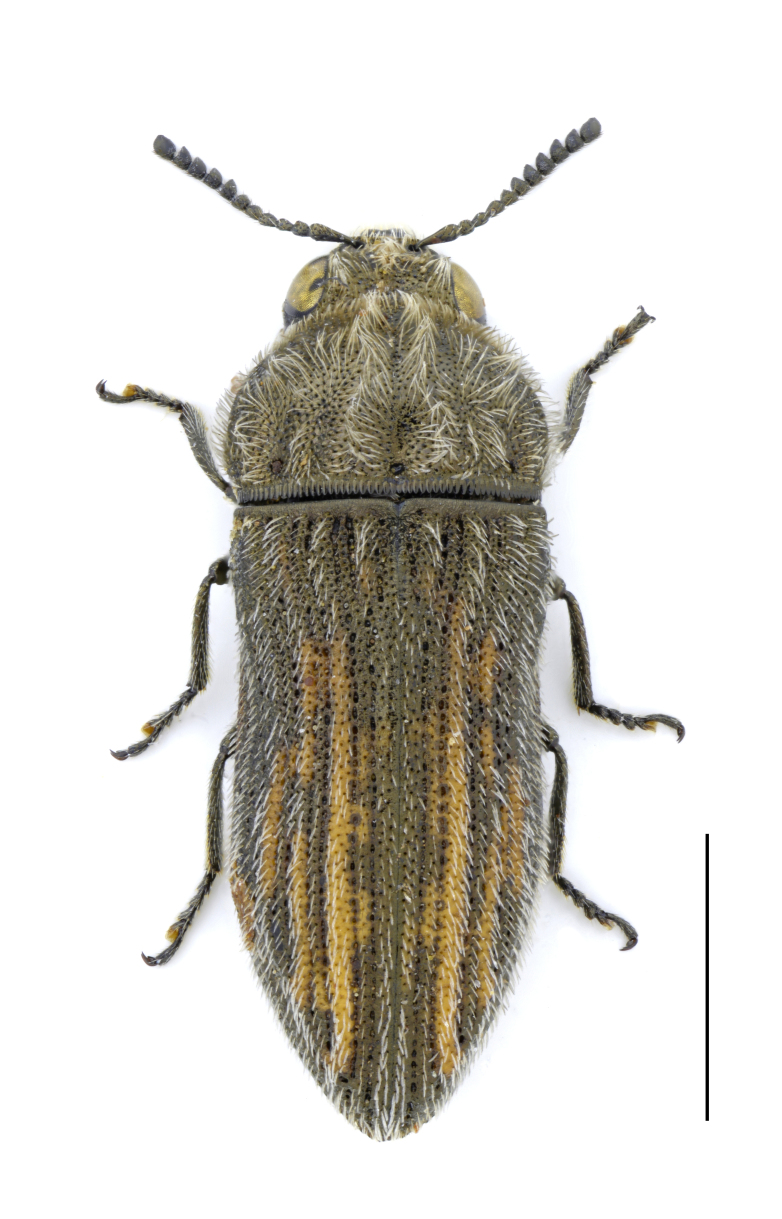
Acmaeoderella (Acmaeoderella) discoida (Fabricius, 1787) - scale bar: 2.0 mm;

**Figure 13c.**
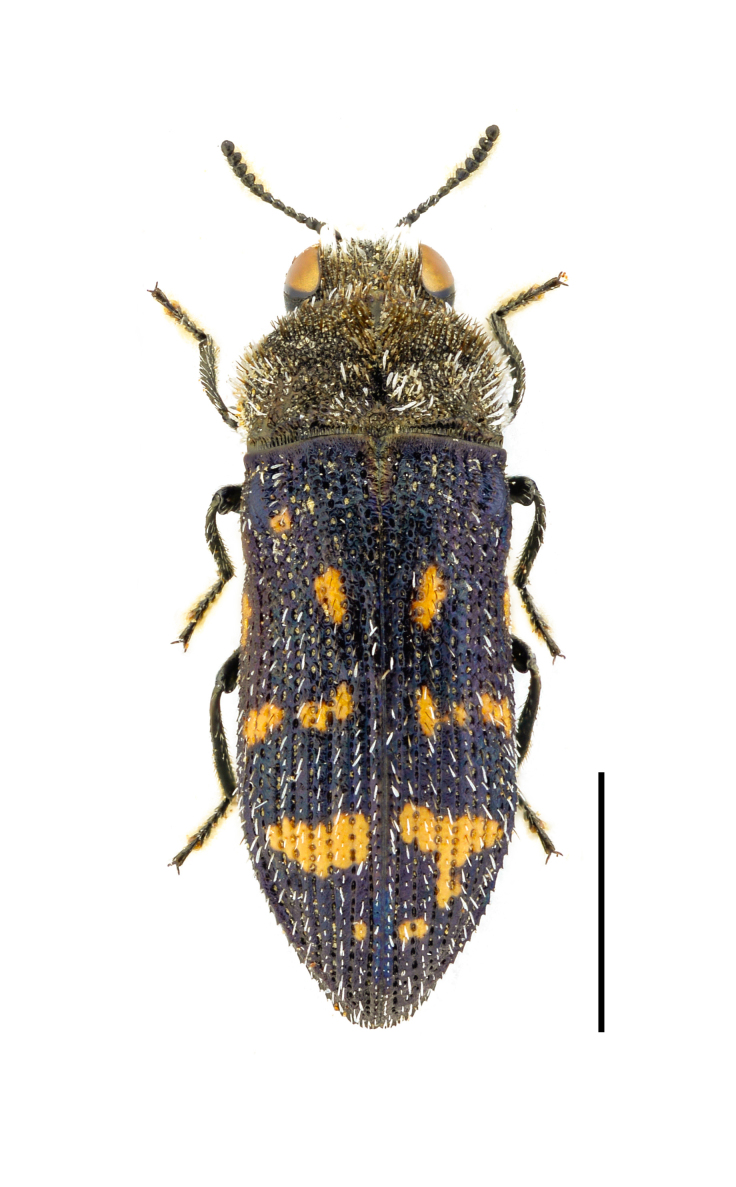
Acmaeoderella (Carininota) flavofasciata ssp. flavofasciata (Piller & Mitterpacher, 1783) - scale bar: 3.0 mm;

**Figure 13d.**
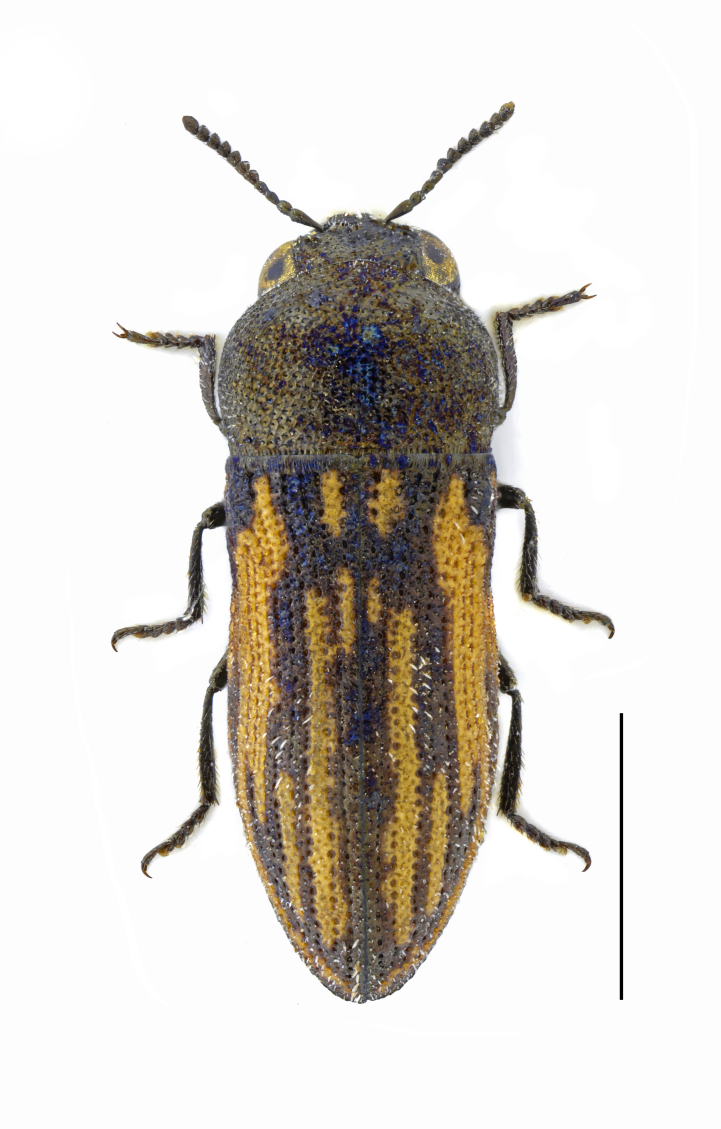
Acmaeoderella (Liogastria) virgulata (Illiger, 1803) - scale bar: 2.0 mm.

**Figure 14a.**
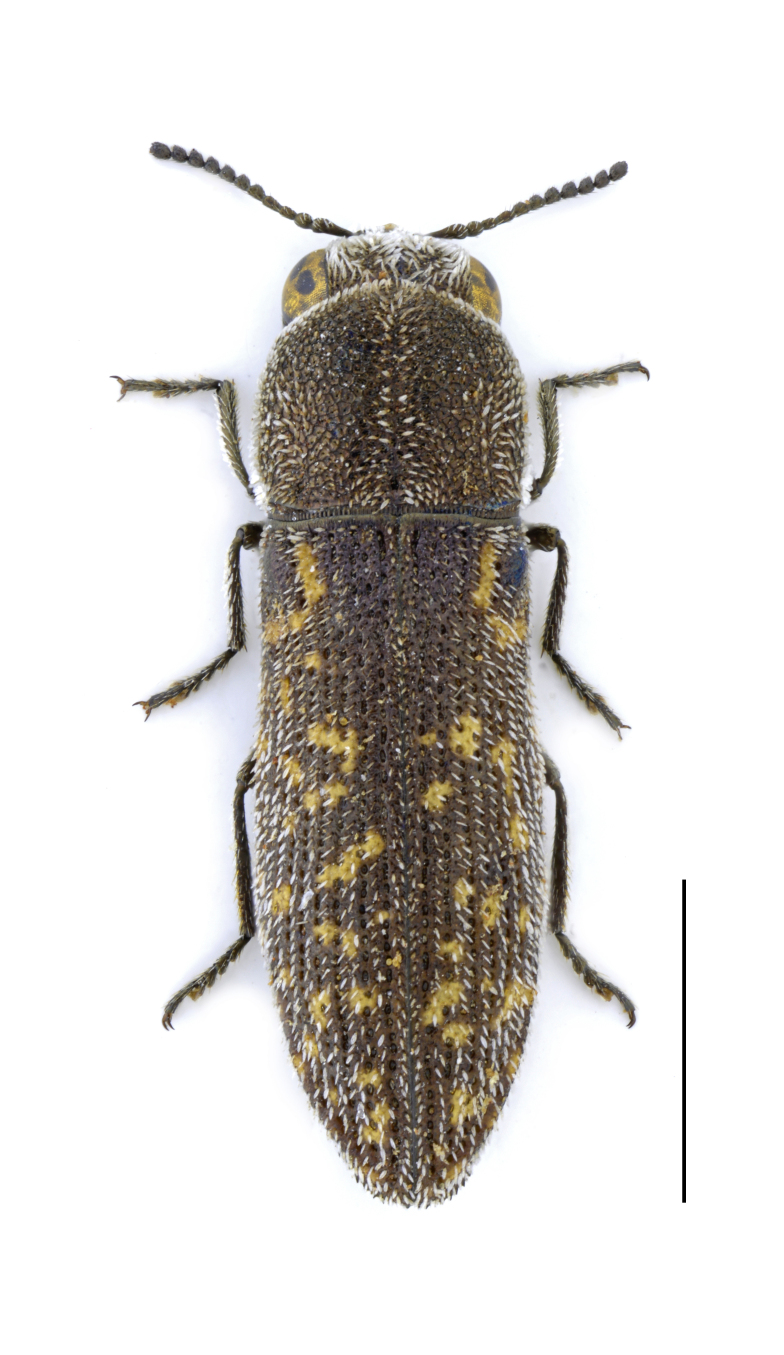
Acmaeoderella (Omphalothorax) adspersula ssp. adspersula (Illiger, 1803) - scale bar: 2.0 mm;

**Figure 14b.**
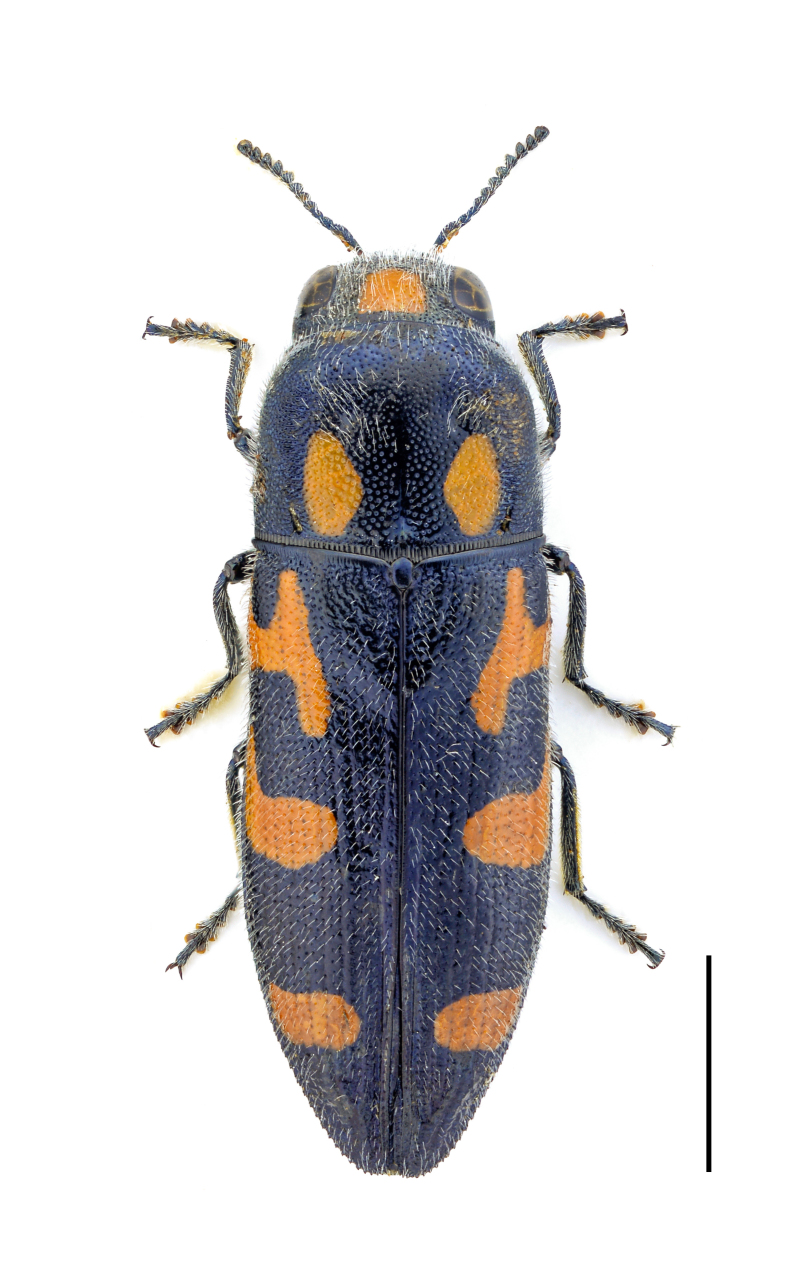
Ptosimaundecimmaculatassp.undecimmaculata (Herbst, 1784) - scale bar: 3.0 mm.

**Figure 15a.**
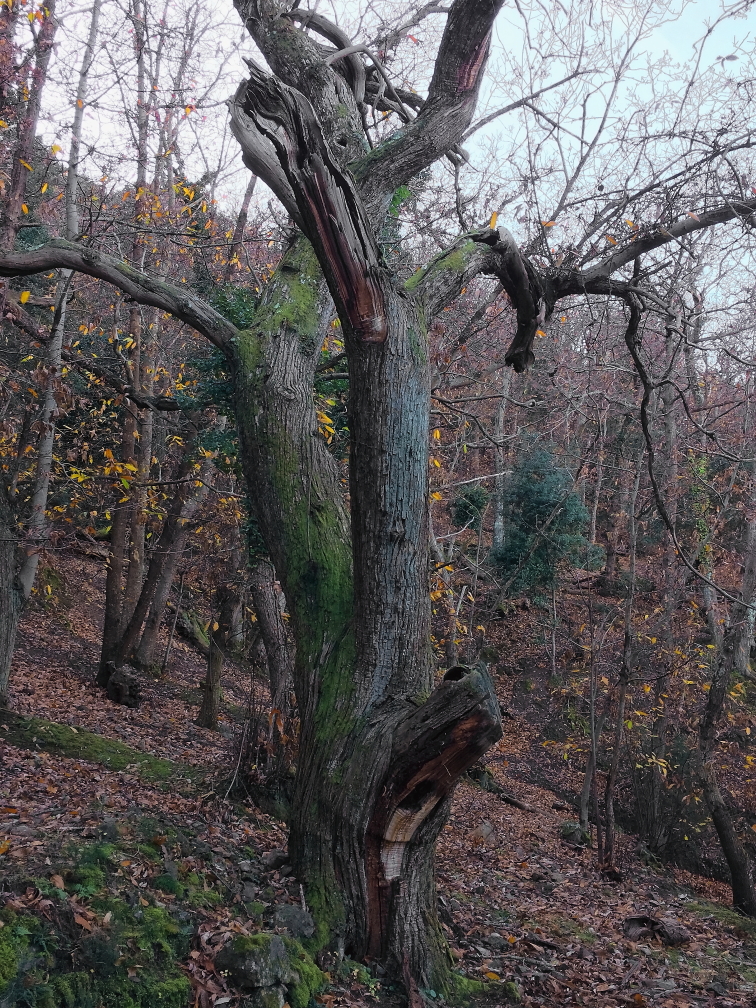
Centuries-old plant of *Castaneasativa* with attacks of *Eurythyreaquercus*;

**Figure 15b.**
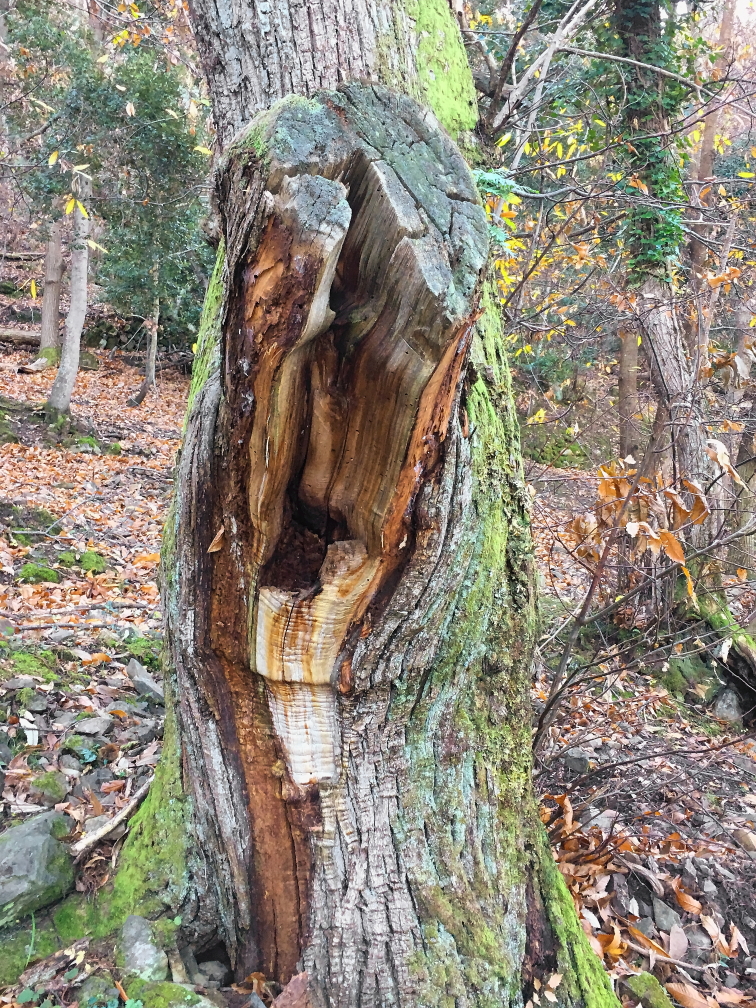
Detail of the trunk presenting emergence holes of *Eurythyreaquercus*.

**Figure 16a.**
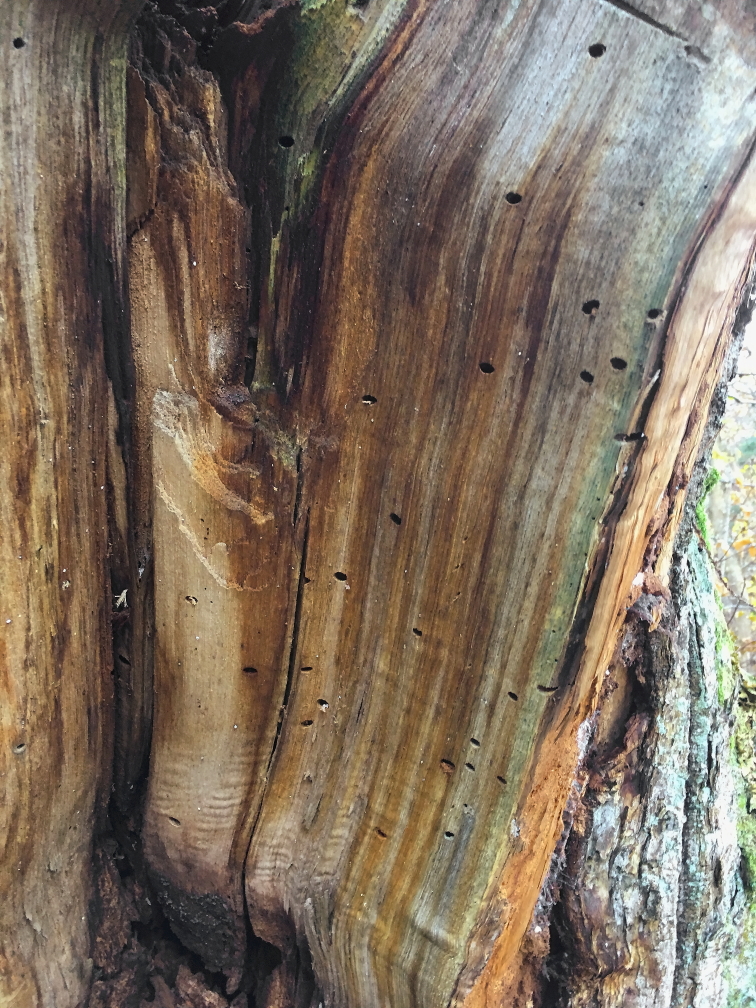
*Eurythyreaquercus* emerging holes, detail;

**Figure 16b.**
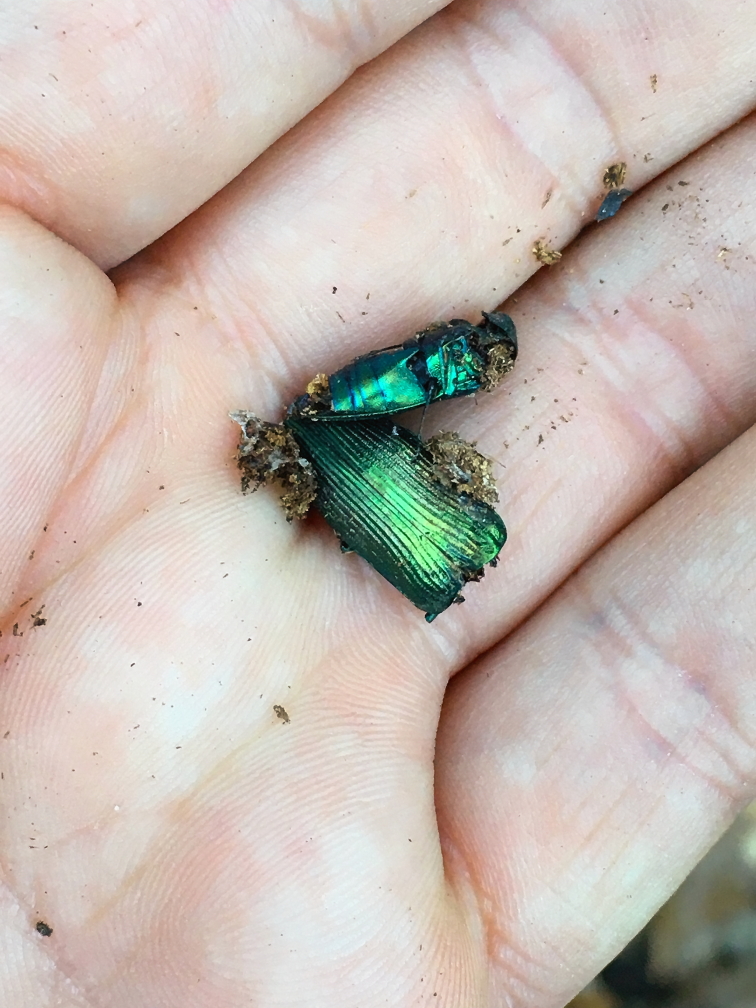
Some of the *Eurythyreaquercus* remains found.

**Table 1.** Presence of species for each island in the Tuscan Archipelago (alphabetical order).

	**Elba**	**Giglio**	**Capraia**	**Pianosa**	**Montecristo**	**Gorgona**
Acmaeodera (*Palaeotethya*) *bipunctatabipunctata*	**X**					
Acmaeoderella (Acmaeoderella) discoida	**X**					
Acmaeoderella (Carininota) flavofasciata flavofasciata	**X**					
Acmaeoderella (Liogastria) virgulata	**X**					
Acmaeoderella (Omphalothorax) adspersula adspersula	**X**	**X**				
*Agrilusangustulusangustulus*	**X**					
*Agriluscuprescens*	**X**					
*Agriluscyanescens*	**X**					
*Agrilusderasofasciatus*	**X**					
*Agriluseleganselegans*		**X**		**X**		
*Agrilusetruscus*	**X**					
*Agrilusevocatus*			**X**			
*Agrilushyperici*	**X**					
*Agrilusintegerrimus*	**X**					
*Agriluslaticornis*	**X**					
*Agrilusmarozzinii*	**X**					
*Agrilusroscidus*	**X**	**X**				
*Agrilussolierisolieri*	**X**					
*Agrilusviridicaerulans* rubi	**X**					
Anthaxia (Anthaxia) chevrieri	**X**					
Anthaxia (Anthaxia) mendizabali	**X**					
Anthaxia (Anthaxia) thalassophila thalassophila	**X**	**X**				
Anthaxia (Haplanthaxia) croesus	**X**	**X**	**X**			**X**
Anthaxia (Haplanthaxia) millefolii polychloros	**X**	**X**				
Anthaxia (Haplanthaxia) umbellatarum umbellatarum	**X**					
Anthaxia (Melanthaxia) nigritula nigritula	**X**					
Aphanisticus (Aphanisticus) elongatus elongatus	**X**					
Aphanisticus (Aphanisticus) pygmaeus	**X**					
Buprestis (Ancylocheira) novemmaculata novemmaculata	**X**	**X**				**X**
*Capnodiscariosacariosa*	**X**					
*Capnodistenebricosatenebricosa*	**X**					
*Capnodistenebrionis*	**X**					
*Chalcophoramassiliensis*	**X**	**X**				
Chrysobothris (Chrysobothris) affinis affinis	**X**					
Chrysobothris (Chrysobothris) solieri	**X**					
*Coraebuselatuselatus*	**X**					
*Coraebusfasciatus*	**X**					
*Coraebusrubi*	**X**					
*Dicercaaeneaaenea*	**X**					
*Dicercaalni*	**X**					
*Eurythyreamicans*	**X**	**X**			**X**	
*Eurythyreaquercus*	**X**					
*Latipalpisplanaplana*	**X**	**X**				
Meliboeus (Meliboeoides) parvulus	**X**					
Meliboeus (Meliboeus) fulgidicollis	**X**					
Meliboeus (Meliboeus) gibbicollis gibbicollis	**X**					
Meliboeus (Meliboeus) graminis graminis	**X**					
*Perotislugubrislugubris*	**X**					
*Ptosimaundecimmaculataundecimmaculata*	**X**					
*Trachyspuncticollisrectilineatus*	**X**					
*Trachystroglodytiformis*	**X**					
